# Application of Lead-Free Metal Halide Perovskite Heterojunctions for the Carbohalogenation of C–C Multiple Bonds

**DOI:** 10.1021/acs.orglett.5c00780

**Published:** 2025-04-01

**Authors:** Camilla Callegari, Costanza Tedesco, Alessia Corbo, Mirko Prato, Lorenzo Malavasi, Davide Ravelli

**Affiliations:** † PhotoGreen Lab, Department of Chemistry, University of Pavia, Viale Taramelli 12, 27100 Pavia, Italy; ‡ Energy and Materials Chemistry Group, Department of Chemistry and INSTM, University of Pavia, Viale Taramelli 16, 27100 Pavia, Italy; § Materials Characterization Facility, Istituto Italiano di Tecnologia, Via Morego 30, 16163 Genova, Italy

## Abstract

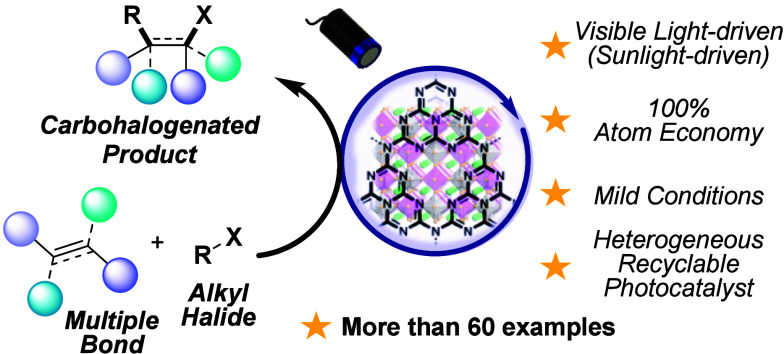

A graphitic carbon nitride/lead-free double perovskite heterojunction (*g*-C_3_N_4_/Cs_2_AgBiCl_6_) has been adopted as a heterogeneous photocatalyst under visible light irradiation. The employed material enabled the atom transfer radical addition-type carbohalogenation of multiple C–C bonds, including (internal) alkenes and alkynes, with alkyl halides. The protocol showed a remarkable functional group tolerance, compatible with the late-stage functionalization of natural and pharmaceutical derivatives, and could be easily scaled up, delivering >1 g of the desired products.

Heterogeneous photocatalysis has emerged as a key strategy for advancing sustainability, offering important technological solutions not only for production of green fuels and pollution mitigation but also toward the development of eco-friendly organic syntheses. These processes harness (solar) light to excite a semiconducting material, which then triggers the transformation of reaction partners, often through redox mechanisms.^1^ Consequently, significant efforts have focused on designing increasingly efficient photocatalytic materials with optimized light absorption, tailored redox potentials, and, most importantly, enhanced lifetimes of photogenerated charge carriers. In this respect, because of their superior optoelectronic properties, metal halide perovskites (MHPs) have recently emerged as suitable and effective semiconductors for performing a series of photocatalyzed reactions. Specifically, MHPs and MHP-based heterojunctions^2^ have been successfully applied in hydrogen generation, nitrogen fixation, CO_2_ reduction, and pollutant degradation.^3^ While such a range of applications are now well consolidated, in recent years there has been increasing interest in the use of MHP photocatalysts for organic chemical transformations. Seminal works were limited to a handful of model reactions, mostly oxidations/reductions; however, the field has now started to express its huge potential in advancing the field of heterogeneous photocatalytic syntheses.^4^

Atom transfer radical addition (ATRA) reactions are versatile transformations that enable the addition of two distinct groups across an unsaturated bond (Scheme 1a).^5^ Notwithstanding the number of diverse approaches available to trigger ATRA chemistry,^6^ progress in the field has been long dominated by catalytic manifolds based on transition metal complexes (e.g., Cu, Ru, and Ni),^7^ with notable applications in polymer chemistry, as well.^7b,8^ Nonetheless, these approaches can face challenges related to the efficient separation of the desired products from the catalyst, limiting their practicality and broader appeal.^7a^

**Scheme 1:**
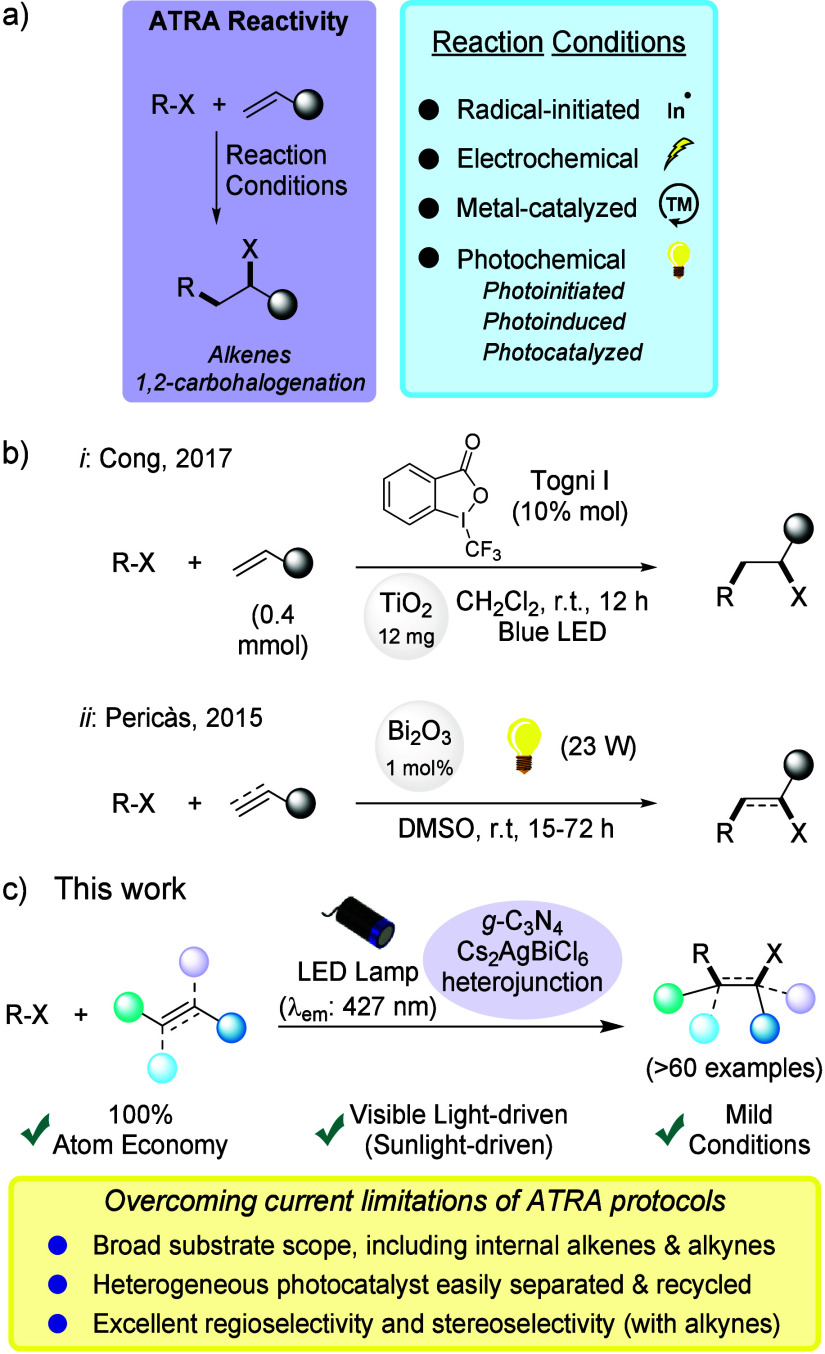
ATRA-Type Reactivity to Trigger the 1,2-Carbohalogenation of Alkenes with Alkyl Halides: (a) Typical Reaction Conditions, (b) Known Manifolds via Heterogeneous Photocatalysis, and (c) This Work

Notably, photochemical approaches^9^ have significantly advanced the field,^10^ fostering the green character of ATRA processes thanks to the mild reaction conditions involved, requiring only (visible) light as the external energy source. However, despite the inherent advantages associated with photocatalyst recovery and recycling, the application of heterogeneous photocatalytic methods to the ATRA-type carbohalogenation of unsaturated systems has been only sparsely reported in the literature,^11,12^ based on traditional metal oxides with poor visible light absorption (Scheme 1b). A prominent example is the work of the Cong group, which utilized titanium dioxide (TiO_2_) to achieve the difunctionalization of unactivated olefins under visible light irradiation. While the protocol performed efficiently, it required the use of an expensive hypervalent iodine(III) reagent (10 mol %) as a co-initiator (Scheme 1b, equation *i*).^13^ Similarly, Riente and Pericàs employed bismuth oxide (Bi_2_O_3_, 1 mol %) as a heterogeneous photocatalyst to promote the reaction between organobromides and functionalized olefins in DMSO (Scheme 1b, path *ii*).^14^ The substrate scope of both studies was limited to terminal olefins with only one example involving a terminal alkyne. Notably, neither study explored the recycling or reuse of the photocatalysts.^13,14^

It is clear that the field of heterogeneous photocatalytic processes for ATRA-type carbohalogenation could strongly benefit from the use of innovative sustainable photocatalysts with wide visible light absorption. To fill this gap, we hereby describe the successful application of MHP-based heterojunctions based on lead-free double perovskite Cs_2_AgBiCl_6_ coupled to nanoexfoliated *g*-C_3_N_4_ to the ATRA-type carbohalogenation of a diverse set of (internal) alkenes and otherwise elusive alkynes^15^ under additive-free conditions (Scheme 1c, this work).

Heterogeneous photocatalysts (**CAT1–6**) employed in the present work have been prepared as previously described.^16^ Briefly, four *g*-C_3_N_4_/Cs_2_AgBiCl_6_ heterojunctions were synthesized with different weight ratios (%_w/w_) of the two components, comprising 10%_w/w_, 30%_w/w_, 50%_w/w_, and 90%_w/w_ expressed as the relative amount of the perovskite component (**CAT2–5**, respectively); at the same time, the pure semiconductors, nanoexfoliated *g*-C_3_N_4_ (**CAT1**) and Cs_2_AgBiCl_6_ (**CAT6**), have been prepared, and their performance was also evaluated for comparison purposes (see Figure 1a). X-ray photoelectron spectroscopy (XPS) was used to confirm the formation of a heterojunction between the two semiconductors (see section 1.1 of the Supporting Information for characterization of **CAT1–6**). We then moved to study the performance of heterojunctions as heterogeneous photocatalysts in the model ATRA-type reaction between 4-pentenyl benzoate **1a** and bromotrichloromethane **2a** to deliver adduct **3** (see Figure 1b). Extensive screening of diverse experimental parameters allowed us to optimize the reaction conditions (see section 1.3 of the Supporting Information for full details); thus, when **1a** (0.1 mmol) and **2a** (2 equiv) were irradiated for 24 h in the presence of the chosen material (**CAT**), product **3** was obtained in up to 90% yield, according to NMR analysis (Figure 1b). In particular, **CAT2** delivered the best performance among all of the screened materials, in both neat and aqueous acetonitrile, in which it offered the highest yield of the desired product **3**. As a general trend, all of the heterojunctions outperformed the pure materials in neat MeCN, while the presence of water was detrimental for photocatalysts containing a large fraction of perovskite. At the same time, **CAT2** offered the best performance upon irradiation in the violet region. Finally, control experiments performed in the absence of any **CAT**, in the dark, or under thermal conditions led to no formation of **3**, clearly demonstrating the photocatalytic nature of the described transformation; at the same time, adopting a *g*-C_3_N_4_/Cs_2_AgBiCl_6_ physical mixture as a photocatalyst led to diminished performance (see Figure 1b and Table S7).

**Figure 1:**
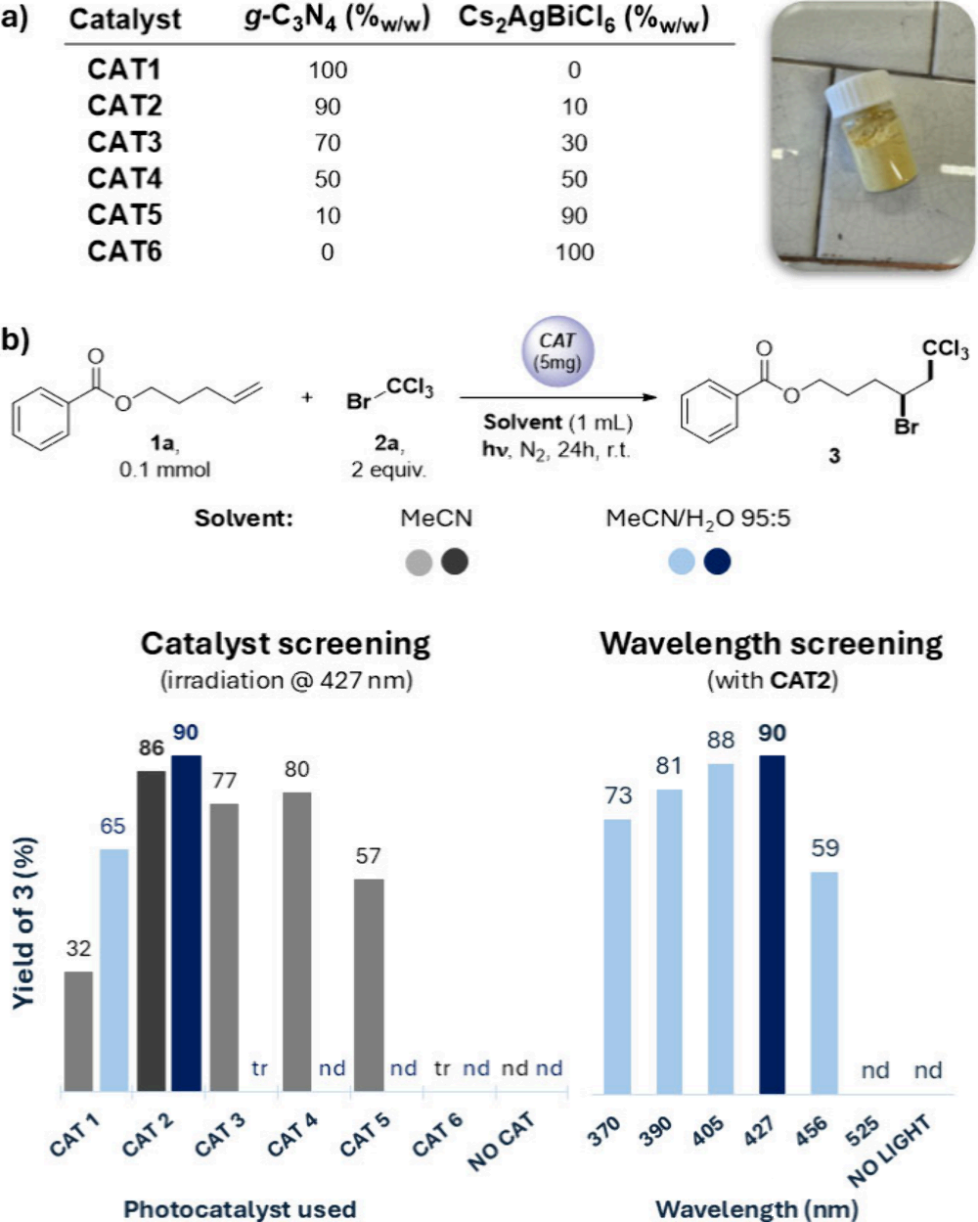
(a) Catalysts (**CAT**) employed in the present work. (b) Screening of selected conditions for the model ATRA-type reaction between 4-pentenyl benzoate (**1a**) and bromotrichloromethane (**2a**); NMR yields are reported (see general procedure GP-*a* in section 3.1 of the Supporting Information for further details). tr, traces; nd, not detected.

With the optimized conditions in hand, the next step was to evaluate the scope, versatility, and robustness of the ATRA-type difunctionalization of unsaturated substrates with alkyl halides in a preparative setting (0.5 mmol (Table 1); the complete list of starting materials is available in section 1.2 of the Supporting Information, along with relevant scope limitations). We initially evaluated the substrate scope in terms of alkyl halides and demonstrated that our model alkene **1a** can be smoothly converted into products **3**–**11** containing a range of different functional groups, including halogenated moieties, ester, nitrile, ketone, and nitro groups. On the other hand, the reaction did not work with diethyl chloromalonate (**2l**) and ethyl α-bromoisobutyrate (**2m**, a known radical initiator in polymerizations).^17^

**Table 1:**
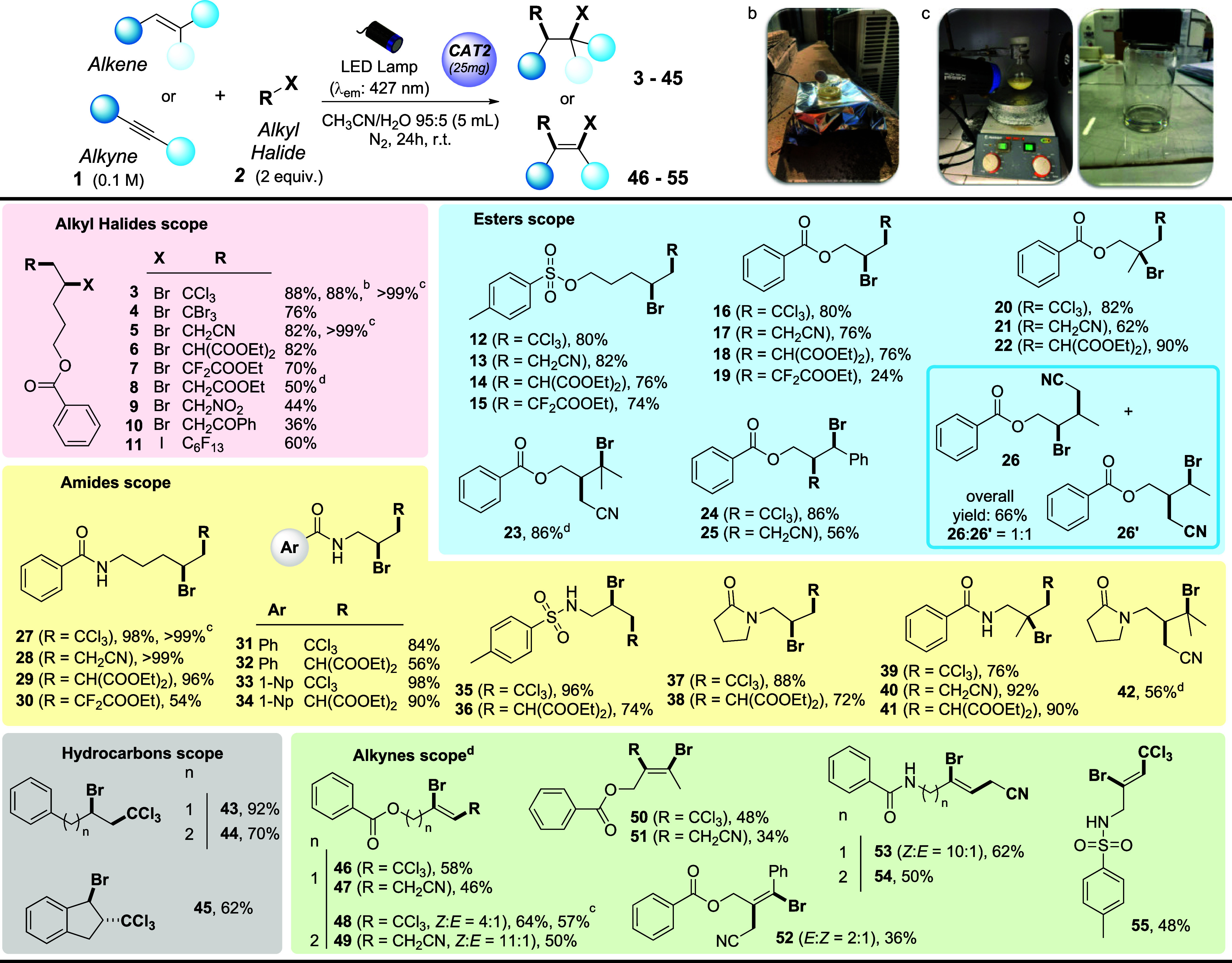
Scope of the Difunctionalization of Alkenes and Alkynes via Photocatalyzed ATRA Processes^a^

a For the reactions, alkenes or alkynes **1** (0.5 mmol, 1 equiv) and alkyl halides **2** (1 mmol, 2 equiv) in 95:5 CH_3_CN/H_2_O (5 mL, 0.1 M) in the presence of **CAT2** (25 mg) were irradiated with a 427 nm LED lamp (40 W) at room temperature for 24 h. Yields of the isolated product after column chromatography on silica gel (cyclohexane/ethyl acetate mixture as the eluant) have been reported (see general procedure GP-*b* in section 3.1 of the Supporting Information for further details). 1-Np = 1-naphthyl.

b Reaction performed under sunlight irradiation (irradiation for 16 h over 2 days).

c Gram scale reaction (3 mmol; see general procedure GP-*c* in section 3.1 of the Supporting Information for further details).

d Reaction performed using 2 mmol (4 equiv) of alkyl halide.

Next, we modified the scaffold of the ester substrate containing the alkene by varying the ester type (see sulfonate products **12–15** obtained in up to 82% isolated yield), chain length (products **16**–**19**), and substitution pattern of the olefin (products **20**–**26**). As for the latter aspect, a regioselective process has been consistently observed, let apart the process leading to **26**/**26′**, which delivered a ∼1:1 regioisomeric mixture. In all of the other cases, installation of the halogen atom took place on the most substituted C atom of the starting olefin (see the case of **20**–**23**) or at the benzylic position (see the case of **24** and **25**) in the case of styrene-type starting olefins. We also appended the alkene substrate to an amide scaffold and ran a series of ATRA-type difunctionalizations by considering the preparation of diversely functionalized aromatic amides (see products **27–34**, up to quantitative yield obtained) and sulfonamides (**35** and **36**), as well as lactams (**37** and **38**). Also in this case, the regiochemistry in the functionalization of polysubstituted olefins followed the same trend observed in the case of esters (see adducts **39–42**). We also tested the reactivity of a set of unsaturated hydrocarbons, which allowed the preparation of adducts **43–45** in good to excellent isolated yields in a manner independent of the linear or cyclic nature of the substrate. After validating the versatility of the proposed method with alkenes, the scope was broadened to include alkynes as unsaturated reaction partners. Such substrates have been combined with selected alkyl bromides, leading to the corresponding functionalized vinyl bromides with complete regioselectivity and good to excellent levels of stereoselectivity (see adducts **46**–**55**). This investigation revealed that both terminal and internal alkynes are competent substrates, although the latter allowed us to obtain the products of interest only in modest yields (34–48%).

The preparation of **3** was also performed under sunlight irradiation (exposure for 16 h over 2 days needed; 88% isolated yield), while scaling up the reaction (3 mmol) was not a problem, as shown by compounds **3**, **5**, and **27**, consistently isolated in quantitative yield on a gram scale. The importance of the synthesized adducts is also confirmed by their further manipulation, as showcased by the preparation of a carboazidated product from **5** (full details are reported in section 3.3 of the Supporting Information). Having confirmed the stability of **CAT2** after catalysis (see section 1.4 of the Supporting Information), the potential of the method was further evaluated through recycling tests, which demonstrated sustained catalytic performance in the preparation of **3** over four recovery cycles, with yields remaining as high as 88–90% (for additional details, see section 1.5 of the Supporting Information).

Finally, the late-stage functionalization of natural and pharmaceutical derivatives by means of our ATRA-type protocol has been attempted (Table 2). Thus, our strategy allowed functionalization of eugenol, as well as derivatives of natural alcohols (−)-menthol and citronellol (adducts **56–60**, 34–68% isolated yields). At the same time, a pendant alkene moiety has been introduced at the carboxylic acid group in nonsteroidal anti-inflammatory drugs (NSAIDS) fenbufen and ibuprofen and amino acid l-phenylalanine, delivering products **61–65** in good to excellent yields (quantitative in the case of adduct **63**).

**Table 2:**
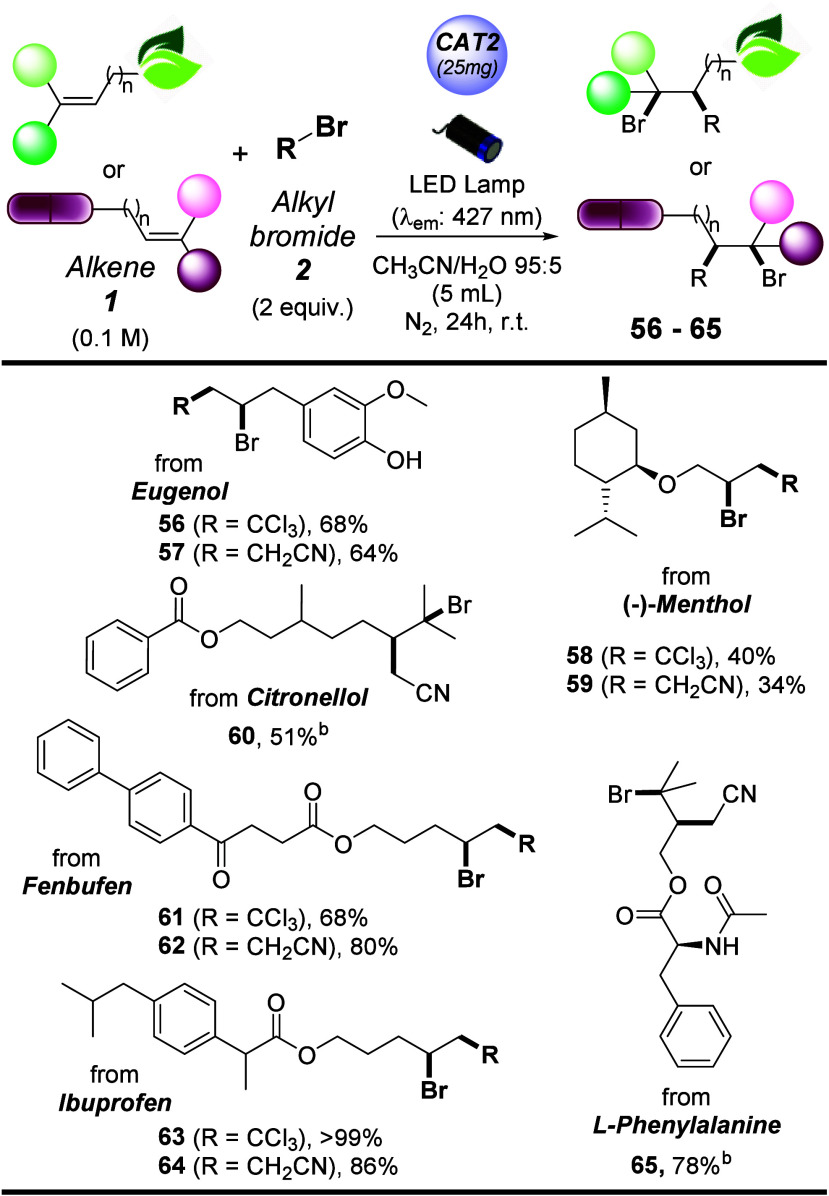
Late-Stage Functionalization of Natural and Pharmaceutical Derivatives via Photocatalyzed ATRA Processes^a^

a See footnote a of Table 1.

b Reaction performed using 2 mmol (4 equiv) of alkyl halide.

Next, mechanistic studies were conducted to gain insights into the elementary steps of the hereby reported ATRA-type transformation (Scheme 2a; see section 1.6 of the Supporting Information for further details). A significant decrease in the yield of compound **3** was observed upon addition of radical scavenger 2,2,6,6-tetramethylpiperidine *N*-oxyl (TEMPO), with a complete suppression of the reactivity in the presence of 3 equiv of TEMPO. To further support the involvement of a radical pathway, substrate **1ah** was subjected to optimized reaction conditions. Upon reaction with CCl_3_Br, such *N*-tosylated diene resulted in the clean formation of product **66** (80% yield) through a 5-*exo*-trig radical cyclization process.^18^

**Scheme 2:**
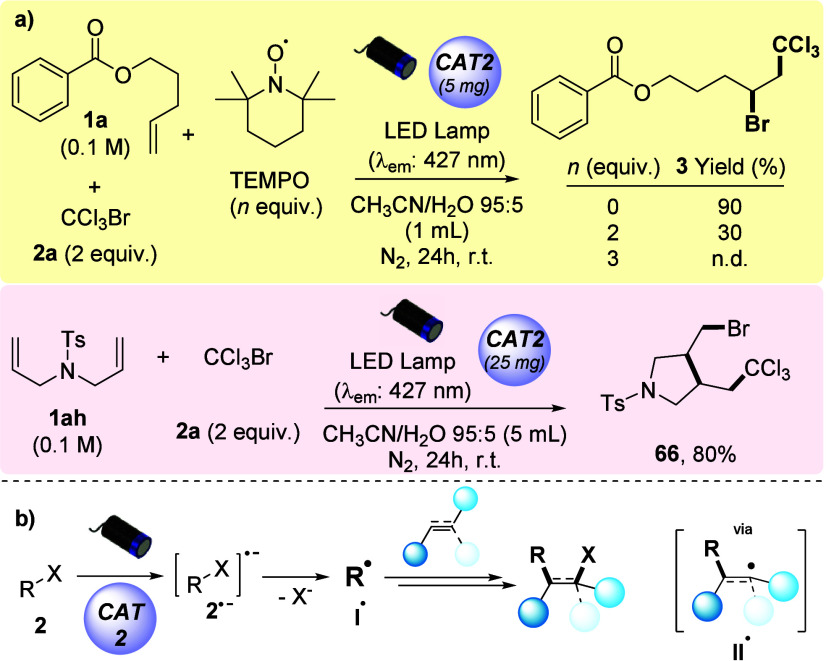
Mechanistic Considerations Experiments supporting a radical pathway. Simplified mechanistic scenario for the photocatalyzed ATRA-type carbohalogenation of alkenes and alkynes.

From a mechanistic standpoint (see Scheme 2b), in accordance with the redox potential of the *g*-C_3_N_4_/Cs_2_AgBiCl_6_ heterojunction catalyst (estimated *E*_CB_ of −1.56 V vs SCE),^19^ we propose that the employed alkyl halides (**2**; general formula R–X) undergo single-electron reduction by the photoexcited electrons in the conduction band.^20^ This delivers a radical anion species (**2**^**•–**^) that subsequently generates a reactive C-centered radical (**I**^**•**^) upon halide anion loss (X^–^). The thus formed organoradical **I**^**•**^ then adds to the unsaturated π-trap to form radical adduct **II**^**•**^ (an alkyl- or vinyl-type^21^ radical intermediate), which then kicks off a radical chain process involving a new equivalent of the starting alkyl halide.^10^ Full rationalization of the observed selectivities in the carbohalogenation of alkenes and alkynes is offered in section 1.6.3 of the Supporting Information.

Overall, this work presents a general and versatile heterogeneous photocatalytic protocol that utilizes a sustainable carbon nitride/lead-free double perovskite heterojunction, improving the charge carrier dynamics relative to the isolated semiconductors. The method facilitates ATRA reactions, enabling the carbohalogenation of alkenes, and even alkynes, with a wide range of alkyl halides. This protocol takes place under mild conditions, does not require any additives, and can be easily run on a gram scale while being fully compatible with the late-stage functionalization (LSF) of natural and pharmaceutical derivatives. The extended absorption features of the employed heterojunctions enable the reaction to smoothly take place under natural sunlight irradiation conditions, while the robustness of the material allows the photocatalyst to be recycled for multiple cycles without performance loss.

## Data Availability

The data underlying this study are available in the published article and its Supporting Information.

## Supporting Information Available

Detailed experimental procedures, characterization data, and NMR spectra for all new compounds (PDF)
