# Supplementary material for: Application of Lead-Free Metal Halide Perovskite Heterojunctions for the Carbohalogenation of C–C Multiple Bonds
Source: Org Lett. 2025 Apr 1;27(14):3667–72. doi: 10.1021/acs.orglett.5c00780 (PMC11998073; doi:10.1021/acs.orglett.5c00780)

Supporting information for

Application of Lead-Free Metal Halide Perovskite Heterojunctions for the Carbohalogenation of C–C Multiple Bonds

Camilla Callegari,[a] Costanza Tedesco,[b] Alessia Corbo,[a]

Mirko Prato,[c] Lorenzo Malavasi,[b,*] Davide Ravelli[a,*]

[a] *PhotoGreen Lab, Department of Chemistry, University of Pavia, Viale Taramelli 12, 27100 Pavia (PV), Italy. E-mail:* davide.ravelli@unipv.it, www.unipv.it/photogreenlab

[b] *Energy and Materials Chemistry Group, Department of Chemistry and INSTM, University of Pavia, Viale Taramelli 16, 27100 Pavia (PV), Italy. E-mail:* lorenzo.malavasi@unipv.it

[c] *Materials Characterization Facility, Istituto Italiano di Tecnologia, Via Morego 30, 16163 – Genova, Italy*

TABLE OF CONTENTS (176 pages)

1. Data cited in the main text p. S2

- **1.1 Catalysts characterization** p. S2
- **1.2 Chart of starting materials** p. S7
- **1.3 Optimization of reaction conditions** p. S10
- **1.4 *post*-Catalysis characterization** p. S14
- **1.5 Catalyst recovery** p. S16
- **1.6 Mechanistic investigations** p. S17

2. Experimental details p. S23

- **2.1 General information** p. S23
- **2.2 General procedure for the synthesis of the catalyst (GPC)** p. S25
- **2.3 Synthesis of starting materials (GP-I – GP-VI)** p. S27
- **2.4 Characterization of starting materials** p. S30

3. Photocatalyzed ATRA reactions p. S41

- **3.1 General procedures for photocatalyzed ATRA reactions (GP-*a* – GP-*c*)** p. S41
- **3.2 Characterization data for products 3-66** p. S44
- **3.3 Products *post*-functionalization** p. S76

4. References p. S77

5. Copy of ^{1}H and ^{13}C NMR spectra p. S80

1. Data cited in the main text

1.1 Catalysts characterization

We previously reported the synthesis of *g*-C_3N_4/$Cs_2AgBiCl_6$ heterojunctions and consistently adopted the preparation protocol available in the literature; for the sake of clarity, section 2.2 details the adopted experimental procedure (**GPC**; see below).[S1] The following weight ratios between nano-exfoliated *g*-C_3N_4 and $Cs_2AgBiCl_6$ have been investigated: 0, 10, 30, 50, 90 and 100 (these values do refer to the %$_{W/W}$ of perovskite in the material; see also **Figure 1a** in the main text).

The characterization data of such materials (X-ray diffraction patterns and UV-Vis absorption spectra) have been previously reported in the literature and are reported here for the sake of completeness.[S1]

Thus, **Figure S1a** reports the room temperature powder X-ray diffraction (PXRD) patterns of nano-exfoliated *g*-C_3N_4 (**CAT1**; upper portion), the complete series of the prepared *g*-C_3N_4/$Cs_2AgBiCl_6$ heterojunctions (**CAT2-5**), and pure $Cs_2AgBiCl_6$ (**CAT6**; lower portion). As for the pattern of **CAT1**, two main peaks, around 13° and 28°, are clearly identified and are attributed to the (100) and (002) reflections; as an inset (top left), the crystal structure of $Cs_2AgBiCl_6$ is depicted, displaying a cubic symmetry with $Fm\bar{3}m$ space group (#225) and a lattice parameter of 10.7761(2) Å, as previously reported.[S1]

Figure S1b reports the UV-Vis spectra of nano-exfoliated *g*-C_3N_4 (**CAT1**), the complete series of the prepared *g*-C_3N_4/$Cs_2AgBiCl_6$ heterojunctions (**CAT2-5**), and pure $Cs_2AgBiCl_6$ (**CAT6**). As for the **CAT2-5** heterojunctions, they all show a strong absorption at the edge between UV and visible light, with a tail extending up to *ca*. 600 nm. As a rule of thumb, it can be concluded that the composites show an absorption spectrum similar to the most abundant component.[S1]

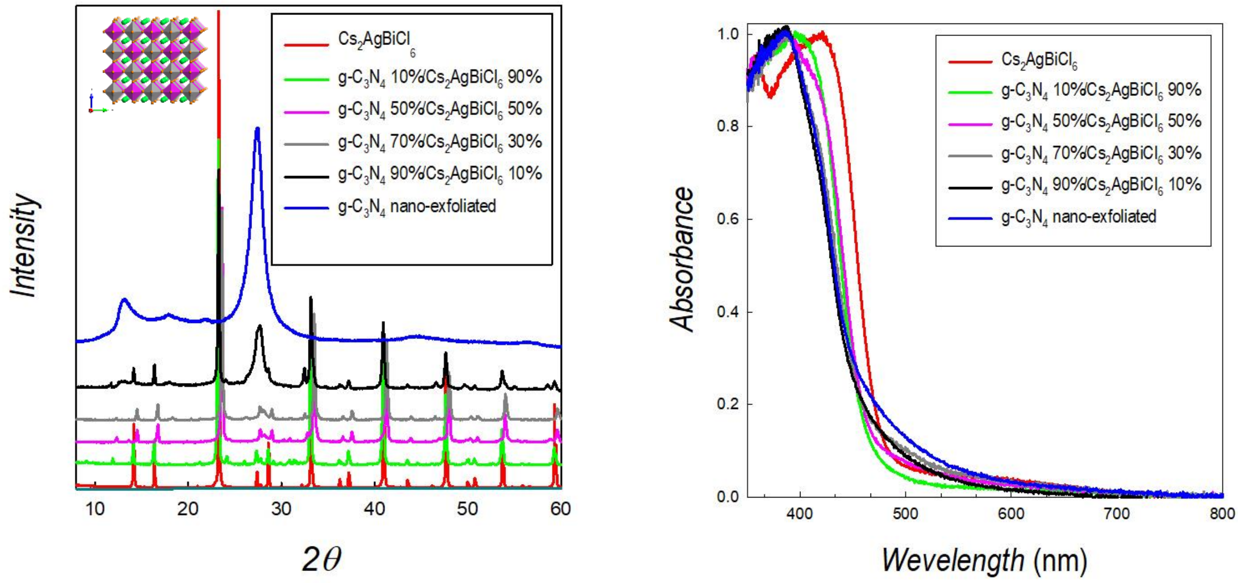

Figure S1. Structural and optical characterization of *g*-C_3N_4/$Cs_2AgBiCl_6$ heterojunctions and the pure semiconductors: a) PXRD patterns; b) UV-Vis absorption spectra. The inset in panel a shows $Cs_2AgBiCl_6$ crystal structure, where Cs^+ ions are shown as green spheres, the chloride ions as orange spheres, while the Ag and Bi octahedra are shown as silver and purple polyhedra, respectively. Adapted from Ref. S1 (Creative Commons CC BY license).

Morphological characterization of the best performing heterojunction (**CAT2**, namely *g*-C_3N_4/$Cs_2AgBiCl_6$ 90:10) has been carried out by Scanning Electron Microscopy (SEM) and is reported in **Figure S2** (the same image has already been reported in a previous work and is duplicated here for the sake of completeness).[S1]

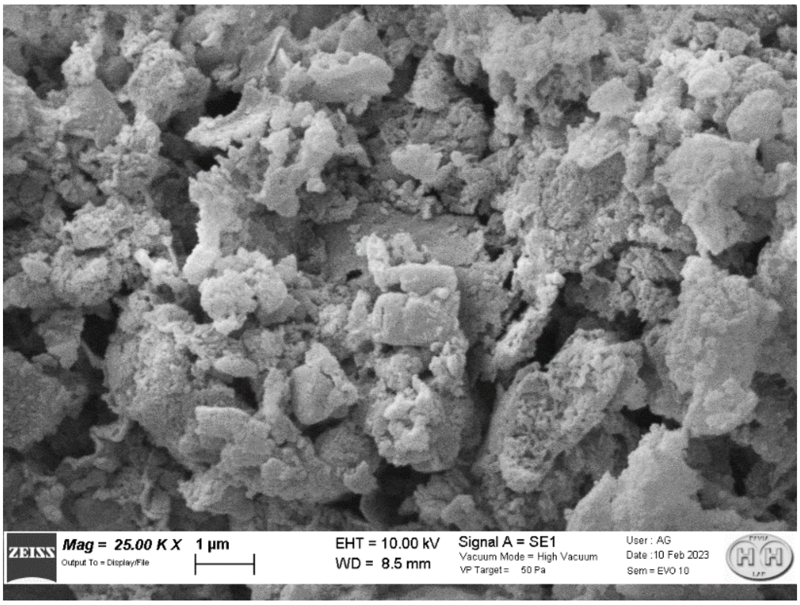

Figure S2. Representative SEM image of **CAT2**, namely *g*-C_3N_4/$Cs_2AgBiCl_6$ 90:10. Reproduced from Ref. S1 (Creative Commons CC BY license).

X-ray Photoelectron Spectroscopy (XPS) was used to study the surface chemistry of **CAT2** catalyst, and compare it to that of the composing materials, namely *g*-C_3N_4 and $Cs_2AgBiCl_6$. XPS specimens were prepared by pressing a few mg of the powder samples onto high purity indium pellets (99.99% purity).

XPS data were acquired using a Kratos Axis Ultra^{DLD} spectrometer, equipped with a monochromatic Al Kα source operated at 20 mA and 15 kV. Wide scans were acquired at a pass energy of 160 eV, energy step of 1 eV, over an analysis area of 300 × 700 μm^2. High-resolution spectra were then acquired at a pass energy of 10 eV, energy step of 0.1 eV, over the same analysis area, with a specific focus on the main signals from the elements composing the materials, namely Cs 3d, Ag 3d, Bi 4f, Cl 2p, C 1s, and N 1s. The Kratos charge neutralizer system was used on all specimens; spectra have been charge-corrected, setting the lowest binding-energy component of the carbon 1s spectrum to 284.8 eV.

The collected data were then analyzed with CasaXPS software (version 2.3.24).[S2]

Comparison of XPS wide scans

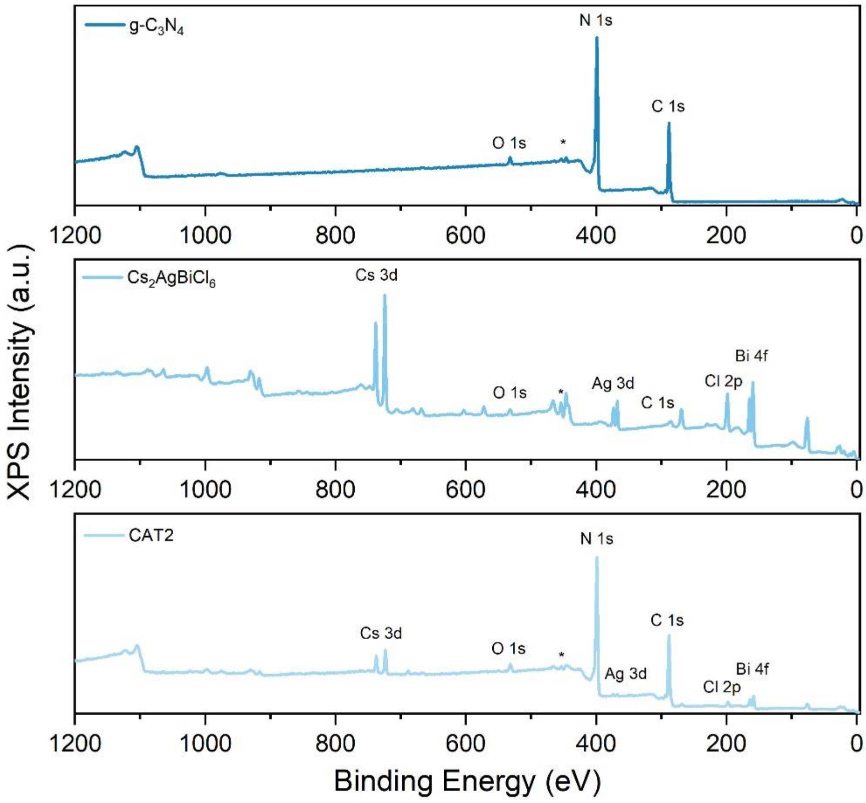

Figure S3: Comparison of the wide scans collected on the three analyzed samples – from top to bottom: *g*-C_3N_4, $Cs_2AgBiCl_6$, and **CAT2** catalyst. The asterisks mark peaks coming from the In substrate.

Figure S3 shows the wide scans collected on the three samples. The main XPS peaks are labelled with the element and orbital the photoelectrons are coming from. As expected, the spectrum collected on *g*-C_3N_4 shows intense C 1s and N 1s peaks, and a low intensity O 1s peak, that might come from surface contamination by adventitious species. The absence of peaks from other elements certifies the high purity of the nanoexfoliated *g*-C_3N_4. Similarly, all the expected signals are visible in the spectrum collected on the lead-free double perovskite $Cs_2AgBiCl_6$ material. Also in this case, a low intensity O 1s peak is visible in the spectrum. The spectrum collected on the *g*-C_3N_4/$Cs_2AgBiCl_6$ heterostructure (**CAT2**) displays signals from both the constituent materials. As expected, taking into consideration the used *g*-C_3N_4/$Cs_2AgBiCl_6$ w/w ratio, the perovskite-related peaks appear extremely reduced in intensity, as the XPS peaks intensity is related to the amount of the elements the signals are originated from.

Comparison of XPS high-resolution spectra

Figure S4 shows the high-resolution spectra collected on the three samples. The nanoexfoliated *g*-C_3N_4 sample is characterized by intense N 1s and C 1s signals, with main peaks centered at (399.0±0.2) eV and (288.6±0.2) eV, respectively, associated to pyridinic N and to the carbons in the N−C=N moieties, in line with reports on related systems.[S3,S4]

The spectra collected on the $Cs_2AgBiCl_6$ perovskite material show peaks centered at binding energy values here summarized:

Cs $3d_{5/2}$ @ (724.5±0.2) eV

Ag $3d_{5/2}$ @ (367.9±0.2) eV

Bi $4f_{7/2}$ @ (159.6±0.2) eV

Cl $2p_{3/2}$ @ (198.2±0.2) eV

consistent with the expected oxidation states for Cs (Cs^+), Ag (Ag^+), Bi (Bi^{3+}), and Cl (Cl^-).[S5]

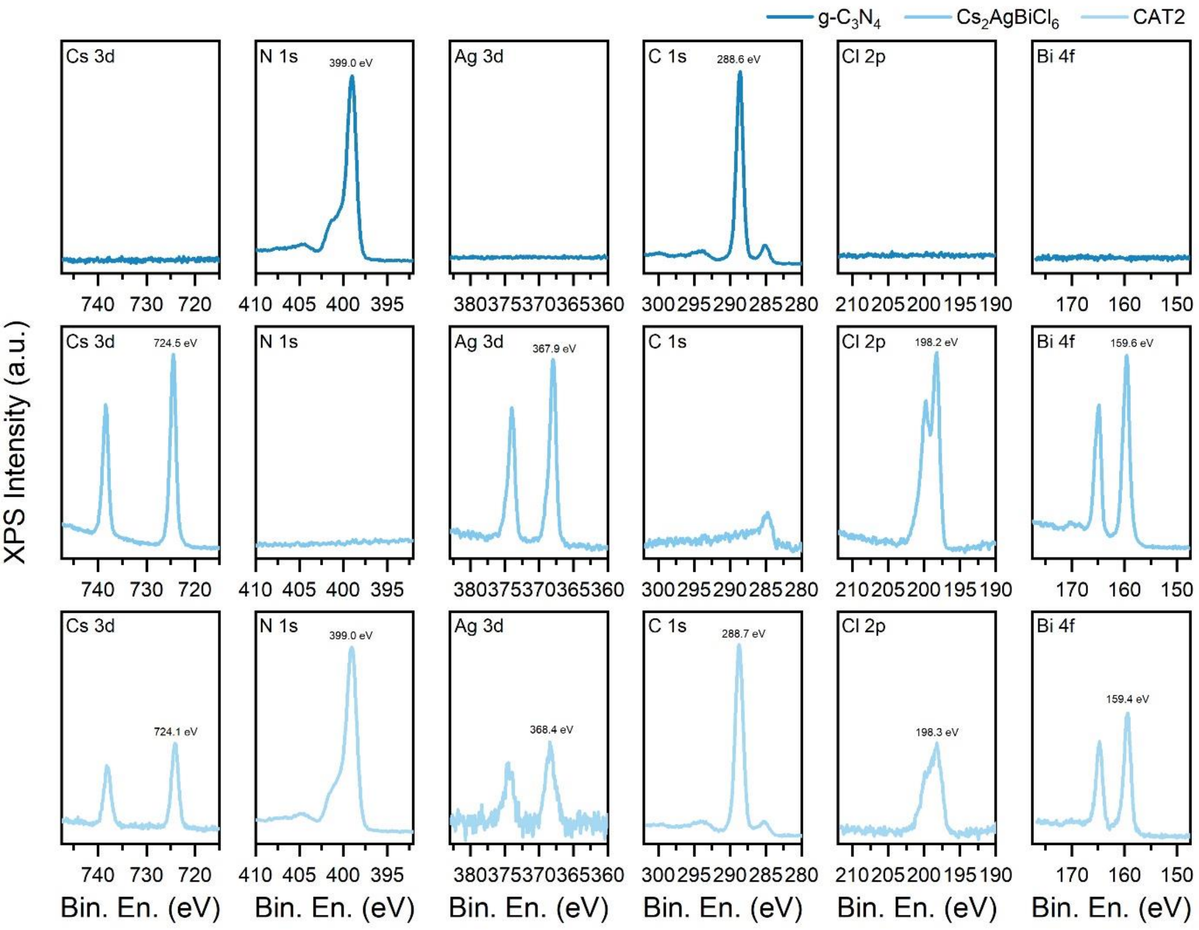

Figure S4: Comparison of the high-resolution spectra collected on the three analysed samples – from top to bottom: *g*-C_3N_4, $Cs_2AgBiCl_6$, and CAT2 catalyst.

The heterostructure sample, **CAT2**, displays signals of both perovskite and graphitic carbon nitride. While the signals from *g*-C_3N_4 do not show significant variations with respect to the pure *g*-C_3N_4 sample, some minor shifts were observed, especially on Cs and Ag related peaks, with respect to the position of the same peaks in the perovskite, suggesting that the interaction with *g*-C_3N_4 might have induced some electronic rearrangements within the perovskite structure.

1.2 Chart of starting materials

Compounds shown in gray color refer to limitations of the substrate scope.

Starting materials (alkenes) used in Table 1

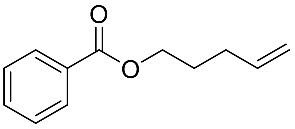

1a

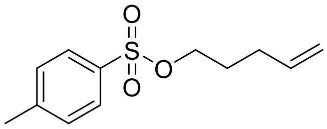

1b

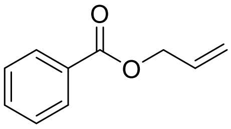

1c

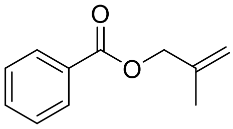

1d

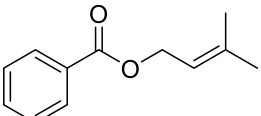

1e

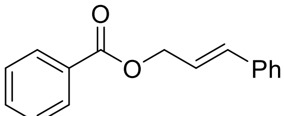

1f

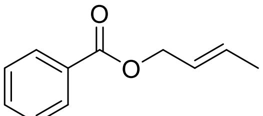

1g

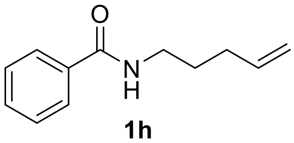

1h

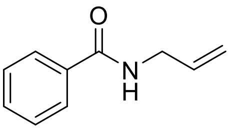

1i

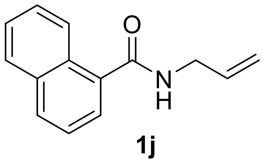

1j

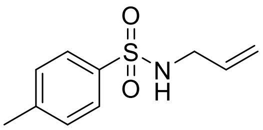

1k

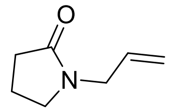

1l

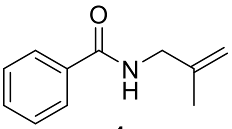

1m

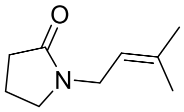

1n

Starting materials (hydrocarbons) used in Table 1

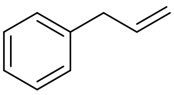

1o

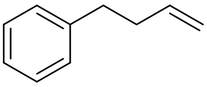

1p

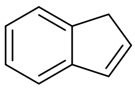

1q

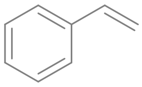

1r

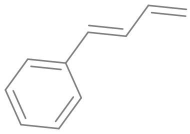

1s

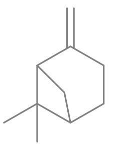

1t

Starting materials (alkynes) used in Table 1

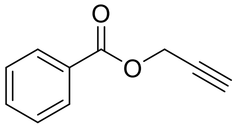

1u

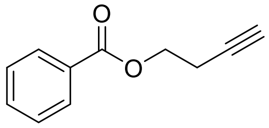

1v

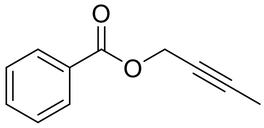

1w

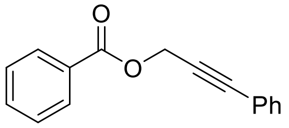

1x

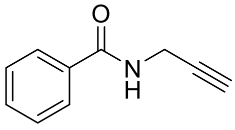

1y

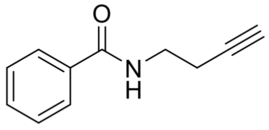

1z

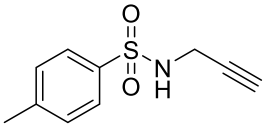

1aa

Starting materials (alkenes) used in Table 2

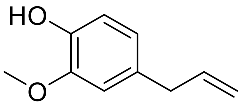

1ab

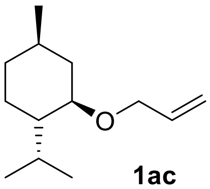

1ac

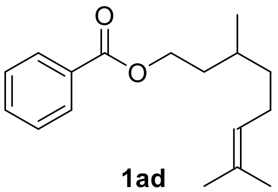

1ad

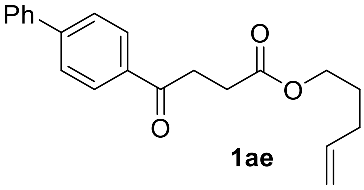

1ae

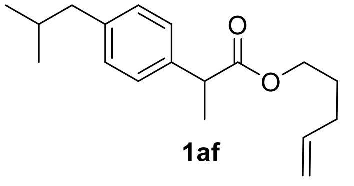

1af

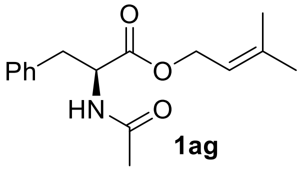

1ag

Starting material used for the clock experiment reported in Scheme 2

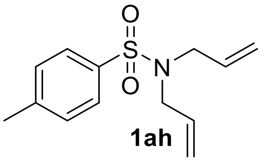

1ah

Alkyl halides used in the present work

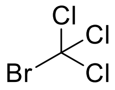

2a

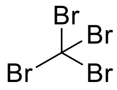

2b

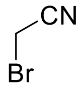

2c

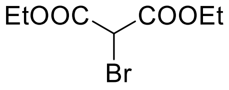

2d

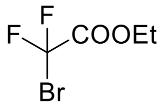

2e

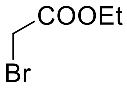

2f

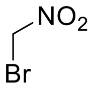

2g

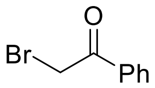

2h

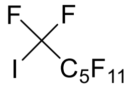

2i

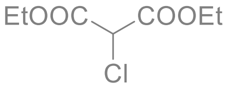

2l

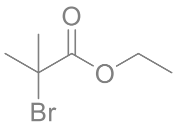

2m

1.3 Optimization of reaction conditions

The reaction conditions were optimized using the experimental setup described in the **GP-*a*** method (see **SECTION** 3.1 below), by studying the model reaction between 4-pentenyl benzoate (**1a**; 0.1 mmol, 0.1 M) and bromotrichloromethane (**2a**; 2.0 equiv.) to prepare the carbohalogenation product 4-bromo-6,6,6-trichlorohexyl benzoate (**3**). Yields reported in the **TABLEs S1-7** were determined via ^{1}H-NMR using dimethyl fumarate as internal standard (0.1 mmol, 0.1 M, 1.0 equiv.), unless otherwise specified.

Table S1. Catalyst screening in acetonitrile.

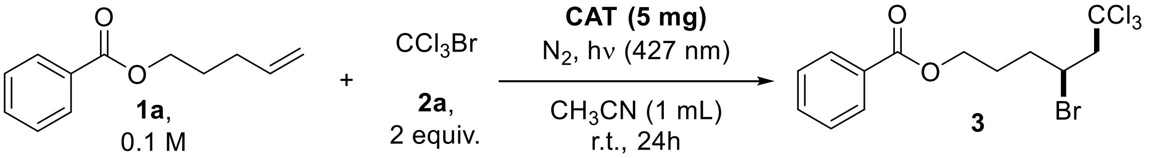

Entry	CAT	Catalyst	**3** Yield
1	**CAT1**	*g*-C_3N_4	32%
**2**	**CAT2**	***g*-C_3N_4/$Cs_2AgBiCl_6$ 90%/10%**	**86%, 76%[a]**
3	**CAT3**	*g*-C_3N_4/$Cs_2AgBiCl_6$ 70%/30%	77%
4	**CAT4**	*g*-C_3N_4/$Cs_2AgBiCl_6$ 50%/50%	80%
5	**CAT5**	*g*-C_3N_4/$Cs_2AgBiCl_6$ 10%/90%	57%
6	**CAT6**	$Cs_2AgBiCl_6$	traces

[a] isolated yield of **3** after column chromatography on silica gel (hexane/ethyl acetate as eluants); see method GP-*b*.

Table S2. Catalyst screening in aqueous acetonitrile.

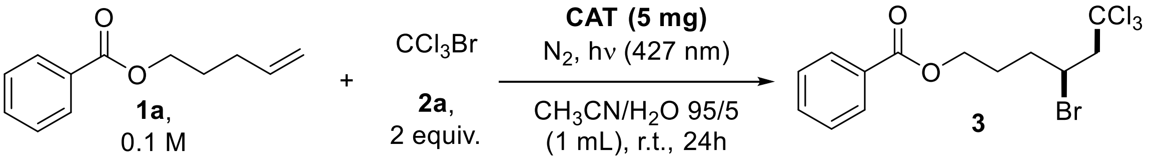

Entry	CAT	Catalyst	**3** Yield
1	**CAT1**	*g*-C_3N_4	65%
**2**	**CAT2**	***g*-C_3N_4/$Cs_2AgBiCl_6$ 90%/10%**	**90%, 78%[a]**
3	**CAT3**	*g*-C_3N_4/$Cs_2AgBiCl_6$ 70%/30%	traces
4	**CAT4**	*g*-C_3N_4/$Cs_2AgBiCl_6$ 50%/50%	n.d.
5	**CAT5**	*g*-C_3N_4/$Cs_2AgBiCl_6$ 10%/90%	n.d.
6	**CAT6**	$Cs_2AgBiCl_6$	n.d.

[a] isolated yield of **3** after column chromatography on silica gel (hexane/ethyl acetate as eluants); see method GP-*b*.

Table S3. Solvent screening.

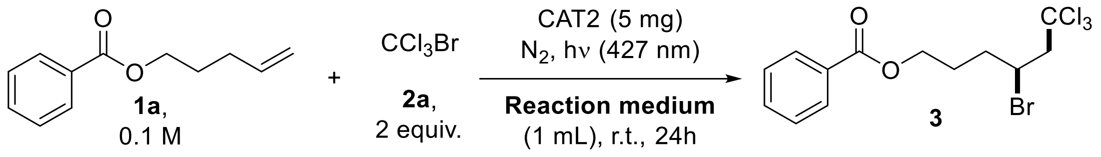

Entry	Reaction medium	**3** Yield
1	CH_3CN	86%, 76%[a]
**2**	**CH_3CN/H_2O 95/5**	**90%, 78%[a]**
3	CH_3CN/H_2O 9/1	45%
4	DCM	65%

[a] isolated yield of **3** after column chromatography on silica gel (hexane/ethyl acetate as eluants); see method GP-*b*.

Table S4. Wavelength screening.

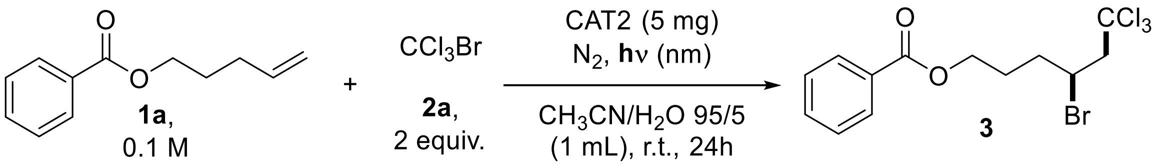

Entry	hν	**3** Yield
1	525 nm	n.p
**2**	**427 nm**	**90%, 78%[a]**
3	405 nm	88%, 66%[a]
4	456 nm	59%
5	390 nm	81%
6	370 nm	73%

[a] isolated yield of **3** after column chromatography on silica gel (hexane/ethyl acetate as eluants); see method GP-*b*.

Table S5. Alkyl halide equivalents screening.

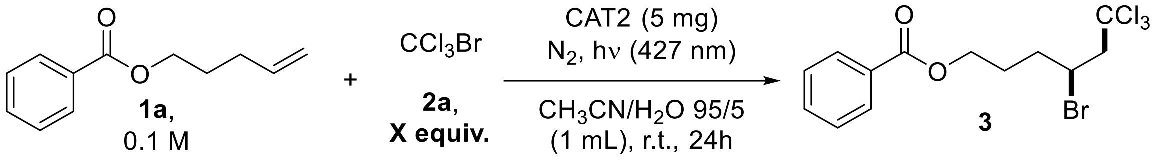

Entry	equiv. of CCl_3Br	**3** Yield
**1**	**2 equiv.**	**90%, 78%[a]**
2	1.5 equiv.	70%
3	1 equiv.	77%

[a] isolated yield of **3** after column chromatography on silica gel (hexane/ethyl acetate as eluants); see method GP-*b.*

Table S6. Catalyst loading screening.

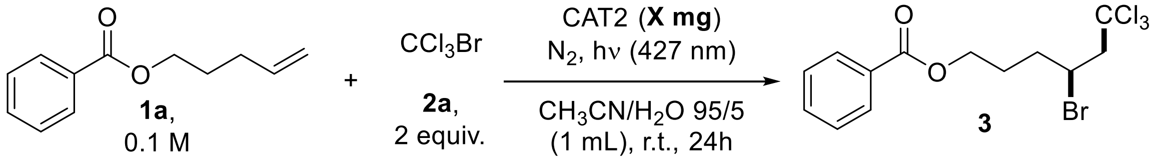

Entry	Catalyst loading	**3** Yield
**1**	**5 mg**	**90%, 78%[a]**
2	4 mg	68%
3	3 mg	65%

[a] isolated yield of **3** after column chromatography on silica gel (hexane/ethyl acetate as eluants); see method GP-*b.*

Table S7. Control experiments.

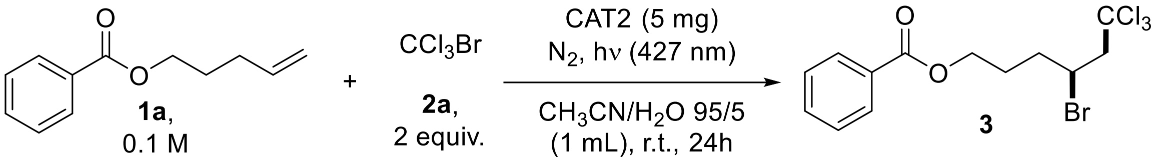

Entry	Control experiment	**3** Yield
1	no light	n.d.
2	no catalyst	n.d.
3	O_2	n.d.
4	no alkyl halide	n.d.
5	no catalyst, heating (50°C)	n.d.
6[a]	CAT1/CAT6 90:10%$_{w/w}$ ratio	67%

[a] A *g*-$C_3N_4/Cs_2AgBiCl_6$ physical mixture has been used in the role of heterogeneous photocatalyst.

1.4 *post*-Catalysis material characterization

Figure S5 gathers the structural characterization (via powder X-ray diffraction - PXRD) of the best performing heterojunction (**CAT2**, namely *g*-$C_3N_4/Cs_2AgBiCl_6$ 90:10) collected *pre*- and *post*-catalysis, where the main peaks of the perovskite phase are clearly detectable, both before and after the photocatalytic reaction. The slight broadening of the peaks observed in the latter case is probably correlated with the partial amorphization of the perovskite phase that occurs during the photocatalytic process.

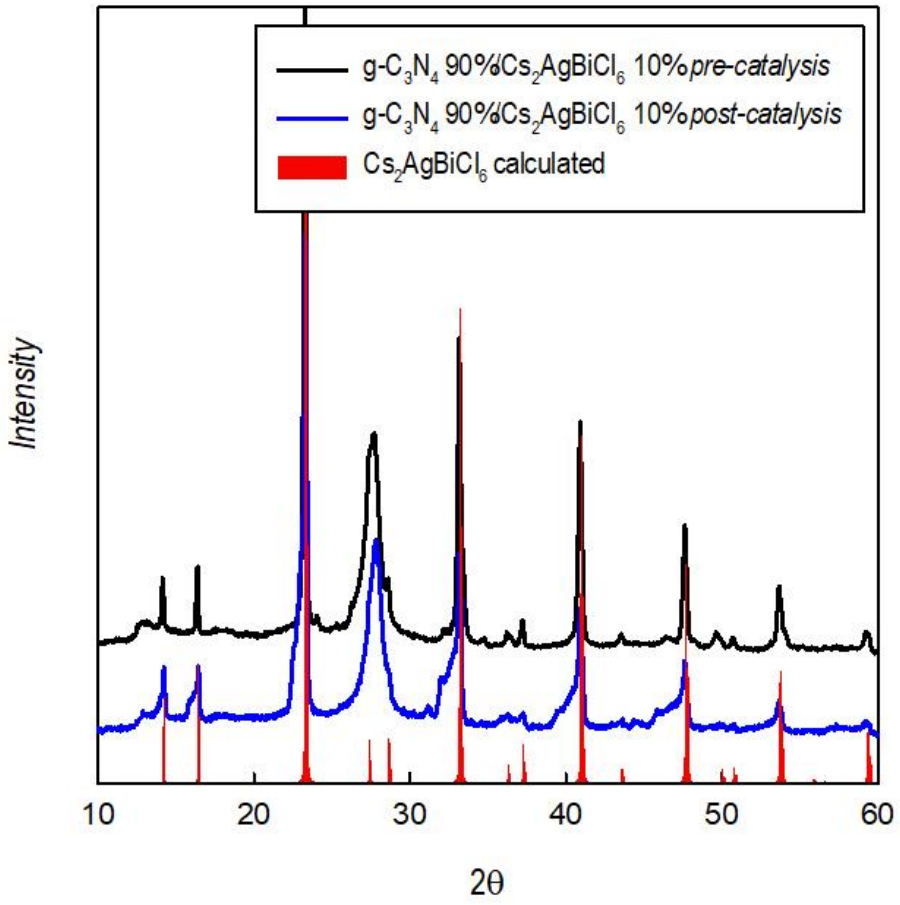

Figure S5. Structural characterization of the best performing heterojunction (**CAT2**, namely *g*-$C_3N_4/Cs_2AgBiCl_6$ 90:10) collected *pre*- and *post*-catalysis.

Likewise, scanning electron microscopy (SEM) showed that there was no obvious difference in the morphology of *pre*- and *post*-catalysis **CAT2**. Microstructural characterization of the samples was

made using a high-resolution scanning electron microscope (SEM, TESCAN Mira 3) operated at 25 kV.

The results of Energy Dispersive X-ray Spectroscopy (EDS) *post*-catalysis analysis are reported in **Figure S6**. Thus, the post-catalysis EDS elemental mapping results confirm the presence of the perovskite phase, proving a remarkable stability of the composite system under the implemented reaction conditions.

Element	App Conc.	Intensity Corrn.	Weight%	Weight% Sigma	Atomic%
C K	5.72	0.3487	17.66	0.44	52.21
N K	0.56	0.1307	4.63	0.63	11.74
O K	0.76	0.4998	1.63	0.17	3.62
Cl K	13.07	0.8268	17.02	0.18	17.05
Br L	3.43	0.6640	5.57	0.11	2.47
Ag L	6.04	0.7303	8.90	0.15	2.93
Cs L	17.50	0.7659	24.59	0.26	6.57
Bi M	15.70	0.8446	20.00	0.29	3.40
Totals			100.00		

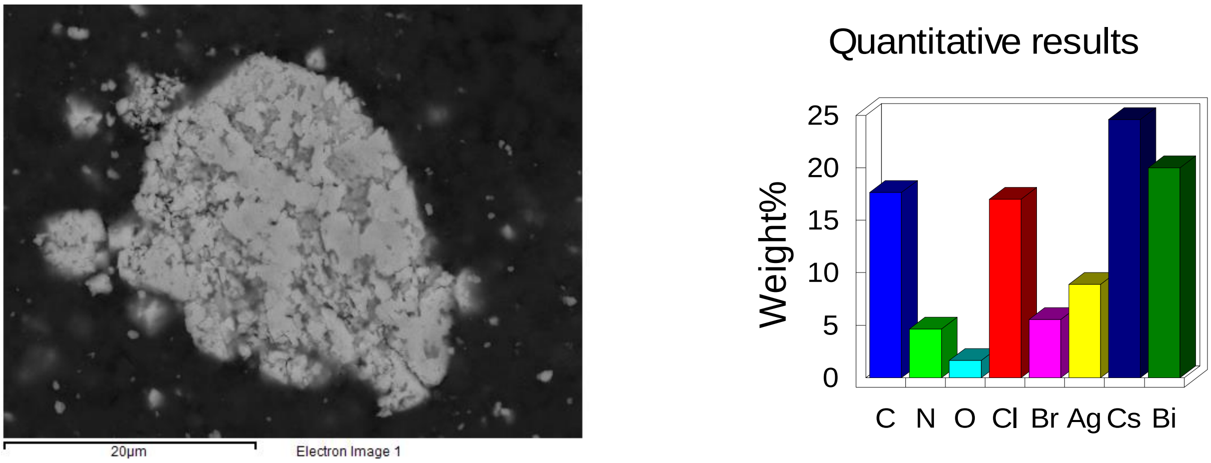

Figure S6. Energy Dispersive X-ray Spectroscopy (EDS) *post*-catalysis analysis.

1.5 Catalyst recovery

To demonstrate the recyclability of the *g*-C_3N_4/$Cs_2AgBiCl_6$ (90%/10%) catalyst (**CAT2**), we run the model reaction between 4-pentenyl benzoate (**1a**; 0.5 mmol, 0.1 M) and bromotrichloromethane (**2a**; 1 mmol, 2.0 equiv.) to produce 4-bromo-6,6,6-trichlorohexyl benzoate (**3**) according to the conditions described in general procedure **GP-*b***. The reported yield values do refer to the isolated yield of **3** obtained after each run.

After each reaction run, the solids were centrifuged for 10 minutes at 10.000 rpm, and the supernatant was subjected to the purification described in general procedure **GP-*b*** to isolate **3**. For each cycle, the catalyst was washed with acetonitrile, centrifuged, and then dried under vacuum overnight. The recovered sample of the *g*-C_3N_4/$Cs_2AgBiCl_6$ (90%/10%) catalyst (**CAT2**) was then recovered for 5 runs, demonstrating unchanged performance over 4 runs and a slight decrease at the 5th cycle (see **TABLE S8**).

TABLE S8. Results from multiple recycling of the *g*-C_3N_4/$Cs_2AgBiCl_6$ (90%/10%) catalyst.

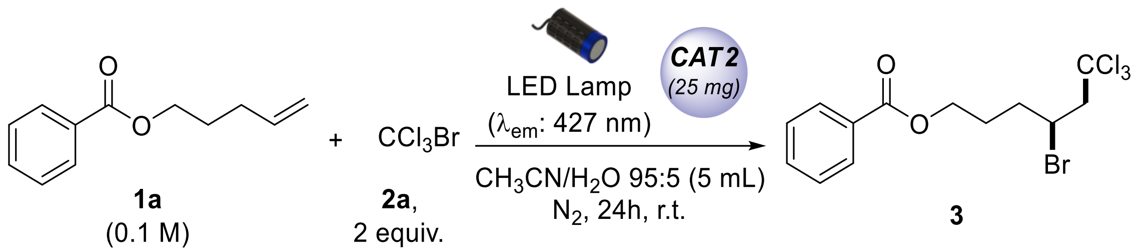

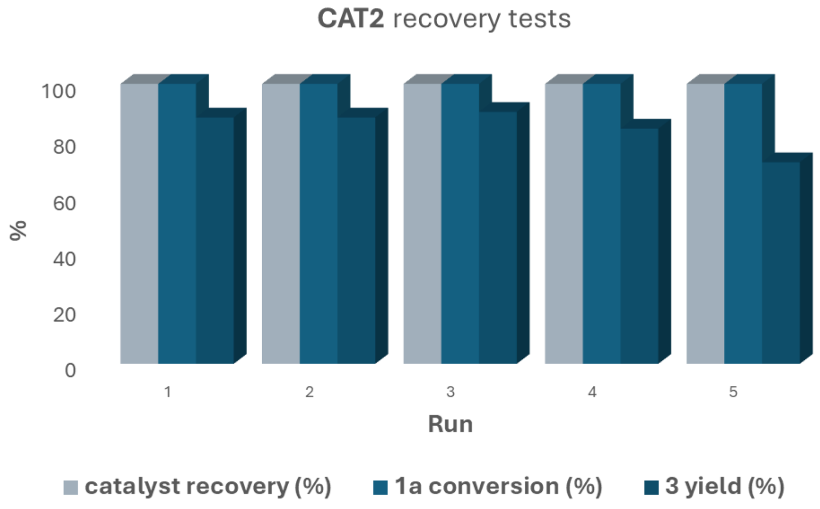

Run	3 Yield (%)
1	88
2	88
3	90
4	84
5	72

1.6 Mechanistic investigations

1.6.1 Radical scavenger experiment

To have insights into the reaction mechanism, the model reaction the model reaction between 4-pentenyl benzoate (**1a**; 0.1 mmol, 0.1 M) and bromotrichloromethane (**2a**; 1 mmol, 2.0 equiv.) to produce 4-bromo-6,6,6-trichlorohexyl benzoate (**3**) was run according to the conditions described in general procedure **GP-*a***, in the presence of different amounts of the additive TEMPO (2,2,6,6-tetramethylpyrrolidine *N*-oxyl) as free radical scavenger. Different experiments were performed, as indicated in **Scheme S1** (see also **Scheme 2a** in the main text, upper part), while the corresponding reaction crudes have been subjected to GC-MS analysis (**Figure S7**). The resulting GC traces were compared with those of selected reference compounds (**Figure S8**).

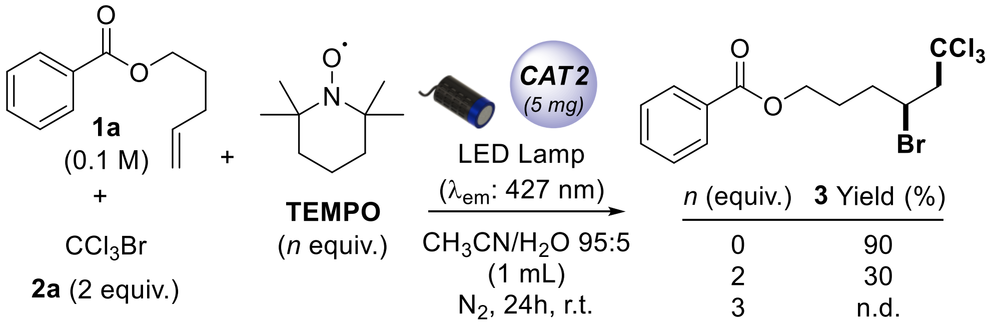

Scheme S1. Experiments performed to evaluate the effect of the TEMPO additive on the model reaction between 4-pentenyl benzoate (**1a**) and bromotrichloromethane (**2a**).

Upon inspection of the GC charts in **Figure S7**, it is apparent the effect of the TEMPO additive on the reaction course, clearly demonstrating the radical nature of the reported process. Thus, the model reaction in **Scheme S1** (0 equiv. TEMPO added) cleanly leads to product **3** (peak at 23.99 min. in **Figure S7, *i***; compare also **Figure S8, *i***), while its formation is largely suppressed by the presence of TEMPO (**Figure S7, *ii*** and ***iii***). On the other hand, the latter two traces show peaks of residual starting materials, namely **1a** (peak at *ca*. 14.5 min.; compare also **Figure S8, *ii***) and TEMPO (peak at *ca*. 9.0 min.; compare also **Figure S8, *iii***).

i) 0 equiv. TEMPO

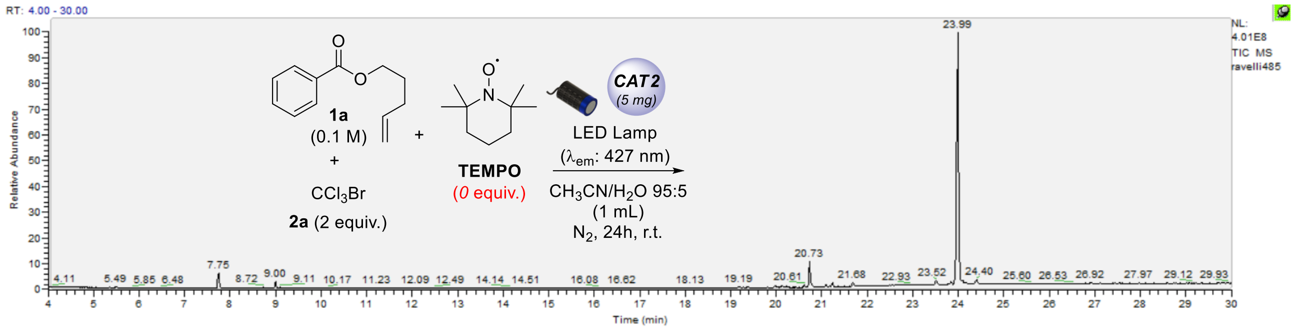

ii) 2 equiv. TEMPO

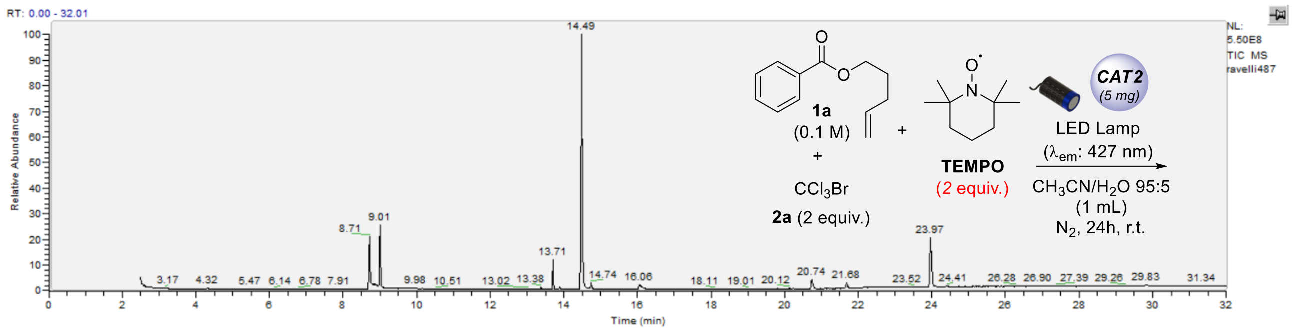

iii) 3 equiv. TEMPO

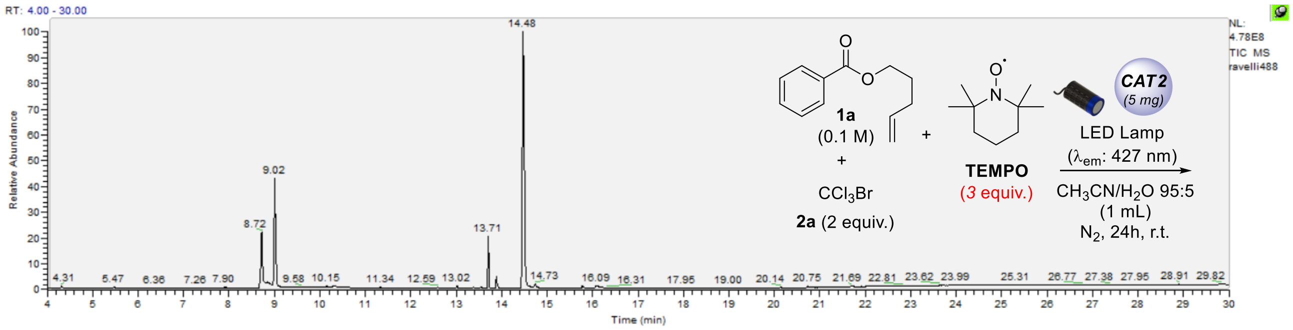

Figure S7. GC-MS traces of the crude reaction mixtures between **1a** and **2a** corresponding to the experiments gathered in **Scheme S1**, in the presence of different amounts of the TEMPO additive: *i*) 0 equiv, *ii*) 2 equiv. and *iii*) 3 equiv..

i) *Reference compound: 3*

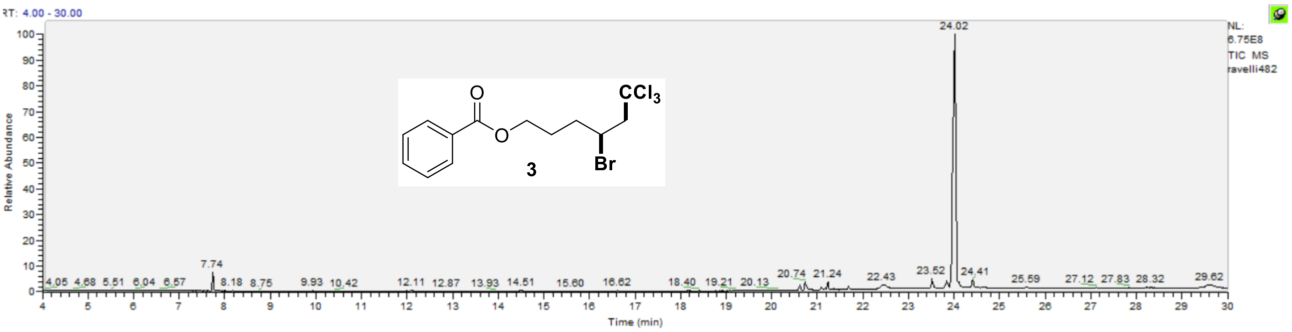

ii) *Reference compound: 1a*

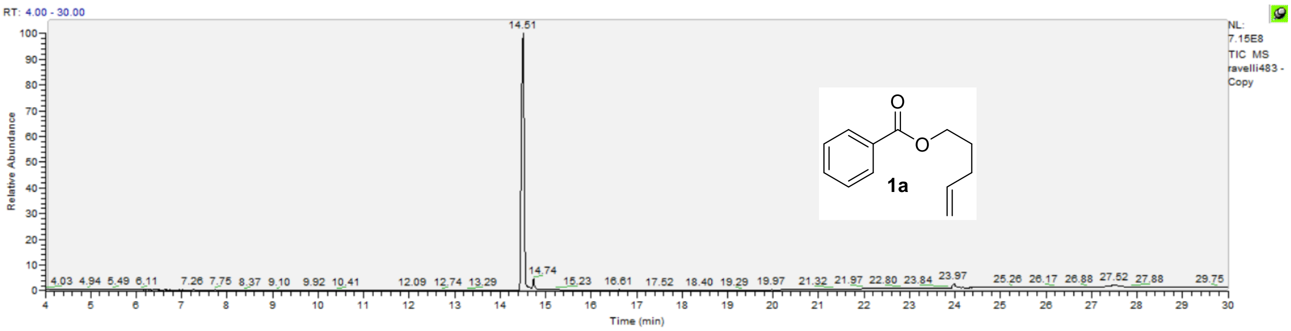

iii) *Reference compound: TEMPO*

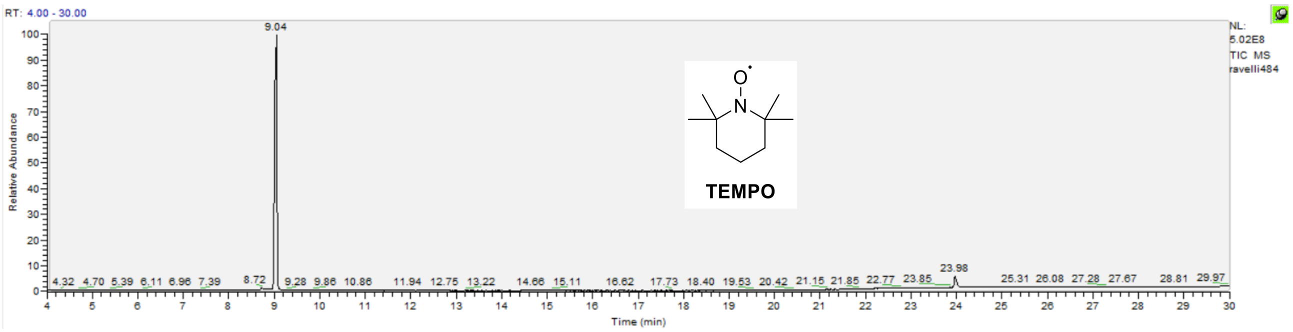

Figure S8. GC-MS traces selected reference compounds: ***i)*** product **3**; ***ii)*** 4-pentenyl benzoate (**1a**); ***iii)*** TEMPO additive.

1.6.2 Radical clock experiment

To support the involvement of a radical pathway, substrate **1ah** was subjected to optimized reaction conditions (see general procedure **GP-*b***). When reacted with CCl_3Br, the clean formation of product **63** (80% yield) through a 5-*exo*-trig radical cyclization process was observed (see Scheme S2).[S6]

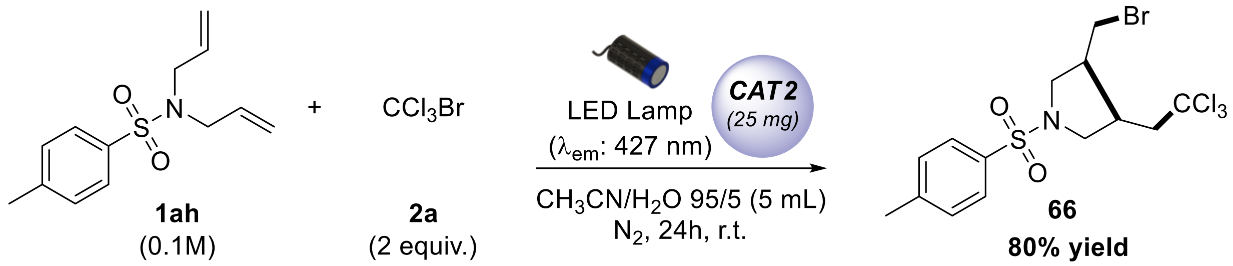

Scheme S2. Radical clock experiment between *N*-tosylated diene **1ah** and bromotrichloromethane **2a**.

1.6.3 Mechanistic proposal

Based on experimental evidence, **Scheme S3** reports a mechanistic proposal for the difunctionalization of C–C multiple bonds in the presence of the $90:10_{w/w}$ *g*-$C_3N_4/Cs_2AgBiCl_6$ heterojunction. In accordance with the redox potential of the catalyst, the employed alkyl halides (**2**; general formula R–X) undergo single electron-reduction by the photoexcited electrons in the conduction band, forming a radical anion species ($\mathbf{2}^{\bullet-}$) that subsequently generates a reactive *C*-centered radical ($\mathbf{I}^{\bullet}$) upon halide anion loss (X^-). The thus formed organoradical $\mathbf{I}^{\bullet}$ then adds onto the unsaturated π-trap to form radical adduct $\mathbf{II}^{\bullet}$, which then kicks off a radical chain process involving a new equivalent of the starting alkyl halide. According to this mechanistic scenario, the radical addition step governs the regiochemistry of the transformation, with the selective formation of the most stabilized radical intermediate (see path *a*, applied to the case of alkenes difunctionalization). As a matter of fact, the only example wherein a regioisomeric mixture has been obtained, is represented by the process leading to **26**/**26'**, wherein the competition between two analogous secondary-type radical adducts occurs (see Table 1 in the main text).

An additional element relates to the stereoselectivity associated with alkynes difunctionalization, wherein a vinyl-type radical intermediate is involved. As reported in the literature, either linear π-type (see path *b*) or *Z*/*E*-configured σ-type (see path *c*) intermediates exist.[S7] In our work, most examples involve a σ-type intermediate, wherein *E*/*Z*-isomerization is facile. Indeed, the stereochemistry of the final products is dictated by a complex interplay of effects, including relative population of the involved intermediates, rate of radical trapping and steric of the substituents.[S7] Experimentally, it has been observed that the final products are formed with a strong preference toward a *syn* addition of the R/X groups across the triple bond. By contrast, a poor selectivity (*E*/*Z* 2:1) has been observed for product **52**, formed through a linear π-type intermediate.

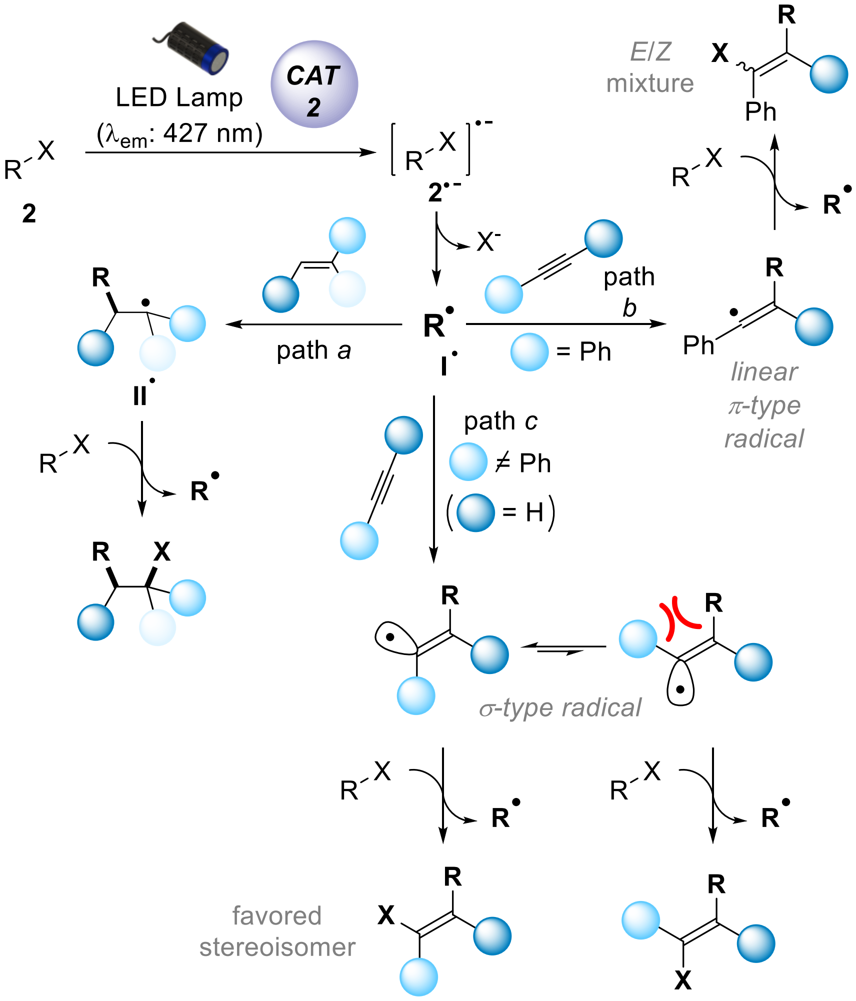

Scheme S3. Proposed mechanistic scenario for the photocatalyzed ATRA-type difunctionalization of alkenes (path *a*) and alkynes (paths *b*, *c*).

2. Experimental details

2.1 General information

All reagents, chemicals and solvents used in this work were sourced from various commercial suppliers (TCI Europe, Merck - Sigma Aldrich, Fluorochem, Carlo Erba). Solvents and reagents **2** were used as supplied without any further purification. Compounds **1** were synthesized according to the procedures reported below (see Section 2.3, Experimental Methods), with the only exception of compounds **1o-t** and **1ab**, that are commercially available and have been used as received. The *g*-$C_3N_4/Cs_2AgBiCl_6$ materials used as photocatalyst were prepared according to a procedure previously reported by some of us.[S1]

The light source adopted for photochemical experiments was a 40 W Kessil LED lamp (with emission centered at the specified wavelength; no filters used), operated at full power (technical specifications at https://www.kessil.com/science/PR160L) and positioned at *ca*. 10 cm from the reaction vessel.

Analytical thin layer chromatography (TLC) plates (silica gel 60 F254) were visualized either with a UV lamp (254 nm), or by submersion in the chosen stain for TLC. Column chromatography was carried out using 40-63 μm particle sized silica gel.

NMR spectra were recorded on a 400 (for ^{1}H) or 101 (for ^{13}C) MHz spectrometer; the attributions were made based on ^{1}H and ^{13}C NMR. Data for ^{1}H NMR are reported as follows: chemical shifts referred to TMS (δ ppm), multiplicity (s = singlet, bs = broad singlet, d = doublet, dd= double doublet, t = triplet, dt = double triplet, q = quadruplet, quint = quintuplet, m = multiplet), coupling constant (Hz) and integration. NMR yields were calculated using dimethyl fumarate as internal standard. Data for ^{13}C NMR are reported in terms of chemical shift. When specified, structural assignments were made with additional information from gCOSY, gHSQC, gHMBC and gNOESY experiments.

GC/MS analyses were carried out on a Thermo Scientific DSQII single quadrupole GC/MS system. The injection in the GC/MS system was performed at 250°C in split mode. A Restek Rxi-5ms 30 m × 0.25 mm × 0.25 μm film thickness capillary column was used with helium as the carrier gas at a constant flow rate of 1.0 mL/min. The transfer line temperature was held at 270°C and the ion source temperature was 250°C. Electron ionization mode was used with 70 eV and the ions were registered in full scan mode in a mass range of m/z 40-600 amu. The chromatogram acquisition, detection of mass spectral peaks and their waveform processing were performed using Xcalibur MS Software Version 2.2 (Thermo Scientific Inc.).

For catalyst recovery purposes, an OHAUS Frontier™ Multi Pro FC5816 centrifuge with 6×50 mL rotor has been adopted.

HRMS data were acquired using a X500B QTOF System (SCIEX, Framingham, MA 01701 USA) available at the CGS facility of the University of Pavia (https://cgs.unipv.it/eng/), equipped with the Twin Sprayer ESI probe and coupled to an ExionLC™ system (SCIEX). The SCIEX OS software 2.1.6 was used as operating platform. For MS detection the following parameters were applied: curtain gas: 30 psi, ion source gas 1: 45 psi, ion source gas 2: 55 psi, temperature: 450°C, polarity positive, ion spray voltage: -4500 V, TOF mass range: 50-1600 Da, declustering potential: -60 V and collision energy: -10 V.

2.2 General procedure for the synthesis of the catalyst (GPC)

The procedure adopted in this work for the synthesis of *g*-C_3N_4/$Cs_2AgBiCl_6$ heterojunctions has been previously reported in the literature.[S1]

Briefly, *g*-C_3N_4 nanosheets have been synthesized from the bulk form (bulk *g*-C_3N_4).

Bulk *g*-C_3N_4 was prepared through a polymerization of dicyandiamine (DCD; $NH_2C(=NH)NHCN$, Aldrich, 99%) by the following thermal treatment under N_2 flux: heating (1 °C min^{-1}) to a selected temperature of 550 °C, 4h isothermal step, cooling to room temperature (10 °C min^{-1}). The synthesis was carried out in a partially closed alumina crucible.

The thermally exfoliated catalyst (*g*-C_3N_4 nanosheets) was prepared by heating bulk *g*-C_3N_4 in air to a selected temperature of 500 °C followed by an isothermal step for 2 h.

The *g*-C_3N_4 nanosheet / $Cs_2AgBiCl_6$ heterojunctions have been synthesized dissolving a stoichiometric ratio of the precursor salts AgCl (Aldrich 99%), $BiCl_3$ (Aldrich 99%) and CsCl (Aldrich 99%) in *N*,*N*-dimethylformamide (DMF) and dried under vigorous stirring at 65 °C. This polar solvent can dissolve most organic and inorganic salts and may be suitable for the cations that are insoluble in HCl.

The composites series has been prepared by changing the percentage of the $Cs_2AgBiCl_6$ perovskite and the *g*-C_3N_4 nanosheets in the composites, as reported above (see section 1.1) and in the main text (see **Figure 1a**).

The determination of band gap energy through the Tauc plot method was done by plotting the value of "$(\alpha h\nu)^{1/2}(eV/cm)^2$" against "Energy (hν)", then by extrapolating the linear range of each curve. The intersection of the resulting lines with the x-axis offers an estimate of the corresponding energy gap (see **Figure S9**).

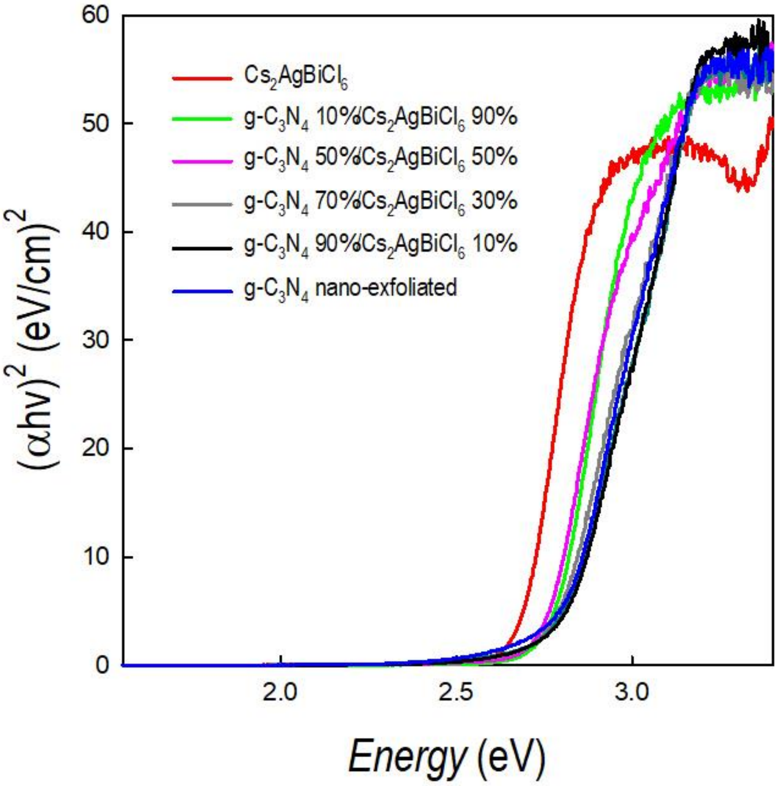

Figure S9. Tauc plots for nano-exfoliated *g*-C_3N_4 (**CAT1**), the complete series of the prepared *g*-C_3N_4/$Cs_2AgBiCl_6$ heterojunctions (**CAT2-5**), and pure $Cs_2AgBiCl_6$ (**CAT6**). Adapted from Ref. S1 (Creative Commons CC BY license).

2.3 Synthesis of starting materials (GP-I – GP-VI)

Synthesis of compounds 1a-g, 1u-x and 1ad (GP-I)

Esters **1a-g**, **1u-x** and **1ad** were synthesized by following a known procedure adapted from the literature (**GP-I**; see **Scheme S4**).[S8] In a round bottom flask, 10 mmol (0.4 M, 1 equiv.) of the chosen alcohol, 112 mg (1 mmol, 10 mol%) of 4-dimethylaminopyridine (DMAP) and 1.44 mL (10 mmol, 1 equiv.) of triethylamine have been dissolved in 25 mL of DCM under Ar atmosphere. The mixture was cooled in an ice bath, then 12 mmol (1.2 equiv.) of acyl chloride have been added dropwise. The resulting mixture was left under stirring at room temperature for one night. The mixture was then washed with water and $NaHCO_3$ solution (20 mL) and extracted with DCM. The organic phase was dried with $MgSO_4$ and filtered. After solvent removal, the crude mixture was purified by flash column chromatography.

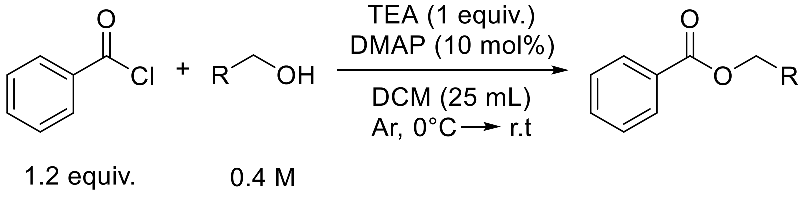

Scheme S4. General procedure **I** (**GP-I**) for the synthesis of substrates.

Synthesis of compounds 1h-k, 1m and 1x-aa (GP-II)

Amides **1h-k**, **1m**, **1x-aa** were synthesized by following a known procedure adapted from the literature (**GP-II**; see **Scheme S5**).[S9] In a round bottom flask, 10.4 mmol (1.1 equiv.) of the chosen acyl chloride have been dissolved in 10 mL of dry DCM under Ar atmosphere. The solution was cooled in an ice bath, then 10.4 mmol (1.1 equiv.) of triethylamine and 9.4 mmol (0.9 M, 1 equiv.) of the chosen amine have been added dropwise. The resulting mixture was left under stirring at room temperature for one night. The mixture was then washed with water (20 mL) and extracted with DCM. The organic phase was dried with $MgSO_4$ and filtered. After solvent removal, the crude mixture was purified by flash column chromatography.

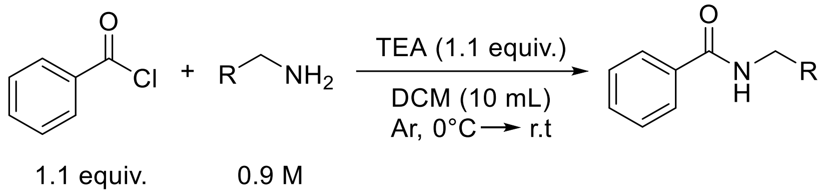

Scheme S5. General procedure **II** (**GP-II**) for the synthesis of substrates.

Synthesis of compounds 1l, 1n and 1ah (GP-III)

Amides **1l**, **1n** and **1ah** were synthesized by following a known procedure adapted from the literature (**GP-III**; see **Scheme S6**).[S10] In a round bottom flask, 16 mmol (1.6 equiv.) of KOH were suspended in 15 mL of THF under Ar atmosphere and 15 mmol (1.5 equiv.) of the chosen amide are added. The solution was cooled in an ice bath, then 10 mmol (0.7 M, 1 equiv.) of the chosen propargyl bromide were added dropwise. The resulting mixture was left under stirring at room temperature for one night. The mixture was then washed with water (20 mL) and extracted with DCM. The organic phase was then dried with $MgSO_4$. The organic phase was dried with $MgSO_4$ and filtered. After solvent removal, the crude mixture was purified by flash column chromatography.

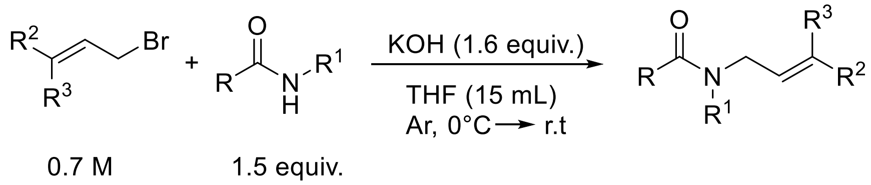

Scheme S6. General procedure **III** (**GP-III**) for the synthesis of substrates.

Synthesis of compound 1ac (GP-IV)

Compound **1ac** was synthesized by following a known procedure adapted from the literature (**GP-IV**; see **Scheme S7**).[S11] The aliphatic alcohol (0.75 M, 1 equiv.) was added to a flame-dried round bottom flask equipped with a stir bar and dissolved in dry 10 mL of DMF. The reaction mixture was cooled to 0 °C in an ice-bath, followed by portion-wise addition of sodium hydride (3 equiv., 60% oil dispersion); the resulting mixture was stirred for additional 10 minutes. Then, allyl bromide (1.5 equiv.) was added dropwise to the reaction mixture. The resulting mixture was left under stirring at room temperature for one night. The reaction was finally quenched by the slow addition of aq. NH_4Cl, diluted with water and extracted with ethyl acetate (3×). The organic phase was dried with $MgSO_4$ and filtered. After solvent removal, the crude mixture was purified by flash column chromatography.

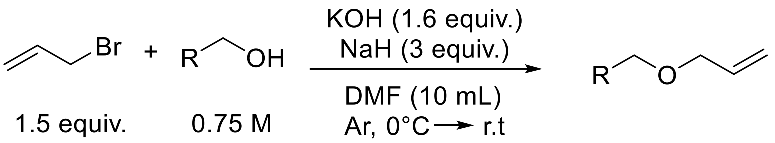

Scheme S7. General procedure **IV** (**GP-IV**) for the synthesis of substrates.

Synthesis of compounds 1ad and 1af (GP-V)

Compounds **1ad** and **1af** were synthesized by following a known procedure adapted from the literature (**GP-V**; see **Scheme S8**).[S12] In a round bottom flask, 5 mmol (0.5 M) of the chosen carboxylic acid were added to a solution of 1-(3- dimethylaminopropyl)-3-ethylcarbodiimide (EDC, 1.3 equiv.) and DMAP (0.1 equiv.) in DCM (10 mL) at 0 °C. Alcohol (1.2 equiv.) was then added. The resulting mixture was left under stirring at room temperature for one night. The solution was then diluted with DCM (40 mL) and washed with 1M HCl (3×10 mL), saturated $NaHCO_3$ (20 mL) and brine (20 mL) sequentially. The organic layer was dried over anhydrous Na_2SO_4. After removal of solvent under reduced pressure, the crude product was purified by column chromatography.

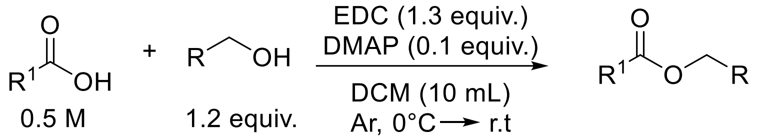

Scheme S8. General procedure **V** (**GP-V**) for the synthesis of substrates.

Synthesis of compound 1ag (GP-VI)

Compound **1ag** was synthesized by following a known procedure adapted from the literature (**GP-VI**; see **Scheme S9**).[S13] In a round bottom flask, 5.0 mmol (0.3 M) of the *N*-protected amino acid were added to DMF (15 mL) and the solution was cooled to 0 °C. Hunig's base (1.3 equiv.) was then added, followed by the chosen allyl bromide (1.3 equiv.). The resulting mixture was left under stirring at room temperature for one night. The solution was then diluted with EtOAc (40 mL), and washed with water (2×30 mL) and brine (30 mL). The organic phase was dried with $MgSO_4$ and filtered. After solvent removal, the crude mixture was purified by flash column chromatography.

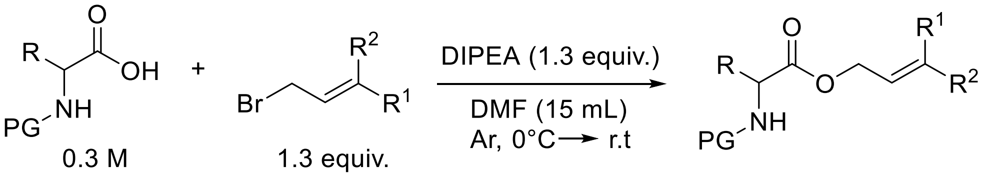

Scheme S9. General procedure **VI** (**GP-VI**) for the synthesis of substrates.

2.4 Characterization of starting materials 1a-1n, 1u-1aa, 1ac-1ah

4-Pentenyl-benzoate (1a)

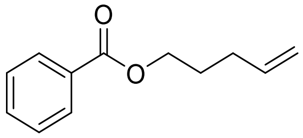

Following **GP-I**, starting from 1.03 mL (10 mmol, 1 equiv.) of pent-4-en-1-ol, 122 mg (1 mmol, 10% mol) of 4-dimethylaminopyridine (DMAP), 1.44 mL (10 mmol, 1 equiv.) of triethylamine and 1.40 mL (12 mmol, 1.2 equiv.) of benzoyl chloride in 20 mL of dry DCM. The crude mixture was purified using silica gel chromatography (95:5, cyclohexane/ethyl acetate) to afford 1.90 g of **1a** (10 mmol, >99% yield) as a colorless oil. Spectroscopic data for **1a** are in accordance with literature.[S8]

^{1}H NMR (400 MHz, CDCl$_3$): δ 8.09 – 8.01 (m, 2H), 7.60 – 7.51 (m, 1H), 7.49 – 7.40 (m, 2H), 5.91 – 5.81 (m, 1H), 5.13 – 4.98 (m, 2H), 4.34 (t, *J* = 6.6 Hz, 2H), 2.28 – 2.18 (m, 2H), 1.88 (dq, *J* = 8.5, 6.6 Hz, 2H).

^{13}C{^{1}H} NMR (101 MHz, CDCl$_3$): δ 166.5, 137.3, 132.7, 130.3, 129.4, 128.2, 115.2, 64.2, 30.0, 27.8.

4-Pentenyl 4-methylbenzenesulfonate (1b)

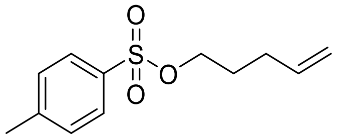

Following **GP-I**, starting from 1.03 mL (10 mmol, 1 equiv.) of pent-4-en-1-ol, 122 mg (1 mmol, 10% mol) of 4-dimethylaminopyridine (DMAP), 1.44 mL (10 mmol, 1 equiv.) of triethylamine and 2.30 g (12 mmol, 1.2 equiv.) of 4-methylbenzenesulfonyl chloride in 20 mL of dry DCM. The crude mixture was purified using silica gel chromatography (95:5, cyclohexane/ethyl acetate) to afford 1.80 g of **1b** (7.5 mmol, 75% yield) as a colorless oil. Spectroscopic data for **1b** are in accordance with literature.[S14]

^{1}H NMR (400 MHz, CDCl$_3$): δ 7.78 (d, *J* = 8.4 Hz, 2H), 7.34 (d, *J* = 8.1 Hz, 2H), 5.76 – 5.64 (m, 1H), 4.96 (dq, *J* = 4.3, 1.7 Hz, 1H), 4.93 (t, *J* = 1.4 Hz, 1H), 4.03 (t, *J* = 6.4 Hz, 2H), 2.44 (s, 3H), 2.13 – 2.02 (m, 2H), 1.73 (dq, *J* = 8.2, 6.5 Hz, 2H).

^{13}C{^{1}H} NMR (101 MHz, CDCl$_3$): δ 145.1, 137.0, 133.6, 130.3, 128.3, 116.3, 70.2, 29.8, 28.4, 22.0.

Allyl benzoate (1c)

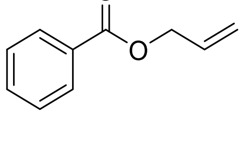

Following **GP-I**, starting from 0.70 mL (10 mmol, 1 equiv.) of prop-2-en-1-ol, 122 mg (1 mmol, 10% mol) of 4-dimethylaminopyridine (DMAP), 1.44 mL (10 mmol, 1 equiv.) of triethylamine and 1.40 mL (12 mmol, 1.2 equiv.) of benzoyl

chloride in 20 mL of dry DCM. The crude mixture was purified using silica gel chromatography (95:5 cyclohexane/ethyl acetate) to afford 1.17 g of **1c** (7.2 mmol, 72% yield) as a colorless oil. Spectroscopic data for **1c** are in accordance with literature.[S15]

^{1}H NMR (400 MHz, CDCl$_3$): δ 8.11 – 8.04 (m, 2H), 7.61 – 7.52 (m, 1H), 7.45 (dd, *J* = 8.4, 7.0 Hz, 2H), 6.12 – 6.00 (m, 1H), 5.42 (dq, *J* = 17.2, 1.6 Hz, 1H), 5.29 (dq, *J* = 10.4, 1.3 Hz, 1H), 4.83 (dt, *J* = 5.6, 1.4 Hz, 2H).

^{13}C{^{1}H} NMR (101 MHz, CDCl$_3$): δ 166.1, 132.8, 132.1, 130.0, 129.5, 128.2, 118.0, 65.4.

2-Methylallyl benzoate (1d)

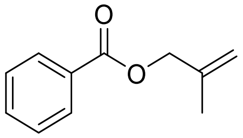

Following **GP-I**, starting from 0.85 mL (10 mmol, 1 equiv.) of 2-methylprop-2-en-1-ol, 122 mg (1 mmol, 10% mol) of 4-dimethylaminopyridine (DMAP), 1.44 mL (10 mmol, 1 equiv.) of triethylamine and 1.40 mL (12 mmol, 1.2 equiv.) of benzoyl chloride in 20 mL of dry DCM. The crude mixture was purified using silica gel chromatography (95:5, cyclohexane/ethyl acetate) to afford 1.60 g of **1d** (9.1 mmol, 91% yield) as a colorless oil. Spectroscopic data for **1d** are in accordance with literature.[S8]

^{1}H NMR (400 MHz, CDCl$_3$): δ 8.12 – 8.04 (m, 2H), 7.61 – 7.52 (m, 1H), 7.50 – 7.40 (m, 2H), 5.08 (t, *J* = 1.3 Hz, 1H), 4.99 (t, *J* = 1.4 Hz, 1H), 4.75 (s, 2H), 1.85 (t, *J* = 1.2 Hz, 3H).

^{13}C{^{1}H} NMR (101 MHz, CDCl$_3$): δ 166.1, 139.9, 132.8, 130.1, 129.5, 128.2, 112.8, 68.0, 19.4.

3-Methylbut-2-en-1-yl benzoate (1e)

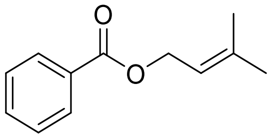

Following **GP-I**, starting from 1.01 mL (10 mmol, 1 equiv.) of 3-methylbut-2-en-1-ol, 122 mg (1 mmol, 10% mol) of 4-dimethylaminopyridine (DMAP), 1.44 mL (10 mmol, 1 equiv.) of triethylamine and 1.40 mL (12 mmol, 1.2 equiv.) of benzoyl chloride in 20 mL of dry DCM. The crude mixture was purified using silica gel chromatography (95:5, cyclohexane/ethyl acetate) to afford 1.90 g of **1e** (10 mmol, >99% yield) as a colorless oil. Spectroscopic data for **1e** are in accordance with literature.[S16]

^{1}H NMR (400 MHz, CDCl$_3$): δ 8.09 – 8.01 (m, 2H), 7.59 – 7.41 (m, 1H), 7.48 – 7.38 (m, 2H), 5.50 – 5.45 (m, 1H), 4.82 (d, *J* = 7.2 Hz, 2H), 1.78 (dd, *J* = 6.6, 1.4 Hz, 6H).

^{13}C{^{1}H} NMR (101 MHz, CDCl$_3$): δ 166.5, 138.96, 132.6, 130.4, 129.4, 128.1, 118.5, 61.7, 25.6, 18.0.

Cinnamyl benzoate (1f)

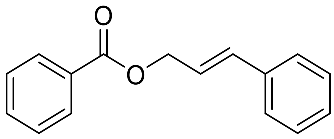

Following **GP-I**, starting from 1.30 mL (10 mmol, 1 equiv.) of cinnamyl alcohol, 122 mg (1 mmol, 10% mol) of 4-dimethylaminopyridine (DMAP), 1.44 mL (10 mmol, 1 equiv.) of triethylamine and 1.40 mL (12 mmol, 1.2 equiv.) of benzoyl chloride in 20 mL of dry DCM. The crude mixture was purified using silica gel chromatography (95:5, cyclohexane/ethyl acetate) to afford 1.67 g of **1f** (7.0 mmol, 70% yield) as a colorless oil. Spectroscopic data for **1f** are in accordance with literature.[S17]

^{1}H NMR (400 MHz, CDCl$_3$): δ 8.14 – 8.05 (m, 2H), 7.63 – 7.51 (m, 1H), 7.50 – 7.39 (m, 4H), 7.38 – 7.29 (m, 2H), 7.29 – 7.23 (m, 2H), 6.75 (dt, J = 16.0, 1.5 Hz, 1H), 6.42 (dt, J = 15.9, 6.4 Hz, 1H), 4.99 (dd, J = 6.4, 1.4 Hz, 2H).

^{13}C{^{1}H} NMR (101 MHz, CDCl$_3$): δ 166.2, 136.1, 134.1, 132.8, 130.1, 129.5, 128.5, 128.2, 127.9, 126.5, 123.1, 65.4.

But-2-en-1-yl benzoate (1g)

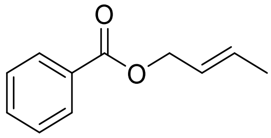

Following **GP-I**, starting from 0.86 mL (10 mmol, 1 equiv.) of but-2-en-1-ol, 122 mg (1 mmol, 10% mol) of 4-dimethylaminopyridine (DMAP), 1.44 mL (10 mmol, 1 equiv.) of triethylamine and 1.40 mL (12 mmol, 1.2 equiv.) of benzoyl chloride in 20 mL of dry DCM. The crude mixture was purified using silica gel chromatography (95:5, cyclohexane/ethyl acetate) to afford 1.20 g of **1g** (6.8 mmol, 68% yield) as a colorless oil. Spectroscopic data for **1g** are in accordance with literature.[S18]

^{1}H NMR (400 MHz, CDCl$_3$): δ 8.09 – 8.01 (m, 2H), 7.59 – 7.51 (m, 1H), 7.48 – 7.39 (m, 2H), 5.95 – 5.84 (m, 1H), 5.79 – 5.65 (m, 1H), 4.76 (dt, J = 6.4, 1.2 Hz, 2H), 1.76 (dq, J = 6.5, 1.3 Hz, 3H).

^{13}C{^{1}H} NMR (101 MHz, CDCl$_3$): δ 166.3, 132.7, 131.2, 130.3, 129.4, 128.2, 125.0, 65.5, 17.6.

***N*-(Pent-4-en-1-yl)benzamide (1h)**

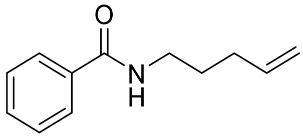

Following **GP-II**, starting from 1.24 mL (10.4 mmol, 1.1 equiv.) of benzoyl chloride, 1.00 mL (9.4 mmol, 1 equiv.) of pent-4-en-1-amine and 1.44 mL (10.4 mmol, 1.1 equiv.) of triethylamine in 10 mL of dry DCM. The crude mixture was purified using silica gel chromatography (8:2, cyclohexane/ethyl acetate) to afford 1.70

g of **1h** (9.2 mmol, 98% yield) as a colorless oil. Spectroscopic data for **1h** are in accordance with literature.[S19]

^{1}H NMR (400 MHz, CDCl$_3$): δ 7.78 – 7.71 (m, 2H), 7.54 – 7.47 (m, 1H), 7.47 – 7.39 (m, 2H), 6.13 (bs, 1H), 5.92 – 5.81 (m, 1H), 5.15 – 4.98 (m, 2H), 3.49 (td, *J* = 7.1, 5.8 Hz, 2H), 2.23 – 2.12 (m, 2H), 1.80 – 1.66 (m, 2H).

^{13}C{^{1}H} NMR (101 MHz, CDCl$_3$): δ 167.3, 137.7, 134.7, 131.2, 128.4, 126.6, 115.2, 39.5, 31.1, 28.6.

N-Allylbenzamide (1i)

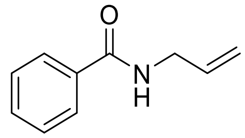

Following **GP-II**, starting from 1.24 mL (10.4 mmol, 1.1 equiv.) of benzoyl chloride, 0.70 mL (9.4 mmol, 1 equiv.) of allyl amine and 1.44 mL (10.4 mmol, 1.1 equiv.) of triethylamine in 10 mL of dry DCM. The crude mixture was purified using silica gel chromatography (1:1, cyclohexane/ethyl acetate) to afford 1.13 g of **1i** (7.1 mmol, 75% yield) as a pale yellow oil. Spectroscopic data for **1i** are in accordance with literature.[S20]

^{1}H NMR (400 MHz, CDCl$_3$): δ 7.82 – 7.75 (m, 2H), 7.55 – 7.39 (m, 3H), 6.22 (bs, 1H), 6.02 – 5.91 (m, 1H), 5.32 – 5.15 (m, 2H), 4.10 (tt, *J* = 5.7, 1.6 Hz, 2H).

^{13}C{^{1}H} NMR (101 MHz, CDCl$_3$): δ 167.8, 135.0, 134.6, 132.0, 129.1, 127.4, 117.2, 42.9.

N-Allyl-1-naphtamide (1j)

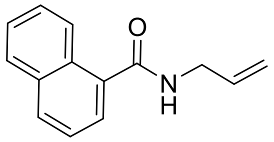

Following **GP-II**, starting from 1.55 mL (10.4 mmol, 1.1 equiv.) of 1-naphtoyl chloride, 0.70 mL (9.4 mmol, 1 equiv.) of allyl amine and 1.44 mL (10.4 mmol, 1.1 equiv.) of triethylamine in 10 mL of dry DCM. The crude mixture was purified using silica gel chromatography (1:1, cyclohexane/ethyl acetate) to afford 1.50 g of **1j** (7.1 mmol, 75%) as a pale yellow oil. Spectroscopic data for **1j** are in accordance with literature.[S21]

^{1}H NMR (400 MHz, CDCl$_3$): δ 8.33 – 8.25 (m, 1H), 7.93 – 7.81 (m, 2H), 7.62 – 7.47 (m, 3H), 7.42 (dd, *J* = 8.3, 7.0 Hz, 1H), 6.04 – 5.92 (m, 1H), 5.28 (dq, *J* = 17.1, 1.6 Hz, 1H), 5.19 (dq, *J* = 10.2, 1.4 Hz, 1H), 4.13 (tt, *J* = 5.8, 1.6 Hz, 2H).

^{13}C{^{1}H} NMR (101 MHz, CDCl$_3$): δ 169.2, 134.2, 133.8, 133.5, 130.4, 130.0, 128.1, 126.9, 126.2, 125.2, 124.7, 124.5, 116.5, 42.2.

N-Allyl-4-methylbenzenesulfonamide (1k)

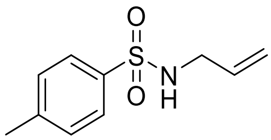

Following **GP-II**, starting from 2.00 g (10.4 mmol, 1.1 equiv.) of 4-methylbenzenesulfonyl chloride, 0.70 mL (9.4 mmol, 1 equiv.) of allyl amine and 1.44 mL (10.4 mmol, 1.1 equiv.) of triethylamine in 10 mL of dry DCM. The crude mixture was purified using silica gel chromatography (1:1, cyclohexane/ethyl acetate) to afford 1.40 g of **1k** (6.5 mmol, 69%) as a white solid. Spectroscopic data for **1k** are in accordance with literature.[S22]

^{1}H NMR (400 MHz, CDCl$_3$): δ 7.79 – 7.72 (m, 2H), 7.34 – 7.26 (m, 2H), 5.78 – 5.66 (m, 1H), 5.08 (dq, *J* = 10.3, 1.3 Hz, 1H), 4.71 (bs, 1H), 3.57 (d, *J* = 5.9 Hz, 2H), 2.42 (s, 3H).

^{13}C{^{1}H} NMR (101 MHz, CDCl$_3$): δ 143.3, 136.8, 132.8, 129.5, 127.0, 117.5, 45.6, 21.3.

1-Allyl-pyrrolidin-2-one (1l)

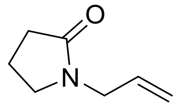

Following **GP-III**, starting from 1.70 mL (15 mmol, 1.5 equiv.) of pyrrolidine-2-one, 900 mg (16 mmol, 1.6 equiv.) of KOH and 0.85 mL of allyl bromide in 15 mL of THF. The crude mixture was purified using silica gel chromatography (1:1, cyclohexane/ethyl acetate) to afford 0.89 g of **1l** (7.1 mmol, 71%) as a pale yellow oil. Spectroscopic data for **1l** are in accordance with literature.[S10]

^{1}H NMR (400 MHz, CDCl$_3$): δ 5.74 – 5.73 (m, 1H), 5.22 – 5.13 (m, 2H), 3.89 (dt, *J* = 6.1, 1.5 Hz, 2H), 3.39 – 3.31 (m, 2H), 2.42 (t, *J* = 8.1 Hz, 2H), 2.08 – 1.96 (m, 2H).

^{13}C{^{1}H} NMR (101 MHz, CDCl$_3$): δ 174.6, 132.3, 117.7, 46.6, 45.1, 30.8, 17.6.

N-(2-Methylallyl)benzamide (1m)

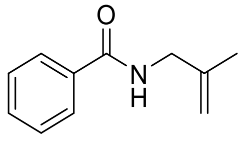

Following **GP-II**, starting from 1.24 mL (10.4 mmol, 1.1 equiv.) of benzoyl chloride, 0.86 mL (9.4 mmol, 1 equiv.) of 2-methylprop-2-en-1-amine and 1.44 mL (10.4 mmol, 1.1 equiv.) of triethylamine in 10 mL of dry DCM. The crude mixture was purified using silica gel chromatography (8:2, cyclohexane/ethyl acetate) to afford 1.60 g of **1m** (8.9 mmol, 87% yield) as a white solid. Spectroscopic data for **1m** are in accordance with literature.[S23]

^{1}H NMR (400 MHz, CDCl$_3$): δ 7.83 – 7.75 (m, 2H), 7.53 – 7.46 (m, 1H), 7.46 – 7.35 (m, 2H), 6.39 (bs, 1H), 4.93 – 4.84 (m, 2H), 4.04 – 3.97 (m, 2H), 1.78 (t, *J* = 1.2 Hz, 3H).

^{13}C{^{1}H} NMR (101 MHz, CDCl$_3$): δ 167.2, 141.8, 134.4, 131.3, 128.4, 126.7, 110.9, 45.3, 20.2.

1-(3-Methylbut-2-en-1-yl)pyrrolidin-2-one (1n)

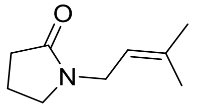

Following **GP-III**, starting from 1.70 mL (15 mmol, 1.5 equiv.) of pyrrolidine-2-one, 900 mg (16 mmol, 1.6 equiv.) of KOH and 1.16 mL of 1-bromo-3-methylbut-2-ene in 15 mL of THF. The crude mixture was purified using silica gel chromatography (1:1, Cyclohexane/Ethyl acetate) to afford 1.41 g of **1n** (9.2mmol, 92% yield) as a yellowish oil.

^{1}H NMR (400 MHz, $CDCl_3$): δ 5.12 (tt, *J* = 7.3, 1.4 Hz, 1H), 3.86 (d, *J* = 7.2 Hz, 2H), 3.36 – 3.27 (m, 2H), 2.37 (t, *J* = 8.1 Hz, 2H), 2.04 – 1.92 (m, 2H), 1.72 (s, 3H), 1.69 (s, 3H).

$^{13}C\{^1H\}$ NMR (101 MHz, $CDCl_3$): δ 174.2, 136.7, 118.5, 46.4, 39.9, 30.9, 25.5, 17.6, 17.6.

HRMS (EI, m/z): calcd. for $C_9H_{16}NO$ $[M+H]^+$ 154.1226, found 154.1226.

Prop-2-yn-1-yl benzoate (1u)

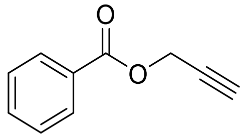

Following **GP-I**, starting from 0.6 mL (10 mmol, 1 equiv.) of prop-2-yn-1-ol, 122 mg (1 mmol, 10% mol) of 4-dimethylaminopyridine (DMAP), 1.44 mL (10 mmol, 1 equiv.) of triethylamine and 1.40 mL (12 mmol, 1.2 equiv.) of benzoyl chloride in 20 mL of dry DCM. The crude mixture was purified using silica gel chromatography (95:5, Cyclohexane/Ethyl acetate) to afford 1.51 g of **1u** (9.7 mmol, 97% yield) as a colorless oil. Spectroscopic data for **1u** are in accordance with literature.[S24]

^{1}H NMR (400 MHz, $CDCl_3$): δ 8.12 – 8.04 (m, 2H), 7.63 – 7.54 (m, 1H), 7.50 – 7.41 (m, 2H), 4.93 (d, *J* = 2.5 Hz, 2H), 2.52 (t, *J* = 2.5 Hz, 1H).

$^{13}C\{^1H\}$ NMR (101 MHz, $CDCl_3$): δ 166.0, 133.5, 130.0, 129.6, 128.6, 77.9, 75.1, 52.6.

But-3-yn-1-yl benzoate (1v)

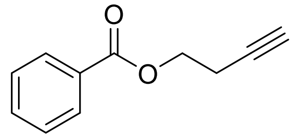

Following **GP-I**, starting from 0.75 mL (10 mmol, 1 equiv.) of but-3-yn-1-ol, 122 mg (1 mmol, 10% mol) of 4-dimethylaminopyridine (DMAP), 1.44 mL (10 mmol, 1 equiv.) of triethylamine and 1.40 mL (12 mmol, 1.2 equiv.) of benzoyl chloride in 20 mL of dry DCM. The crude mixture was purified using silica gel chromatography (95:5, cyclohexane/ethyl acetate) to afford 1.51 g of **1v** (0.87 mmol, 87% yield) as a colorless oil. Spectroscopic data for **1v** are in accordance with literature.[S25]

^{1}H NMR (400 MHz, CDCl$_3$): δ 8.10 – 8.03 (m, 2H), 7.61 – 7.52 (m, 1H), 7.49 – 7.40 (m, 2H), 4.43 (t, J = 6.8 Hz, 2H), 2.68 (td, J = 6.8, 2.7 Hz, 2H), 2.03 (t, J = 2.7 Hz, 1H).

^{13}C{^{1}H} NMR (101 MHz, CDCl$_3$): δ 166.2, 133.0, 129.8, 129.5, 128.2, 79.9, 69.8, 62.4, 19.0.

But-2-yn-1-yl benzoate (1w)

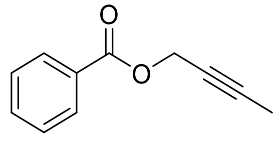

Following **GP-I**, starting from 0.75 mL (10 mmol, 1 equiv.) of but-2-yn-1-ol, 122 mg (1 mmol, 10% mol) of 4-dimethylaminopyridine (DMAP), 1.44 mL (10 mmol, 1 equiv.) of triethylamine and 1.40 mL (12 mmol, 1.2 equiv.) of benzoyl chloride in 20 mL of dry DCM. The crude mixture was purified using silica gel chromatography (95:5, cyclohexane/ethyl acetate) to afford 1.68 g of **1w** (9.6 mmol, 96% yield) as a colorless oil. Spectroscopic data for **1w** are in accordance with literature.[S24]

^{1}H NMR (400 MHz, CDCl$_3$): δ 8.11 – 8.04 (m, 2H), 7.61 – 7.52 (m, 1H), 7.49 – 7.40 (m, 2H), 4.89 (q, J = 2.4 Hz, 2H), 1.88 (t, J = 2.4 Hz, 3H).

^{13}C{^{1}H} NMR (101 MHz, CDCl$_3$): δ 165.9, 133.0, 129.6, 128.2, 83.1, 73.1, 53.1, 3.5.

3-Phenylprop-2-yn-1-yl benzoate (1x)

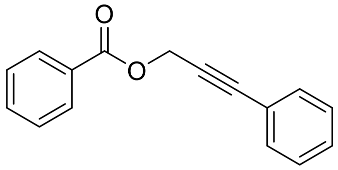

Following **GP-I**, starting from 1.25 mL (10 mmol, 1 equiv.) of 3-phenylprop-2-yn-1-ol, 122.00 mg (1 mmol, 10% mol) of 4-dimethylaminopyridine (DMAP), 1.44 mL (10 mmol, 1 equiv.) of triethylamine and 1.40 mL (12 mmol, 1.2 equiv.) of benzoyl chloride in 20 mL of dry DCM. The crude mixture was purified using silica gel chromatography (95:5, cyclohexane/ethyl acetate) to afford 1.6 g of **1x** (6.7 mmol, 67% yield) as a colorless oil. Spectroscopic data for **1x** are in accordance with literature.[S26]

^{1}H NMR (400 MHz, CDCl$_3$): δ 8.11 – 8.01 (m, 2H), 7.58 – 7.46 (m, 1H), 7.43 – 7.34 (m, 4H), 7.29 – 7.22 (m, 3H), 5.10 (s, 2H).

^{13}C{^{1}H} NMR (101 MHz, CDCl$_3$): δ 166.3, 133.6, 132.3, 130.2, 130.0, 129.1, 128.8, 128.7, 122.6, 87.0, 83.5, 53.7.

***N*-(Prop-2-yn-1-yl)benzamide (1y)**

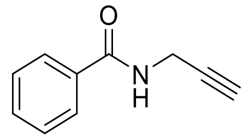

Following **GP-II**, starting from 1.24 mL (10.4 mmol, 1.1 equiv.) of benzoyl chloride, 0.60 mL (9.4 mmol, 1 equiv.) of 2-propynylamine and 1.44 mL (10.4 mmol, 1.1 equiv.) of triethylamine in 10 mL of dry DCM. The crude mixture was purified using silica gel chromatography (1:1, cyclohexane/ethyl acetate) to afford 1.44 g of **1y** (9.0 mmol, 96% yield) as a white solid. Spectroscopic data for **1y** are in accordance with literature.[S27]

^{1}H NMR (400 MHz, CDCl$_3$): δ 7.82 – 7.75 (m, 2H), 7.56 – 7.48 (m, 1H), 7.48 – 7.39 (m, 2H), 6.32 (bs, 1H), 4.26 (dd, *J* = 5.2, 2.6 Hz, 2H), 2.28 (t, *J* = 2.6 Hz, 1H).

^{13}C{^{1}H} NMR (101 MHz, CDCl$_3$): δ 166.9, 133.6, 131.6, 128.5, 126.8, 79.3, 71.7, 29.6.

***N*-(But-3-yn-1-yl)benzamide (1z)**

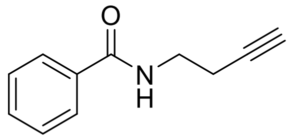

Following **GP-II**, starting from 1.24 mL (10.4 mmol, 1.1 equiv.) of benzoyl chloride, 0.81 mL (9.4 mmol, 1 equiv.) of but-3-yn-1-amine and 1.44 mL (10.4 mmol, 1.1 equiv.) of triethylamine in 10 mL of dry DCM. The crude mixture was purified ed using silica gel chromatography (1:1, cyclohexane/ethyl acetate) to afford 1.54 g of **1z** (8.9 mmol, 89% yield) as a white solid. Spectroscopic data for **1z** are in accordance with literature.[S28]

^{1}H NMR (400 MHz, CDCl$_3$): δ 7.82 – 7.72 (m, 2H), 7.60 – 7.48 (m, 1H), 7.47 – 7.38 (m, 2H), 6.50 (bs, 1H), 3.62 (q, *J* = 6.2 Hz, 2H), 2.53 (td, *J* = 6.4, 2.7 Hz, 2H), 2.05 (t, *J* = 2.6 Hz, 1H).

^{13}C{^{1}H} NMR (101 MHz, CDCl$_3$): δ 167.4, 134.3, 131.4, 128.4, 126.7, 81.4, 70.0, 38.2, 19.3.

4-Methyl-N-(prop-2-yn-1-yl)benzenesulfonamide (1aa)

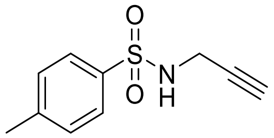

Following **GP-II**, starting from 2.00 g (10.4 mmol, 1.1 equiv.) of 4-methylbenzenesulfonyl chloride, 0.60 mL (9.4 mmol, 1 equiv.) of 2-propynylamine and 1.44 mL (10.4 mmol, 1.1 equiv.) of triethylamine in 10 mL of dry DCM The crude mixture was purified using silica gel chromatography (1:1, cyclohexane/ethyl acetate) to afford 1.90 g of **1aa** (9.0 mmol, 96% yield) as a white solid. Spectroscopic data for **1aa** are in accordance with literature.[S29]

^{1}H NMR (400 MHz, CDCl$_3$): δ 7.81 – 7.73 (m, 2H), 7.35 – 7.28 (m, 2H), 4.60 (bs, 1H), 3.83 (dd, *J* = 6.1, 2.6 Hz, 2H), 2.43 (s, 3H), 2.11 (t, *J* = 2.5 Hz, 1H).

^{13}C{^{1}H} NMR (101 MHz, CDCl$_3$): δ 143.7, 136.4, 129.5, 127.2, 77.8, 72.8, 32.7, 21.4.

(1R,2R,4R)-2-(Allyloxy)-1-isopropyl-4-methylcyclohexane (1ac)

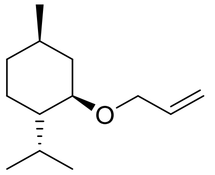

Following **GP-IV**, starting from 1.17 g (7.5 mmol, 1 equiv.) of (1R,2R,5R)-2-isopropyl-5-methylcyclohexan-1-ol, 0.90 g (22.5 mmol, 3 equiv.) of sodium hydride and 1.05 mL of allyl bromide (11.2 mmol, 1.5 equiv.) in 10 mL of dry DMF. The crude mixture was purified using silica gel chromatography (95:5, cyclohexane/ethyl acetate) to afford 1.17 g of **1ac** (4.2 mmol, 85% yield) as a colorless oil. Spectroscopic data for **1ac** are in accordance with literature.[S11]

^{1}H NMR (400 MHz, $CDCl_3$): δ 5.93 (ddt, J = 17.2, 10.3, 5.7 Hz, 1H), 5.26 (dq, J = 17.2, 1.7 Hz, 1H), 5.13 (dq, J = 10.3, 1.4 Hz, 1H), 4.12 (ddt, J = 12.6, 5.6, 1.4 Hz, 1H), 3.88 (ddt, J = 12.6, 5.8, 1.4 Hz, 1H), 3.08 (td, J = 10.5, 4.2 Hz, 1H), 2.31 – 2.15 (m, 1H), 2.09 (dtd, J = 12.1, 3.8, 2.0 Hz, 1H), 1.70 – 1.60 (m, 2H), 1.41 – 1.31 (m, 1H), 1.31 – 1.19 (m, 1H), 1.05 – 0.80 (m, 8H), 0.77 (d, J = 6.9 Hz, 3H).

^{13}C{^{1}H} NMR (101 MHz, $CDCl_3$): δ 136.3, 116.8, 79.2, 70.0, 48.8, 41.0, 35.1, 32.0, 26.0, 23.9, 22.8, 21.5, 16.7.

3,7-Dimethyloct-6-en-1-yl benzoate (1ad)

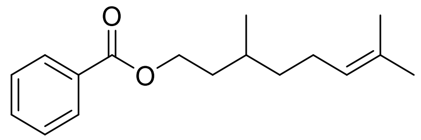

Following **GP-I**, starting from 1.81 mL (10 mmol, 1 equiv.) of 3,7-dimethyloct-6-en-1-ol, 122 mg (1 mmol, 10% mol) of 4-dimethylaminopyridine (DMAP), 1.44 mL (10 mmol, 1 equiv.) of triethylamine and 1.40 mL (12 mmol, 1.2 equiv.) of benzoyl chloride in 20 mL of dry DCM. The crude mixture was purified using silica gel chromatography (95:5, cyclohexane/ethyl acetate) to afford 1.37 g of **1ad** (5.2 mmol, 52% yield) as a colorless oil. Spectroscopic data for **1ad** are in accordance with literature.[S30]

^{1}H NMR (400 MHz, $CDCl_3$): δ 8.08 – 8.00 (m, 2H), 7.60 – 7.51 (m, 1H), 7.48 – 7.39 (m, 2H), 5.12 – 5.08 (m, 1H), 4.38 – 4.29 (m, 2H), 2.11 – 1.91 (m, 2H), 1.84 – 1.78 (m, 1H), 1.70 – 1.52 (m, 8H), 1.44 – 1.37 (m, 1H), 1.31 – 1.20 (m, 1H), 0.97 (d, J = 6.5 Hz, 3H).

^{13}C{^{1}H} NMR (101 MHz, $CDCl_3$): δ 167.2, 133.3, 131.9, 131.0, 130.0, 128.8, 125.0, 64.0, 37.5, 36.0, 30.0, 26.2, 25.8, 20.0, 18.1.

Pent-4-en-1yl 4-([1,1'-biphenyl]-4-yl)-4-oxobutanoate (1ae)

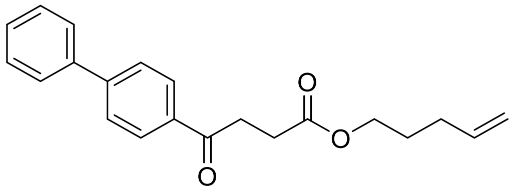

Following **GP-V**, starting from 1.27 g (5 mmol, 1 equiv.) of 4-([1,1'-biphenyl]-4-yl)-4-oxobutanoic acid, 1.00 g (6.5 mmol, 1.3 equiv.) of 1-(3-dimethylaminopropyl)-3-ethylcarbodiimide (EDC), 61 mg (0.5 mmol, 0.1 equiv.) of 4-dimethylaminopyridine (DMAP) and 0.52 mL (6 mmol, 1.2 equiv.) of pent-4-en-1-ol in 10 mL of DCM. The crude mixture was purified using silica gel chromatography (95:5, cyclohexane/ethyl acetate) to obtain 0.97 g of **1ae** (3.0 mmol, 60% yield) as a colorless dense oil.

^{1}H NMR (400 MHz, CDCl$_3$): δ 8.09 – 8.03 (m, 2H), 7.72 – 7.67 (m, 2H), 7.65 – 7.61 (m, 2H), 7.52 – 7.44 (m, 2H), 7.44 – 7.36 (m, 1H), 5.88 – 5.76 (m, 1H), 5.09 – 4.95 (m, 2H), 4.13 (t, J = 6.7 Hz, 2H), 3.35 (t, J = 6.6 Hz, 2H), 2.79 (t, J = 6.7 Hz, 2H), 2.19 – 2.08 (m, 2H), 1.78 – 1.71 (m, 2H).

^{13}C{^{1}H} NMR (101 MHz, CDCl$_3$): δ 197.5, 172.8, 145.7, 139.7, 137.3, 135.1, 128.8, 128.5, 128.1, 127.1, 115.1, 64.0, 33.2, 29.8, 28.1, 27.6.

HRMS (EI, m/z): calcd. for $C_{21}H_{23}O_3$ $[M+H]^+$ 323.1642, found 323.1640.

Pent-4-en-1-yl 2-(4-isobutylphenyl)propanoate (1af)

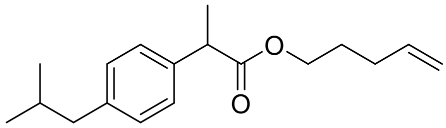

Following **GP-V**, starting from 1.03 g (5 mmol, 1 equiv.) of 2-(4-isobutylphenyl)propanoic acid, 1.00 g (6.5 mmol, 1.3 equiv.) of 1-(3-dimethylaminopropyl)-3-ethylcarbodiimide (EDC), 61 mg (0.5 mmol, 0.1 equiv.) of 4-dimethylaminopyridine (DMAP) and 0.52 mL (6 mmol, 1.2 equiv.) of pent-4-en-1-ol in 10 mL of DCM. The crude mixture was purified using silica gel chromatography (95:5, cyclohexane/ethyl acetate) to afford 1.17 g of **1af** (4.2 mmol, 85% yield) as a colorless oil. Spectroscopic data for **1af** are in accordance with literature.[S31]

^{1}H NMR (400 MHz, CDCl$_3$): δ 7.24 – 7.16 (m, 2H), 7.13 – 7.05 (m, 2H), 5.80 – 5.68 (m, 1H), 4.99 – 4.89 (m, 2H), 4.12 – 4.02 (m, 2H), 3.68 (q, J = 7.1 Hz, 1H), 2.44 (d, J = 7.2 Hz, 2H), 2.06 – 1.98 (m, 2H), 1.84 (dt, J = 13.6, 6.8 Hz, 1H), 1.68 (dt, J = 7.9, 6.6 Hz, 2H), 1.49 (d, J = 7.2 Hz, 3H), 0.89 (d, J = 6.6 Hz, 6H).

^{13}C{^{1}H} NMR (101 MHz, CDCl$_3$): δ 175.2, 141.0, 138.3, 137.9, 129.8, 127.6, 115.7, 64.5, 45.7, 45.5, 30.6, 30.4, 28.2, 22.8, 18.9.

3-Methylbut-2-en-1-yl acetyl-L-phenylalaninate (1ag)

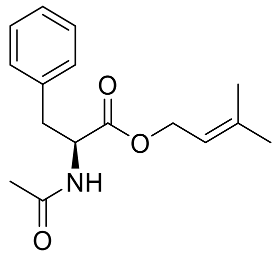

Following **GP-VI**, starting from 1.00 g (5 mmol, 1 equiv.) of *N*-Acetyl-L-phenylalanine, 1.10 mL (6.5 mmol, 1.3 equiv.) of Hunig's base and 0.76 mL (6.5 mmol, 1.3 equiv.) of 1-bromo-3-methylbut-2-ene in DMF. The crude mixture was purified using silica gel chromatography (7:3, cyclohexane/ethyl acetate) to afford 0.98 g of **1ag** (3.5 mmol, 70% yield) as a colorless oil.

^{1}H NMR (400 MHz, CDCl$_3$): δ 7.33 – 7.18 (m, 3H), 7.12 – 7.05 (m, 2H), 5.96 (d, *J* = 7.9 Hz, 1H), 5.32 – 5.29 (m, 1H), 4.87 (dt, *J* = 7.9, 5.6 Hz, 1H), 4.68 – 4.54 (m, 2H), 3.19 – 3.04 (m, 2H), 1.97 (s, 3H), 1.77 (d, *J* = 1.4 Hz, 3H), 1.71 (d, *J* = 1.4 Hz, 3H).

^{13}C{^{1}H} NMR (101 MHz, CDCl$_3$): δ 172.0, 170.0, 140.5, 136.3, 129.9, 128.9, 127.5, 118.4, 62.8, 53. 6, 38.3, 26.2, 23.7, 18.5.

HRMS (EI, m/z): calcd. for $C_{16}H_{22}NO_3$ $[M+H]^+$ 276.1594, found 276.1597.

N,N-Diallyl 4-methylbenzenesulfonamide (1ah)

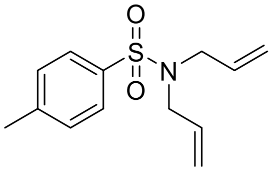

Following **GP-III**, starting from 1.0 g (5 mmol, 1.5 equiv.) of *N*-Allyl 4-methylbenzenesulfonamide (**1k**), 300 mg (5.3 mmol, 1.6 equiv.) of KOH and 0.53 mL of propargyl bromide (3.3 mmol, 1 equiv.) in 5 mL of THF. The crude mixture was purified using silica gel chromatography (1:1, cyclohexane/ethyl acetate) to afford 0.80 g of **1ah** (3.2 mmol, 97% yield) as a colorless oil. Spectroscopic data for **1ah** are in accordance with literature.[S6]

^{1}H NMR (400 MHz, CDCl$_3$): δ 7.74 – 7.67 (m, 2H), 7.29 (d, *J* = 8.0 Hz, 2H), 5.68 – 5.57 (m, 2H), 5.19 – 5.08 (m, 4H), 3.80 (dt, *J* = 6.2, 1.3 Hz, 4H), 2.43 (s, 3H).

^{13}C{^{1}H} NMR (101 MHz, CDCl$_3$): δ 143.0, 137.3, 132.5, 129.5, 127.0, 118.8, 49.2, 21.3.

3. Photocatalyzed ATRA reactions

3.1 General procedures for photocatalyzed ATRA reactions (GP-*a* – GP-*c*)

Typical procedure for small scale reactions (GP-*a*):

In a glass irradiation vial (1-dram) equipped with a magnetic microstir bar, the chosen unsaturated compound (**1a**, 0.1 mmol, 0.1 M) and the alkyl halide (**2a**, 2.0 equiv.) were dissolved in the chosen solvent mixture (95:5 MeCN/H_2O, 1 mL) in the presence of 5 mg of the *g*-C_3N_4/$Cs_2AgBiCl_6$ catalyst (90:10$_{W/W}$, **CAT2**). After bubbling N_2 for 1 minute, the vial was capped, and the resulting mixture was then placed in a home-made reactor equipped with a side fan and irradiated for 24h with a Kessil lamp (λ_{em} = 427 nm, 40W; see **Figure S10**) at room temperature (*ca*. 20/25°C). After solvent removal under vacuo, the amount of product formed, and residual unsaturated starting material was monitored via ^1H-NMR using dimethyl fumarate as internal standard (0.1 mmol, 0.1 M).

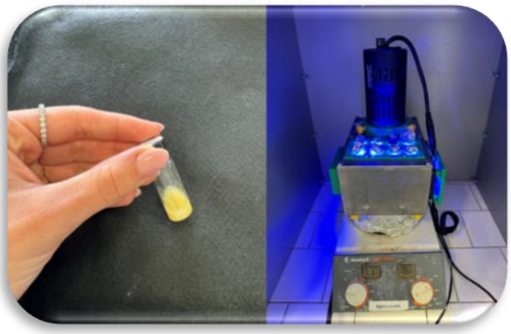

Figure S10. Left part: glass irradiation vial containing the reaction mixture. Right part: experimental apparatus used for small scale experiments, consisting of a home-made photoreactor equipped with a Kessil Lamp (λ_{em} = 427 nm, 40W).

Typical procedure for preparative reactions (GP-*b*):

In a Pyrex glass vessel equipped with a magnetic stir bar, the chosen unsaturated compound (**1**; 0.5 mmol, 0.1 M) and alkyl halide (**2**, 2.0 equiv., except where indicated otherwise; 4.0 equiv. of **2** have been used for alkyne-type unsaturated compounds) were dissolved in a 95:5 acetonitrile/water mixture (5 mL) in the presence of 25 mg of the *g*-C_3N_4/$Cs_2AgBiCl_6$ catalyst (90:10$_{W/W}$, **CAT2**). After bubbling N_2 for 3 minutes, the vial was capped, and the resulting mixture was irradiated at room temperature (*ca*. 20/25°C) in a box equipped with a side fan for 24h with a Kessil lamp (λ_{em} = 427 nm, 40W; see **Figure S11**) kept at *ca*. 10 cm from the flask. After solvent removal under vacuo, the crude mixture was purified by column chromatography (silica gel; cyclohexane/ethyl acetate mixture as eluant) to afford the pure product.

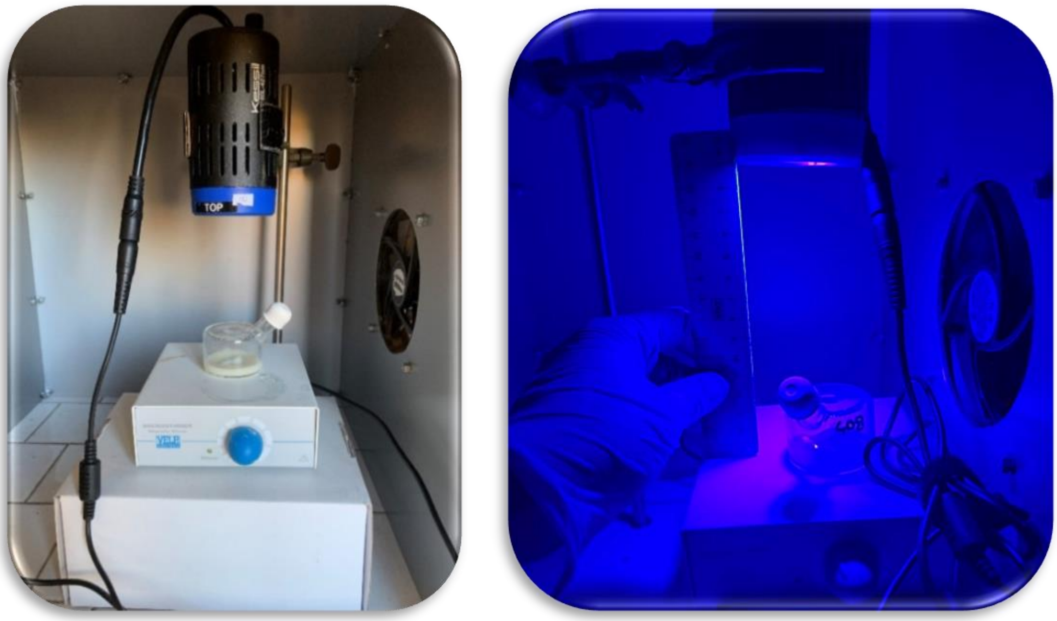

Figure S11. Typical reaction setup adopted for preparative experiments, consisting of a Pyrex glass vessel containing the reaction mixture and equipped with a magnetic stir bar, irradiated from the top with a Kessil Lamp (λ_{em} = 427 nm, 40W) kept at *ca*. 10 cm from the glass vessel in a fan-cooled box.

In selected instances, the same **GP-*b*** procedure has been adopted upon irradiation with natural sunlight (experiments were performed in Pavia, Italy, location: 45°11′07.01″N 9°09′18″E, in July 2024), as shown in **Figure S12**.

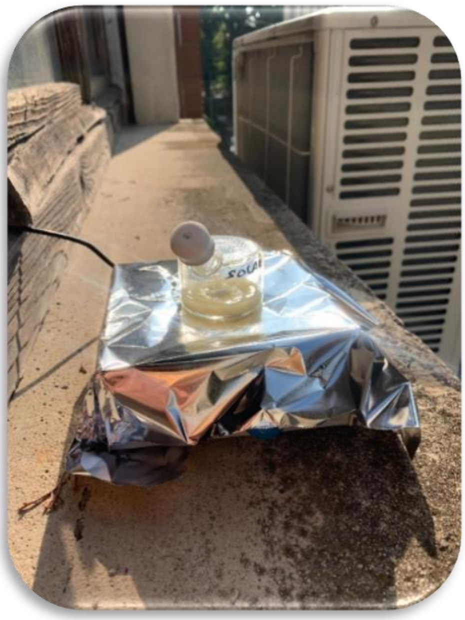

Figure S12. Setup adopted for natural sunlight induced preparative experiments.

Typical procedure for gram scale reactions (GP-*c*):

In a Pyrex round bottom flask equipped with a magnetic stir bar, the chosen unsaturated compound (**1**, 3 mmol, 0.1 M) and alkyl halide (**2**, 2.0 equiv., except where indicated otherwise) were dissolved in a 95:5 acetonitrile/water mixture (30 mL) in the presence of 150 mg of the *g*-$C_3N_4/Cs_2AgBiCl_6$ catalyst (90:10$_{W/W}$, **CAT2**). After bubbling N_2 for 3 minutes, the glass vessel was capped, and the resulting mixture was irradiated at room temperature (*ca*. 20/25°C) in a box equipped with a side fan with a Kessil lamp (λ_{em} = 427 nm, 40W; see **Figure S13**) kept at *ca*. 10 cm from the flask. The irradiation was monitored by TLC until completion (generally, 36h); after solvent removal under vacuo, the crude mixture was purified by column chromatography (silica gel; cyclohexane/ethyl acetate mixture as eluant) to afford the pure product.

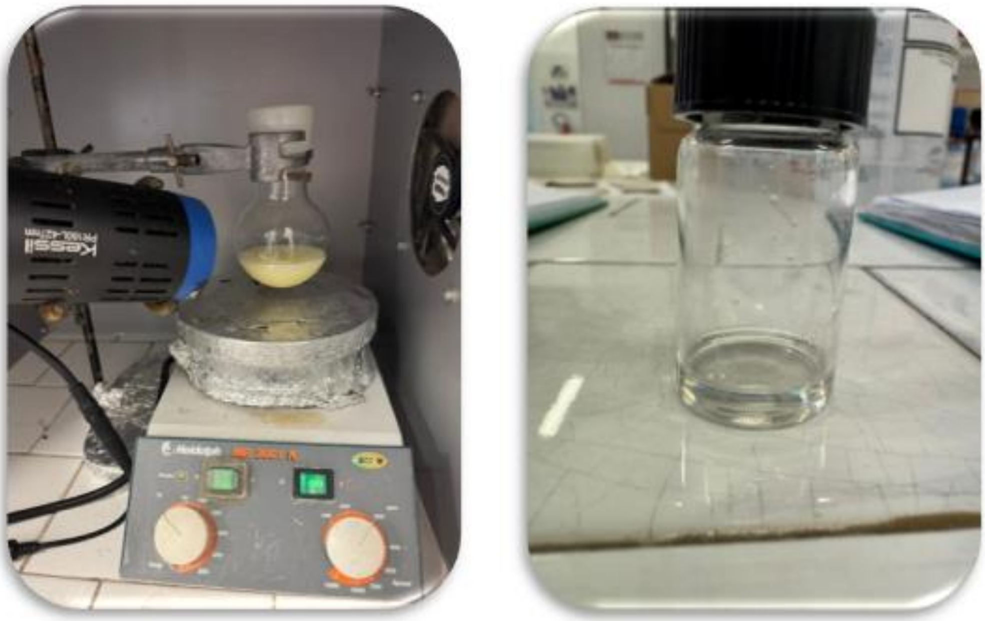

Figure S13. Left part: experimental setup adopted for gram-scale reactions, consisting of a Pyrex round bottom flask containing the solution mixture and equipped with a magnetic stir bar, irradiated with a Kessil Lamp (λ_{em} = 427 nm, 40W) kept at *ca*. 10 cm from the flask in a fan-cooled box. Right part: Vial with the obtained purified product.

3.2 Characterization data for products 3-66

4-Bromo-6,6,6-trichlorohexyl benzoate (3)

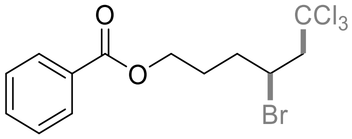

Following **GP-*b***, starting from 95 mg (0.5 mmol, 0.1M) of pent-4-en-1-yl-benzoate **(1a)**, 99 µL (1 mmol, 2.0 equiv.) of bromotrichloromethane (**2a**) and 25 mg of g-C_3N_4/$Cs_2AgBiCl_6$ (90%/10%) catalyst in 5 mL of acetonitrile:water (95:5) mixture. The crude mixture was purified using silica gel chromatography (95:5 cyclohexane/ethyl acetate) to afford 171 mg of **3** (0.44 mmol, 88% yield) as a colorless oil. Spectroscopic data for **3** are in accordance with literature.[S8]

The same reaction, following **GP-*b***, was also conducted by sunlight irradiation (16h, over 2 irradiation days), affording 170 mg (0.44 mmol, 88% yield) of product **3**.

Following **GP-*c***, starting from 570 mg (3.0 mmol, 0.1M) of pent-4-en-1-yl-benzoate (**1a**), 587 µL (6 mmol, 2.0 equiv.) of bromotrichloromethane (**2a**) and 150 mg of g-C_3N_4/$Cs_2AgBiCl_6$ (90%/10%) catalyst in 30 mL of acetonitrile:water (95:5) mixture. The crude mixture was purified using silica gel chromatography (95:5 cyclohexane/ethyl acetate) to afford 1.18 g of **3** (3.0 mmol, >99% yield).

^{1}H NMR (400 MHz, $CDCl_3$): δ 8.09 – 8.00 (m, 2H), 7.61 – 7.52 (m, 1H), 7.49 – 7.40 (m, 2H), 4.47 – 4.33 (m, 3H), 3.50 (dd, J = 15.8, 4.8 Hz, 1H), 3.25 (dd, J = 15.8, 5.7 Hz, 1H), 2.34 – 2.21 (m, 1H), 2.18 – 1.93 (m, 3H).

^{13}C{^{1}H} NMR (101 MHz, $CDCl_3$): δ 166.3, 132.9, 129.9, 129.4, 128.2, 96.8, 63.6, 62.4, 48.1, 35.9, 26.6.

4,6,6,6-Tetrabromohexyl benzoate (4)

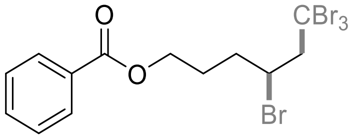

Following **GP-*b***, starting from 95 mg (0.5 mmol, 0.1M) of pent-4-en-1-yl-benzoate **(1a)**, 332 mg (1 mmol, 2.0 equiv.) of tetrabromomethane (**2b**) and 25 mg of g-C_3N_4/$Cs_2AgBiCl_6$ (90%/10%) catalyst in 5 mL of acetonitrile:water (95:5) mixture. The crude mixture was purified using silica gel chromatography (9:1 cyclohexane/ethyl acetate) to afford 200 mg of **4** (0.38 mmol, 76% yield) as a colorless oil.

^{1}H NMR (400 MHz, $CDCl_3$): δ 7.96 (dd, J = 8.4, 1.4 Hz, 2H), 7.48 (t, J = 7.5 Hz, 1H), 7.36 (t, J = 7.7 Hz, 2H), 4.40 – 4.24 (m, 2H), 4.27 – 4.14 (m, 1H), 3.80 (dd, J = 16.1, 4.3 Hz, 1H), 3.50 (dd, J = 16.1, 5.3 Hz, 1H), 2.27 – 2.19 (m, 1H), 2.13 – 1.82 (m, 3H).

$^{13}C\{^1H\}$ NMR (101 MHz, $CDCl_3$): δ 166.9, 133.5, 130.6, 130.0, 128.8, 67.2, 64.3, 51.6, 36.7, 36.3, 27.3.

HRMS (EI, m/z): calcd. for $C_{13}H_{15}Br_4O_2$ $[M+H]^+$ 518.7800, found 518.7805.

4-Bromo-6-cyanohexyl benzoate (5)

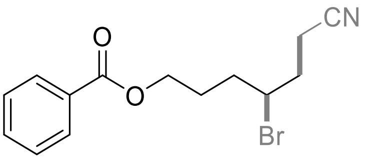

Following **GP-*b***, starting from 95 mg (0.5 mmol, 0.1M) of pent-4-en-1-yl-benzoate **(1a)**, 71 µL (1 mmol, 2.0 equiv.) of bromoacetonitrile (**2c**) and 25 mg of g-C_3N_4/$Cs_2AgBiCl_6$ (90%/10%) catalyst in 5 mL of acetonitrile:water (95:5) mixture. The crude mixture was purified using silica gel chromatography (8:2 cyclohexane/ethyl acetate) to afford 120.20 mg of **5** (0.41 mmol, 82% yield) as a colorless oil.

Following **GP-*c***, starting from 570 mg (3.0 mmol, 0.1M) of pent-4-en-1-yl-benzoate **(1a)**, 424 µL (6 mmol, 2.0 equiv.) of bromoacetonitrile (**2c**) and 150 mg of g-C_3N_4/$Cs_2AgBiCl_6$ (90%/10%) catalyst in 30 mL of acetonitrile:water (95:5) mixture. The crude mixture was purified using silica gel chromatography (8:2 cyclohexane/ethyl acetate) to afford 0.91 g of **5** (3.0 mmol, >99% yield).

^1H NMR (400 MHz, $CDCl_3$): δ 8.06 – 7.99 (m, 2H), 7.60 – 7.51 (m, 1H), 7.44 (dd, J = 8.4, 7.1 Hz, 2H), 4.43 – 4.28 (m, 2H), 4.15 – 4.00 (m, 1H), 2.74 – 2.42 (m, 2H), 2.26 – 1.85 (m, 6H).

$^{13}C\{^1H\}$ NMR (101 MHz, $CDCl_3$): δ 166.2, 132.8, 129.8, 129.3, 128.2, 118.4, 63.6, 53.9, 35.3, 34.4, 26.7, 15.8.

HRMS (EI, m/z): calcd. for $C_{14}H_{16}BrNNaO_2$ $[M+Na]^+$ 332.0257, found 332.0254.

Diethyl-2-(5-(benzoyloxy)-2-bromopentyl)malonate (6)

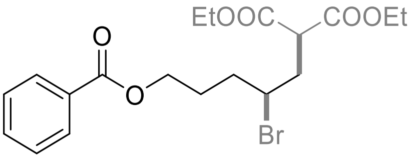

Following **GP-*b***, starting from 95 mg (0.5 mmol, 0.1M) of pent-4-en-1-yl-benzoate (**1a**), 171 µL (1 mmol, 2.0 equiv.) of diethyl bromomalonate (**2d**) and 25 mg of g-C_3N_4/$Cs_2AgBiCl_6$ (90%/10%) catalyst in 5 mL of acetonitrile:water (95:5) mixture. The crude mixture was purified using silica gel chromatography (9:1 cyclohexane/ethyl acetate) to afford 175 mg of **6** (0.41 mmol, 82% yield) as a colorless oil. Spectroscopic data for **6** are in accordance with literature.[S8]

^1H NMR (400 MHz, $CDCl_3$): δ 8.05 – 7.98 (m, 2H), 7.58 – 7.49 (m, 1H), 7.42 (dd, J = 8.4, 7.0 Hz, 2H), 4.33 (td, J = 5.6, 1.5 Hz, 2H), 4.24 – 4.12 (m, 4H), 4.09 – 4.02 (m, 1H), 3.77 (dd, J = 10.2, 4.2

Hz, 1H), 2.47 (ddd, J = 14.8, 10.2, 3.2 Hz, 1H), 2.28 (ddd, J = 14.8, 10.6, 4.3 Hz, 1H), 2.12 – 1.85 (m, 4H), 1.24 (td, J = 7.1, 2.6 Hz, 6H).

$^{13}C\{^1H\}$ NMR (101 MHz, $CDCl_3$): δ 168.6, 168.4, 166.2, 132.7, 130.0, 129.3, 128.1, 63.8, 61.4, 61.4, 53.8, 50.3, 37.7, 35.8, 26.7, 13.8, 13.7.

4-Bromo-7-ethoxy-6,6-difluoro-7-oxoheptyl benzoate (7)

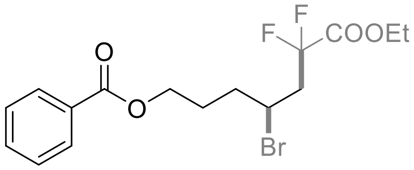

Following **GP-*b***, starting from 95 mg (0.5 mmol, 0.1M) of pent-4-en-1-yl-benzoate (**1a**), 128 μL (1 mmol, 2.0 equiv.) of ethyl 2-bromo-2,2-difluoroacetate (**2e**) and 25 mg of g-C_3N_4/$Cs_2AgBiCl_6$ (90%/10%) catalyst in 5 mL of acetonitrile:water (95:5) mixture. The crude mixture was purified using silica gel chromatography (9:1 cyclohexane/ethyl acetate) to afford 136 mg of **7** (0.35 mmol, 70% yield) as a colorless oil. Spectroscopic data for **7** are in accordance with literature.[S32]

^1H NMR (400 MHz, $CDCl_3$): δ 8.07 – 7.99 (m, 2H), 7.60 – 7.51 (m, 1H), 7.48 – 7.38 (m, 2H), 4.39 – 4.29 (m, 4H), 4.28 – 4.23 (m, 1H), 2.94 – 2.79 (m, 1H), 2.73 – 2.63 (m, 1H), 2.14 – 1.87 (m, 4H), 1.34 (t, J = 7.2 Hz, 3H).

$^{13}C\{^1H\}$ NMR (101 MHz, $CDCl_3$): δ 166.6, 163.5 (t, J = 32.2 Hz), 133.1, 130.2, 129.7, 128.5, 114.8 (dd, J = 253.6, 250.7 Hz), 64.0, 63.4, 45.6 (dd, J = 5.6, 3.7 Hz), 43.8 (t, J = 23.5 Hz), 35.9, 26.8, 14.0.

4-Bromo-7-ethoxy-7-oxoheptyl benzoate (8)

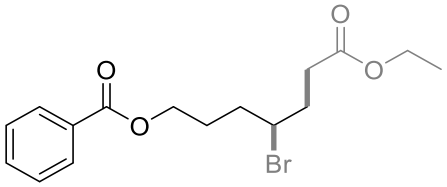

Following **GP- *b***, starting from 95 mg (0.5 mmol, 0.1M) of pent-4-en-1-yl-benzoate (**1a**), 112 μL (2 mmol, 4.0 equiv.) of ethyl bromoacetate (**2f**) and 25 mg of g-C_3N_4/$Cs_2AgBiCl_6$ (90%/10%) catalyst in 5 mL of acetonitrile:water (95:5) mixture. The crude mixture was purified using silica gel chromatography (8:2 cyclohexane/ethyl acetate) to afford 90 mg of **8** (0.25 mmol, 50% yield) as a colorless oil.

^1H NMR (400 MHz, $CDCl_3$): δ 8.08 – 7.99 (m, 2H), 7.60 – 7.50 (m, 1H), 7.49 – 7.39 (m, 2H), 4.35 (td, J = 5.7, 2.1 Hz, 2H), 4.13 (q, J = 7.2 Hz, 3H), 2.66 – 2.45 (m, 2H), 2.27 – 2.18 (m, 1H), 2.15 – 1.84 (m, 5H), 1.25 (t, J = 7.2 Hz, 3H).

$^{13}C\{^1H\}$ NMR (101 MHz, $CDCl_3$): δ 172.8, 166.7, 133.1, 130.3, 129.7, 128.5, 64.3, 60.7, 56.3, 36.0, 34.2, 32.5, 27.1, 14.3.

HRMS (EI, m/z): calcd. for $C_{16}H_{21}BrNaO_4$ $[M+Na]^+$ 379.0515, found 379.0516.

4-Bromo-6-nitrohexyl benzoate (9)

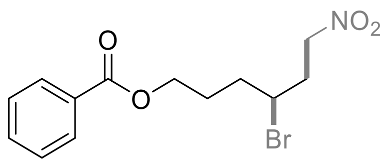

Following **GP-** ***b***, starting from 95 mg (0.5 mmol, 0.1M) of pent-4-en-1-yl-benzoate **(1a)**, 68 μL (1 mmol, 2.0 equiv.) of bromonitromethane (**2g**) and 25 mg of g-C_3N_4/$Cs_2AgBiCl_6$ (90%/10%) catalyst in 5 mL of acetonitrile:water (95:5) mixture. The crude mixture was purified using silica gel chromatography (9:1 cyclohexane/ethyl acetate) to afford 72 mg of **9** (0.22 mmol, 44% yield) as a colorless oil.

^{1}H NMR (400 MHz, CD_3COCD_3): δ 8.13 – 7.97 (m, 2H), 7.68 – 7.59 (m, 1H), 7.59 – 7.45 (m, 2H), 4.85 – 4.68 (m, 2H), 4.49 – 4.25 (m, 3H), 2.77 – 2.67 (m, 1H), 2.56 – 2.44 (m, 1H), 2.24 – 2.14 (m, 1H), 2.14 – 1.92 (m, 3H).

^{13}C{^{1}H} NMR (101 MHz, CD_3COCD_3): δ 166.8, 134.0, 131.5, 130.3, 129.5, 74.6, 64.9, 54.4, 37.0, 36.5, 27.8.

HRMS (EI, m/z): calcd. for $C_{13}H_{16}BrNNaO_4$ $[M+Na]^+$ 352.0155, found 352.0157.

4-Bromo-7-oxo-7phenylheptyl benzoate (10)

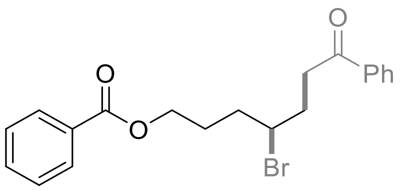

Following **GP-*b***, starting from 95 mg (0.5 mmol, 0.1M) of pent-4-en-1-yl-benzoate (**1a**), 199 mg (1 mmol, 2.0 equiv.) of phenacyl bromide (**2h**) and 25 mg of g-C_3N_4/$Cs_2AgBiCl_6$ (90%/10%) catalyst in 5 mL of acetonitrile:water (95:5) mixture. The crude mixture was purified using silica gel chromatography (98:2 cyclohexane/ethyl acetate) to afford 70 mg of **10** (0.18 mmol, 36% yield) as an orange oil.

^{1}H NMR (400 MHz, $CDCl_3$): δ 8.09 – 8.02 (m, 2H), 8.02 – 7.91 (m, 2H), 7.66 – 7.51 (m, 2H), 7.39 – 7.50 (m, 4H), 4.41 – 4.32 (m, 2H), 4.26 – 4.16 (m, 1H), 3.34 – 3.17 (m, 2H), 2.46 – 2.33 (m, 1H), 2.25 – 1.87 (m, 5H).

^{13}C{^{1}H} NMR (101 MHz, $CDCl_3$): δ 198.8, 166.4, 136.6, 133.1, 132.8, 130.1, 129.4, 128.5, 128.2, 127.9, 64.00, 56.9, 36.4, 36.0, 33.1, 26.9.

HRMS (EI, m/z): calcd. for $C_{20}H_{21}O_3$ $[M-Br]^+$ 309.1485, found 309.1485.

6,6,7,7,8,8,9,9,10,10,11,11,11-Tridecafluoro-4-iodoundecyl benzoate (11)

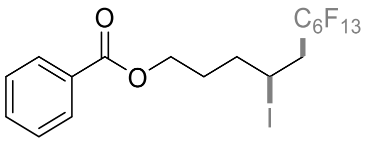

Following **GP-*b***, starting from 95 mg (0.5 mmol, 0.1M) of pent-4-en-1-yl-benzoate (**1a**), 217 µL (1 mmol, 2.0 equiv.) of perfluorohexyl iodide (**2i**) and 25 mg of g-C_3N_4/$Cs_2AgBiCl_6$ (90%/10%) catalyst in 5 mL of acetonitrile:water (95:5) mixture. The crude mixture was purified using silica gel chromatography (9:1 cyclohexane/ethyl acetate) to afford 190 mg of **11** (0.30 mmol, 60% yield) as a colorless oil. Spectroscopic data for **11** are in accordance with literature.[S8]

^{1}H NMR (400 MHz, $CDCl_3$): δ 8.04 (dd, *J* = 8.4, 1.4 Hz, 2H), 7.61 – 7.52 (m, 1H), 7.44 (dd, *J* = 8.4, 7.1 Hz, 2H), 4.46 – 4.34 (m, 3H), 3.06 – 2.71 (m, 2H), 2.15 – 1.85 (m, 4H).

^{13}C{^{1}H} NMR (101 MHz, $CDCl_3$): δ 166.3, 132.8, 129.9, 129.4, 128.2, 123.0 – 104.3 (m), 63.3, 41.5 (t, *J* = 20.9 Hz), 36.7 (d, *J* = 2.4 Hz), 28.8, 19.3.

4-Bromo-6,6,6-trichlorohexyl 4-methylbenzenesulfonate (12)

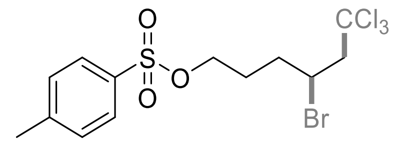

Following **GP-*b***, starting from 120 mg (0.5 mmol, 0.1M) of pent- 4-en-1-yl-4-methylbenzenesulfonate (**1b**), 99 µL (1 mmol, 2.0 equiv.) of bromotrichloromethane (**2a**) and 25 mg of g-C_3N_4/$Cs_2AgBiCl_6$ (90%/10%) catalyst in 5 mL of acetonitrile:water (95:5) mixture. The crude mixture was purified using silica gel chromatography (9:1 cyclohexane/ethyl acetate) to afford 175 mg of **12** (0.40 mmol, 80% yield) as a colorless oil. Spectroscopic data for **12** are in accordance with literature.[S33]

^{1}H NMR (400 MHz, $CDCl_3$): δ 7.83 – 7.74 (m, 2H), 7.39 – 7.31 (m, 2H), 4.27 – 4.22 (m, 1H), 4.09 (t, *J* = 5.8 Hz, 2H), 3.43 (dd, *J* = 15.8, 5.0 Hz, 1H), 3.15 (dd, *J* = 15.8, 5.5 Hz, 1H), 2.45 (s, 3H), 2.20 – 2.06 (m, 1H), 2.01 – 1.79 (m, 3H).

^{13}C{^{1}H} NMR (101 MHz, $CDCl_3$): δ 145.4, 133.4, 130.4, 128.3, 97.2, 69.7, 62.9, 48.2, 35.8, 27.4, 22.1.

4-Bromo-6-cyanohexyl 4-methylbenzenesulfonate (13)

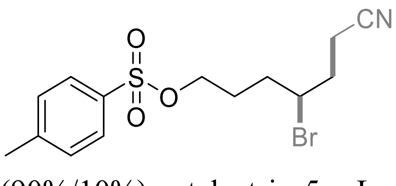

Following **GP-*b***, starting from 120 mg (0.5 mmol, 0.1M) of pent-4-en-1-yl-4-methylbenzenesulfonate (**1b**), 71 µL (1 mmol, 2.0 equiv.) of bromoacetonitrile (**2c**) and 25 mg of g-C_3N_4/$Cs_2AgBiCl_6$ (90%/10%) catalyst in 5 mL of acetonitrile:water (95:5) mixture. The crude mixture was purified

using silica gel chromatography (9:1 cyclohexane/ethyl acetate) to afford 147 mg of **13** (0.41 mmol, 82% yield) as a colorless oil. Spectroscopic data for **13** are in accordance with literature.[S34]

^{1}H NMR (400 MHz, CDCl$_3$): δ 7.81 – 7.74 (m, 2H), 7.39 – 7.31 (m, 2H), 4.10 – 4.03 (m, 2H), 4.00 – 3.94 (m, 1H), 2.65 – 2.49 (m, 2H), 2.45 (s, 3H), 2.19 – 1.70 (m, 6H).

^{13}C{^{1}H} NMR (101 MHz, CDCl$_3$): δ 144.8, 132.7, 129.7, 127.6, 118.3, 69.1, 53.4, 34.5, 34.6, 26.9, 21.4, 15.8.

Diethyl-2-(2-bromo-5-(tosyloxy)pentyl)malonate (14)

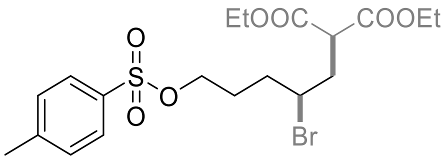

Following **GP- *b***, starting from 120 mg (0.5 mmol, 0.1M) of pent- 4-en-1-yl-4-methylbenzenesulfonate (**1b**), 171 μL (1 mmol, 2.0 equiv.) of diethyl bromomalonate (**2d**) and 25 mg of g-$C_3N_4/Cs_2AgBiCl_6$ (90%/10%) catalyst in 5 mL of acetonitrile:water (95:5) mixture. The crude mixture was purified using silica gel chromatography (9:1 cyclohexane/ethyl acetate) to afford 184 mg of **14** (0.38 mmol, 76% yield) as a colorless oil.

^{1}H NMR (400 MHz, CDCl$_3$): δ 7.75 (d, *J* = 8.4 Hz, 2H), 7.33 (d, *J* = 8.1 Hz, 2H), 4.27 – 4.09 (m, 4H), 4.08-3.97 (m, 2H), 3.96 – 3.84 (m, 1H), 3.70 (dd, *J* = 10.1, 4.3 Hz, 1H), 2.42 (s, 3H), 2.43 – 2.31 (m, 1H), 2.24-2.13 (m, 1H), 1.95 – 1.69 (m, 4H), 1.24 (td, *J* = 7.1, 2.9 Hz, 6H).

^{13}C{^{1}H} NMR (101 MHz, CDCl$_3$): δ 168.5, 168.3, 144.6, 132.7, 129.7, 127.6, 69.3, 61.5, 61.4, 53.2, 50.2, 37.5, 35.0, 26.8, 21.4, 13.8, 13.8.

HRMS (EI, m/z): calcd. for $C_{19}H_{28}BrO_7S$ $[M+H]^+$ 479.0734, found 479.0727.

Ethyl 4-bromo-2,2-difluoro-7-(tosyloxy)heptanoate (15)

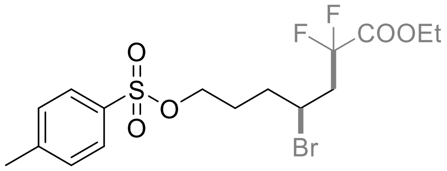

Following **GP-*b***, starting from 120 mg (0.5 mmol, 0.1M) of pent-4-en-1-yl-4-methylbenzenesulfonate (**1b**), 128 μL (1 mmol, 2.0 equiv.) of ethyl 2-bromo-2,2-difluoroacetate (**2e**) and 25 mg of g-$C_3N_4/Cs_2AgBiCl_6$ (90%/10%) catalyst in 5 mL of acetonitrile:water (95:5) mixture. The crude mixture was purified using silica gel chromatography (9:1 cyclohexane/ethyl acetate) to afford 162.00 mg of **15** (0.37 mmol, 74% yield) as a colorless oil.

^{1}H NMR (400 MHz, CDCl$_3$): δ 7.76 (d, *J* = 8.4 Hz, 2H), 7.34 (d, *J* = 8.0 Hz, 2H), 4.32 (q, *J* = 7.1 Hz, 2H), 4.14 – 3.96 (m, 3H), 2.90-2.67 (m, 1H), 2.66-2.49 (m, 1H), 2.43 (s, 3H), 2.05 – 1.84 (m, 2H), 1.85 – 1.71 (m, 2H), 1.34 (t, *J* = 7.2 Hz, 3H).

^{13}C{^{1}H} NMR (101 MHz, CDCl$_3$): δ 163.1 (t, *J* = 32.1 Hz), 144.7, 132.7, 129.7, 127.6, 114.3 (dd, *J* = 253.6, 250.7 Hz), 69.1, 63.1, 44.8 (dd, *J* = 5.7, 3.7 Hz), 43.3 (t, *J* = 23.6 Hz), 34.7, 26.6, 21.4, 13.6.

HRMS (EI, m/z): calcd. for $C_{16}H_{22}BrF_2O_5S$ $[M+H]^+$ 443.0334, found 443.0328.

2-Bromo-4,4,4-trichlorobutyl benzoate (16)

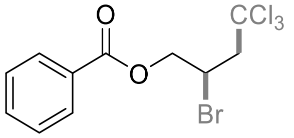

Following **GP-*b***, starting from 81 mg (0.5 mmol, 0.1M) of allyl benzoate (**1c**), 99 µL (1 mmol, 2.0 equiv.) of bromotrichloromethane (**2a**) and 25 mg of g-$C_3N_4/Cs_2AgBiCl_6$ (90%/10%) catalyst in 5 mL of acetonitrile:water (95:5) mixture. The crude mixture was purified using silica gel chromatography (95:5 cyclohexane/ethyl acetate) to afford 143 mg of **16** (0.40 mmol, 80% yield) as a white solid (m.p.: 89.7-90.2 °C). Spectroscopic data for **16** are in accordance with literature.[S8]

^{1}H NMR (400 MHz, CDCl$_3$): δ 8.12 – 8.03 (m, 2H), 7.65 – 7.56 (m, 1H), 7.52 – 7.42 (m, 2H), 4.78 – 4.68 (m, 1H), 4.72 – 4.55 (m, 2H), 3.54 – 3.40 (m, 2H).

^{13}C{^{1}H} NMR (101 MHz, CDCl$_3$): δ 165.6, 133.3, 129.6, 129.1, 128.4, 96.4, 67.0, 59.1, 42.8.

2-Bromo-4-cyanobutyl benzoate (17)

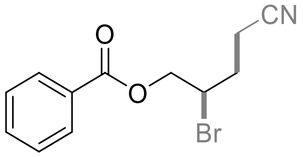

Following **GP- *b***, starting from 81 mg (0.5 mmol, 0.1M) of allyl benzoate (**1c**), 71 µL (1 mmol, 2.0 equiv.) of bromoacetonitrile (**2c**) and 25 mg of g-$C_3N_4/Cs_2AgBiCl_6$ (90%/10%) catalyst in 5 mL of acetonitrile:water (95:5) mixture. The crude mixture was purified using silica gel chromatography (9:1 cyclohexane/ethyl acetate) to afford 108 mg of **17** (0.38 mmol, 76% yield) as colorless oil. Spectroscopic data for **17** are in accordance with literature.[S34]

^{1}H NMR (400 MHz, CDCl$_3$): δ 8.09 – 7.98 (m, 2H), 7.64 – 7.55 (m, 1H), 7.51 – 7.40 (m, 2H), 4.65 (dd, *J* = 11.8, 5.7 Hz, 1H), 4.54 (dd, *J* = 11.8, 6.4 Hz, 1H), 4.43 – 4.27 (m, 1H), 2.76 – 2.57 (m, 2H), 2.40 – 2.29 (m, 1H), 2.18 – 2.05 (m, 1H).

^{13}C{^{1}H} NMR (101 MHz, CDCl$_3$): δ 166.2, 134.0, 130.2, 129.6, 129.0, 118.7, 67.6, 48.8, 31.6, 16.2.

Diethyl 2-(3-(benzoyloxy)-2-bromopropyl)malonate (18)

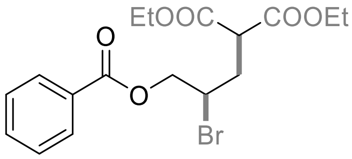

Following **GP-*b***, starting from 81 mg (0.5 mmol, 0.1M) of allyl benzoate (**1c**), 171 µL (1 mmol, 2.0 equiv.) of diethyl bromomalonate (**2d**) and 25 mg of g-$C_3N_4/Cs_2AgBiCl_6$ (90%/10%) catalyst in 5 mL of acetonitrile:water (95:5) mixture. The crude mixture was purified using silica gel chromatography (8:2 cyclohexane/ethyl acetate) to afford 151 mg of **18** (0.38 mmol, 76% yield) as a colorless oil.

^{1}H NMR (400 MHz, $CDCl_3$): δ 8.09 – 7.97 (m, 2H), 7.60 – 7.52 (m, 1H), 7.49 – 7.37 (m, 2H), 4.61 (dd, *J* = 11.8, 5.9 Hz, 1H), 4.51 (dd, *J* = 11.8, 6.0 Hz, 1H), 4.36 – 4.11 (m, 5H), 3.78 (dd, *J* = 10.1, 4.4 Hz, 1H), 2.70 – 2.58 (m, 1H), 2.40 – 2.28 (m, 1H), 1.25 (t, *J* = 7.1 Hz, 6H).

^{13}C{^{1}H} NMR (101 MHz, $CDCl_3$): δ 168.4, 168.2, 165.5, 133.1, 129.5, 129.2, 128.2, 67.6, 61.6, 61.5, 49.8, 48.0, 34.1, 13.8, 13.7.

HRMS (EI, m/z): calcd. for $C_{17}H_{22}BrO_6$ $[M+H]^+$ 401.0594, found 401.0592.

2-Bromo-5-ethoxy-4,4-difluoro-5-oxopentyl benzoate (19)

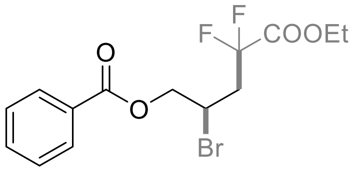

Following **GP-*b***, starting from 81 mg (0.5 mmol, 0.1M) of allyl benzoate (**1c**), 128 µL (1 mmol, 2.0 equiv.) of ethyl 2-bromo-2,2-difluoroacetate (**2e**) and 25 mg of g-$C_3N_4/Cs_2AgBiCl_6$ (90%/10%) catalyst in 5 mL of acetonitrile:water (95:5) mixture. The crude mixture was purified using silica gel chromatography (8:2 cyclohexane/ethyl acetate) to afford 43 mg of **19** (0.12 mmol, 24% yield) as a colorless oil.

^{1}H NMR (400 MHz, $CDCl_3$): δ 8.12 – 7.98 (m, 2H), 7.65 – 7.52 (m, 1H), 7.52 – 7.40 (m, 2H), 4.65 – 4.52 (m, 2H), 4.49 – 4.39 (m, 1H), 4.39 – 4.30 (m, 2H), 2.94 – 2.75 (m, 2H), 1.35 (td, *J* = 7.1, 5.1 Hz, 3H).

^{13}C{^{1}H} NMR (101 MHz, $CDCl_3$): δ 165.5, 163.1 (t, *J* = 32.1 Hz), 133.3, 129.6, 129.1, 128.4, 114.3 (dd, *J* = 253.7, 251.3 Hz), 67.2 (d, *J* = 1.4 Hz), 63.4, 42.4 – 37.6 (m), 13.7.

HRMS (EI, m/z): calcd. for $C_{14}H_{15}BrF_2NaO_4$ $[M+H]^+$ 387.0014, found 387.0018.

2-Bromo-4,4,4-trichloro-2-methylbutyl benzoate (20)

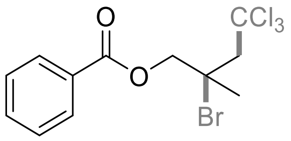

Following **GP-*b***, starting from 88.00 mg (0.5 mmol, 0.1M) of 2-methylallyl benzoate (**1d**), 98.50 µL (1 mmol, 2.0 equiv.) of bromotrichloromethane (**2a**) and 25 mg of g-C_3N_4/$Cs_2AgBiCl_6$ (90%/10%) catalyst in 5 mL of acetonitrile:water (95:5) mixture. The crude mixture was purified using silica gel chromatography (95:5 cyclohexane/ethyl acetate) to afford 154 mg of **20** (0.41 mmol, 82% yield) as a colorless oil. Spectroscopic data for **20** are in accordance with literature.[S8]

^{1}H NMR (400 MHz, $CDCl_3$): δ 8.14 – 8.05 (m, 2H), 7.65 – 7.56 (m, 1H), 7.53 – 7.43 (m, 2H), 4.88 – 4.63 (m, 2H), 3.77 (d, *J* = 15.9 Hz, 1H), 3.63 (d, *J* = 16.0 Hz, 1H), 2.17 (s, 3H).

^{13}C{^{1}H} NMR (101 MHz, $CDCl_3$): δ 165.3, 133.3, 129.6, 129.2, 128.4, 95.2, 71.2, 62.4, 61.1, 28.2.

2-Bromo-4cyano-2-methylbutyl benazoate (21)

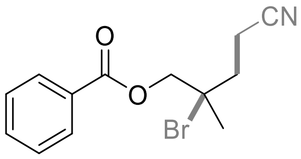

Following **GP- *b***, starting from 88 mg (0.5 mmol, 0.1M) of 2-methylallyl benzoate (**1d**), 71 µL (1 mmol, 2.0 equiv.) of bromoacetonitrile (**2c**) and 25 mg of g-C_3N_4/$Cs_2AgBiCl_6$ (90%/10%) catalyst in 5 mL of acetonitrile:water (95:5) mixture. The crude mixture was purified using silica gel chromatography (9:1 cyclohexane/ethyl acetate) to afford 92 mg of **21** (0.31 mmol, 62% yield) as a colorless oil.

^{1}H NMR (400 MHz, $CDCl_3$): δ 8.08 – 7.99 (m, 2H), 7.64 – 7.56 (m, 1H), 7.52 – 7.40 (m, 2H), 4.50 (d, *J* = 2.3 Hz, 2H), 2.76 – 2.57 (m, 2H), 2.38 – 2.29 (m, 1H), 2.27 – 2.18 (m, 1H), 1.85 (s, 3H).

^{13}C{^{1}H} NMR (101 MHz, $CDCl_3$): δ 165.3, 133.3, 129.5, 129.0, 128.4, 118.7, 70.7, 62.8, 37.2, 28.2, 14.2.

HRMS (EI, m/z): calcd. for $C_{13}H_{15}BrNO_2$ $[M+H]^+$ 296.0281, found 296.0284.

Diethyl 2-(3-(benzoyloxy)-2-bromo-2-methylpropyl)malonate (22)

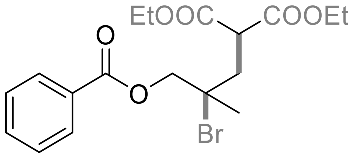

Following **GP-*b***, starting from 88 mg (0.5 mmol, 0.1M) of 2-methylallyl benzoate (**1d**), 171 µL (1 mmol, 2.0 equiv.) of diethyl bromomalonate (**2d**) and 25 mg of g-C_3N_4/$Cs_2AgBiCl_6$ (90%/10%) catalyst in 5 mL of acetonitrile:water (95:5) mixture. The crude mixture was purified using silica gel chromatography (8:2 cyclohexane/ethyl acetate) to afford 188 mg of **22** (0.45 mmol, 90% yield) as a colorless oil.

^{1}**H NMR (400 MHz, CDCl$_3$)**: δ 8.09 – 8.01 (m, 2H), 7.61 – 7.52 (m, 1H), 7.49 – 7.39 (m, 2H), 4.53 – 4.39 (m, 2H), 4.31 – 4.05 (m, 4H), 3.76 (t, *J* = 5.9 Hz, 1H), 2.76 – 2.59 (m, 2H), 1.79 (s, 3H), 1.23 (dt, *J* = 16.1, 7.1 Hz, 6H).

^{13}**C{^{1}H} NMR (101 MHz, CDCl$_3$)**: δ 168.9, 168.8, 165.3, 133.1, 129.5, 129.2, 128.3, 71.3, 63.7, 61.7, 61.6, 49.4, 40.1, 28.6, 13.7, 13.7.

HRMS (EI, m/z): calcd. for $C_{18}H_{24}BrO_6$ $[M+H]^+$ 415.0751, found 415.0751.

3-Bromo-2-(cyanomethyl)-3-methylbutyl benzoate (23)

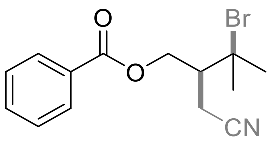

Following **GP-*b***, starting from 95 mg (0.5 mmol,0.1M) of 3-methylbut-2-en-1-yl benzoate (**1e**), 141 µL (2 mmol, 4.0 equiv.) of bromoacetonitrile (**2c**) and 25 mg of g-C_3N_4/$Cs_2AgBiCl_6$ (90%/10%) catalyst in 5 mL of acetonitrile:water (95:5) mixture. The crude mixture was purified using silica gel chromatography (8:2 cyclohexane/ethyl acetate) to afford 132 mg of **23** (0.43 mmol, 86% yield) as a white solid (m.p.: 86.3-87.5 °C).

^{1}**H NMR (400 MHz, CDCl$_3$)**: δ 8.10 – 7.99 (m, 2H), 7.64 – 7.52 (m, 1H), 7.51 – 7.39 (m, 2H), 4.74 (dd, *J* = 11.7, 4.3 Hz, 1H), 4.40 (dd, *J* = 11.8, 7.2 Hz, 1H), 2.89 (dd, *J* = 17.2, 5.0 Hz, 1H), 2.73 (dd, *J* = 17.2, 7.3 Hz, 1H), 2.48 – 2.42 (m, 1H), 1.92 (d, *J* = 11.7 Hz, 6H).

^{13}**C{^{1}H} NMR (101 MHz, CDCl$_3$)**: δ 165.9, 133.2, 129.5, 129.1, 128.4, 118.3, 65.7, 64.3, 47.8, 33.4, 32.0, 18.2.

HRMS (EI, m/z): calcd. for $C_{14}H_{17}BrNO_2$ $[M+H]^+$ 310.0437, found 310.0437.

2-Bromo(phenyl)methyl-3,3,3-trichloropropyl benzoate (24)

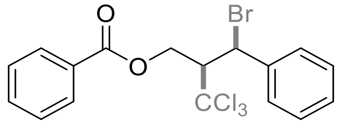

Following **GP-*b***, starting from 119 mg (0.5 mmol, 0.1M) of cynnamil benzoate (**1f**), 99 µL (1 mmol, 2.0 equiv.) of bromotrichloromethane (**2a**) and 25 mg of g-C_3N_4/$Cs_2AgBiCl_6$ (90%/10%) catalyst in 5 mL of acetonitrile:water (95:5) mixture. The crude mixture was purified using silica gel chromatography (95:5 cyclohexane/DCM) to afford 187 mg of **24** (0.43 mmol, 86% yield) as a colorless oil.

^{1}**H NMR (400 MHz, CDCl$_3$)**: δ 7.90 – 7.84 (m, 2H), 7.56 – 7.50 (m, 3H), 7.38 (dd, *J* = 8.4, 7.2 Hz, 2H), 7.33 – 7.21 (m, 3H), 5.86 (d, *J* = 2.5 Hz, 1H), 5.11 (dd, *J* = 12.5, 5.1 Hz, 1H), 5.01 (dd, *J* = 12.5, 4.5 Hz, 1H), 3.49 (td, *J* = 4.9, 2.6 Hz, 1H).

$^{13}C\{^1H\}$ **NMR (101 MHz, CDCl$_3$)**: δ 165.9, 140.7, 133.3, 129.8, 129.5, 128.9, 128.5, 128.4, 128. 0, 100.1, 65.0, 62.7, 51.8.

HRMS (EI, m/z): calcd. for $C_{17}H_{14}BrCl_3NaO_2$ $[M+Na]^+$ 456.9135, found 456.9145.

3-Bromo-2-(cyanomethyl)-3-phenylpropyl benzoate (25)

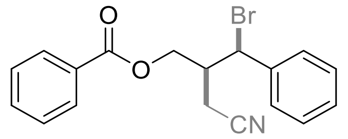

Following **GP-*b***, starting from 119 mg (0.5 mmol, 0.1M) of cynnamil benzoate (**1f**), 71 µL (1 mmol, 2.0 equiv.) of bromoacetonitrile (**2c**) and 25 mg of g-C_3N_4/$Cs_2AgBiCl_6$ (90%/10%) catalyst in 5 mL of acetonitrile:water (95:5) mixture. The crude mixture was purified using silica gel chromatography (9:1 cyclohexane/ethyl acetate) to afford 104 mg of **25** (0.29 mmol, 56% yield; *ca.* 1:1 diastereoisomeric mixture) as a colorless oil.

^1H **NMR (400 MHz, mixture of diastereoisomers, CDCl$_3$)**: δ 8.11 – 7.97 (m, 4H), 7.64 – 7.54 (m, 2H), 7.51 – 7.44 (m, 6H), 7.44 – 7.30 (m, 8H), 5.15 – 5.07 (m, 2H), 4.84 (dd, J = 11.7, 3.7 Hz, 1H), 4.66 (dd, J = 11.7, 6.7 Hz, 1H), 4.36 (dd, J = 11.7, 4.0 Hz, 1H), 4.09 (dd, J = 11.7, 6.3 Hz, 1H), 3.08 – 2.97 (m, 2H), 2.97 – 2.84 (m, 2H), 2.59 (dd, J = 17.0, 4.7 Hz, 1H), 2.34 (dd, J = 17.0, 6.8 Hz, 1H).

$^{13}C\{^1H\}$ **NMR (101 MHz, mixture of diastereoisomers, CDCl$_3$)**: δ 166.0, 165.8, 138.5, 138.5, 133.5, 133.5, 129.8, 129.7, 129.4, 129.3, 129.3, 129.2, 128.6, 128.6, 127.8, 127.6, 117.4, 116.9, 65.0, 63.5, 54.3, 52.8, 43.3, 43.0, 19.9, 18.6.

HRMS (EI, m/z): calcd. for $C_{18}H_{17}BrNO_2$ $[M+H]^+$ 358.0437, found 358.0435.

Reaction between but-2-en-1-yl benzoate (1g) and bromoacetonitrile (2c)

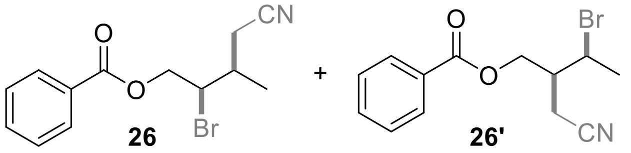

Following **GP-*b***, starting from 88 mg (0.5 mmol, 0.1M) of but-2-en-1-yl benzoate (**1g**), 70.50 µL (1 mmol, 2.0 equiv.) of bromoacetonitrile (**2c**) and 25 mg of g-C_3N_4/$Cs_2AgBiCl_6$ (90%/10%) catalyst in 5 mL of acetonitrile:water (95:5) mixture. The crude mixture was purified using silica gel chromatography (8:2 cyclohexane/ethyl acetate) to afford 98 mg of a *ca.* 1:1 regioisomeric mixture of 2-bromo-4-cyano-3-methylbutyl benzoate **26** and 3-bromo-2-(cyanomethyl)butyl benzoate **26'** (0.33 mmol, 66% overall yield; both regioisomers were obtained as *ca.* 1:1 diastereoisomeric mixtures) as colorless oil.

^{1}H NMR (400 MHz, regio- and diastereo- isomers mixture, $CDCl_3$): δ 8.10 – 7.99 (m, 8H), 7.70 – 7.54 (m, 4H), 7.49 – 7.44 (m, 8H), 4.70 – 4.23 (m, 12H), 2.81 – 2.63 (m, 5H), 2.62 – 2.27 (m, 7H), 1.86 (dd, *J* = 6.9, 6.1 Hz, 6H), 1.31 (d, *J* = 6.8 Hz, 3H), 1.19 (d, *J* = 6.6 Hz, 3H).

$^{13}C\{^1H\}$ NMR (101 MHz, regio- and diastereo- isomers mixture, $CDCl_3$): δ 166.5, 166.4, 166.2, 166.1, 134.0, 134.0, 133.9, 130.2, 130.2, 130.1, 129.7, 129.7, 129.7, 129.6, 129.0, 129.0, 118.4, 118.3, 118.2, 118.0, 66.1, 66.0, 65.1, 64.7, 55.2, 55.0, 49.6, 49.5, 43.4, 43.0, 34.6, 33.6, 24.5, 24.2, 24.1, 22.7, 19.7, 18.4, 18.3, 15.0.

HRMS (EI, m/z): calcd. for $C_{13}H_{15}BrNO_2$ $[M+H]^+$ 296.0281, found 296.0282.

N-(4-Bromo-6,6,6-trichlorohexyl)benzamide (27)

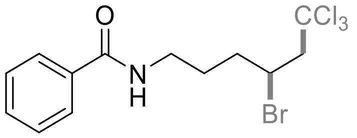

Following **GP-*b***, starting from 95 mg (0.5 mmol, 0.1M) of *N*-(pent-4-en-1-yl)benzamide (**1h**), 99 μL (1 mmol, 2.0 equiv.) of bromotrichloromethane (**2a**) and 25 mg of g-C_3N_4/$Cs_2AgBiCl_6$ (90%/10%) catalyst in 5 mL of acetonitrile:water (95:5) mixture. The crude mixture was purified using silica gel chromatography (8:2 ciclohexane/ethyl acetate) to afford 189 mg of **27** (0.49 mmol, 98% yield) as a colorless dense oil.

Following **GP-*c***, starting from 567 mg (3.0 mmol, 0.1M) of *N*-(pent-4-en-1-yl)benzamide (**1h**), 587 μL (6 mmol, 2.0 equiv.) of bromotrichloromethane (**2a**) and 150 mg of g-C_3N_4/$Cs_2AgBiCl_6$ (90%/10%) catalyst in 30 mL of acetonitrile:water (95:5) mixture. The crude mixture was purified using silica gel chromatography (95:5 cyclohexane/ethyl acetate) to afford 1.15 g of **27** (3.0 mmol, >99% yield).

^{1}H NMR (400 MHz, $CDCl_3$): δ 7.80 – 7.71 (m, 2H), 7.53 – 7.45 (m, 1H), 7.45 – 7.35 (m, 2H), 6.53 (t, *J* = 6.0 Hz, 1H), 4.55 – 4.18 (m, 1H), 3.56 – 3.39 (m, 3H), 3.20 (dd, *J* = 15.8, 5.5 Hz, 1H), 2.20 – 2.07 (m, 1H), 2.06 – 1.91 (m, 2H), 1.95 – 1.73 (m, 1H).

$^{13}C\{^1H\}$ NMR (101 MHz, $CDCl_3$): δ 168.2, 134.9, 131.9, 129.0, 127.3, 97.4, 62.9, 48.9, 39.5, 37.1, 28.1.

HRMS (EI, m/z): calcd. for $C_{13}H_{15}BrCl_3NNaO$ $[M+Na]^+$ 407.9295, found 407.9295.

N-(4-Bromo-6-cyanohexyl)benzamide (28)

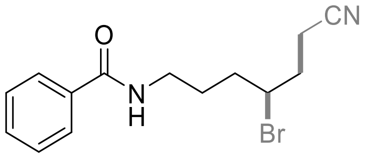

Following **GP-*b***, starting from 95 mg (0.5 mmol, 0.1M) of *N*-(pent-4-en-1-yl)benzamide (**1h**), 71 µL (1 mmol, 2.0 equiv.) of bromoacetonitrile (**2c**) and 25 mg of g-C_3N_4/$Cs_2AgBiCl_6$ (90%/10%) catalyst in 5 mL of acetonitrile:water (95:5) mixture. The crude mixture was purified using silica gel chromatography (8:2 cyclohexane/ethyl acetate) to afford 156 mg of **28** (0.50 mmol, >99% yield) as a colorless dense oil.

^{1}H NMR (400 MHz, CDCl$_3$): δ 7.82 – 7.72 (m, 2H), 7.53 – 7.44 (m, 1H), 7.44 (s, 2H), 6.56 (t, J = 5.9 Hz, 1H), 4.13 – 4.02 (m, 1H), 3.57 – 3.38 (m, J = 6.7 Hz, 2H), 2.67 – 2.49 (m, 2H), 2.21 – 1.98 (m, 2H), 1.98 – 1.80 (m, 3H), 1.80 – 1.69 (m, 1H).

^{13}C{^{1}H} NMR (101 MHz, CDCl$_3$): δ 167.5, 134.2, 131.3, 128.3, 126.7, 118.5, 54.1, 38.8, 35.8, 34.3, 27.6, 15.8.

HRMS (EI, m/z): calcd. for $C_{14}H_{17}BrN_2O$ $[M+H]^+$ 309.0597, found 309.0597.

Diethyl 2-(5-benzamido-2-bromopentyl)malonate (29)

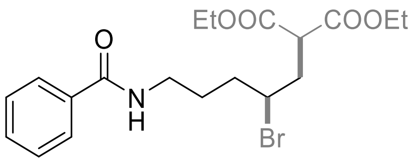

Following **GP-*b***, starting from 95 mg (0.5 mmol, 0.1M) of *N*-(pent-4-en-1-yl)benzamide (**1h**), 171 µL (1 mmol, 2.0 equiv.) of diethyl bromomalonate (**2d**) and 25 mg of g-C_3N_4/$Cs_2AgBiCl_6$ (90%/10%) catalyst in 5 mL of acetonitrile:water (95:5) mixture. The crude mixture was purified using silica gel chromatography (8:2 cyclohexane/ethyl acetate) to afford 203 mg of **29** (0.48 mmol, 96% yield) as a colorless dense oil.

^{1}H NMR (400 MHz, CDCl$_3$): δ 7.81 – 7.72 (m, 2H), 7.52 – 7.41 (m, 1H), 7.44 – 7.35 (m, 2H), 6.50 (t, J = 6.0 Hz, 1H), 4.27 – 4.07 (m, 4H), 4.07 – 3.94 (m, 1H), 3.74 (dd, J = 10.0, 4.4 Hz, 1H), 3.51 – 3.38 (m, 3H), 2.49 – 2.37 (m, 1H), 2.29 – 2.18 (m, 1H), 1.99 – 1.86 (m, 2H), 1.75 (q, J = 6.7 Hz, 1H), 1.24 (td, J = 7.1, 3.1 Hz, 6H).

^{13}C{^{1}H} NMR (101 MHz, CDCl$_3$): δ 168.7, 168.5, 167.4, 134.3, 131.2, 128.3, 126.7, 61.5, 61.5, 53.9, 50.3, 39.0, 37.6, 36.4, 27.5, 13.8, 13.8.

HRMS (EI, m/z): calcd. for $C_{19}H_{26}BrNNaO_5$ $[M+Na]^+$ 450.0887, found 450.0884.

Ethyl 7-benzamido-4-bromo-2,2-difluoroheptanoate (30)

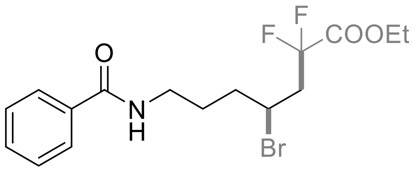

Following **GP-*b***, starting from 95 mg (0.5 mmol, 0.1M) of *N*-(pent-4-en-1-yl)benzamide (**1h**), 128 µL (1 mmol, 2.0 equiv.) of ethyl 2-bromo-2,2-difluoroacetate (**2e**) and 25 mg of g-$C_3N_4/Cs_2AgBiCl_6$ (90%/10%) catalyst in 5 mL of acetonitrile:water (95:5) mixture. The crude mixture was purified using silica gel chromatography (8:2 cyclohexane/ethyl acetate) to afford 104.20 mg of **30** (0.27 mmol, 54% yield) as a colorless dense oil.

^{1}H NMR (400 MHz, $CDCl_3$): δ 7.80 – 7.71 (m, 2H), 7.54 – 7.46 (m, 1H), 7.43 – 7.38 (m, 2H), 6.42 (s, 1H), 4.32 (q, *J* = 7.1 Hz, 2H), 4.26 – 4.13 (m, 1H), 3.49 – 3.38 (m, 2H), 2.89 – 2.76 (m, 1H), 2.71 – 2.58 (m, 1H), 2.08 – 1.83 (m, 3H), 1.79 – 1.70 (m, 1H), 1.34 (t, *J* = 7.2 Hz, 3H).

^{13}C{^{1}H} NMR (101 MHz, $CDCl_3$): δ 168.1 (d, *J* = 221.8 Hz), 163.9 (t, *J* = 32.2 Hz), 135.0, 131.9, 129.0, 127.3, 115.1 (dd, *J* = 275.9, 257.0 Hz), 63.7, 46.1 (dd, *J* = 9.5, 4.7 Hz), 44.1 (t, *J* = 23.5 Hz), 39.5, 36.7, 28.0, 14.3.

HRMS (EI, m/z): calcd. for $C_{16}H_{20}BrF_2NNaO_3$ $[M+Na]^+$ 414.0487, found 414.0486.

***N*-(2-Bromo-4,4,4-trichlorobutyl)benzamide (31)**

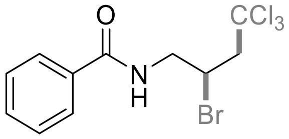

Following **GP-*b***, starting from 81 mg (0.5 mmol, 0.1M) of *N*-allylbenzamide (**1i**), 99 µL (1 mmol, 2.0 equiv.) of bromotrichloromethane (**2a**) and 25 mg of g-$C_3N_4/Cs_2AgBiCl_6$ (90%/10%) catalyst in 5 mL of acetonitrile:water (95:5) mixture. The crude mixture was purified using silica gel chromatography (7:3 cyclohexane/ethyl acetate) to afford 151 mg of **31** (0.42 mmol, 84% yield) as a white solid (m.p.: 125.2-126-4 °C).

^{1}H NMR (400 MHz, $CDCl_3$): δ 7.79 (dd, *J* = 8.4, 1.1 Hz, 2H), 7.51 (t, *J* = 7.4 Hz, 1H), 7.42 (t, *J* = 7.5 Hz, 2H), 6.98 (t, *J* = 6.0 Hz, 1H), 4.57-4.49 (m, 1H), 4.10–4.02 (m, 1H), 3.85–3.76 (m,1H), 3.45 – 3.31 (m, 2H).

^{13}C{^{1}H} NMR (101 MHz, $CDCl_3$): δ 168.0, 133.8, 132.1, 128.8, 127.2, 96.8, 60.2, 47.5, 46.7.

HRMS (EI, m/z): calcd. for $C_{11}H_{12}BrCl_3NO$ $[M+H]^+$ 357.9162, found 357.9166.

Diethyl 2-(3-benzamido-2-bromopropyl)malonate (32)

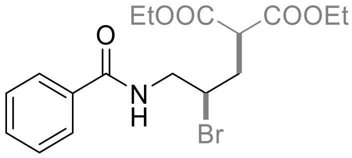

Following **GP-*b***, starting from 81 mg (0.5 mmol, 0.1M) of *N*-allylbenzamide (**1i**), 171 µL (1 mmol, 2.0 equiv.) of diethyl bromomalonate (**2d**) and 25 mg of g-C_3N_4/$Cs_2AgBiCl_6$ (90%/10%) catalyst in 5 mL of acetonitrile:water (95:5) mixture. The crude mixture was purified using silica gel chromatography (8:2 cyclohexane/ethyl acetate) to afford 112.00 mg of **32** (0.28 mmol, 56% yield) as a colorless dense oil.

^{1}H NMR (400 MHz, $CDCl_3$): δ 7.84 – 7.71 (m, 2H), 7.61 – 7.48 (m, 1H), 7.44 (dt, *J* = 6.9, 1.5 Hz, 2H), 6.61 (d, *J* = 6.3 Hz, 1H), 4.34 – 4.13 (m, 5H), 4.01 – 3.62 (m, 3H), 2.58 – 2.32 (m, 2H), 1.28 (td, *J* = 7.1, 1.3 Hz, 6H).

$^{13}C\{^1H\}$ NMR (101 MHz, $CDCl_3$): δ 168.7, 168.3, 167.2, 133.8, 131.6, 128.5, 126.8, 61.8, 61.7, 52.8, 49.9, 45.9, 34.6, 13.9, 13.8.

HRMS (EI, m/z): calcd. for $C_{17}H_{23}BrNO_5$ $[M+H]^+$ 400.0754, found 400.0756.

***N*-(2-Bromo-4,4,4-trichlorobutyl)-1-naphtamide (33)**

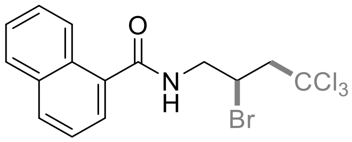

Following **GP-*b***, starting from 106 mg (0.5 mmol, 0.1M) of *N*-allyl 1-naphtamide (**1j**), 99 µL (1 mmol, 2.0 equiv.) of bromotrichloromethane (**2a**) and 25 mg of g-C_3N_4/$Cs_2AgBiCl_6$ (90%/10%) catalyst in 5 mL of acetonitrile:water (95:5) mixture. The crude mixture was purified using silica gel chromatography (8:2 cyclohexane/ethyl acetate) to afford 200 mg of **33** (0.49 mmol, 98% yield) as a pale yellow oil.

^{1}H NMR (400 MHz, $CDCl_3$): δ 8.38 – 8.31 (m, 1H), 7.95 (d, *J* = 8.3 Hz, 1H), 7.89 (dd, *J* = 7.9, 1.6 Hz, 1H), 7.66 (dd, *J* = 7.1, 1.3 Hz, 1H), 7.62 – 7.50 (m, 2H), 7.47 (dd, *J* = 8.3, 7.0 Hz, 1H), 6.52 (s, 1H), 4.73 – 4.50 (m, 1H), 4.25 – 4.12 (m, 1H), 3.97 – 3.83 (m, 1H), 3.46 (dd, *J* = 5.1, 1.5 Hz, 2H).

$^{13}C\{^1H\}$ NMR (101 MHz, $CDCl_3$): δ 169.5, 133.6, 133.3, 131.0, 129.9, 128.2, 127.2, 126.4, 125.1, 125.0, 124.5, 96.5, 60.0, 47.3, 46.5.

HRMS (EI, m/z): calcd. for $C_{15}H_{13}BrCl_3NNaO$ $[M+Na]^+$ 429.9138, found 429.9135.

Diethyl 2-(3-(1-naphtamido)-2-bromoprpyl)malonate (34)

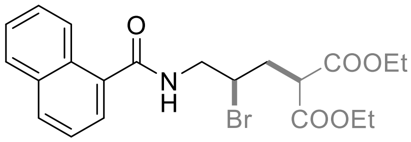

Following **GP-*b***, starting from 106 mg (0.5 mmol, 0.1M) of *N*-allyl 1-naphtamide (**1j**), 171 μL (1 mmol, 2.0 equiv.) of diethyl bromomalonate (**2d**) and 25 mg of g-$C_3N_4/Cs_2AgBiCl_6$ (90%/10%) catalyst in 5 mL of acetonitrile:water (95:5) mixture. The crude mixture was purified using silica gel chromatography (9:1 cyclohexane/DCM) to afford 201 mg of **34** (0.45 mmol, 90% yield) as a colorless dense oil.

^{1}H NMR (400 MHz, CDCl$_3$): δ 8.33 (d, *J* = 8.4 Hz, 1H), 7.93 (d, *J* = 8.3 Hz, 1H), 7.89 – 7.85 (m, 1H), 7.66 (dd, *J* = 7.0, 1.2 Hz, 1H), 7.59 – 7.50 (m, 2H), 7.47 (dd, *J* = 8.3, 7.0 Hz, 1H), 6.48 (t, *J* = 6.1 Hz, 1H), 4.42 – 4.30 (m, 1H), 4.29 – 4.15 (m, 4H), 4.04 – 3.93 (m, 1H), 3.93 – 3.82 (m, 1H), 3.81 (dd, *J* = 9.3, 5.0 Hz, 1H), 2.71 – 2.53 (m, 1H), 2.49-2.32 (m, 1H), 1.29 (td, *J* = 7.1, 2.8 Hz, 6H).

^{13}C{^{1}H} NMR (101 MHz, CDCl$_3$): δ 170.0, 169.3, 168.9, 134.3, 134.2, 131.4, 130.6, 128.8, 127.7, 127.0, 125.8, 125.6, 125.2, 62.4, 62.3, 53.2, 50.6, 46.6, 35.4, 14.5, 14.5.

HRMS (EI, m/z): calcd. for $C_{21}H_{25}BrNO_5$ $[M+H]^+$ 450.0911, found 450.0917.

***N*-(2-Bromo-4,4,4-trichlorobutyl) 4-methylbenzenesulfonamide (35)**

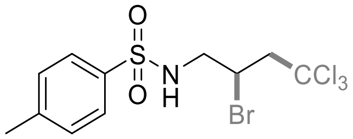

Following **GP-*b***, starting from 106 mg (0.5 mmol, 0.1M) of *N*-allyl 4-methylbenzenesulfonamide (**1k**), 99 μL (1 mmol, 2.0 equiv.) of bromotrichloromethane (**2a**) and 25 mg of g-$C_3N_4/Cs_2AgBiCl_6$ (90%/10%) catalyst in 5 mL of acetonitrile:water (95:5) mixture. The crude mixture was purified using silica gel chromatography (8:2 cyclohexane/ethyl acetate) to afford 198 mg of **35** (0.48 mmol, 96% yield) as a pale yellow dense oil.

^{1}H NMR (400 MHz, CDCl$_3$): δ 7.76 (d, *J* = 8.4 Hz, 2H), 7.32 (d, *J* = 8.1 Hz, 2H), 5.27 (t, *J* = 6.7 Hz, 1H), 4.33-4.22 (m, 1H), 3.57 – 3.47 (m, 1H), 3.44 – 3.35 (m, 1H), 3.29 (dd, *J* = 5.2, 1.6 Hz, 2H), 2.43 (s, 3H).

^{13}C{^{1}H} NMR (101 MHz, CDCl$_3$): δ 144.1, 136.7, 130.0, 127.1, 96.5, 59.4, 49.7, 46.4, 21.6.

HRMS (EI, m/z): calcd. for $C_{11}H_{13}BrCl_3NNaO_2S$ $[M+Na]^+$ 429.8808, found 429.8805.

Diethyl 2-(2-bromo-3-((4-methylphenyl)sulfonamido)propyl) malonate (36)

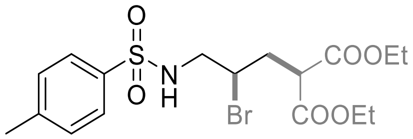

Following **GP-*b***, starting from 106 mg (0.5 mmol, 0.1M) of *N*-allyl-4-methylbenzenesulfonamide (**1k**), 171.00 µL (1 mmol, 2.0 equiv.) of diethyl bromomalonate (**2d**) and 25 mg of g-$C_3N_4/Cs_2AgBiCl_6$ (90%/10%) catalyst in 5 mL of acetonitrile:water (95:5) mixture. The crude mixture was purified using silica gel chromatography (8:2 cyclohexane/ethyl acetate) to afford 166 mg of **36** (0.37 mmol, 74% yield) as a colorless dense oil.

^{1}H NMR (400 MHz, $CDCl_3$): δ 7.74 (d, *J* = 8.4 Hz, 2H), 7.32 (d, *J* = 8.0 Hz, 2H), 4.99 (t, *J* = 6.6 Hz, 1H), 4.27 – 4.12 (m, 4H), 4.11 – 3.90 (m, 1H), 3.65 (dd, *J* = 9.4, 4.9 Hz, 1H), 3.39 – 3.19 (m, 2H), 2.49 – 2.38 (m, 4H), 2.33 – 2.18 (m, 1H), 1.26 (td, *J* = 7.1, 4.3 Hz, 6H).

^{13}C{1H} NMR (101 MHz, $CDCl_3$): δ 168.5, 168.1, 143.6, 136.6, 129.7, 126.9, 61.7, 61.6, 51.6, 49.7, 49.2, 34.4, 21.4, 13.8, 13.8.

HRMS (EI, m/z): calcd. for $C_{17}H_{25}BrNO_6S$ $[M+H]^+$ 450.0580, found 450.0572.

1-(2-Bromo-4,4,4-trichlorobutyl)pyrrolidin-2-one (37)

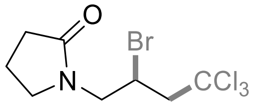

Following **GP-*b***, starting from 63 mg (0.5 mmol, 0.1M) of 1-allyl-pyrrolidin-2-one (**1l**), 99 µL (1 mmol, 2.0 equiv.) of bromotrichloromethane (**2a**) and 25 mg of g-$C_3N_4/Cs_2AgBiCl_6$ (90%/10%) catalyst in 5 mL of acetonitrile:water (95:5) mixture. The crude mixture was purified using silica gel chromatography (6:4 cyclohexane/ethyl acetate) to afford 141 mg of **37** (0.44 mmol, 88% yield) as a white solid (m.p: 112.7-1139. °C).

^{1}H NMR (400 MHz, $CDCl_3$): δ 4.45 – 4.29 (m, 1H), 3.92 (dd, *J* = 14.2, 7.4 Hz, 1H), 3.63 – 3.53 (m, 2H), 3.52 – 3.45 (m, 1H), 3.43 – 3.26 (m, 2H), 2.46 – 2.37 (m, 2H), 2.16 – 1.96 (m, 2H).

^{13}C{^{1}H} NMR (101 MHz, $CDCl_3$): δ 175.7, 97.0, 60.0, 49.6, 47.8, 43.3, 30.4, 18.0.

HRMS (EI, m/z): calcd. for $C_8H_{12}BrCl_3NO$ $[M+H]^+$ 321.9162, found 321.9161.

Diethyl 2-(2-bromo-3-(2-oxopyrrolidin-1-yl)propyl)malonate (38)

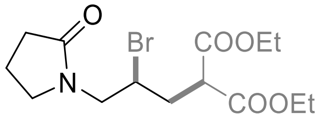

Following **GP-*b***, starting from 63 mg (0.5 mmol, 0.1M) of 1-allyl-pyrrolidin-2-one (**1l**), 171 µL (1 mmol, 2.0 equiv.) of diethyl

bromomalonate (**2d**) and 25 mg of g-C_3N_4/$Cs_2AgBiCl_6$ (90%/10%) catalyst in 5 mL of acetonitrile:water (95:5) mixture. The crude mixture was purified using silica gel chromatography (6:4 cyclohexane/ethyl acetate) to afford 134 mg of **38** (0.37 mmol, 72% yield) as a colorless oil.

^{1}H NMR (400 MHz, $CDCl_3$): δ 4.26 – 4.10 (m, 5H), 3.79 – 3.65 (m, 2H), 3.65 – 3.25 (m, 3H), 2.51 – 2.40 (m, 1H), 2.37 (t, *J* = 8.1 Hz, 2H), 2.25 – 2.09 (m, 1H), 2.04 (dt, *J* = 14.4, 7.6 Hz, 2H), 1.25 (td, *J* = 7.1, 4.4 Hz, 6H).

^{13}C{^{1}H} NMR (101 MHz, $CDCl_3$): δ 175.3, 168.5, 168.3, 61.6, 61.5, 49.9, 49.4, 49.3, 47.7, 34.7, 30.4, 18.0, 13.8, 13.8.

HRMS (EI, m/z): calcd. for $C_{14}H_{23}BrNO_5$ $[M+H]^+$ 364.0754, found 364.0753.

N-(2-Bromo-4,4,4-trichloro-2-methylbutyl)benzamide (39)

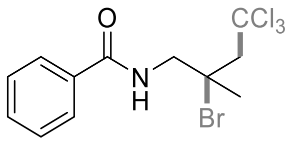

Following **GP-*b***, starting from 88 mg (0.5 mmol, 0.1M) of *N*-(2-methylallyl)benzamide (**1m**), 99 µL (1 mmol, 2.0 equiv.) of bromotrichloromethane (**2a**) and 25 mg of g-C_3N_4/$Cs_2AgBiCl_6$ (90%/10%) catalyst in 5 mL of acetonitrile:water (95:5) mixture. The crude mixture was purified using silica gel chromatography (8:2 cyclohexane/ethyl acetate) to afford 140 mg of **39** (0.38 mmol, 76% yield) as a white solid (m.p.: 94.1-94.7 °C).

^{1}H NMR (400 MHz, $CDCl_3$): δ 7.86 – 7.77 (m, 2H), 7.58 – 7.49 (m, 1H), 7.49 (s, 2H), 6.74 (t, *J* = 6.3 Hz, 1H), 4.13 (dd, *J* = 14.4, 6.7 Hz, 1H), 3.96 (dd, *J* = 14.4, 5.9 Hz, 1H), 3.67 – 3.53 (m, 2H), 2.09 (s, 3H).

^{13}C{^{1}H} NMR (101 MHz, $CDCl_3$): δ 167.3, 133.7, 131.8, 128.6, 126.8, 95.2, 67.4, 63.4, 51.6, 28.7.

HRMS (EI, m/z): calcd. for $C_{12}H_{14}BrCl_3NO$ $[M+H]^+$ 371.9319, found 371.9316.

N-(2-Bromo-4-cyano-2-methylbutyl)benzamide (40)

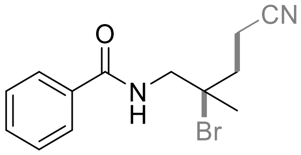

Following **GP-*b***, starting from 87.50 mg (0.5 mmol, 0.1M) of *N*-(2-methylallyl)benzamide (**1m**), 71 µL (1 mmol, 2.0 equiv.) of bromoacetonitrile (**2c**) and 25 mg of g-C_3N_4/$Cs_2AgBiCl_6$ (90%/10%) catalyst in 5 mL of acetonitrile:water (95:5) mixture. The crude mixture was purified using silica gel chromatography (8:2 cyclohexane/ethyl acetate) to afford 135.70 mg of **40** (0.46 mmol, 92% yield) as a colorless dense oil.

^{1}H NMR (400 MHz, CDCl$_3$): δ 7.83 – 7.75 (m, 2H), 7.58 – 7.49 (m, 1H), 7.48 – 7.39 (m, 2H), 6.76 (t, *J* = 6.5 Hz, 1H), 3.79 (qd, *J* = 14.4, 6.4 Hz, 2H), 2.75 – 2.54 (m, 2H), 2.33 – 2.23 (m, 1H), 2.17 – 2.08 (m, 1H), 1.76 (s, 3H).

^{13}C{^{1}H} NMR (101 MHz, CDCl$_3$): δ 168.1, 134.1, 132.4, 129.1, 127.4, 119.5, 69.7, 51.2, 38.3, 29.0, 14.7.

HRMS (EI, m/z): calcd. for $C_{13}H_{16}BrN_2O$ $[M+H]^+$ 295.0441, found 295.0442.

Diethyl 2-(3-benzamido-2-bromo-2-methylpropyl)malonate (41)

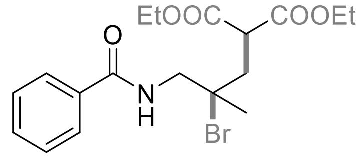

Following **GP-*b***, starting from 88 mg (0.5 mmol, 0.1M) of *N*-(2-methylallyl)benzamide (**1m**), 171 µL (1 mmol, 2.0 equiv.) of diethyl bromomalonate (**2d**) and 25 mg of g-C_3N_4/$Cs_2AgBiCl_6$ (90%/10%) catalyst in 5 mL of acetonitrile:water (95:5) mixture. The crude mixture was purified using silica gel chromatography (8:2 cyclohexane/ethyl acetate) to afford 187 mg of **41** (0.45 mmol, 90% yield) as a colorless dense oil.

^{1}H NMR (400 MHz, CDCl$_3$): δ 7.88 – 7.75 (m, 2H), 7.56 – 7.47 (m, 1H), 7.47 – 7.39 (m, 2H), 6.93 (t, *J* = 6.4 Hz, 1H), 4.30 – 4.13 (m, 4H), 3.96 (dd, *J* = 14.4, 7.1 Hz, 1H), 3.79 (dd, *J* = 6.6, 5.0 Hz, 1H), 3.56 (dd, *J* = 14.4, 5.8 Hz, 1H), 2.66 (dd, *J* = 15.5, 6.7 Hz, 1H), 2.46 (dd, *J* = 15.5, 5.0 Hz, 1H), 1.74 (s, 3H), 1.26 (td, *J* = 7.1, 3.5 Hz, 6H).

^{13}C{^{1}H} NMR (101 MHz, CDCl$_3$): δ 170.3, 169.5, 167.7, 134.4, 132.2, 129.1, 127.3, 69.9, 62.6, 62.4, 50.3, 41.0, 29.9, 14.4, 14.4.

HRMS (EI, m/z): calcd. for $C_{18}H_{25}BrNO_5$ $[M+H]^+$ 414.0911, found 414.0913.

4-Bromo-4-methyl-3-((2-oxopyrrolidin-1-yl)methyl)pentanenitrile (42)

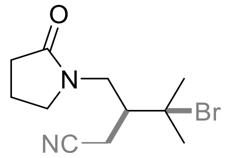

Following **GP-*b***, starting from 77 mg (0.5 mmol, 0.1M) of 1-(3-methylbut-2-en-1-yl)pyrrolidin-2-one (**1n**), 141 µL (2 mmol, 4.0 equiv.) of bromoacetonitrile (**2c**) and 25 mg of g-C_3N_4/$Cs_2AgBiCl_6$ (90%/10%) catalyst in 5 mL of acetonitrile:water (95:5) mixture. The crude mixture was purified using silica gel chromatography (6:4 cyclohexane/ethyl acetate) to afford 77 mg of **42** (0.28 mmol, 56% yield) as a orange dense oil.

^{1}H NMR (400 MHz, $CDCl_3$): δ 3.74 (dd, J = 13.8, 10.5 Hz, 1H), 3.63 – 3.56 (m, 1H), 3.42 – 3.26 (m, 2H), 2.72 (dd, J = 17.3, 4.1 Hz, 1H), 2.62 – 2.22 (m, 4H), 2.22 – 1.94 (m, 2H), 1.83 (d, J = 10.9 Hz, 6H).

^{13}C{^{1}H} NMR (101 MHz, $CDCl_3$): δ 176.2, 118.9, 66.5, 47.7, 47.1, 44.5, 32.6, 31.4, 30.5, 18.3, 17.6.

HRMS (EI, m/z): calcd. for $C_{11}H_{18}BrN_2O$ $[M+H]^+$ 273.0597, found 273.0599.

(2-Bromo-4,4,4-trichlorobutyl)benzene (43)

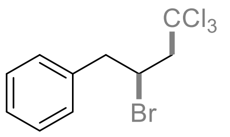

Following **GP-*b***, starting from 66 μL (0.5 mmol, 0.1M) of allyl benzene (**1o**), 99 μL (2 mmol, 4.0 equiv.) of bromotrichloromethane (**2a**) and 25 mg of g-$C_3N_4/Cs_2AgBiCl_6$ (90%/10%) catalyst in 5 mL of acetonitrile:water (95:5) mixture. The crude mixture was purified using silica gel chromatography (95:5 cyclohexane/ethyl acetate) to afford 147 mg of **43** (0.46 mmol, 92% yield) as a colorless oil. Spectroscopic data for **43** are in accordance with literature.[S35]

^{1}H NMR (400 MHz, $CDCl_3$): δ 7.42 – 7.21 (m, 5H), 4.49 (dtd, J = 8.6, 5.6, 4.7 Hz, 1H), 3.52 – 3.38 (m, 2H), 3.36 – 3.17 (m, 2H).

^{13}C{^{1}H} NMR (101 MHz, $CDCl_3$): δ 137.5, 129.5, 128.8, 127.4, 97.2, 61.7, 48.5, 46.1.

(3-Bromo-5,5,5-trichloropentyl)benzene (44)

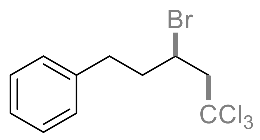

Following **GP-*b***, starting from 75 μL (0.5 mmol, 0.1M) of but-3-en-1-ylbenzene (**1p**), 99 μL (2 mmol, 4.0 equiv.) of bromotrichloromethane (**2a**) and 25 mg of g-$C_3N_4/Cs_2AgBiCl_6$ (90%/10%) catalyst in 5 mL of acetonitrile:water (95:5) mixture. The crude mixture was purified using silica gel chromatography (95:5 cyclohexane/ethyl acetate) to afford 117 mg of **44** (0.35 mmol, 70% yield) as a colorless oil. Spectroscopic data for **44** are in accordance with literature.[S36]

^{1}H NMR (400 MHz, $CDCl_3$): δ 7.40 – 7.28 (m, 2H), 7.28 – 7.20 (m, 3H), 4.33 (dtd, J = 9.3, 5.4, 3.9 Hz, 1H), 3.53 (dd, J = 15.8, 4.9 Hz, 1H), 3.28 (dd, J = 15.8, 5.7 Hz, 1H), 3.01 – 2.96 (m, 1H), 2.86 – 2.79 (m, 1H), 2.46 – 2.39 (m, 1H), 2.27 (dtd, J = 14.4, 9.4, 4.8 Hz, 1H).

^{13}C{^{1}H} NMR (101 MHz, $CDCl_3$): δ 140.6, 129.0, 129.0, 126.8, 97.5, 63.1, 48.9, 41.4, 34.0.

trans-1-Bromo-2-(trichloromethyl)-2,3-dihydro-1_H_-indene (45)

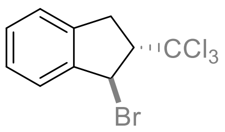

Following **GP-_b_**, starting from 58 µL (0.5 mmol, 0.1M) of indene (**1q**), 197 µL (2 mmol, 4.0 equiv.) of bromotrichloromethane (**2a**) and 25 mg of g-C_3N_4/$Cs_2AgBiCl_6$ (90%/10%) catalyst in 5 mL of acetonitrile:water (95:5) mixture The crude mixture was purified using silica gel chromatography (95:5 cyclohexane/DCM) to afford 99 mg of **45** (0.31 mmol, 62% yield) as an orange oil. Spectroscopic data for **45** are in accordance with literature.[S37]

^{1}H NMR (400 MHz, CD_3COCD_3): δ 7.55 – 7.42 (m, 1H), 7.42 – 7.11 (m, 3H), 5.86 (d, J = 3.3 Hz, 1H), 4.16 – 4.12 (m, 1H), 3.72 (dd, J = 17.7, 9.4 Hz, 1H), 3.37 – 3.15 (m, 1H).

^{13}C{^{1}H} NMR (101 MHz, CD_3COCD_3): δ 142.7, 141.5, 130.3, 128.7, 126.8, 125.4, 102.5, 70.2, 54.2, 36.4.

(_Z_)-2-Bromo-4,4,4-trichlorobut-2-en-1-yl benzoate (46)

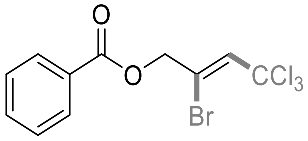

Following **GP-_b_**, starting from 80 mg (0.5 mmol, 0.1M) of prop-2-yn-1-yl benzoate (**1u**), 197 µL (2 mmol, 4.0 equiv.) of bromotrichloromethane (**2a**) and 25 mg of g-C_3N_4/$Cs_2AgBiCl_6$ (90%/10%) catalyst in 5 mL of acetonitrile:water (95:5) mixture. The crude mixture was purified using silica gel chromatography (95:5 cyclohexane/ethyl acetate) to afford 104 mg of **46** (0.29 mmol, 58% yield) as a colorless oil. The stereochemistry of product **46** has been assigned through ^{1}H-NOESY experiments, in analogy with product **47**.

^{1}H NMR (400 MHz, $CDCl_3$): δ 8.07 (dd, J = 8.4, 1.4 Hz, 2H), 7.67 – 7.55 (m, 1H), 7.47 (t, J = 7.7 Hz, 2H), 7.12 (t, J = 1.2 Hz, 1H), 5.04 (d, J = 1.1 Hz, 2H).

^{13}C{^{1}H} NMR (101 MHz, $CDCl_3$): δ 165.4, 133.3, 129.7, 129.1, 128.4, 126.3, 111.7, 67.8.

HRMS (EI, m/z): calcd. for $C_{11}H_9BrCl_3O_2$ $[M+H]^+$ 356.8846, found 356.8844.

(_Z_)-2-Bromo-4cyanobut-2-en-1-yl benzoate (47)

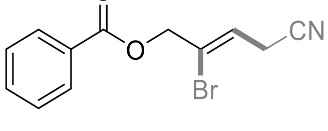

Following **GP-_b_**, starting from 80 mg (0.5 mmol, 0.1M) of prop-2-yn-1-yl benzoate (**1u**), 197 µL (2 mmol, 4.0 equiv.) of bromoacetonitrile (**2c**) and 25 mg of g-C_3N_4/$Cs_2AgBiCl_6$ (90%/10%) catalyst in 5 mL of acetonitrile:water (95:5) mixture. The crude mixture was purified using silica gel chromatography

(85:15 cyclohexane/ethyl acetate) to afford 65 mg of **47** (0.23 mmol, 46% yield) as a colorless dense oil. The stereochemistry of product **47** has been assigned through ^{1}H-NOESY experiments (see below).

^{1}H NMR (400 MHz, CD_3COCD_3): δ 8.13 – 8.00 (m, 2H), 7.73 – 7.60 (m, 1H), 7.60 – 7.47 (m, 2H), 6.50 (tt, *J* = 6.8, 1.2 Hz, 1H), 5.11 (d, *J* = 1.0 Hz, 2H), 3.49 (d, *J* = 6.9 Hz, 2H).

^{13}C{^{1}H} NMR (101 MHz, CD_3COCD_3): δ 166.0, 134.5, 130.6, 130.5, 129.7, 127.0, 123.5, 117.3, 68.8, 20.0.

HRMS (EI, m/z): calcd. for $C_{12}H_{10}BrNNaO_2$ [M+Na]$^+$ 301.9787, found 301.9786.

3-Bromo-5,5,5-trichloropent-3-en-1-yl benzoate (48)

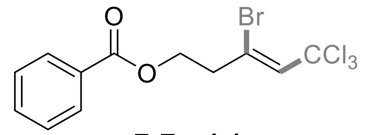

***Z*:*E* = 4:1**

Following **GP-*b***, starting from 87 mg (0.5 mmol, 0.1M) of but-3-yn-1-yl benzoate (**1v**), 197 μL (2 mmol, 4.0 equiv.) of bromotrichloromethane (**2a**) and 25 mg of g-C_3N_4/$Cs_2AgBiCl_6$ (90%/10%) catalyst in 5 mL of acetonitrile:water (95:5) mixture. The crude mixture was purified using silica gel chromatography (95:5 cyclohexane/ethyl acetate) to afford 119 mg of **48** (0.32 mmol, 64% yield; 4:1 *Z*/*E* stereoisomeric mixture) as a colorless oil. The *Z*/*E* stereochemistry of product **48** has been assigned through ^{1}H-NOESY experiments, in analogy with product **49**.

Following **GP-*c***, starting from 519 mg (3.0 mmol, 0.1M) of but-3-yn-1-yl benzoate (**1v**), 1.18 mL (6 mmol, 2.0 equiv.) of bromotrichloromethane (**2a**) and 150 mg of g-C_3N_4/$Cs_2AgBiCl_6$ (90%/10%) catalyst in 30 mL of acetonitrile:water (95:5) mixture. The crude mixture was purified using silica gel chromatography (95:5 cyclohexane/ethyl acetate) to afford 631 mg of **48** (1.70 mmol, 57% yield) as a colorless oil.

^{1}H NMR (400 MHz, isomer *Z*, major, from the mixture, CD_3COCD_3): δ 8.08 – 7.95 (m, 2H), 7.68 – 7.62 (m, 1H), 7.58 – 7.47 (m, 2H), 7.23 (t, *J* = 1.1 Hz, 1H), 4.69 (t, *J* = 6.3 Hz, 2H), 3.49 (td, *J* = 6.3, 1.1 Hz, 2H).

^{13}C{^{1}H} NMR (101 MHz, isomer *Z*, major, from the mixture, CD_3COCD_3): δ 166.2, 139.9, 134.7, 133.7, 130.5, 129.9, 129.1, 91.2, 62.4, 36.1.

^{1}H NMR (400 MHz, isomer *E*, minor, from the mixture, CD_3COCD_3): δ 8.10 – 7.99 (m, 2H), 7.71 – 7.60 (m, 1H), 7.58 – 7.47 (m, 2H), 6.88 (s, 1H), 4.64 – 4.49 (m, 2H), 3.22 – 3.12 (m, 2H).

$^{13}C\{^1H\}$ NMR (101 MHz, isomer *E*, minor, from the mixture, CD_3COCD_3): δ 166.2, 139.9, 134.7, 133.7, 130.5, 129.9, 129.0, 105.8, 61.7, 36.9.

HRMS (EI, m/z): calcd. for $C_{12}H_{11}BrCl_3O_2$ $[M+H]^+$ 370.9003, found 370.9012.

3-Bromo-5-cyanopent-3-en-1yl benzoate (49)

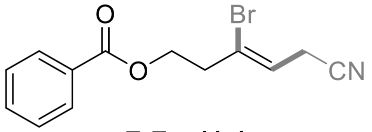

***Z:E* = 11:1**

Following **GP-*b***, starting from 87 mg (0.5 mmol, 0.1M) of but-3-yn-1-yl benzoate (**1v**), 141 µL (2 mmol, 4.0 equiv.) of bromoacetonitrile (**2c**) and 25 mg of g-C_3N_4/$Cs_2AgBiCl_6$ (90%/10%) catalyst in 5 mL of acetonitrile:water (95:5) mixture. The crude mixture was purified using silica gel chromatography (9:1 cyclohexane/ethyl acetate) to afford 73 mg of **49** (0.25 mmol, 50% yield; 11:1 *Z*/*E* stereoisomeric mixture) as a colorless oil. The *Z*/*E* stereochemistry of product **49** has been assigned through ^{1}H-NOESY experiments (see below).

^1H NMR (400 MHz, isomer *Z*, major, from the mixture, CD_3COCD_3): δ 8.02 (dd, *J* = 8.4, 1.5 Hz, 2H), 7.69 – 7.60 (m, 1H), 7.56 – 7.46 (m, 2H), 6.20 (tt, *J* = 6.9, 1.1 Hz, 1H), 4.52 (t, *J* = 6.1 Hz, 2H), 3.42 (dt, *J* = 6.8, 0.9 Hz, 2H), 3.05 (td, *J* = 6.1, 1.0 Hz, 2H).

$^{13}C\{^1H\}$ NMR (101 MHz, isomer *Z*, major, from the mixture, CD_3COCD_3): δ 166.7, 134.2, 131.3, 130.4, 130.1, 129.6, 122.0, 117.8, 62.9, 41.3, 20.5.

^1H NMR (400 MHz, isomer *E*, minor, from the mixture, CD_3COCD_3): δ 8.08 – 7.99 (m, 2H), 7.69 – 7.60 (m, 1H), 7.56 – 7.46 (m, 2H), 6.20 (tt, *J* = 6.9, 1.1 Hz, 1H), 4.37 (t, *J* = 6.5 Hz, 2H), 3.48 (d, *J* = 7.5 Hz, 2H), 3.10 (t, *J* = 6.2 Hz, 2H).

$^{13}C\{^1H\}$ NMR (101 MHz, isomer *E*, minor, from the mixture, CD_3COCD_3): δ 166.7, 134.1, 131.8, 130.5, 130.4, 124.6, 122.5, 117.9, 64.6, 35.8, 18.2.

HRMS (EI, m/z): calcd. for $C_{13}H_{13}BrNO_2$ $[M+H]^+$ 294.0120, found 294.0124.

(*Z*)-3-bromo-2-(tricholoromethyl)but-2-en-1-yl benzoate (50)

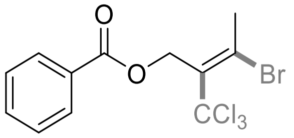

Following **GP-*b***, starting from 87 mg (0.5 mmol, 0.1M) of but-2-yn-1-yl benzoate (**1w**), 197 µL (2 mmol, 4.0 equiv.) of bromotrichloromethane (**2a**) and 25 mg of g-C_3N_4/$Cs_2AgBiCl_6$ (90%/10%) catalyst in 5 mL of acetonitrile:water (95:5) mixture. The crude mixture was purified using silica gel chromatography (95:5 cyclohexane/DCM) to afford 90 mg of **50** (0.24 mmol, 48% yield) as a colorless oil. The

stereochemistry of product **50** has been assigned through ^{1}H-NOESY experiments, in analogy with product **51**.

^{1}H NMR (400 MHz, CD_3COCD_3): δ 8.07 (dd, *J* = 8.3, 1.4 Hz, 2H), 7.73 – 7.59 (m, 1H), 7.55 (dd, *J* = 8.3, 7.1 Hz, 2H), 5.28 (d, *J* = 1.1 Hz, 2H), 2.53 (t, *J* = 1.0 Hz, 3H).

$^{13}C\{^1H\}$ NMR (101 MHz, CD_3COCD_3): δ 166.4, 134.7, 131.0, 130.8, 130.0, 121.9, 116.6, 69.3, 29.5.

HRMS (EI, m/z): calcd. for $C_{12}H_{11}BrCl_3O_2$ $[M+H]^+$ 370.9003, found 370.9004.

(*E*)-3-Bromo-2-(cyanomethyl)but-2-en-1-yl benzoate (51)

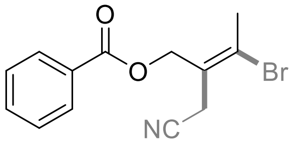

Following **GP-*b***, starting from 87 mg (0.5 mmol, 0.1M) of but-2-yn-1-yl benzoate (**1w**), 141 μL (2 mmol, 4.0 equiv.) of bromoacetonitrile (**2c**) and 25 mg of g-C_3N_4/$Cs_2AgBiCl_6$ (90%/10%) catalyst in 5 mL of acetonitrile:water (95:5) mixture. The crude mixture was purified using silica gel chromatography (9:1 cyclohexane/ethyl acetate) to afford 51 mg of **51** (0.17 mmol, 34% yield) as a colorless oil. The stereochemistry of product **51** has been assigned through ^{1}H-NOESY experiments (see below).

^{1}H NMR (400 MHz, CD_3COCD_3): δ 8.12 – 8.04 (m, 2H), 7.69 – 7.63 (m, 1H), 7.59 – 7.48 (m, 2H), 5.06 (d, *J* = 0.7 Hz, 2H), 3.67 (q, *J* = 1.0 Hz, 2H), 2.60 (t, *J* = 0.9 Hz, 3H).

$^{13}C\{^1H\}$ NMR (101 MHz, CD_3COCD_3): δ 166.1, 133.8, 130.3, 130.1, 129.5, 129.1, 125.5, 117.0, 62.9, 25.5, 24.0.

HRMS (EI, m/z): calcd. for $C_{13}H_{12}BrNNaO_2$ $[M+Na]^+$ 315.9944, found 315.9944.

3-Bromo-2-(cyanomethyl)-3-phenylallyl benzoate (52)

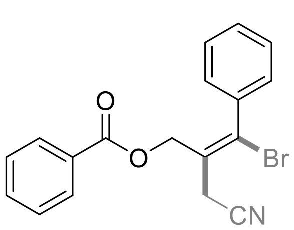

***E:Z* = 2:1**

Following **GP-*b***, starting from 118 mg (0.5 mmol, 0.1M) of 3-phenylprop-2-yn-1-yl benzoate (**1x**), 141 μL (2 mmol, 4.0 equiv.) of bromoacetonitrile (**2c**) and 25 mg of g-C_3N_4/$Cs_2AgBiCl_6$ (90%/10%) catalyst in 5 mL of acetonitrile:water (95:5) mixture. The crude mixture was purified using silica gel chromatography (9:1 cyclohexane/ethyl acetate) to afford 64 mg of **52** (0.18 mmol, 36% yield; 2:1 *E*/*Z* stereoisomeric mixture) as a colorless oil. The *E*/*Z* stereochemistry of product **52** has been assigned through ^{1}H-NOESY experiments (see below).

^{1}H NMR (400 MHz, isomer *E*, major, from the mixture, CD_3COCD_3): 8.11 – 8.04 (m, 2H), 7.72 – 7.62 (m, 1H), 7.58 – 7.49 (m, 2H), 7.48 – 7.43 (m, 5H), 4.88 (s, 2H), 3.87 (s, 2H).

^{13}C{^{1}H} NMR (101 MHz, isomer *E*, major, from the mixture, CD_3COCD_3): δ 166.0, 139.2, 134.0, 130.4, 130.3, 129.7, 129.4, 129.3, 129.2, 129.1, 127.5, 117.0, 64.5, 24.0.

^{1}H NMR (400 MHz, isomer *Z*, minor, from the mixture, CD_3COCD_3): δ 8.20 – 8.13 (m, 2H), 7.72 – 7.62 (m, 1H), 7.58 – 7.49 (m, 2H), 7.48 – 7.43 (m, 5H), 5.33 (s, 2H), 3.46 (s, 2H).

^{13}C{^{1}H} NMR (101 MHz, isomer *Z*, minor, from the mixture, CD_3COCD_3): δ 166.2, 139.4, 134.1, 130.4, 130.3, 130.2, 130.0, 129.4, 129.3, 128.1, 126.9, 117.3, 67.2, 20.7.

HRMS (EI, m/z): calcd. for $C_{18}H_{14}BrNNaO_2$ $[M+Na]^+$ 378.0100, found 378.0096.

***N*-(2-Bromo-4cyanobut-2-en-1-yl)benzamide (53)**

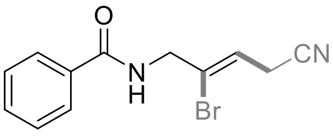

***Z:E* = 10:1**

Following **GP-*b***, starting from 78 mg (0.5 mmol, 0.1M) of *N*-(prop-2-yn-1-yl)benzamide (**1y**), 141 µL (2 mmol, 4.0 equiv.) of bromoacetonitrile (**2c**) and 25 mg of g-C_3N_4/$Cs_2AgBiCl_6$ (90%/10%) catalyst in 5 mL of acetonitrile:water (95:5) mixture. The crude mixture was purified using silica gel chromatography (8:2 cyclohexane/ethyl acetate) to afford 86 mg of **53** (0.31 mmol, 62% yield; 10:1 *Z*/*E* stereoisomeric mixture) as a colorless dense oil. The *Z*/*E* stereochemistry of product **53** has been assigned through ^{1}H-NOESY experiments, in analogy with product **47**.

^{1}H NMR (400 MHz, isomer *Z*, major, from the mixture, CD_3COCD_3): δ 8.26 (bs, 1H), 7.94 (tt, *J* = 7.9, 1.4 Hz, 2H), 7.67 – 7.30 (m, 3H), 6.21 (tt, *J* = 6.9, 1.5 Hz, 1H), 4.37 (dq, *J* = 6.0, 1.3 Hz, 2H), 3.42 (dt, *J* = 6.9, 1.2 Hz, 2H).

^{13}C{^{1}H} NMR (101 MHz, isomer *Z*, major, from the mixture, CD_3COCD_3): δ 166.7, 134.7, 131.8, 130.1, 128.7, 127.6, 119.0, 117.0, 47.5, 19.2.

^{1}H NMR (400 MHz, isomer *E*, minor, from the mixture, CD_3COCD_3): δ 8.26 (bs, 1H), 7.94 (tt, *J* = 7.9, 1.4 Hz, 2H), 7.63 – 7.50 (m, 3H), 7.51 – 7.45 (m, 3H), 6.12 (dd, *J* = 7.8, 6.9 Hz, 1H), 4.45 (d, *J* = 6.5 Hz, 2H), 3.69 (d, *J* = 7.4 Hz, 2H).

^{13}C{^{1}H} NMR (101 MHz, isomer *E*, minor, from the mixture, CD_3COCD_3): δ 167.9, 132.2, 131.6, 128.7, 127.9, 127.5, 124.0, 117.1, 47.4, 21.0.

HRMS (EI, m/z): calcd. for $C_{12}H_{12}BrN_2O$ $[M+H]^+$ 279.0128, found 279.0124.

(Z)-*N*-(3-Bromo-5-cyanopent-3-en-1-yl)benzamide (54)

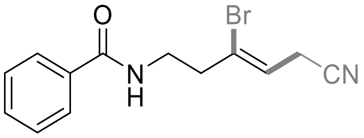

Following **GP-*b***, starting from 87 mg (0.5 mmol, 0.1M) of *N*-(but-3-yn-1-yl)benzamide (**1z**), 141 μL (2 mmol, 4.0 equiv.) of bromoacetonitrile (**2c**) and 25 mg of g-$C_3N_4/Cs_2AgBiCl_6$ (90%/10%) catalyst in 5 mL of acetonitrile:water (95:5) mixture. The crude mixture was purified using silica gel chromatography (7:3 cyclohexane/ethyl acetate) to afford 73 mg of **54** (0.25 mmol, 50% yield) as a colorless dense oil. The stereochemistry of product **54** has been assigned through ^{1}H-NOESY experiments (see below).

^{1}H NMR (400 MHz, CD_3COCD_3): δ 7.93 – 7.82 (m, 3H), 7.56 – 7.49 (m, 1H), 7.49 – 7.40 (m, 2H), 6.06 (tt, *J* = 6.8, 1.1 Hz, 1H), 3.64 (td, *J* = 6.8, 5.8 Hz, 2H), 3.38 (dt, *J* = 6.7, 0.9 Hz, 2H), 2.88 (ddt, *J* = 6.9, 5.9, 1.1 Hz, 2H).

$^{13}C\{^1H\}$ NMR (101 MHz, CD_3COCD_3): δ 167.9, 136.3, 132.3, 131.8, 129.5, 128.4, 121.2, 118.0, 42.2, 39.3, 20.7.

HRMS (EI, m/z): calcd. for $C_{13}H_{14}BrN_2O$ $[M+H]^+$ 293.0284, found 293.0275.

(*Z*)-*N*-(2-Bromo-4,4,4-trichlorobut-2-en-1-yl)-4-methylbenzenesulfonamide (55)

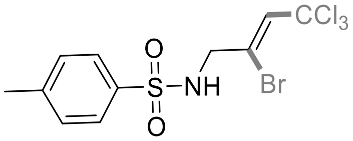

Following **GP-*b***, starting from 105 mg (0.5 mmol, 0.1M) of 4-methyl-*N*-(prop-2-yn-1-yl)benzenesulfonamide (**1aa**), 197 μL (2 mmol, 4.0 equiv.) of bromotrichloromethane (**2a**) and 25 mg of g-$C_3N_4/Cs_2AgBiCl_6$ (90%/10%) catalyst in 5 mL of acetonitrile:water (95:5) mixture. The crude mixture was purified using silica gel chromatography (8:2 cyclohexane/ethyl acetate) to afford 98 mg of **55** (0.24 mmol, 48% yield) as a colorless dense oil. The stereochemistry of product **55** has been assigned through ^{1}H-NOESY experiments, in analogy with product **47**.

^{1}H NMR (400 MHz, CD_3COCD_3): δ 7.87 – 7.75 (m, 2H), 7.43 (t, *J* = 8.3 Hz, 2H), 7.12 (t, *J* = 1.2 Hz, 1H), 7.03 (t, *J* = 6.4 Hz, 1H), 4.35 (dd, *J* = 6.3, 1.2 Hz, 2H), 2.44 (d, *J* = 2.8 Hz, 3H).

$^{13}C\{^1H\}$ NMR (101 MHz, CD_3COCD_3): δ 144.1, 139.8, 138.7, 133.8, 130.3, 127.8, 90.8, 46.2, 21.2.

HRMS (EI, m/z): calcd. for $C_{11}H_{12}BrCl_3NO_2S$ $[M+H]^+$ 405.8832, found 405.8837.

4-(2-Bromo-4,4,4-trichlorobutyl)-2-methoxyphenol (56)

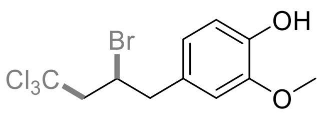

Following **GP-*b***, starting from 82 mg (0.5 mmol, 0.1M) of 4-allyl-2-methoxyphenol (**1ab**), 99 µL (1 mmol, 2.0 equiv.) of bromotrichloromethane (**2a**) and 25 mg of g-$C_3N_4/Cs_2AgBiCl_6$ (90%/10%) catalyst in 5 mL of acetonitrile:water (95:5) mixture. The crude mixture was purified using silica gel chromatography (9:1 cyclohexane/ethyl acetate) to afford 124 mg of **56** (0.34 mmol, 68% yield) as a yellowish oil.

^{1}H NMR (400 MHz, $CDCl_3$): δ 6.92 – 6.85 (m, 1H), 6.78 – 6.71 (m, 2H), 5.61 (s, 1H), 4.48 – 4.43 (m, 1H), 3.90 (s, 3H), 3.42 (dd, *J* = 16.0, 5.6 Hz, 1H), 3.35 – 3.27 (m, 2H), 3.18 (dd, *J* = 14.5, 8.1 Hz, 1H).

$^{13}C\{^1H\}$ NMR (101 MHz, $CDCl_3$): δ 146.9, 145.3, 129.6, 122.7, 114.9, 112.3, 97.6, 61.8, 56.4, 49.1, 46.1.

HRMS (EI, m/z): calcd. for $C_{11}H_{13}BrCl_3O_2$ $[M+H]^+$ 360.9159, found 360.9159.

4-Bromo-5-(4-hydroxy-3-methoxyphenyl)pentanenitrile (57)

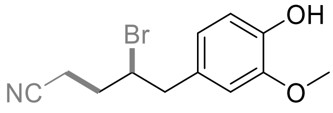

Following **GP-*b***, starting from 82 mg (0.5 mmol, 0.1M) of 4-allyl-2-methoxyphenol (**1ab**), 71 µL (1 mmol, 2.0 equiv.) of bromoacetonitrile (**2c**) and 25 mg of g-$C_3N_4/Cs_2AgBiCl_6$ (90%/10%) catalyst in 5 mL of acetonitrile:water (95:5) mixture. The crude mixture was purified using silica gel chromatography (8:2 cyclohexane/ethyl acetate) to afford 91 mg of **57** (0.32 mmol, 64% yield) as a colorless dense oil.

^{1}H NMR (400 MHz, $CDCl_3$): δ 6.86 (d, *J* = 7.9 Hz, 1H), 6.73 – 6.64 (m, 2H), 5.62 (s, 1H), 4.21 (dtd, *J* = 10.2, 7.2, 3.0 Hz, 1H), 3.89 (s, 3H), 3.22 (dd, *J* = 14.3, 6.9 Hz, 1H), 3.07 (dd, *J* = 14.3, 7.5 Hz, 1H), 2.69 – 2.50 (m, 2H), 2.23 – 2.13 (m, 1H), 2.05 – 1.95 (m, 1H).

$^{13}C\{^1H\}$ NMR (101 MHz, $CDCl_3$): δ 147.0, 145.3, 129.6, 122.4, 119.1, 114.9, 112.0, 56.4, 54.7, 45.6, 33.8, 16.6.

HRMS (EI, m/z): calcd. for $C_{12}H_{15}BrNO_2$ $[M+H]^+$ 284.0281, found 284.0283.

2-Bromo-4,4,4-trichlorobutoxy-1-isopropyl-4-methylcyclohexane (58)

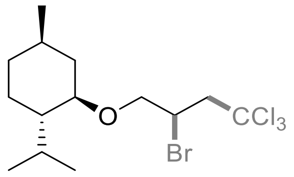

Following **GP-*b***, starting from 98 mg (0.5 mmol, 0.1M) of 2-(allyloxy)-1-isopropyl-4-methylcyclohexane (**1ac**), 99 μL (1 mmol, 2.0 equiv.) of bromotrichloromethane (**2a**) and 25 mg of g-C_3N_4/$Cs_2AgBiCl_6$ (90%/10%) catalyst in 5 mL of acetonitrile:water (95:5) mixture. The crude mixture was purified using silica gel chromatography (95:5 cyclohexane/ethyl acetate) to afford 79 mg of **58** (0.20 mmol, 40% yield; *ca*. 1:1 diastereoisomeric mixture) as a colorless dense oil.

^{1}H NMR (400 MHz, mixture of diastereoisomers, $CDCl_3$): δ 4.35 (ddt, *J* = 10.1, 6.3, 2.8 Hz, 2H), 4.01 (dd, *J* = 10.4, 4.7 Hz, 1H), 3.90 (dd, *J* = 10.4, 6.1 Hz, 1H), 3.66 (dd, *J* = 10.4, 5.7 Hz, 1H), 3.59 – 3.40 (m, 3H), 3.25 (ddd, *J* = 15.9, 6.1, 3.5 Hz, 2H), 3.17 – 3.05 (m, 2H), 2.27 (dtd, *J* = 14.0, 7.0, 2.8 Hz, 2H), 2.08 (ddd, *J* = 9.8, 4.9, 2.8 Hz, 2H), 1.64 (tt, *J* = 9.5, 2.9 Hz, 4H), 1.43 – 1.20 (m, 4H), 1.05 – 0.82 (m, 17H), 0.78 (dd, *J* = 8.9, 7.0 Hz, 6H).

$^{13}C\{^1H\}$ NMR (101 MHz, mixture of diastereoisomers, $CDCl_3$): δ 97.2, 97.1, 80.3, 79.9, 72.2, 71.8, 58.8, 58.8, 48.1, 48.0, 45.5, 45.5, 40.2, 40.0, 34.3, 31.4, 25.4, 25.4, 23.1, 23.1, 22.1, 20.8, 20.8, 16.1 16.0.

HRMS (EI, m/z): calcd. for $C_{14}H_{24}BrCl_3NaO$ $[M+Na]^+$ 414.9968, found 414.9971.

4-Bromo-5-((methylcyclohexyl)oxy)pentanenitrile (59)

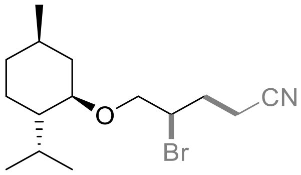

Following **GP-*b***, starting from 98 mg (0.5 mmol, 0.1M) of 2-(allyloxy)-1-isopropyl-4-methylcyclohexane (**1ac**), 71 μL (1 mmol, 2.0 equiv.) of bromoacetonitrile (**2c**) and 25 mg of g-C_3N_4/$Cs_2AgBiCl_6$ (90%/10%) catalyst in 5 mL of acetonitrile:water (95:5) mixture. The crude mixture was purified using silica gel chromatography (9:1 cyclohexane/ethyl acetate) to afford 52.80 mg of **59** (0.17 mmol, 34% yield; *ca*. 1:1 diastereoisomeric mixture) as a colorless dense oil.

^{1}H NMR (400 MHz, mixture of diastereoisomers, $CDCl_3$): δ 4.19 – 4.01 (m, 2H), 3.92 (dd, *J* = 10.1, 4.5 Hz, 1H), 3.77 (dd, *J* = 10.2, 7.5 Hz, 1H), 3.64 (dd, *J* = 10.2, 5.1 Hz, 1H), 3.49 (dd, *J* = 10.1, 7.6 Hz, 1H), 3.10 (td, *J* = 10.6, 4.2 Hz, 2H), 2.68 – 2.49 (m, 4H), 2.46 – 2.29 (m, 2H), 2.18 (qd, *J* = 7.0, 2.8 Hz, 2H), 2.10 – 2.01 (m, 4H), 1.67 – 1.59 (m, 4H), 1.39 – 1.28 (m, 2H), 1.27 – 1.19 (m, 3H), 0.96 – 0.84 (m, 17H), 0.78 (dd, *J* = 7.0, 1.9 Hz, 6H).

$^{13}C\{^1H\}$ NMR (101 MHz, mixture of diastereoisomers, $CDCl_3$): δ 119.2, 119.2, 80.7, 80.4, 72.5, 72.3, 51.2, 51.1, 48.7, 48.6, 40.9, 40.7, 34.9, 32.0, 32.0, 31.8, 31.7, 26.1, 26.1, 23.8, 23.7, 22.7, 21.4, 21.4, 16.7, 16.6, 16.2, 16.2.

HRMS (EI, m/z): calcd. for $C_{15}H_{26}BrNNaO$ $[M+Na]^+$ 338.1090, found 338.1089.

7-Bromo-6-(cyanomethyl)-3,7-dimethyloctyl benzoate (60)

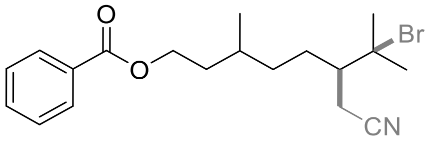

Following **GP-*b***, starting from 130.00 mg (0.5 mmol, 0.1M) of 3,7-dimethyloct-6-en-1-yl benzoate (**1ad**), 141 μL (2 mmol, 4.0 equiv.) of bromoacetonitrile (**2c**) and 25 mg of g-$C_3N_4/Cs_2AgBiCl_6$ (90%/10%) catalyst in 5 mL of acetonitrile:water (95:5) mixture. The crude mixture was purified using silica gel chromatography (95:5 cyclohexane/ethyl acetate) to afford 97 mg of **60** (0.26 mmol, 51% yield; *ca.* 1:1 diastereoisomeric mixture) as a colorless dense oil.

^1H NMR (400 MHz, mixture of diastereoisomers, $CDCl_3$): δ 8.07 – 8.01 (m, 4H), 7.60 – 7.51 (m, 2H), 7.44 (t, *J* = 7.7 Hz, 4H), 4.46 – 4.30 (m, 4H), 2.74 – 2.53 (m, 4H), 1.99 – 1.75 (m, 14H), 1.72 – 1.56 (m, 8H), 1.54 – 1.22 (m, 6H), 1.08 – 0.92 (m, 6H).

$^{13}C\{^1H\}$ NMR (101 MHz, mixture of diastereoisomers, $CDCl_3$): δ 166.5, 132.7, 130.2, 129.4, 128.2, 118.8, 118.8, 70.4, 70.4, 63.0, 63.0, 49.0, 48.8, 35.6, 35.4, 35.2, 35.0, 33.0, 33.0, 31.3, 31.2, 30.1, 30.0, 28.2, 19.7, 19.6, 19.4, 19.1.

HRMS (EI, m/z): calcd. for $C_{19}H_{27}BrNO_2$ $[M+H]^+$ 380.1220, found 280.1223.

4-Bromo-6,6,6-trichlorohexyl 4-([1,1'-biphenyl]-4-yl)-4-oxobutanoate (61)

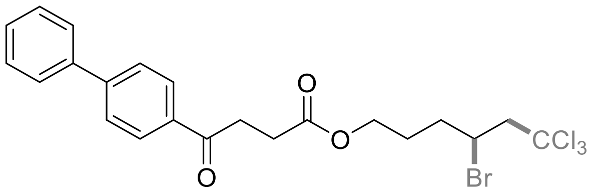

Following **GP-*b***, starting from 161 mg (0.5 mmol, 0.1M) of pent-4-en-1yl 4-([1,1'-biphenyl]-4-yl)-4-oxobutanoate (**1ae**), 99 μL (1 mmol, 2.0 equiv.) of bromotrichloromethane (**2a**) and 25 mg of g-$C_3N_4/Cs_2AgBiCl_6$ (90%/10%) catalyst in 5 mL of acetonitrile:water (95:5) mixture. The crude mixture was purified using silica gel chromatography (95:5 cyclohexane/ethyl acetate) to afford 177 mg of **61** (0.34 mmol, 68% yield) as a white solid (m.p.: 109.3-110.4 °C).

^1H NMR (400 MHz, $CDCl_3$): δ 8.13 – 8.01 (m, 2H), 7.73 – 7.67 (m, 2H), 7.66 – 7.59 (m, 2H), 7.50 – 7.43 (m, 2H), 7.43 – 7.34 (m, 1H), 4.41 – 4.33 (m, 1H), 4.18 (t, *J* = 6.2 Hz, 2H), 3.46 (dd, *J* = 15.8,

5.0 Hz, 1H), 3.35 (t, J = 6.6 Hz, 2H), 3.22 (dd, J = 15.9, 5.4 Hz, 1H), 2.79 (t, J = 6.5 Hz, 2H), 2.26 – 2.09 (m, 1H), 2.06 – 1.92 (m, 2H), 1.91 – 1.77 (m, 1H).

$^{13}C\{^1H\}$ NMR (101 MHz, $CDCl_3$): δ 198.0, 173.2, 146.6, 140.2, 135.6, 129.4, 129.1, 128.7, 127.7, 127.7, 97.4, 64.0, 62.9, 48.8, 36.4, 33.8, 28.7, 27.0.

HRMS (EI, m/z): calcd. for $C_{22}H_{22}BrCl_3NaO_3$ $[M+Na]^+$ 540.9710, found 540.9710.

4-Bromo-6-cyanohexyl 4-([1,1'-biphenyl]-4-yl)-4-oxobutanoate (62)

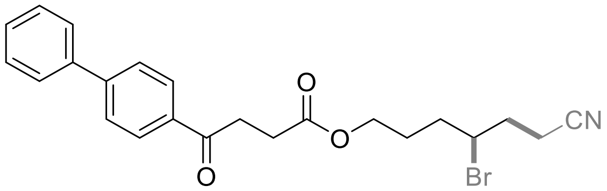

Following **GP-*b***, starting from 161 mg (0.5 mmol, 0.1M) of pent-4-en-1yl 4-([1,1'-biphenyl]-4-yl)-4-oxobutanoate (**1ae**), 71 µL (1 mmol, 2.0 equiv.) of bromoacetonitrile (**2c**) and 25 mg of g-$C_3N_4/Cs_2AgBiCl_6$ (90%/10%) catalyst in 5 mL of acetonitrile:water (95:5) mixture. The crude mixture was purified using silica gel chromatography (9:1 cyclohexane/ethyl acetate) to afford 177 mg of **62** (0.40 mmol, 80% yield) as a yellowish dense oil.

^1H NMR (400 MHz, $CDCl_3$): δ 8.13 – 8.02 (m, 2H), 7.75 – 7.66 (m, 2H), 7.66 – 7.57 (m, 2H), 7.53 – 7.44 (m, 2H), 7.43 – 7.36 (m, 1H), 4.30 – 3.98 (m, 3H), 3.35 (t, J = 6.5 Hz, 2H), 2.78 (t, J = 6.5 Hz, 2H), 2.63 – 2.54 (m, 2H), 2.26 – 2.00 (m, 2H), 2.01 – 1.60 (m, 4H).

$^{13}C\{^1H\}$ NMR (101 MHz, $CDCl_3$): δ 197.5, 172.6, 145.7, 139.5, 135.0, 128.7, 128.4, 128.1, 127.1, 127.0, 118.5, 63.3, 54.0, 35.1, 34.4, 33.6, 28.0, 26.6, 15.8.

HRMS (EI, m/z): calcd. for $C_{23}H_{25}BrNO_3$ $[M+H]^+$ 442.1012, found 442.1022.

4-Bromo-6,6,6-trichlorohexyl 2-(4-isobutylphenyl)propanoate (63)

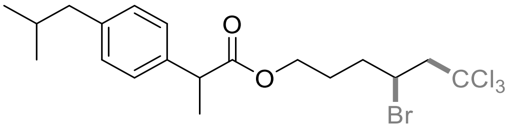

Following **GP-*b***, starting from 137 mg (0.5 mmol, 0.1M) of pent-4-en-1-yl 2-(4-isobutylphenyl)propanoate (**1af**), 99 µL (1 mmol, 2.0 equiv.) of bromotrichloromethane (**2a**) and 25 mg of g-$C_3N_4/Cs_2AgBiCl_6$ (90%/10%) catalyst in 5 mL of acetonitrile:water (95:5) mixture. The crude mixture was purified using silica gel chromatography (95:5 cyclohexane/ethyl acetate) to afford 234 mg of **63** (0.5 mmol, >99% yield; *ca.* 1:1 diastereoisomeric mixture) as a colorless dense oil.

^1H NMR (400 MHz, mixture of diasereoisomers, $CDCl_3$): δ 7.23 – 7.17 (m, 4H), 7.13 – 7.06 (m, 4H), 4.36 – 4.21 (m, 2H), 4.17 – 4.06 (m, 4H), 3.69 (q, J = 7.2 Hz, 2H), 3.41 (ddd, J = 15.8, 5.0, 0.9

Hz, 2H), 3.12 (ddd, *J* = 15.8, 5.5, 2.6 Hz, 2H), 2.45 (d, *J* = 7.2 Hz, 4H), 2.07 – 1.71 (m, 10H), 1.49 (d, *J* = 7.2 Hz, 6H), 0.90 (d, *J* = 6.6 Hz, 12H).

$^{13}C\{^1H\}$ NMR (101 MHz, mixture of diastereoisomers, $CDCl_3$): δ 174.8, 174.7, 140.7, 137.8, 137.8, 129.5, 127.3, 97.1, 63.5, 63.5, 62.7, 62.7, 48.4, 48.4, 45.3, 45.2, 35.9, 30.3, 26.7, 22.5, 18.5, 18.5.

HRMS (EI, m/z): calcd. for $C_{19}H_{27}BrCl_3O_2$ $[M+H]^+$ 471.0255, found 471.0238.

4-Bromo-6-cyanohexyl 2-(4-isobutylphenyl)propanoate (64)

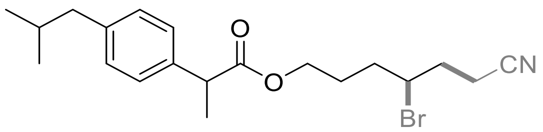

Following **GP-*b***, starting from 137 mg (0.5 mmol, 0.1M) of pent-4-en-1-yl 2-(4-isobutylphenyl)propanoate (**1af**), 71 µL (1 mmol, 2.0 equiv.) of bromoacetonitrile (**2c**) and 25 mg of g-C_3N_4/$Cs_2AgBiCl_6$ (90%/10%) catalyst in 5 mL of acetonitrile:water (95:5) mixture. The crude mixture was purified using silica gel chromatography (9:1 cyclohexane/DCM) to afford 169 mg of **64** (0.43 mmol, 86% yield; *ca*. 1:1 diastereoisomeric mixture) as a colorless dense oil.

^1H NMR (400 MHz, mixture of diastereoisomers, $CDCl_3$): δ 7.19 (d, *J* = 8.2 Hz, 4H), 7.10 (d, *J* = 8.0 Hz, 4H), 4.28 – 4.02 (m, 4H), 3.97 – 3.87 (m, 2H), 3.69 (q, *J* = 7.2 Hz, 2H), 2.63 – 2.49 (m, 4H), 2.45 (d, *J* = 7.2 Hz, 4H), 2.14 – 1.93 (m, 4H), 1.90 – 1.80 (m, 4H), 1.78 – 1.67 (m, 6H), 1.49 (d, *J* = 7.2 Hz, 6H), 0.90 (d, *J* = 6.6 Hz, 12H).

$^{13}C\{^1H\}$ NMR (101 MHz, mixture of diastereoisomers, $CDCl_3$): δ 174.5, 140.5, 137.6, 129.2, 127.0, 118.4, 63.3, 63.2, 53.8, 53.7, 45.0, 44.8, 35.0, 34.9, 34.4, 30.0, 26.5, 22.2, 18.1, 18.1, 15.8.

HRMS (EI, m/z): calcd. for $C_{20}H_{28}BrNNaO_2$ $[M+Na]^+$ 416.1196, found 416.1193.

3-Bromo-2-(cyanomethyl)-3-methylbutyl acetyl-*L*-phenylalaninate (65)

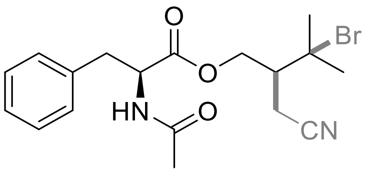

Following **GP-*b***, starting from 138 mg (0.5 mmol, 0.1M) of 3-methylbut-2-en-1-yl acety-*L*-phenylalaninate (**1ag**), 141 µL (2 mmol, 4.0 equiv.) of bromoacetonitrile (**2c**) and 25 mg of g-C_3N_4/$Cs_2AgBiCl_6$ (90%/10%) catalyst in 5 mL of acetonitrile:water (95:5) mixture. The crude mixture was purified using silica gel chromatography (95:5 cyclohexane/ethyl acetate) to afford 154 mg of **65** (0.39 mmol, 78% yield; *ca*. 1:1 diastereoisomeric mixture) as a colorless dense oil.

^{1}H NMR (400 MHz, mixture of diastereoisomers, $CDCl_3$): δ 7.35 – 7.19 (m, 6H), 7.18 – 7.09 (m, 4H), 6.32 (dd, *J* = 10.5, 7.8 Hz, 2H), 4.84 (dq, *J* = 7.9, 6.5 Hz, 2H), 4.52 (dd, *J* = 11.6, 4.1 Hz, 1H), 4.38 (dd, *J* = 11.6, 4.1 Hz, 1H), 4.11 (dd, *J* = 11.5, 7.5 Hz, 1H), 3.98 (dd, *J* = 11.6, 7.7 Hz, 1H), 3.16 – 3.01 (m, 4H), 2.65 – 2.57 (m, 2H), 2.42 – 2.31 (m, 1H), 2.23 (dd, *J* = 17.0, 8.0 Hz, 1H), 2.17 – 2.01 (m, 2H), 1.96 (d, *J* = 0.9 Hz, 6H), 1.79 (d, *J* = 4.5 Hz, 6H), 1.73 (d, *J* = 6.5 Hz, 6H).

$^{13}C\{^1H\}$ NMR (101 MHz, mixture of diastereoisomers, $CDCl_3$): δ 171.0, 170.9, 169.8, 169.7, 135.6, 135.6, 128.9, 128.5, 127.0, 127.0, 118.5, 118.4, 65.2, 65.1, 64.7, 64.6, 53.4, 53.4, 47.2, 47.2, 37.9, 37.8, 33.0, 32.9, 32.0, 31.8, 22.7, 17.7, 17.6.

HRMS (EI, m/z): calcd. for $C_{18}H_{24}BrN_2O_3$ $[M+H]^+$ 395.0965, found 395.0959.

3-(Bromomethyl)-1-tosyl-4-(2,2,2-trichloroethyl)pyrrolidine (66)

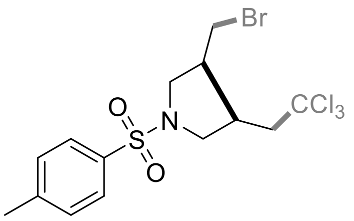

Product **66** was prepared following the conditions described in the general procedure **GP-*b***: from 125.5 mg (0.5 mmol, 0.1 M) of *N*,*N*-diallyl 4-methylbenzenesulfonamide (**1ah**), 98.5 µL (1 mmol, 2.0 equiv.) of bromotrichloromethane (**2a**) and 25 mg of *g*-$C_3N_4/Cs_2AgBiCl_6$ (90%/10%) catalyst (**CAT2**) in 5 mL of acetonitrile:water (95:5) mixture. After solvent removal under reduced pressure, the crude mixture was purified via silica gel chromatography (7:3 cyclohexane/ethyl acetate) to obtain 181 mg of product **66** (80% yield; the *cis*-isomer, accompanied by traces of the *trans*-isomer, has been obtained) as a yellow oil. Spectroscopic data for product **66** are in accordance with the literature.[S6]

^{1}H NMR (400 MHz, CD_3COCD_3): δ 7.84 – 7.69 (m, 2H), 7.46 (d, *J* = 8.1 Hz, 2H), 3.76 – 3.59 (m, 2H), 3.46 (d, *J* = 4.8 Hz, 2H), 3.40 – 3.26 (m, 1H), 3.26 – 3.04 (m, 2H), 2.90 – 2.63 (m, 3H), 2.44 (s, 3H).

$^{13}C\{^1H\}$ NMR (101 MHz, CD_3COCD_3): δ 144.9, 135.4, 131.1, 128.7, 100.2, 53.4, 52.4, 52.4, 45.7, 41.0, 32.6, 21.8.

3.3 Products *post*-functionalization

To demonstrate the versatility of the prepared compounds, product **5** was subjected to a post-functionalization step, encompassing a reaction protocol already known in the literature, which consisted in an aliphatic nucleophilic substitution with the azide anion (N_3^-).[S32] The employed reaction protocol is sketched in **Scheme S9**.

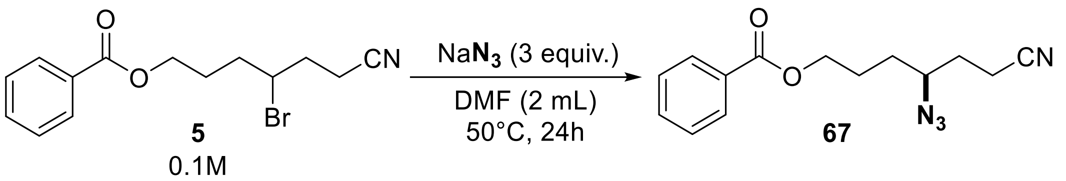

Scheme S9. Reaction protocol adopted for the post-functionalization of product **5**.

Synthesis of 4-azido-6-cyanohexyl benzoate (67)

Under an Ar atmosphere, to 62 mg of 4-bromo-6-cyanohexyl benzoate (**5**; 0.20 mmol, 0.1M) in DMF (2.0 mL) were added 39 mg of sodium azide (NaN_3; 0.60 mmol, 3.0 equiv.) in a dry glass vial. Then, the mixture was stirred at 50°C for 24 h using a sand bath. After completion according to TLC analysis, the reaction was quenched with water; the resulting mixture was then extracted with ethyl acetate, and the organic layer dried over Na_2SO_4. After solvent removal, the crude mixture was purified by flash column chromatography (silica gel; eluant: 8:2 cyclohexane/ethyl acetate) to give 57 mg of the desired product **67** as a colorless oil (0.2 mmol, >99% yield).

^{1}H NMR (400 MHz, $CDCl_3$): δ 8.07 – 7.99 (m, 2H), 7.61 – 7.52 (m, 1H), 7.49 – 7.40 (m, 2H), 4.44 – 4.30 (m, 2H), 3.55 – 3.50 (m, 1H), 2.49 (ddd, J = 7.8, 6.5, 1.3 Hz, 2H), 2.03 – 1.84 (m, 3H), 1.83 – 1.70 (m, 3H).

^{13}C{^{1}H} NMR (101 MHz, $CDCl_3$): δ 166.3, 132.9, 129.8, 129.3, 128.2, 118.5, 63.9, 60.6, 30.7, 30.0, 25.1, 14.1.

HRMS (EI, m/z): calcd. for $C_{14}H_{17}N_4O_2$ $[M+H]^+$ 273.1346, found 273.1341.

4. References

(S1) Tedesco, C.; Gregori, L.; Simbula, A.; Pitzalis, F.; Speltini, A.; Merlo, F.; Colella, S.; Listorti, A.; Mosconi, E.; Alothman, A. A.; Kaiser, W.; Saba, M.; Profumo, A.; De Angelis, F.; Malavasi, L. Reaction Mechanism of Hydrogen Generation and Nitrogen Fixation at Carbon Nitride/Double Perovskite Heterojunctions. *Adv. Energy Sustainability Res.* **2024**, *5*, 2400040.

(S2) Fairley, N.; Fernandez, V.; Richard-Plouet, M.; Guillot-Deudon, C.; Walton, J.; Smith, E.; Flahaut, D.; Greiner, M.; Biesinger, M.; Tougaard, S.; Morgan, D.; Baltrusaitis, J. Systematic and collaborative approach to problem solving using X-ray photoelectron spectroscopy. *Appl. Surf. Sci. Adv.* **2021**, *5*, 100112.

(S3) Akaike, K.; Aoyama, K.; Dekubo, S.; Onishi, A.; Kanai, K. Characterizing Electronic Structure near the Energy Gap of Graphitic Carbon Nitride Based on Rational Interpretation of Chemical Analysis. *Chem. Mater.* **2018**, *30*, 2341–2352.

(S4) Alwin, E.; Nowicki, W.; Wojcieszak, R.; Zieliński, M.; Pietrowski, M. Elucidating the structure of the graphitic carbon nitride nanomaterials via X-ray photoelectron spectroscopy and X-ray powder diffraction techniques. *Dalton Trans.* **2020**, *49*, 12805–12813.

(S5) NIST X-ray Photoelectron Spectroscopy Database, NIST Standard Reference Database Number 20, National Institute of Standards and Technology, Gaithersburg MD, 20899 (**2000**). DOI: 10.18434/T4T88K

(S6) Gilbert, B. C.; Kalz, W.; Lindsay, C. I.; McGrail, P. T.; Parsons, A. F.; Whittaker, D. T. E. Initiation of Radical Cyclisation Reactions Using Dimanganese Decacarbonyl. A Flexible Approach to Preparing 5-Membered Rings. *J. Chem. Soc., Perkin Trans. 1* **2000**, 1187–1194.

(S7) Wille, U. Radical Cascades Initiated by Intermolecular Radical Addition to Alkynes and Related Triple Bond Systems. *Chem. Rev.* **2013**, *113*, 813–853.

(S8) Hamadamin, A.; Benazzi, V.; Campalani, C.; Quattri, L.; Ravelli, D.; Hussain, F.; Perosa, A.; Selva, M.; Protti, S. Nitrogen-doped Carbon Dots as Biobased Catalysts for Visible Light Driven 1,2-Functionalization of Olefins through an Atom Transfer Radical Addition Process. *ChemCatChem* **2023**, *15*, e202300708.

(S9) Hemming, D. S.; Talbot, E. P.; Steel, P. G. A Mild Copper Catalyzed Method for the Selective Deprotection of Aryl Allyl Ethers. *Tetrahedron Lett.* **2017**, *58*, 17–20.

(S10) Ohmura, N.; Nakamura, A.; Hamasaki, A.; Tokunaga, M. Hydrolytic Deallylation of *N*-Allyl Amides Catalyzed by Pd^{II} Complexes. *Eur. J. Org. Chem.* **2008**, *2008*, 5042–5045.

(S11) Kathe, P. M.; Berkefeld, A.; Fleischer, I. Nickel Hydride Catalyzed Cleavage of Allyl Ethers Induced by Isomerization. *Synlett* **2021**, *32*, 1629–1632.

(S12) Sun, P.; Zhang, Z.; Wang, X.; Li, L.; Li, Y.; Li, Z. Cobalt-Catalyzed Intermolecular Hydroamination of Unactivated Alkenes Using NFSI as Nitrogen Source. *Chin. J. Chem.* **2022**, *40*, 1066–1072.

(S13) Lang, S. B.; O'Nele, K. M.; Douglas, J. T.; Tunge, J. A. Dual Catalytic Decarboxylative Allylations of α-Amino Acids and Their Divergent Mechanisms. *Chem. Eur. J.* **2015**, *21*, 18589–18593.

(S14) Mancheno, D. E.; Thornton, A. R.; Stoll, A. H.; Kong, A.; Blakey, S. B. Copper-Catalyzed Olefin Aminoacetoxylation. *Org. Lett.* **2010**, *12*, 4110–4113.

(S15) Mastitski, A.; Vellemäe, E.; Smorodina, V.; Konist, A.; Järv, J. Alcoholysis of Primary Amides in the Presence of CF_3SO_3H. *Org. Prep. Proced. Int.* **2023**, *55*, 458–468.

(S16) Mahajani, N. S.; Meador, R. I. L.; Smith, T. J.; Canarelli, S. E.; Adhikari, A. A.; Shah, J. P.; Russo, C. M.; Wallach, D. R.; Howard, K. T.; Millimaci, A. M.; Chisholm, J. D. Ester Formation via Symbiotic Activation Utilizing Trichloroacetimidate Electrophiles. *J. Org. Chem.* **2019**, *84*, 7871–7882.

(S17) Chun, S.; Chung, Y. K. Transition-Metal-Free Poly(Thiazolium) Iodide/1,8-Diazabicyclo[5.4.0]Undec-7-Ene/Phenazine-Catalyzed Esterification of Aldehydes with Alcohols. *Org. Lett.* **2017**, *19*, 3787–3790.

(S18) Yu, H.; Wang, J.; Wu, Z.; Zhao, Q.; Dan, D.; Han, S.; Tang, J.; Wei, Y. Aldehydes as Potential Acylating Reagents for Oxidative Esterification by Inorganic Ligand-Supported Iron Catalysis. *Green Chem.* **2019**, *21*, 4550–4554.

(S19) Scheidt, F.; Thiehoff, C.; Yilmaz, G.; Meyer, S.; Daniliuc, C. G.; Kehr, G.; Gilmour, R. Fluorocyclisation via I(I)/I(III) Catalysis: A Concise Route to Fluorinated Oxazolines. *Beilstein J. Org. Chem.* **2018**, *14*, 1021–1027.

(S20) Mohanty, A.; Sadhukhan, S.; Nayak, M. K.; Roy, S. Aminocarbonylation Reaction Using a Pd–Sn Heterobimetallic Catalyst: Three-Component Coupling for Direct Access of the Amide Functionality. *J. Org. Chem.* **2024**, *89*, 1010–1017.

(S21) Parella, R.; Babu, S. A. Pd(II)-Catalyzed, Picolinamide-Assisted, *Z*-Selective γ-Arylation of Allylamines To Construct *Z*-Cinnamylamines. *J. Org. Chem.* **2017**, *82*, 6550–6567.

(S22) Suzuki, A.; Kamei, Y.; Yamashita, M.; Seino, Y.; Yamaguchi, Y.; Yoshino, T.; Kojima, M.; Matsunaga, S. Photocatalytic Deuterium Atom Transfer Deuteration of Electron-Deficient Alkenes with High Functional Group Tolerance. *Angew. Chem. Int. Ed.* **2023**, *62*, e202214433.

(S23) Wood, D. P.; Guan, W.; Lin, S. Titanium and Cobalt Bimetallic Radical Redox Relay for the Isomerization of *N*-Bz Aziridines to Allylic Amides. *Synthesis* **2021**, *53*, 4213–4220.

(S24) Ramanjaneyulu, B. T.; Reddy, V.; Arde, P.; Mahesh, S.; Anand, R. V. Combining Oxidative *N*-Heterocyclic Carbene Catalysis with Click Chemistry: A Facile One-Pot Approach to 1,2,3-Triazole Derivatives. *Chem. Asian J.* **2013**, *8*, 1489–1496.

(S25) Huang, C.; Wu, D.; Li, Y.; Yin, G. Asymmetric *Anti* -Selective Borylalkylation of Terminal Alkynes by Nickel Catalysis. *J. Am. Chem. Soc.* **2023**, *145*, 18722–18730.

(S26) Hazra, C. K.; Oestreich, M. Copper(I)-Catalyzed Regio- and Chemoselective Single and Double Addition of Nucleophilic Silicon to Propargylic Chlorides and Phosphates. *Org. Lett.* **2012**, *14*, 4010–4013.

(S27) Elías-Rodríguez, P.; Matador, E.; Benítez, M.; Tejero, T.; Díez, E.; Fernández, R.; Merino, P.; Monge, D.; Lassaletta, J. M. Silver-Free Gold-Catalyzed Heterocyclizations through Intermolecular H-Bonding Activation. *J. Org. Chem.* **2023**, *88*, 2487–2492.

(S28) Elwood, J. M. L.; Henry, M. C.; Lopez-Fernandez, J. D.; Mowat, J. M.; Boyle, M.; Buist, B.; Livingstone, K.; Jamieson, C. Functionalized Tetrazoles as Latent Active Esters in the Synthesis of Amide Bonds. *Org. Lett.* **2022**, *24*, 9491–9496.

(S29) Li, H.; Zhao, P.-G.; Wang, C.-Y.; Zhang, R.-Y.; Li, J.-J.; Wu, Y.; Wang, P. SPSiPs, a Class of Diphosphine Ligands Based on SPSiOL with a Large Dihedral Angle. *Org. Lett.* **2023**, *25*, 3859–3863.

(S30) Zheng, Y.-L.; Daneshfar, O.; Li, J.-Y.; Masson-Makdissi, J.; Pinault-Masson, É.; Newman, S. G. Nickel-Catalyzed Transesterification of Methyl Esters. *Synlett* **2024**, *35*, 908–914.

(S31) Bian, K.-J.; Nemoto, D.; Kao, S.-C.; He, Y.; Li, Y.; Wang, X.-S.; West, J. G. Modular Difunctionalization of Unactivated Alkenes through Bio-Inspired Radical Ligand Transfer Catalysis. *J. Am. Chem. Soc.* **2022**, *144*, 11810–11821.

(S32) Li, D.; Mao, T.; Huang, J.; Zhu, Q. Copper-Catalyzed Bromodifluoroacetylation of Alkenes with Ethyl Bromodifluoroacetate. *J. Org. Chem.* **2018**, *83*, 10445–10452.

(S33) Nikitas, N. F.; Voutyritsa, E.; Gkizis, P. L.; Kokotos, C. G. Metal-free Photochemical Atom Transfer Radical Addition (ATRA) of $BrCCl_3$ to Alkenes. *Eur. J. Org. Chem.* **2021**, *2021*, 96–101.

(S34) Voutyritsa, E.; Nikitas, N.; Apostolopoulou, M.; Gerogiannopoulou, A.; Kokotos, C. Photoorganocatalytic Atom Transfer Radical Addition of Bromoacetonitrile to Aliphatic Olefins. *Synthesis* **2018**, *50*, 3395–3401.

(S35) He, J.; Yan, B.; Meng, J.; Ran, M.; Zhou, Y.; Deng, J.; Li, C.; Yao, Q. Study of Rhodamine-Based Fluorescent Probes for Organic Radical Intermediates. *Eur. J. Org. Chem.* **2021**, *2021*, 4059–4064.

(S36) Tejeda-Serrano, M.; Lloret, V.; Márkus, B. G.; Simon, F.; Hauke, F.; Hirsch, A.; Doménech-Carbó, A.; Abellán, G.; Leyva-Pérez, A. Few-layer Black Phosphorous Catalyzes Radical Additions to Alkenes Faster than Low-valence Metals. *ChemCatChem* **2020**, *12*, 2226–2232.

(S37) Chaibuth, P.; Chuaytanee, N.; Hojitsiriyanont, J.; Chainok, K.; Wacharasindhu, S.; Reiser, O.; Sukwattanasinitt, M. Copper(II) complexes of quinoline-based ligands for efficient photoredox catalysis of atom transfer radical addition (ATRA) reaction. *New J. Chem.* **2022**, *46*, 12158–12168.

5. Copy of ^{1}H and ^{13}C NMR spectra

4-Pentenyl benzoate (1a)

^{1}H-NMR (400MHz)

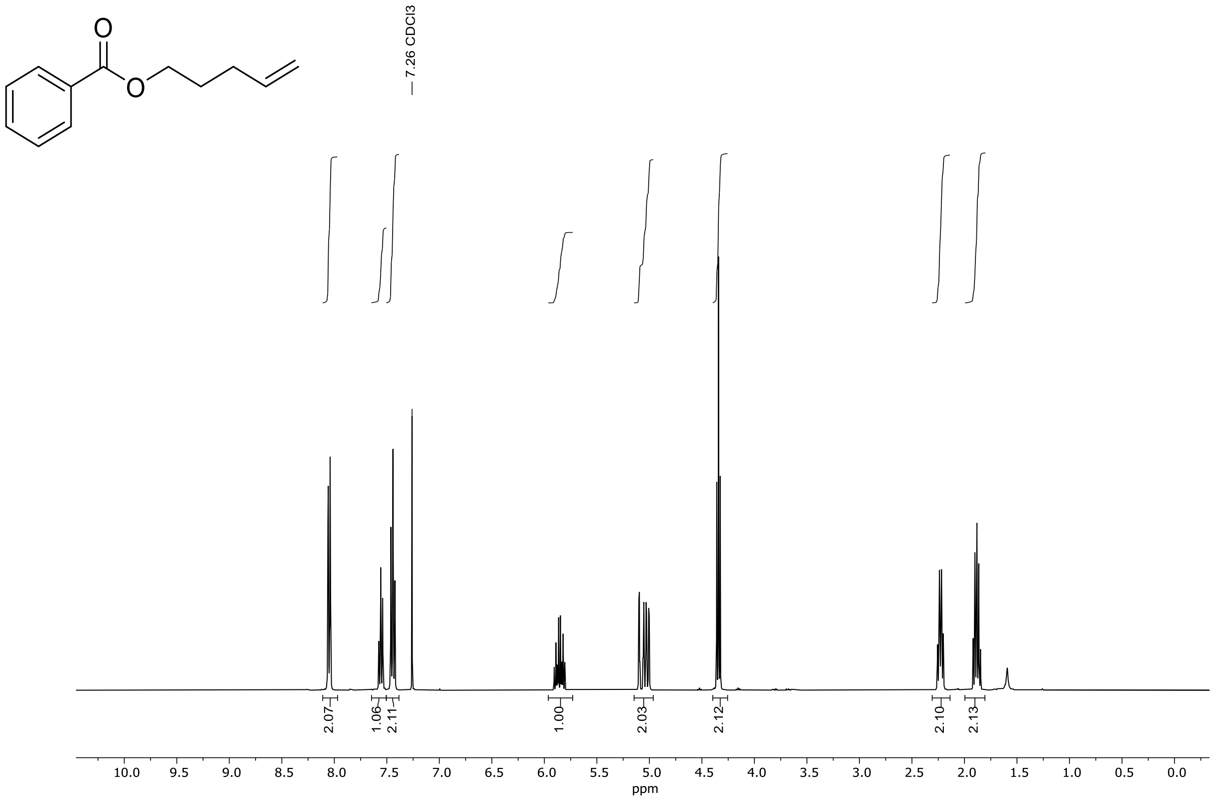

^{13}C-NMR (101MHz)

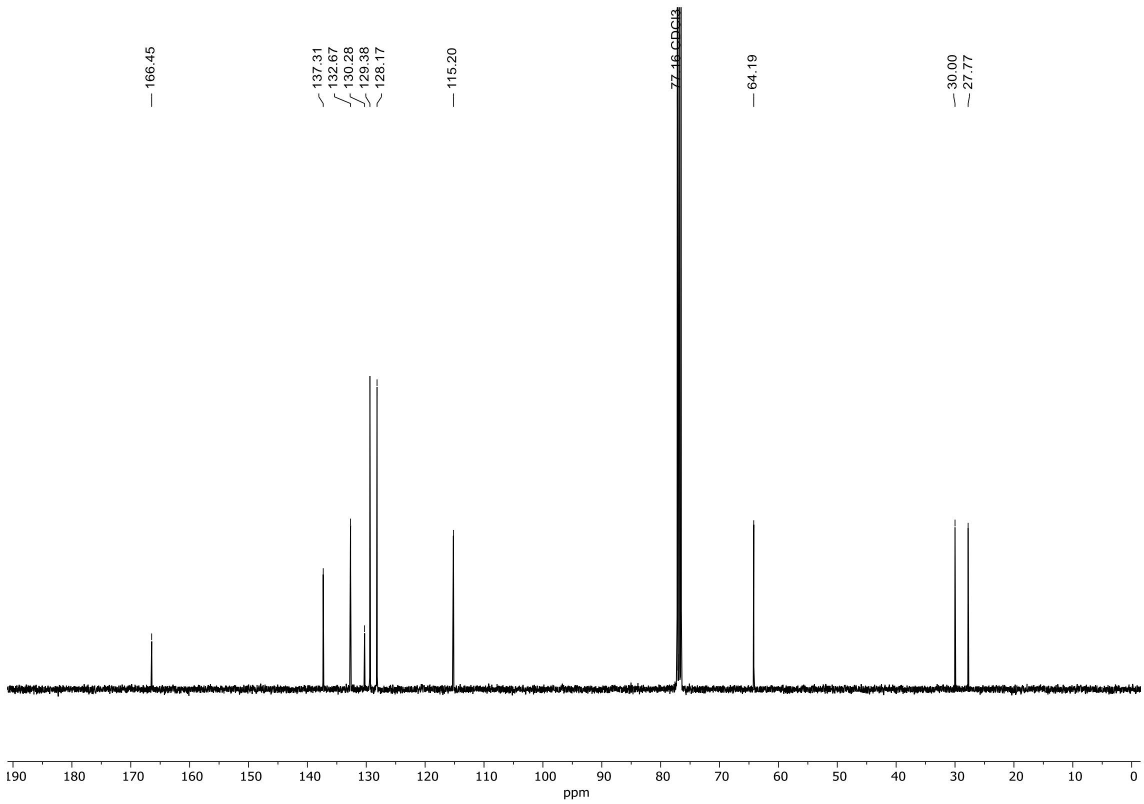

4-Pentenyl 4-methylbenzenesulfonate (1b)

1H-NMR (400MHz)

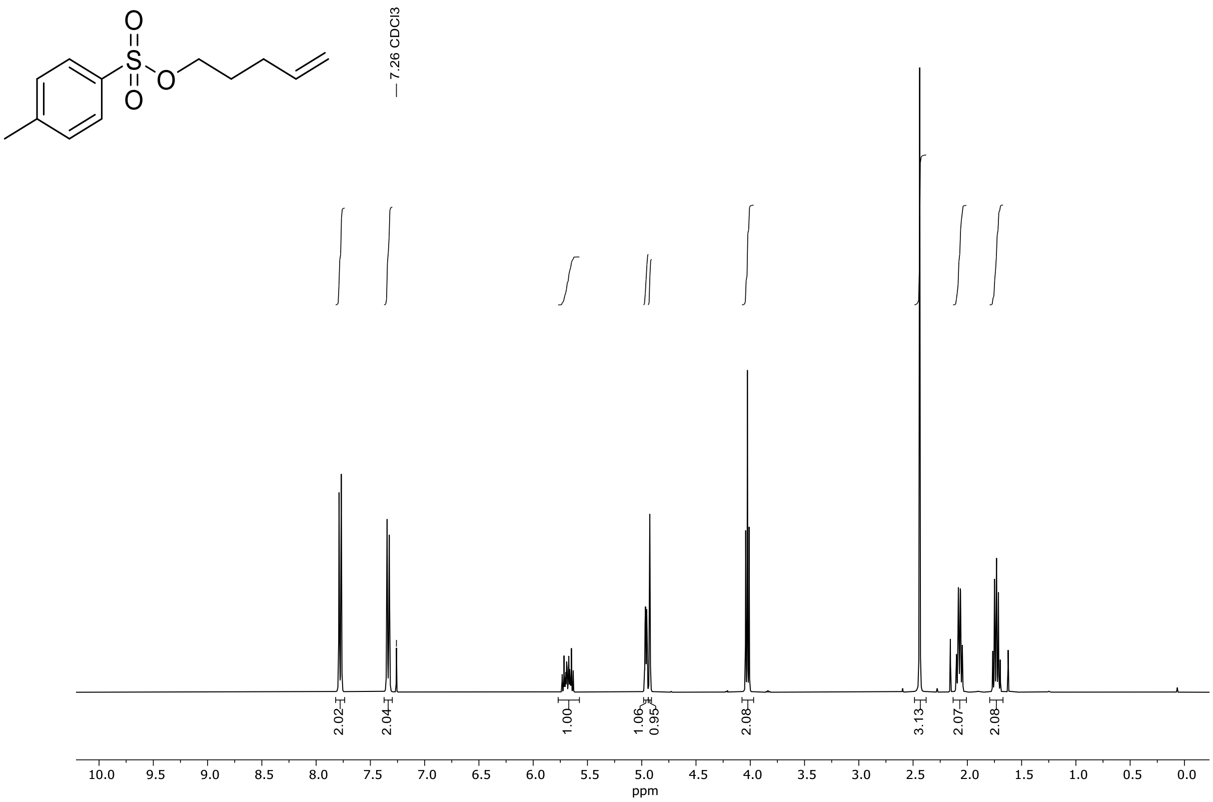

13C-NMR (101MHz)

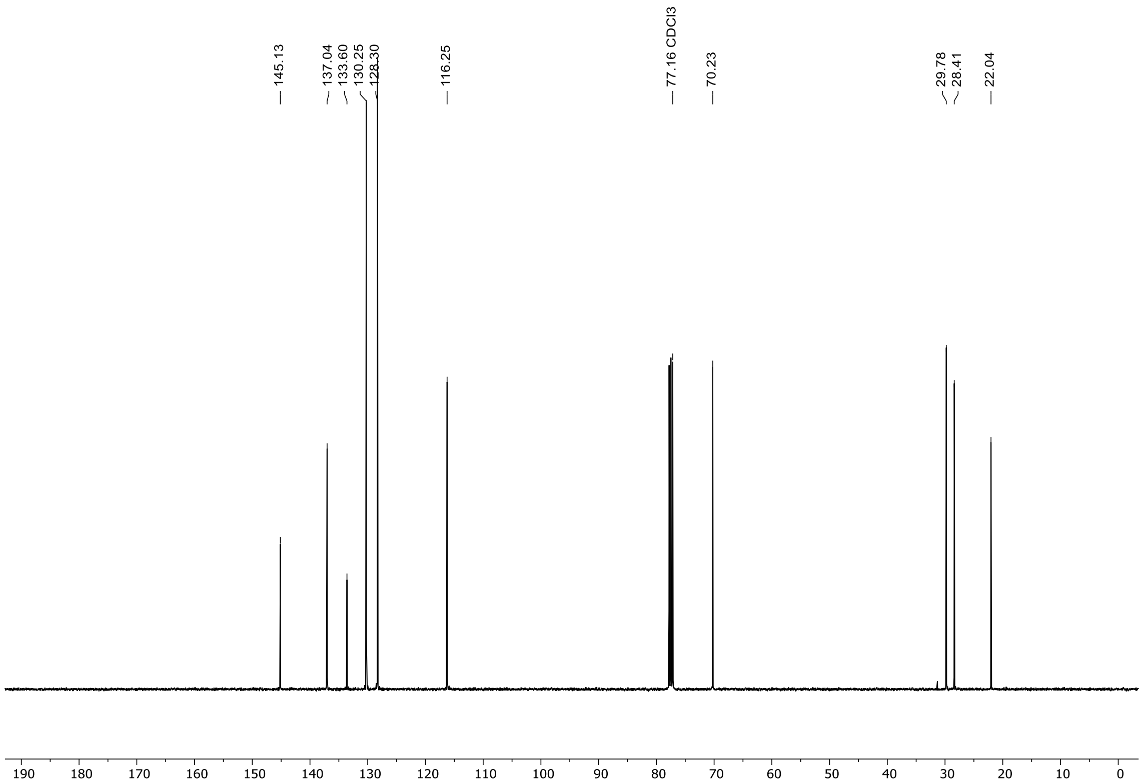

Allyl benzoate (1c)

^{1}H-NMR (400MHz)

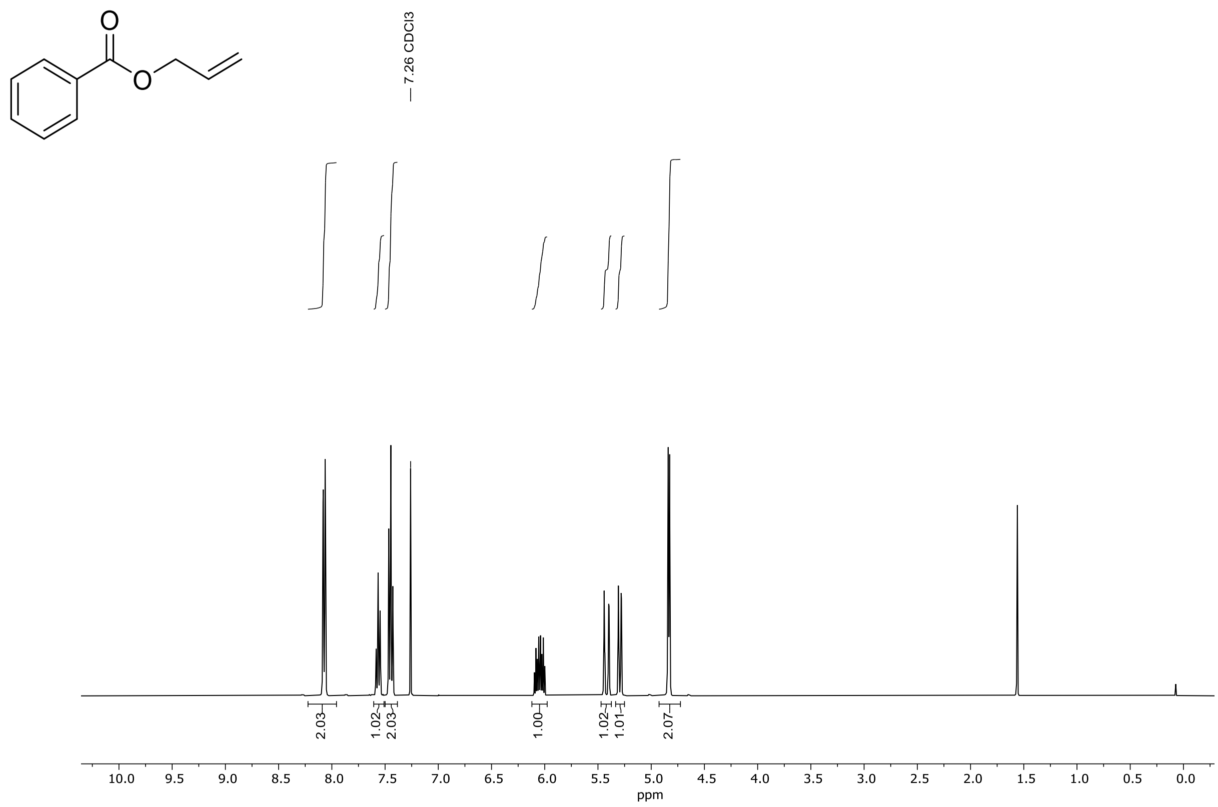

^{13}C-NMR (101MHz)

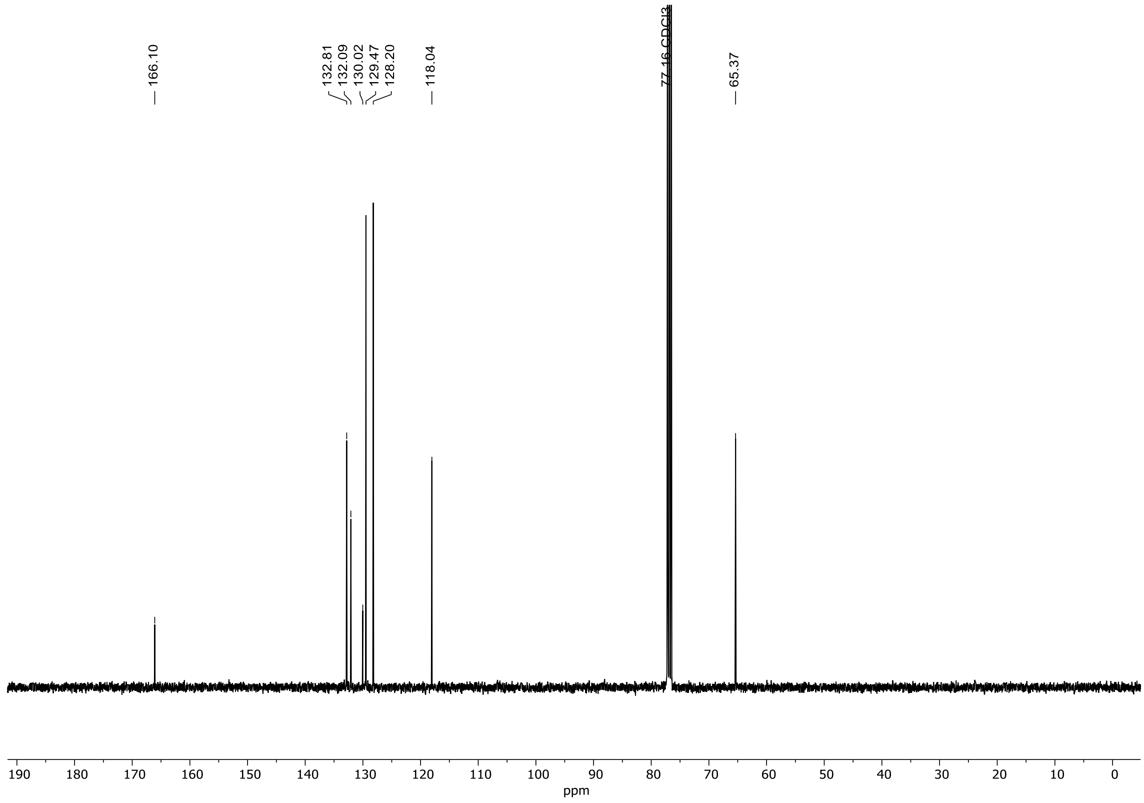

2-Methylallyl benzoate (1d)

^{1}H-NMR (400MHz)

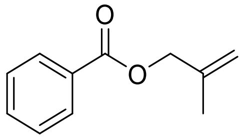

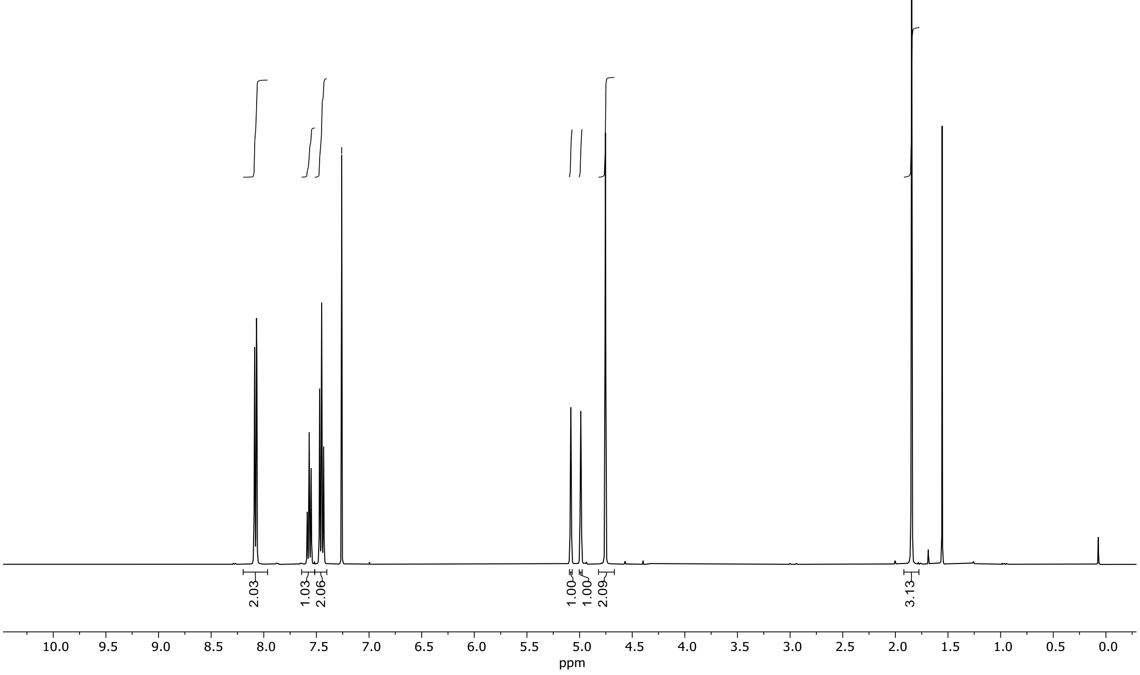

^{13}C-NMR (101MHz)

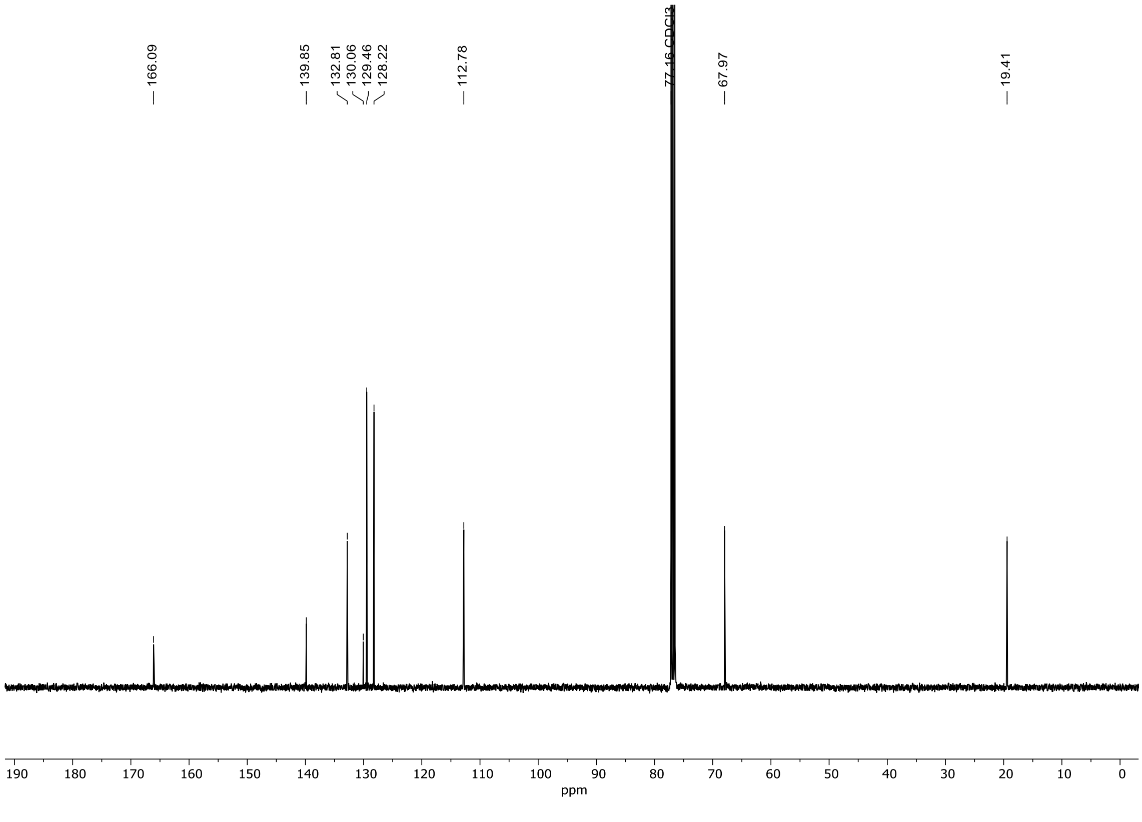

3-Methylbut-2-en-1-yl benzoate (1e)

^{1}H-NMR (400MHz)

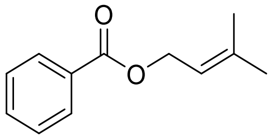

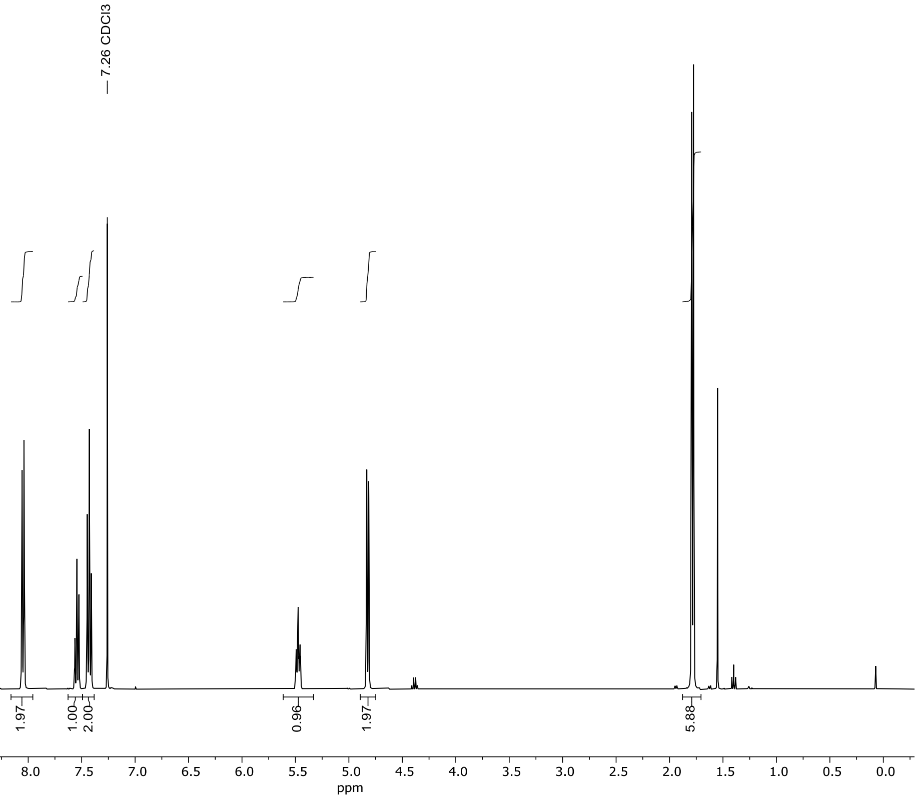

^{13}C-NMR (101MHz)

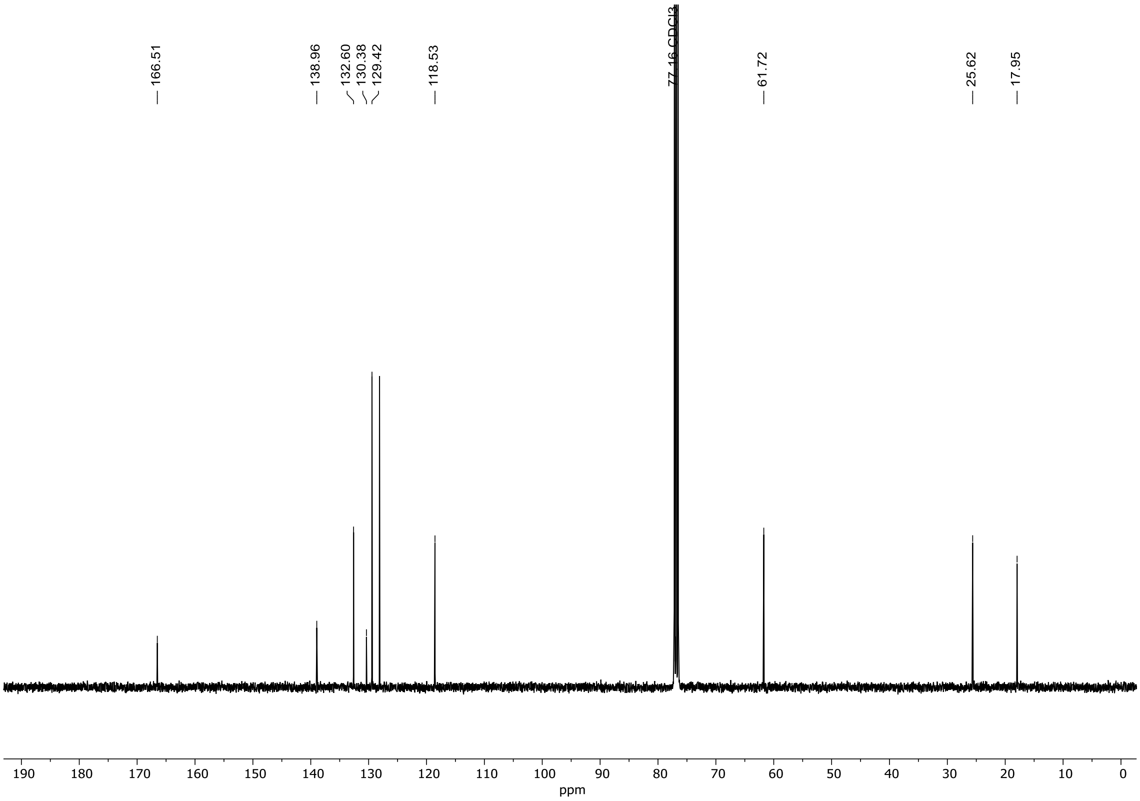

Cinnamyl benzoate (1f)

^{1}H-NMR (400MHz)

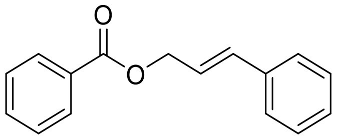

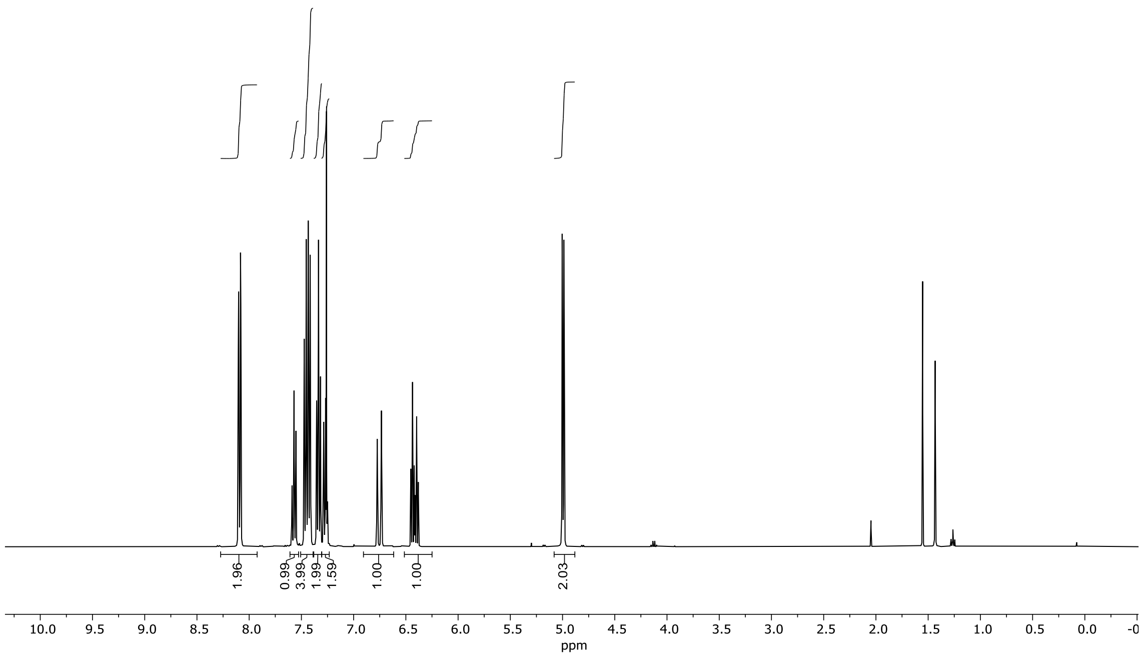

^{13}C-NMR (101MHz)

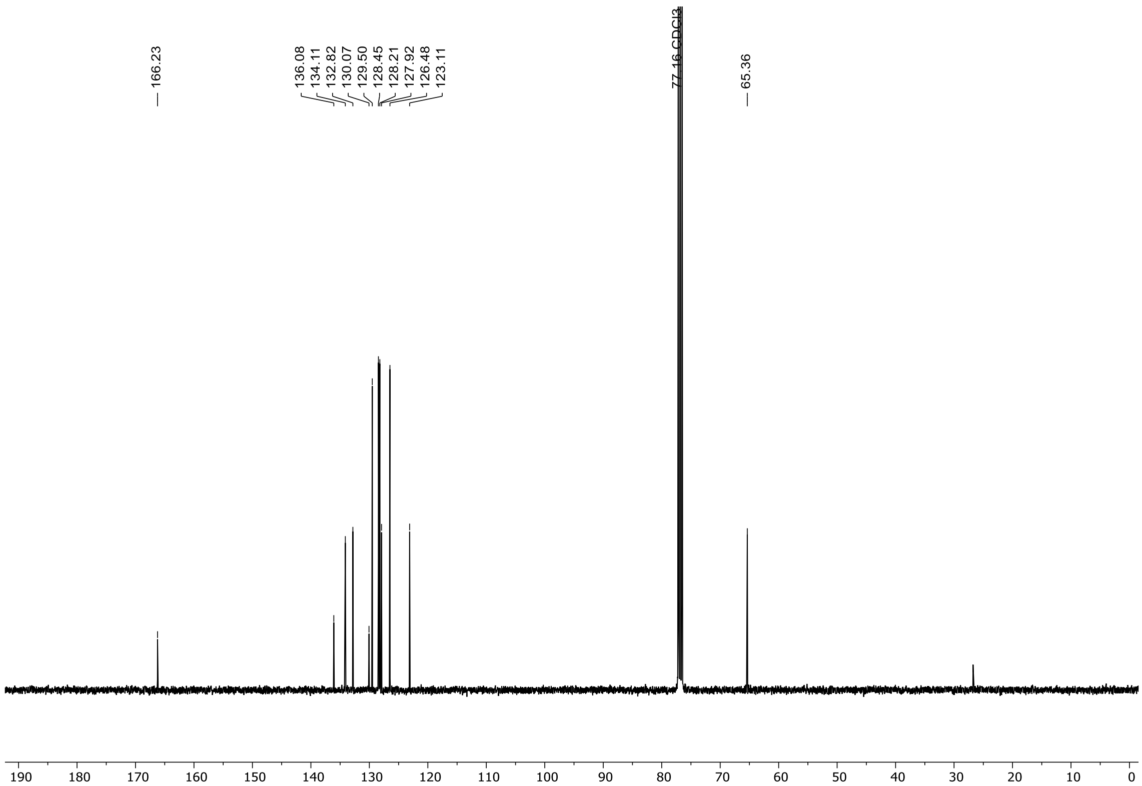

But-2-en-1-yl benzoate (1g)

^{1}H-NMR (400MHz)

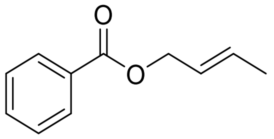

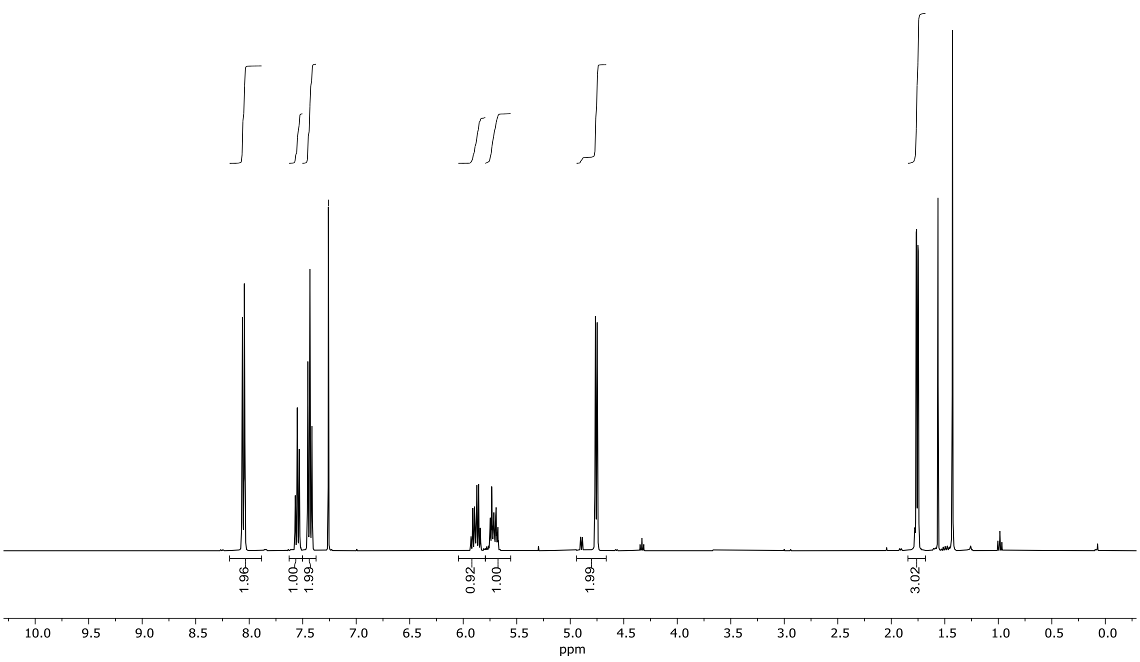

^{13}C-NMR (101MHz)

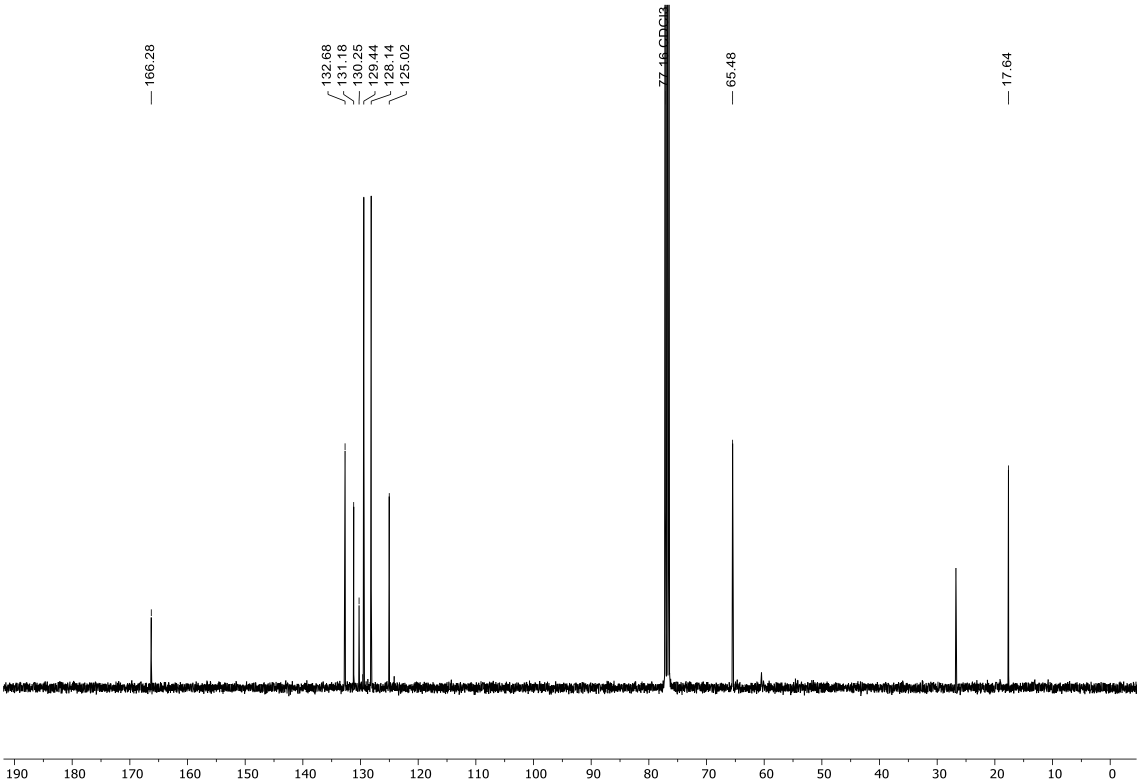

N-(Pent-4-en-1-yl) benzamide (1h)

^{1}H-NMR (400MHz)

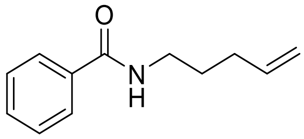

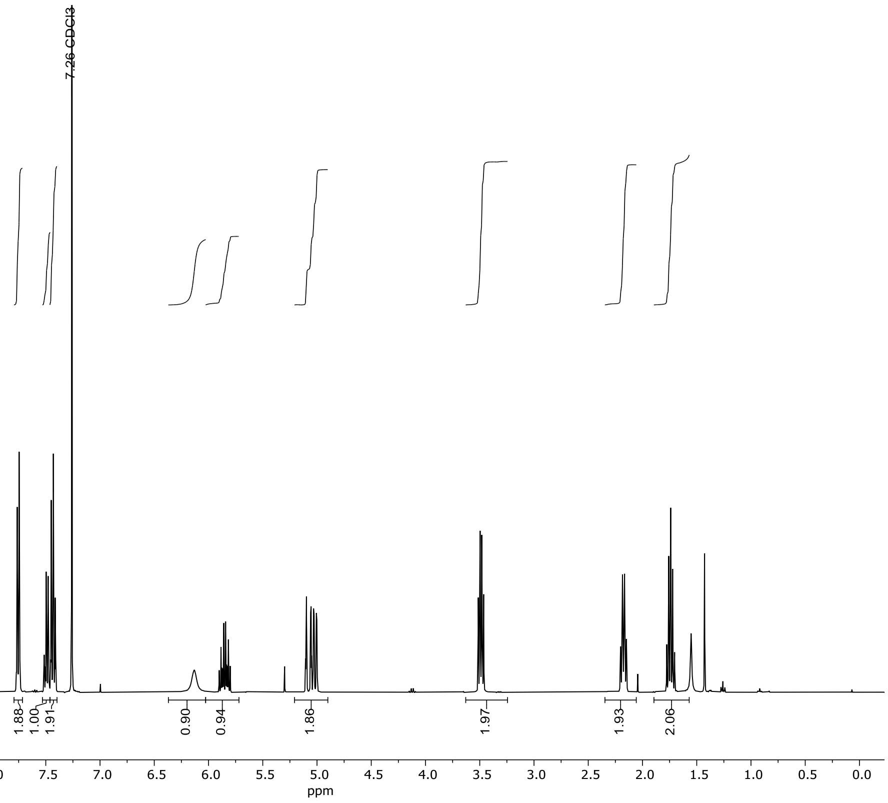

^{13}C-NMR (101MHz)

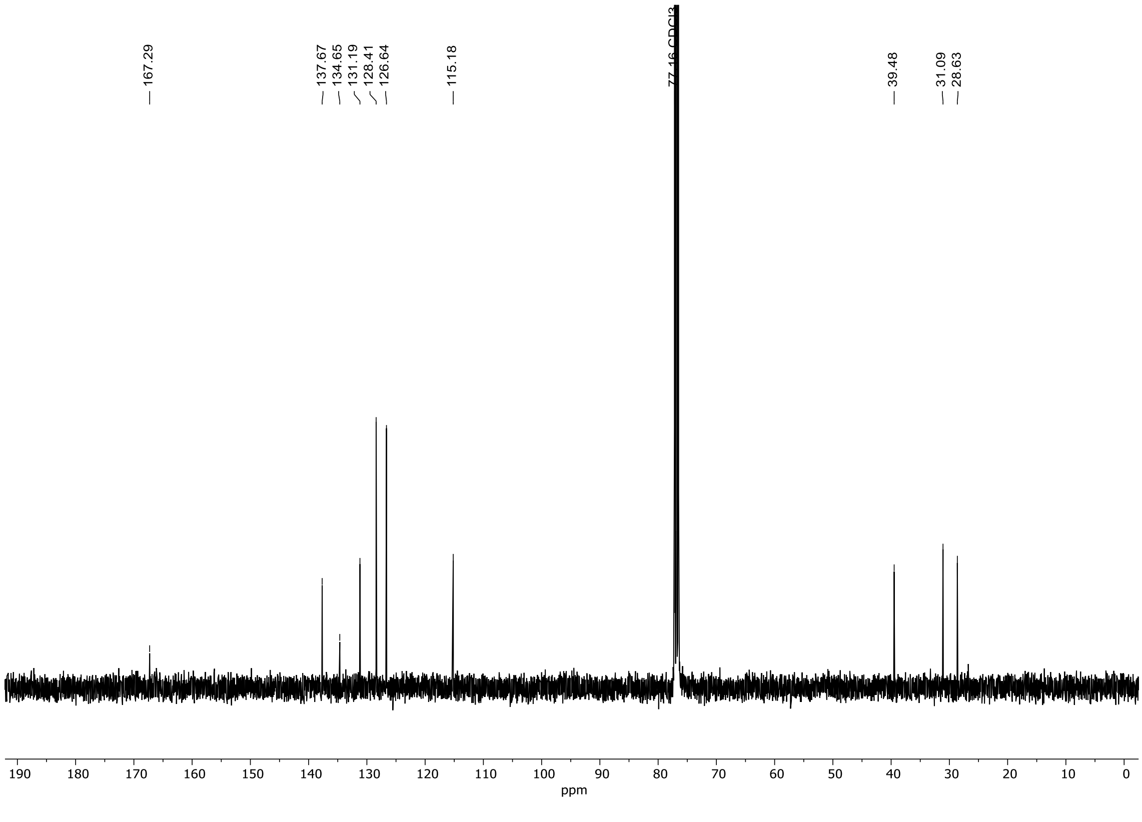

N-Allyl benzamide (1i)

^{1}H-NMR (400MHz)

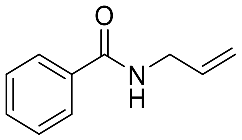

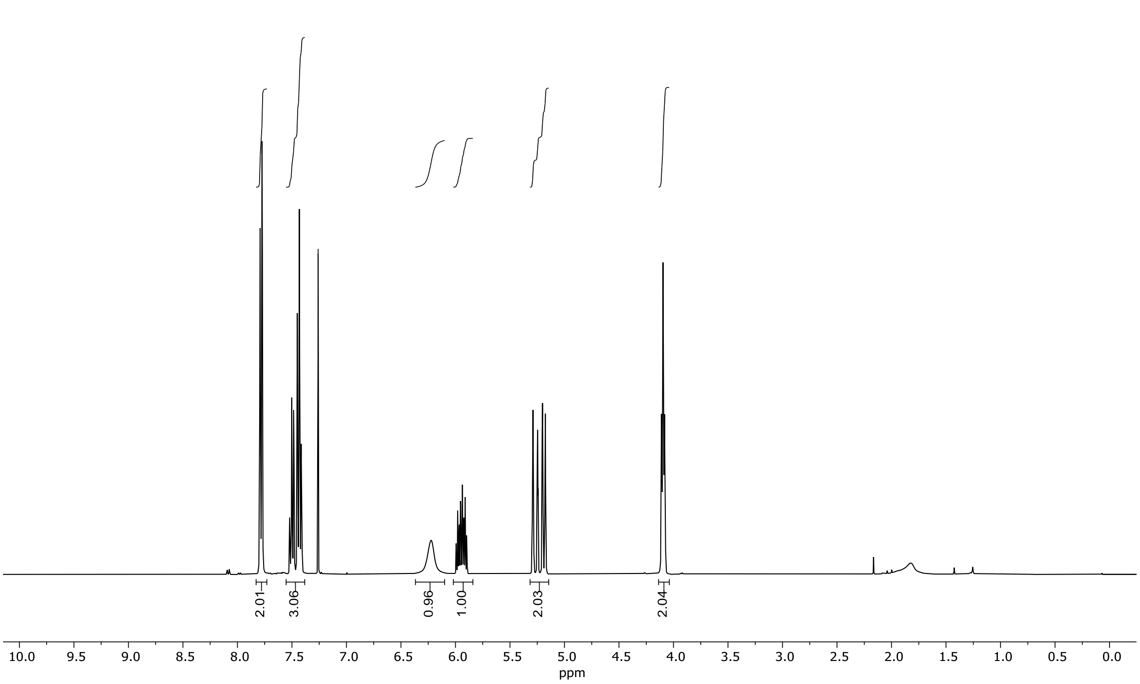

^{13}C-NMR (101MHz)

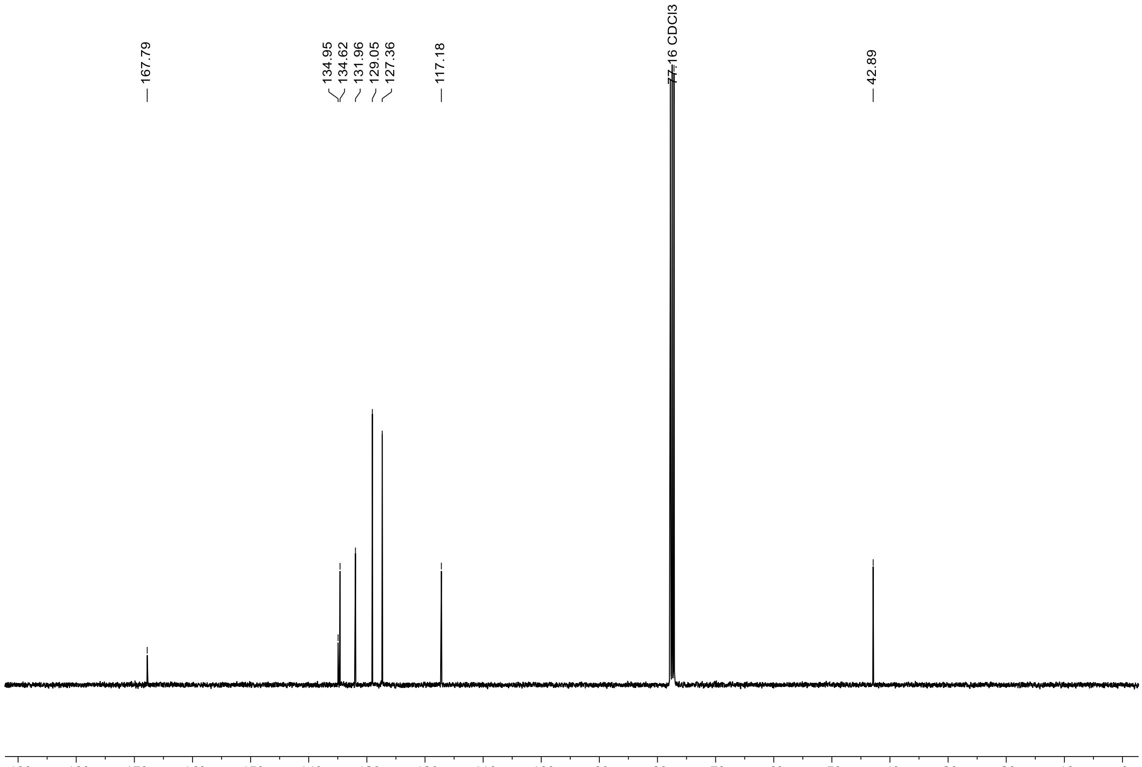

N-Allyl 1-naphtamide (1j)

^{1}H-NMR (400MHz)

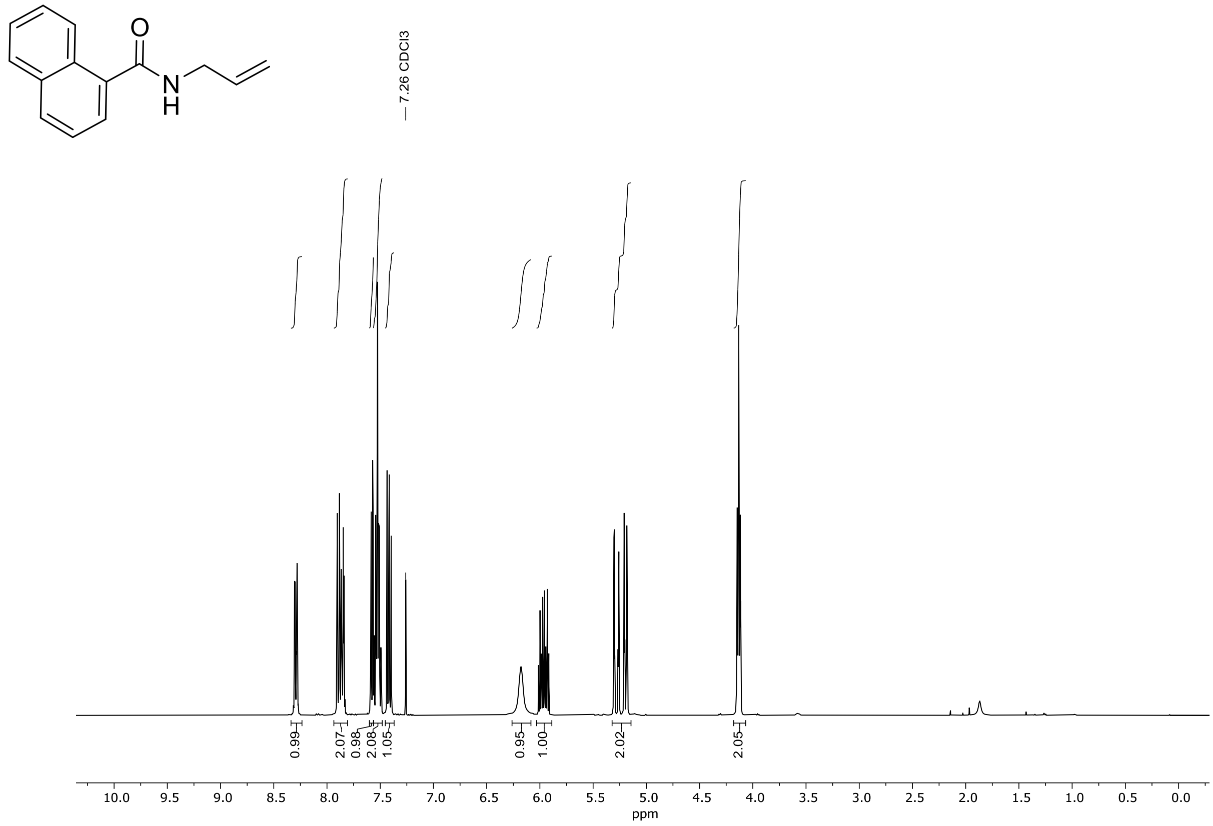

^{13}C-NMR (101MHz)

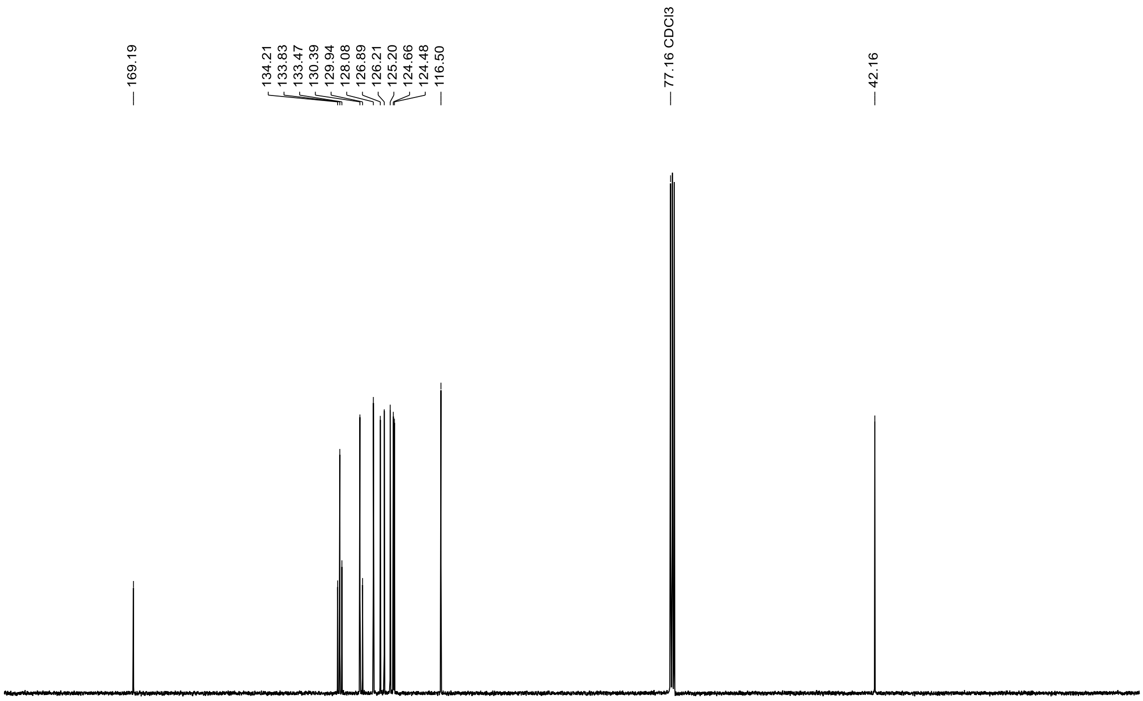

N-Allyl 4-methylbenzenesulfonamide (1k)

¹H-NMR (400MHz)

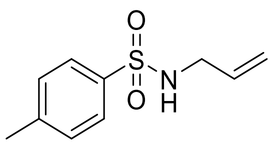

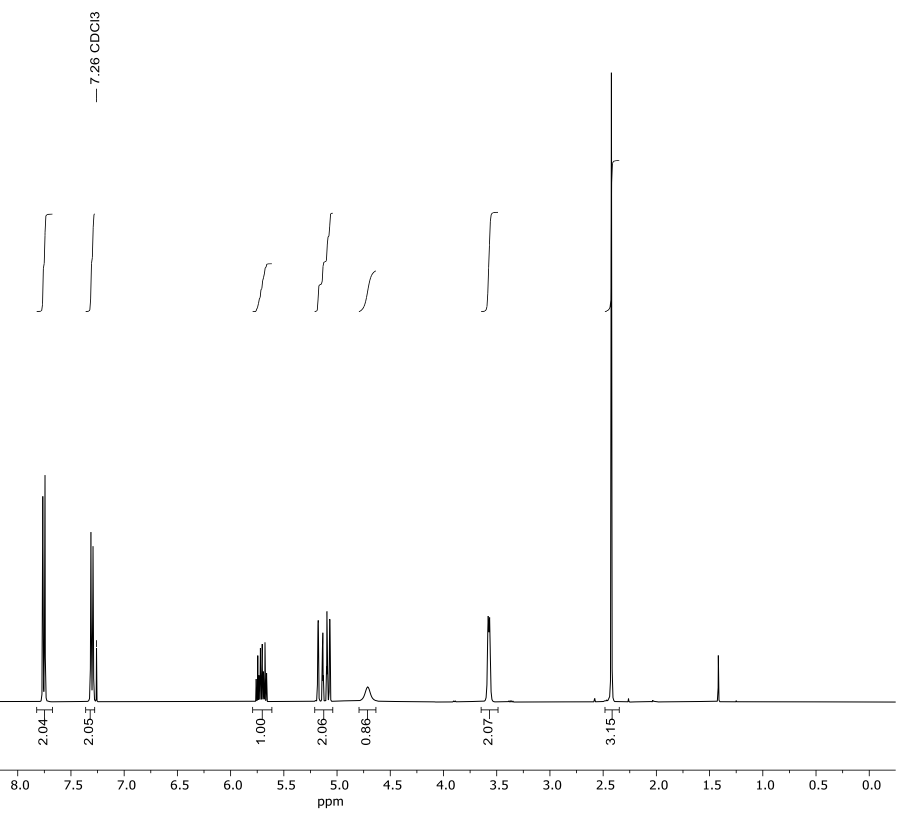

¹³C-NMR (101MHz)

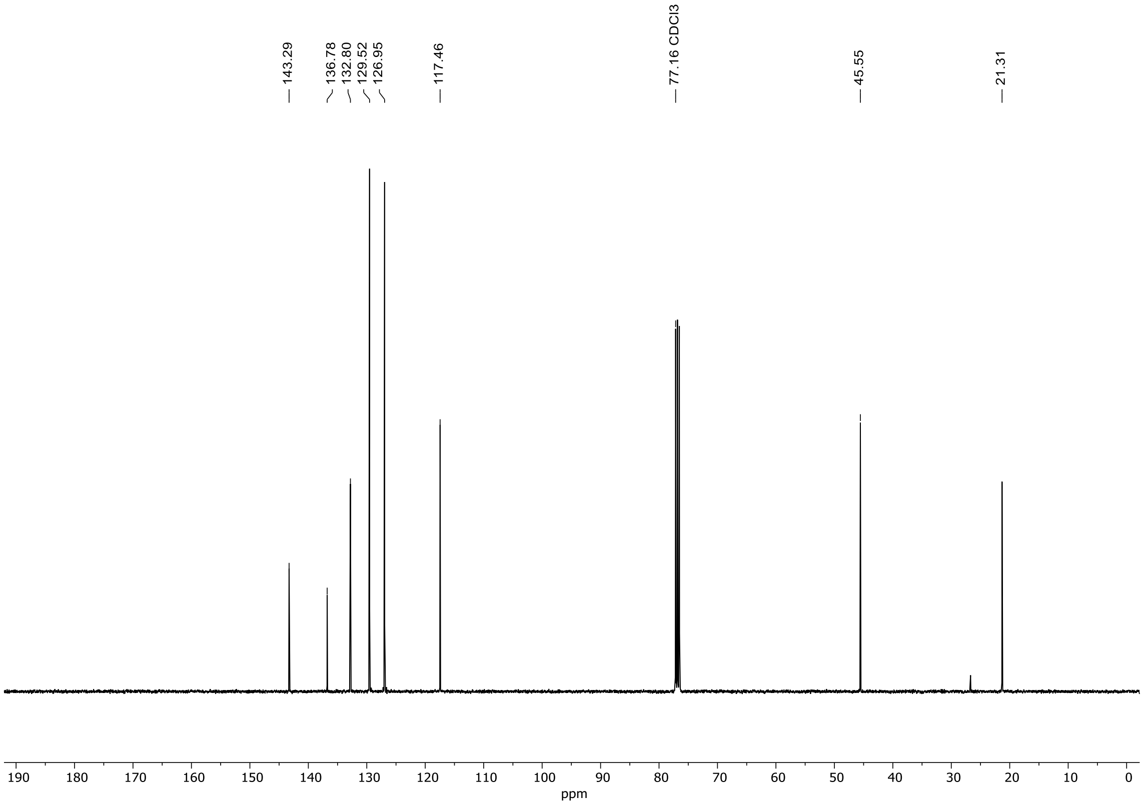

1-Allyl-pyrrolidin-2-one (1l)

^{1}H-NMR (400MHz)

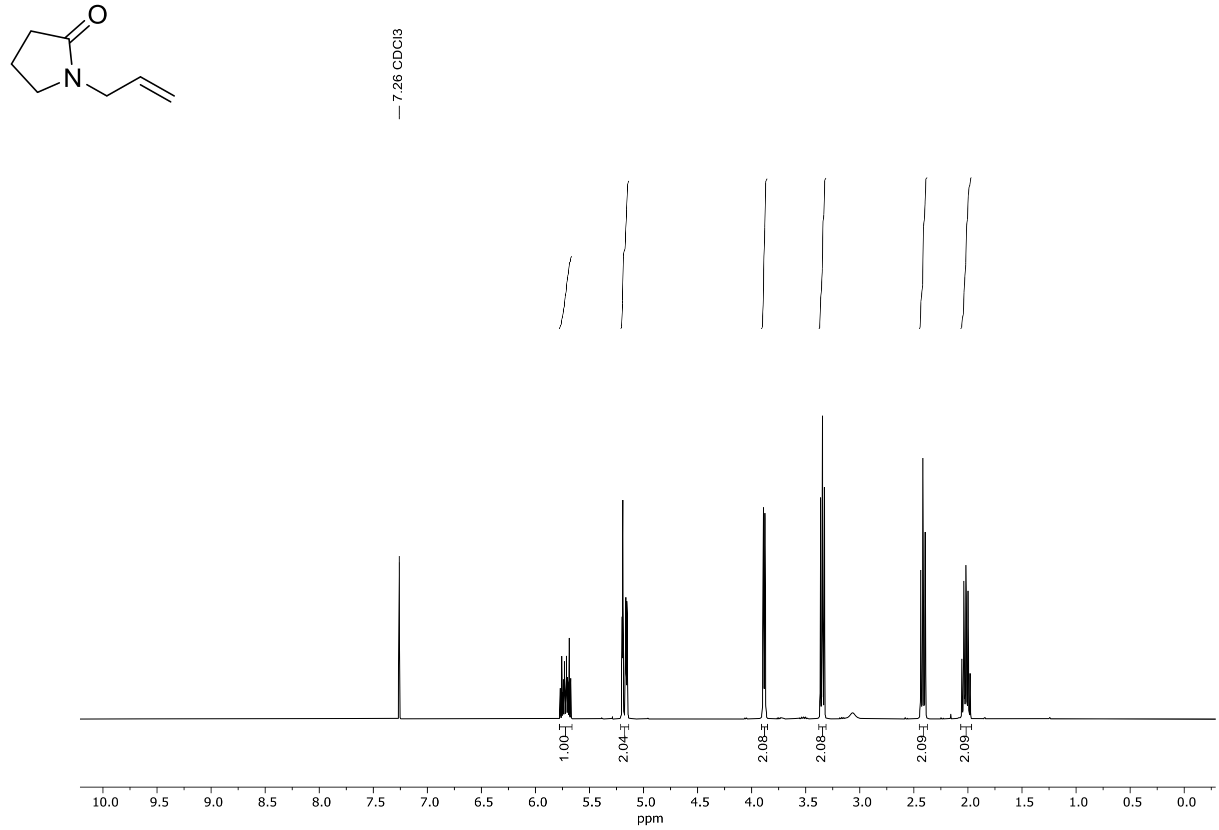

^{13}C-NMR (101MHz)

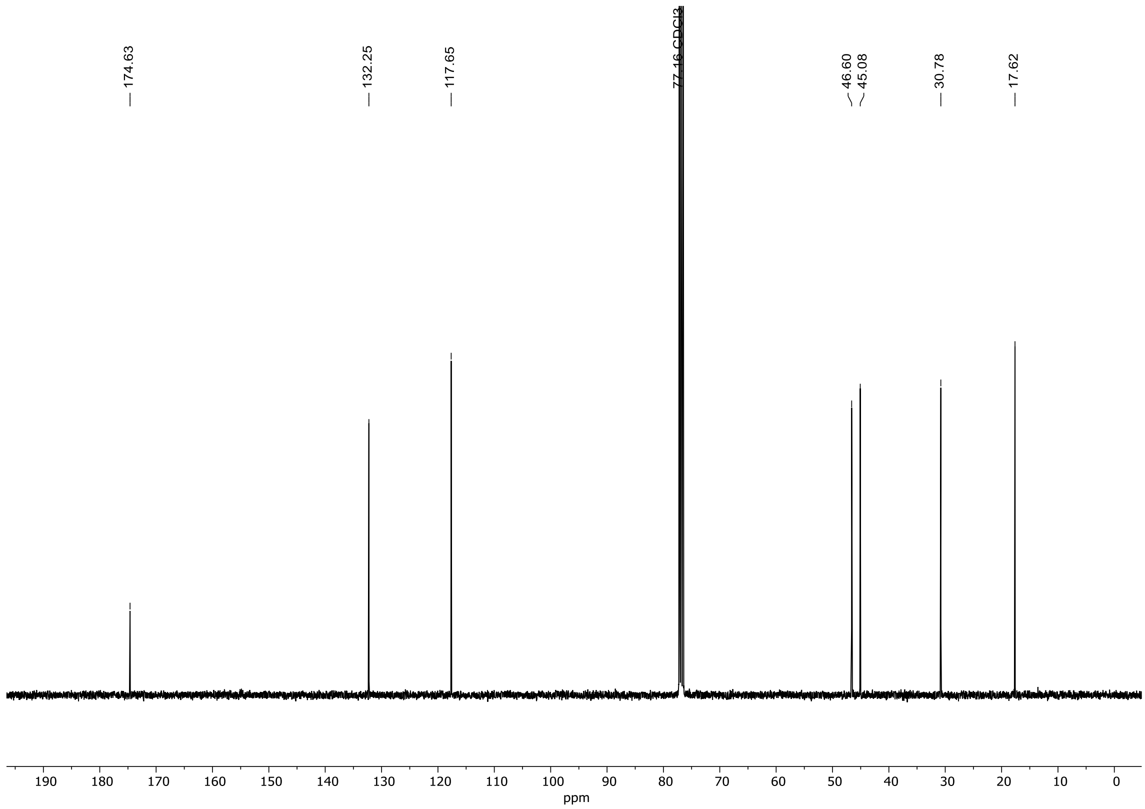

N-(2-Methylallyl)benzamide (1m)

^{1}H-NMR (400MHz)

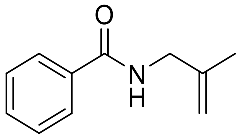

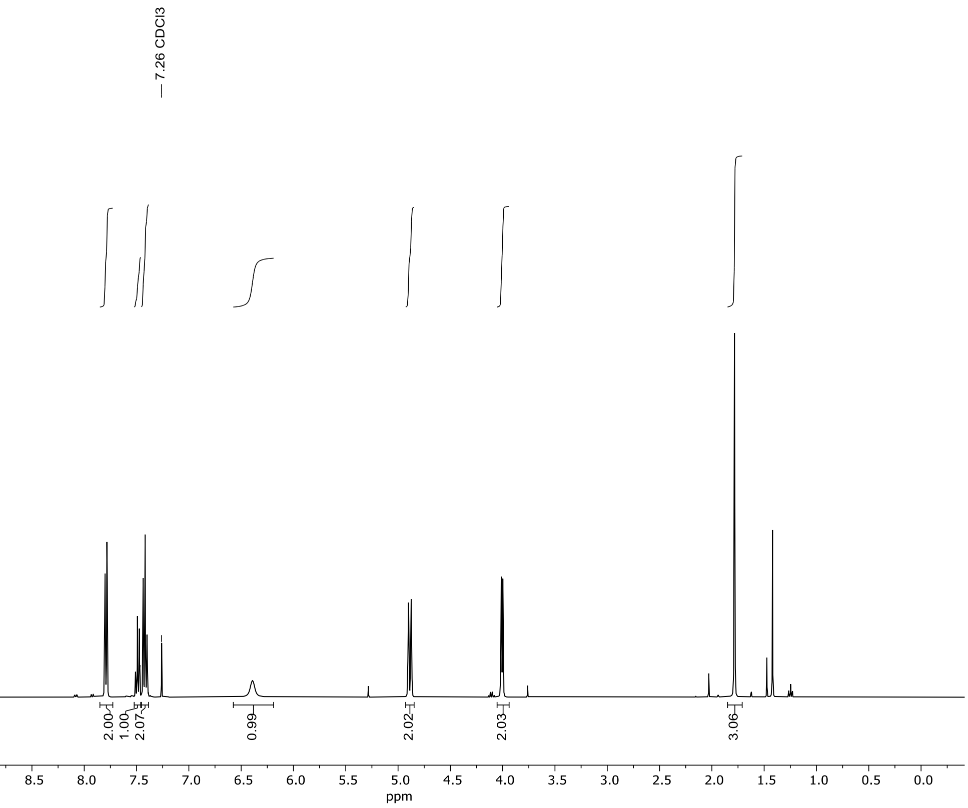

^{13}C-NMR (101MHz)

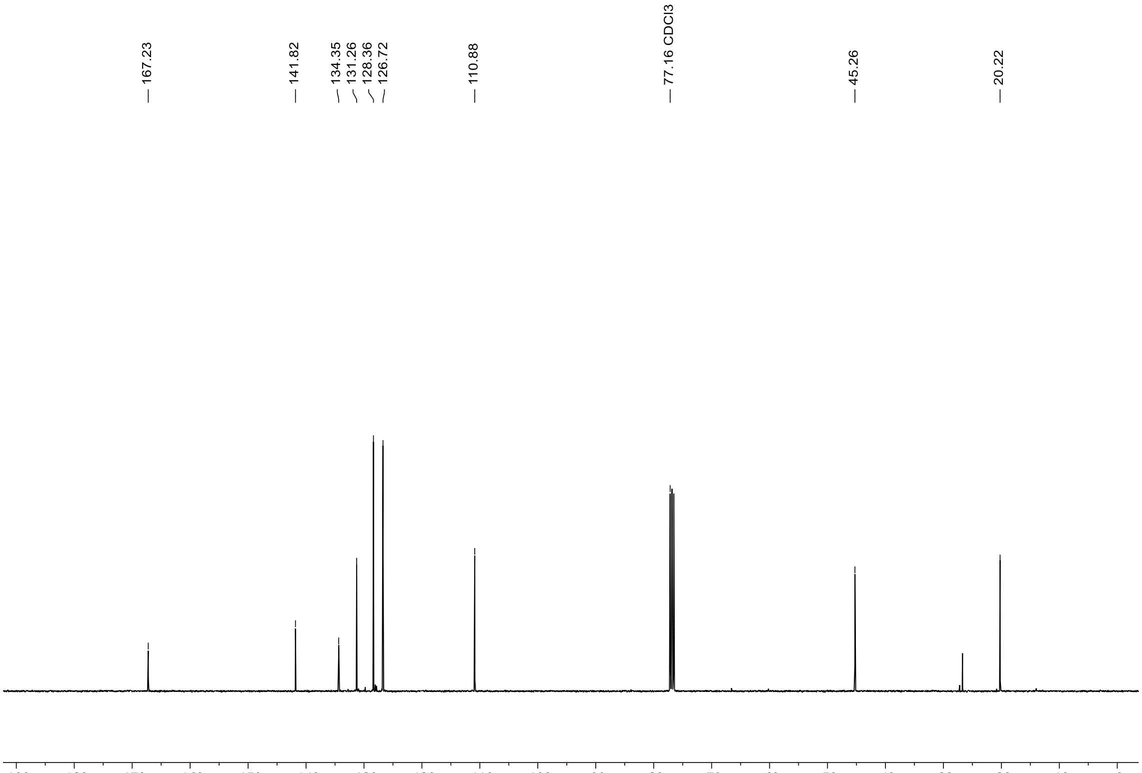

1-(3-Methylbut-2-en-1-yl)pyrrolidin-2-one (1n)

^{1}H-NMR (400MHz)

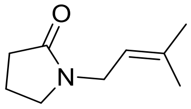

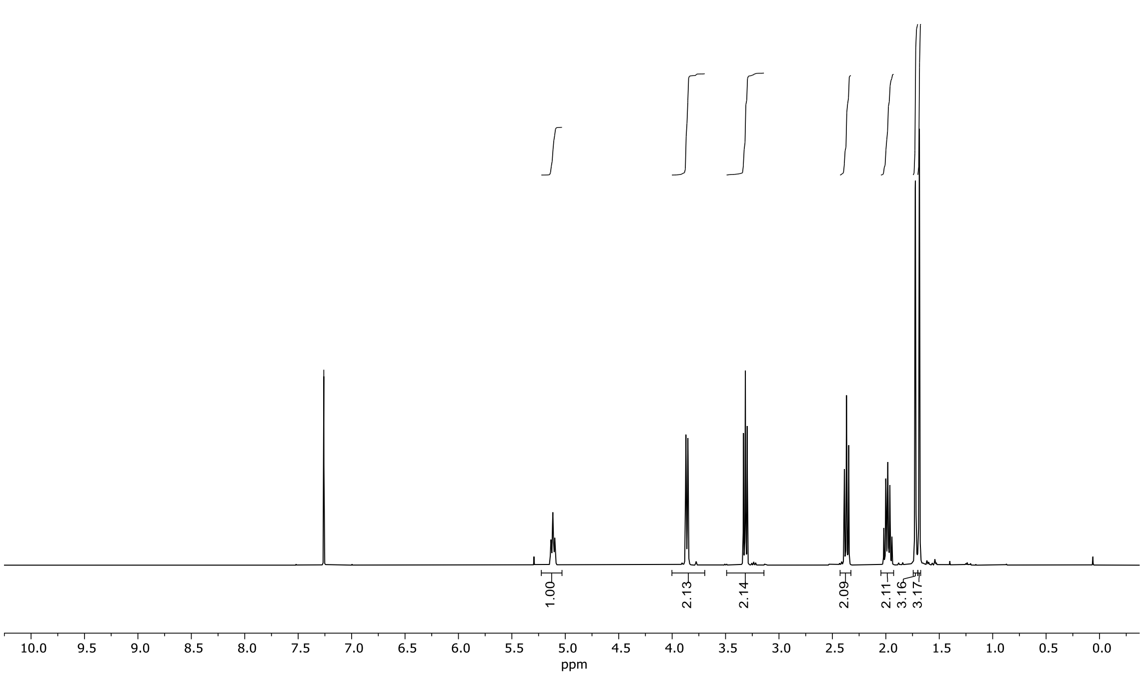

^{13}C-NMR (101MHz)

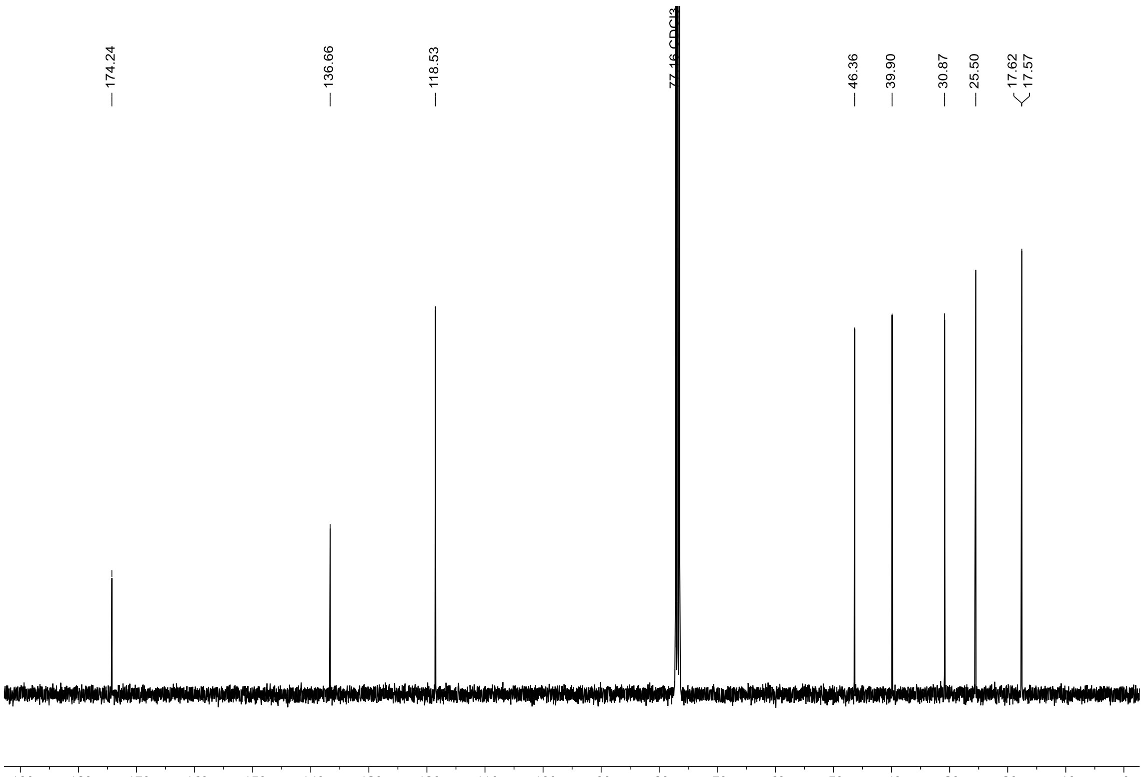

Prop-2-yn-1-yl benzoate (1u)

^{1}H-NMR (400MHz)

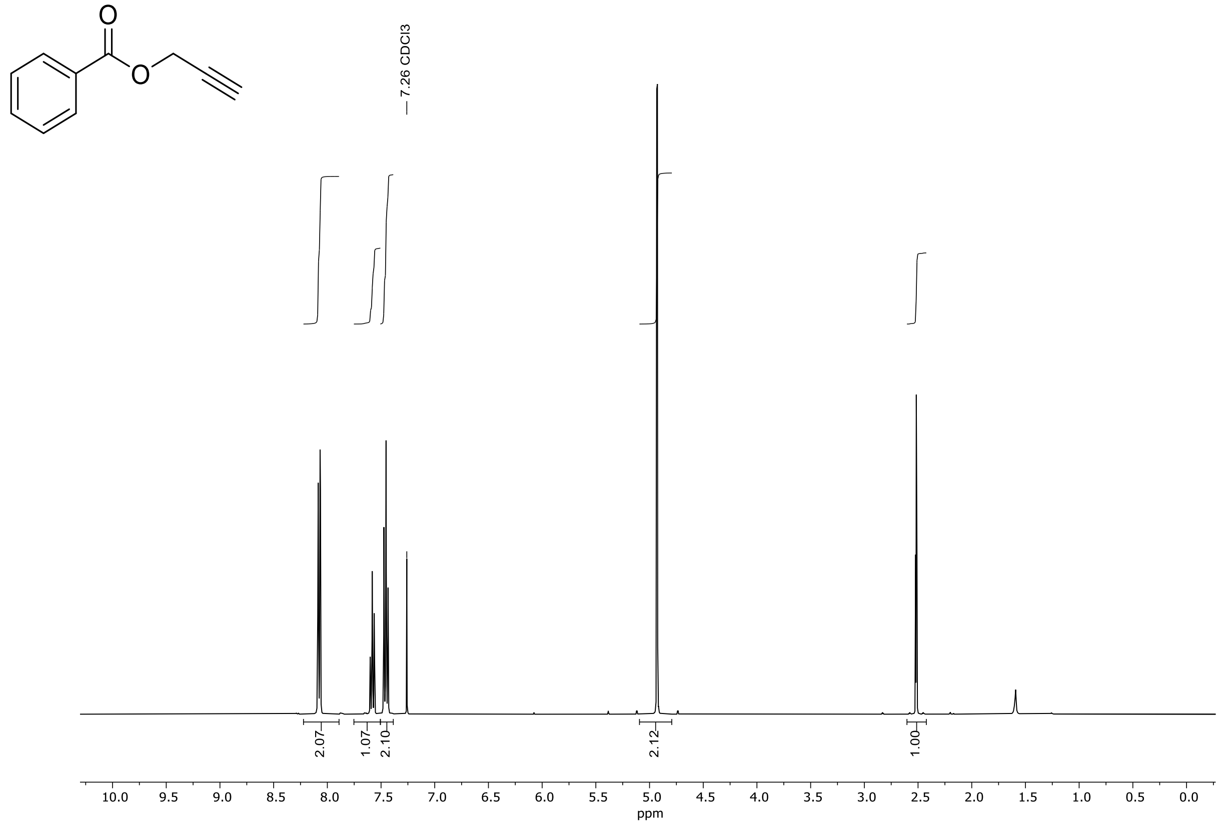

^{13}C-NMR (101MHz)

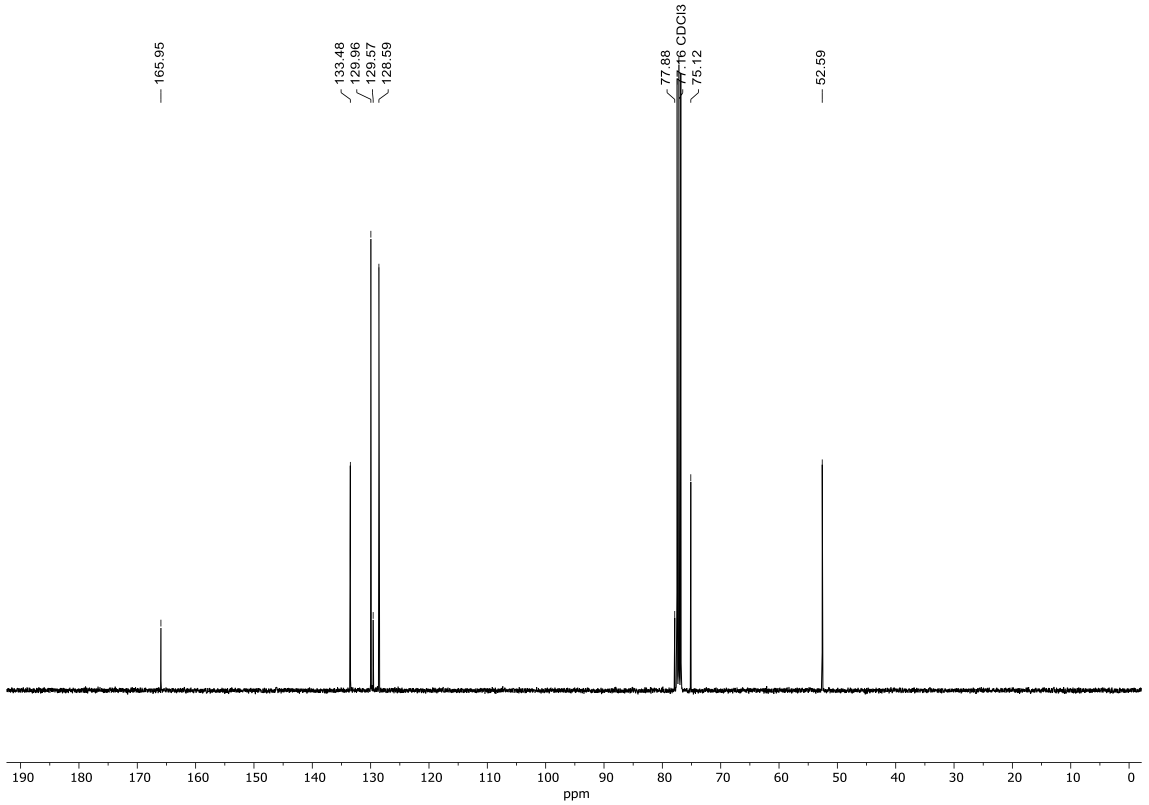

But-3-yn-1-yl benzoate (1v)

^{1}H-NMR (400MHz)

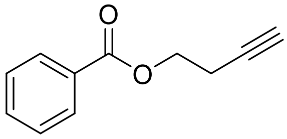

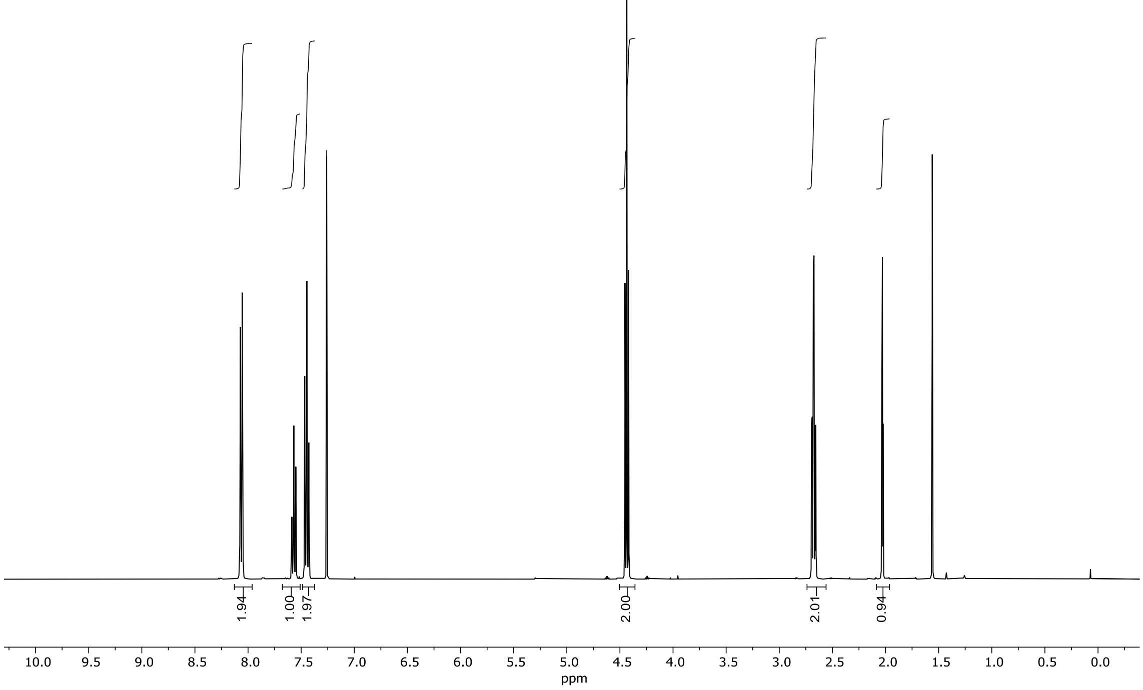

^{13}C-NMR (101MHz)

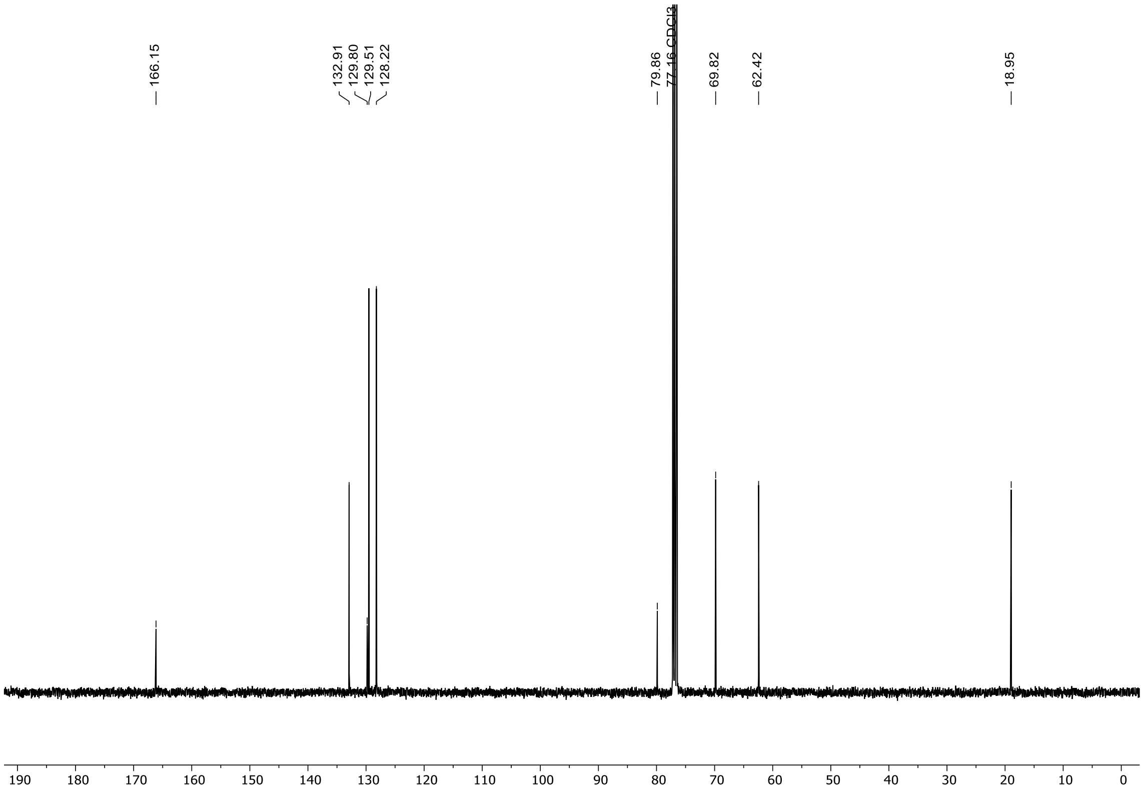

But-2-yn-1-yl benzoate (1w)

^{1}H-NMR (400MHz)

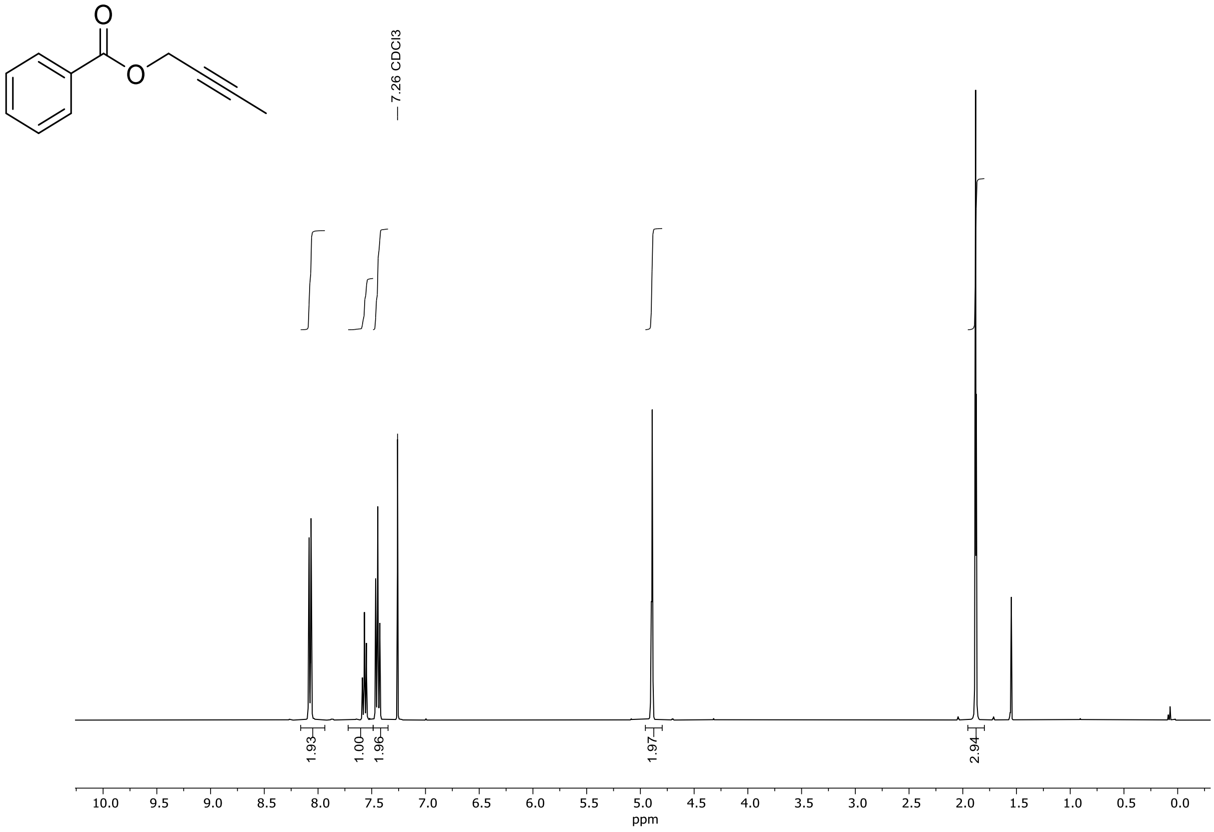

^{13}C-NMR (101MHz)

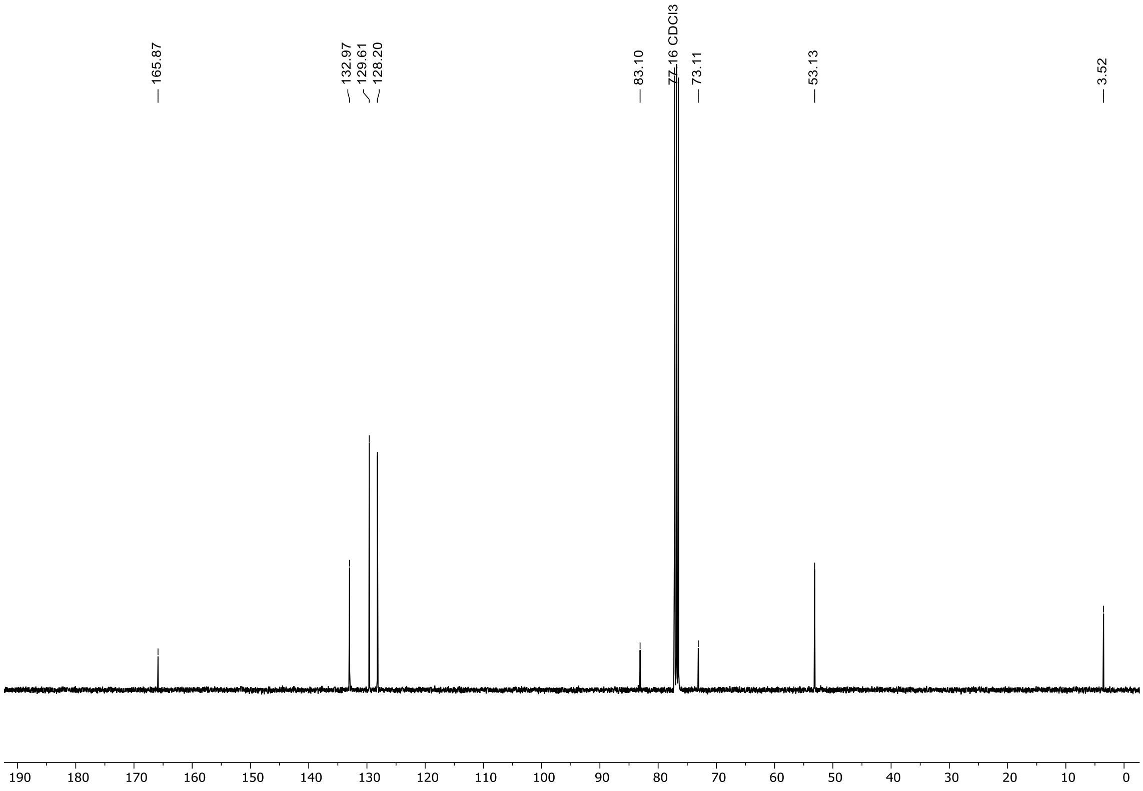

3-Phenylprop-2-yn-1-yl benzoate (1x)

^{1}H-NMR (400MHz)

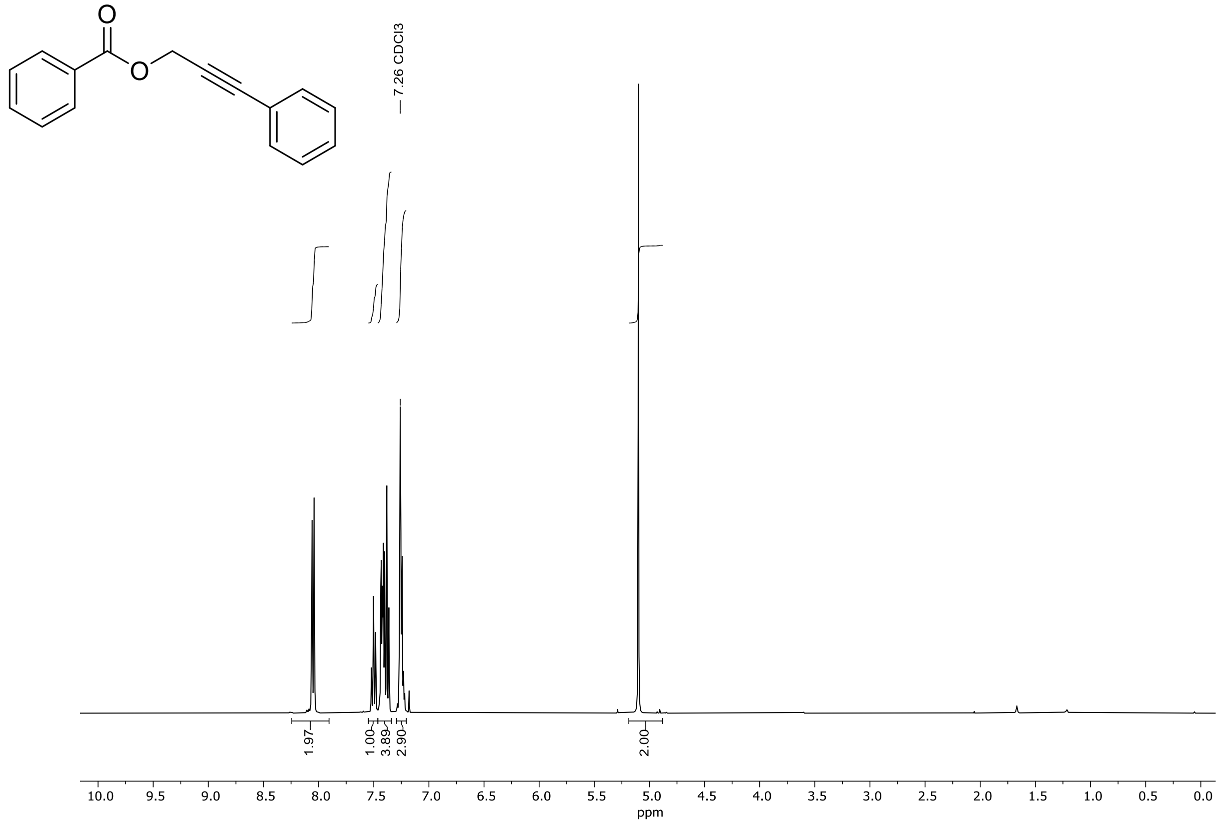

^{13}C-NMR (101MHz)

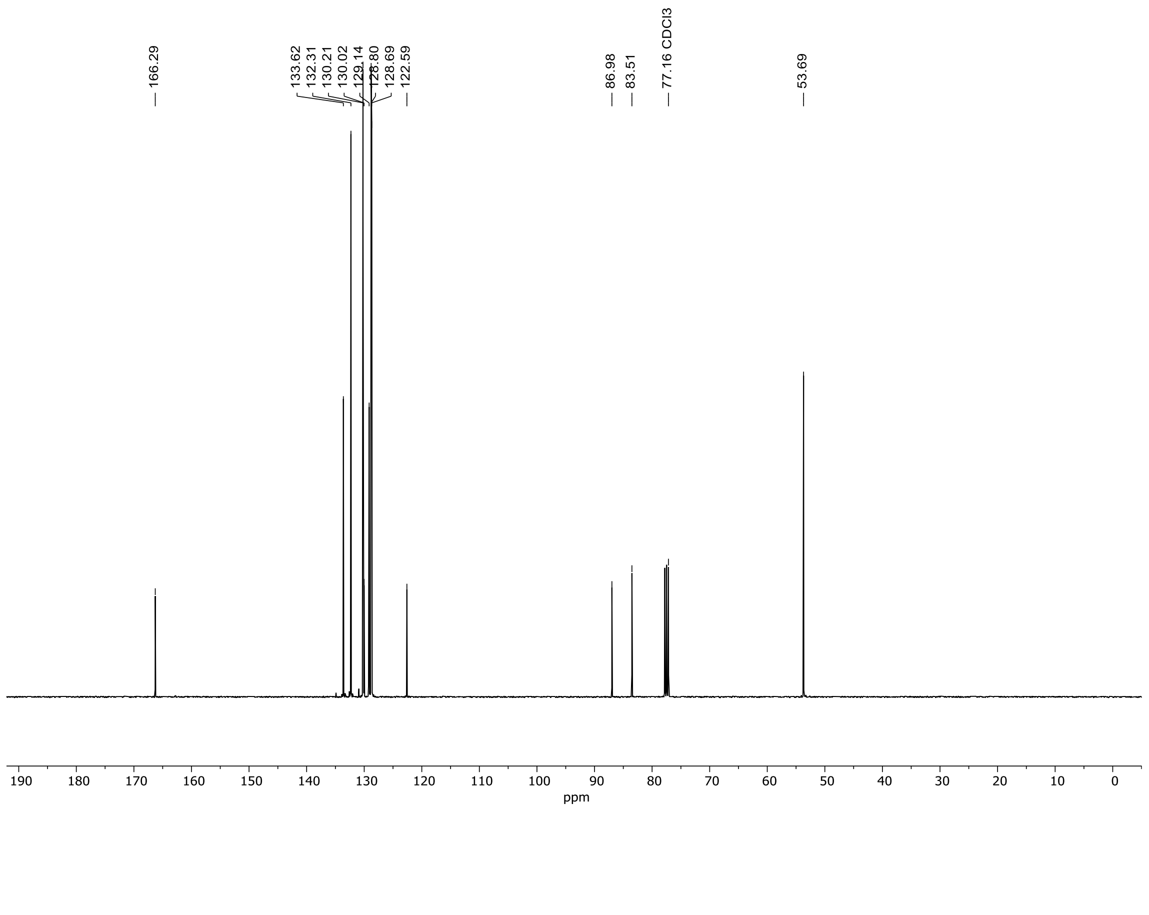

N-(Prop-2-yn-1-yl)benzamide (1y)

^{1}H-NMR (400MHz)

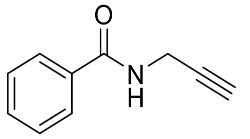

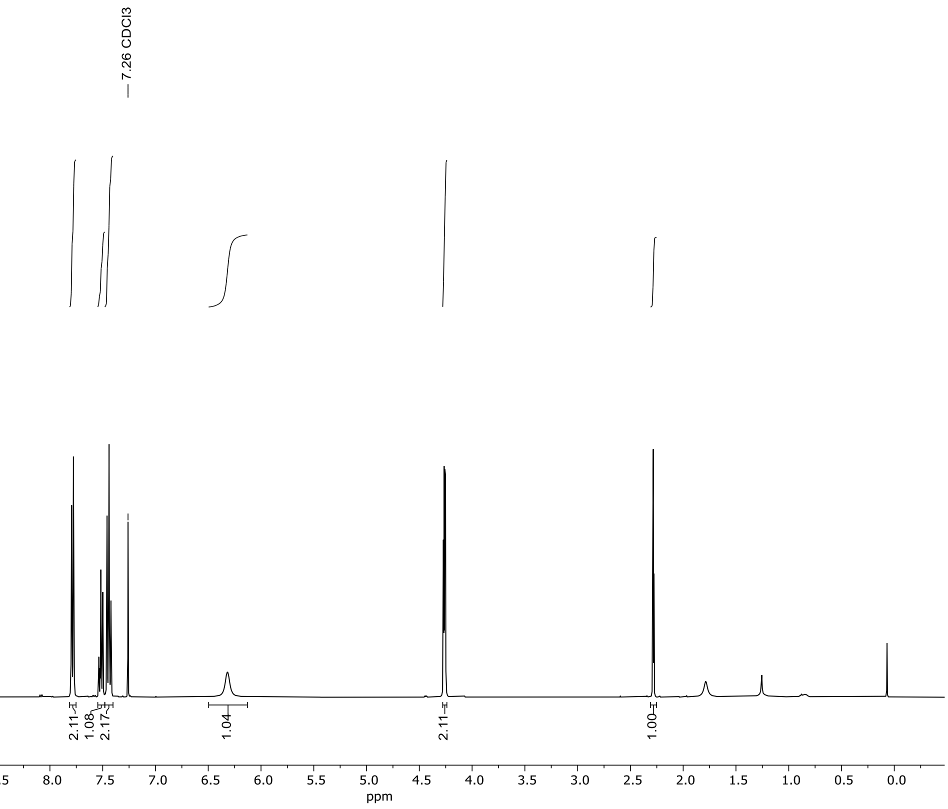

^{13}C-NMR (101MHz)

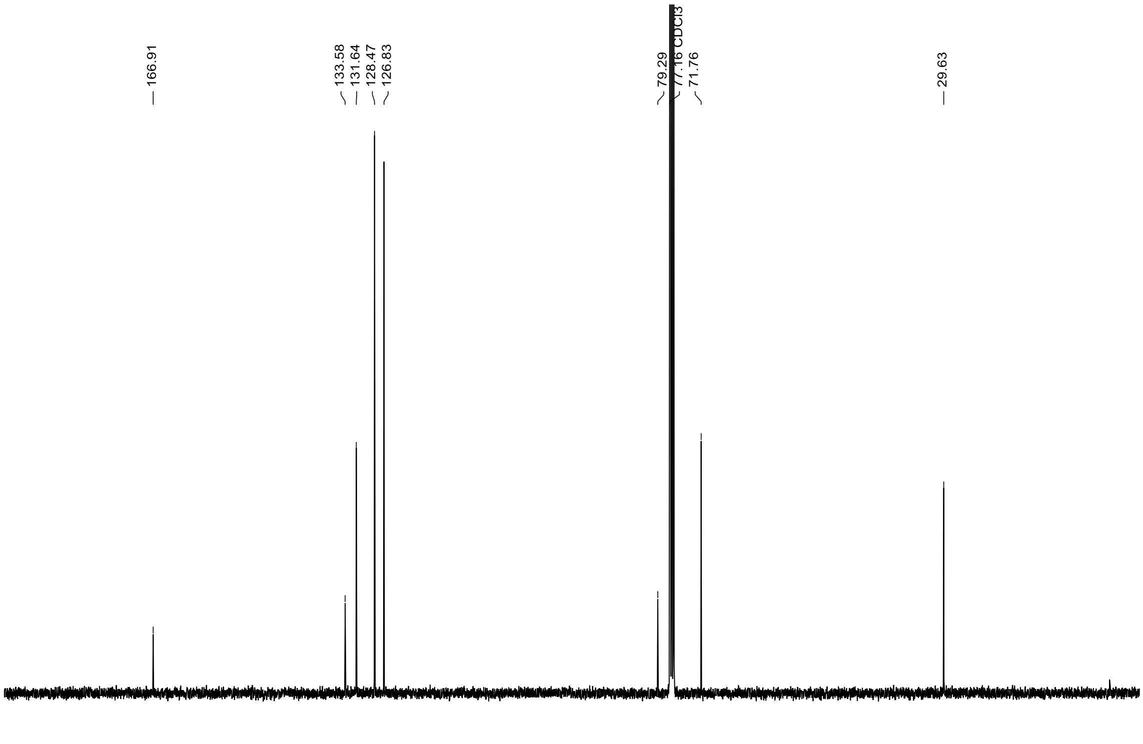

N-(But-3-yn-1-yl)benzamide (1z)

^{1}H-NMR (400MHz)

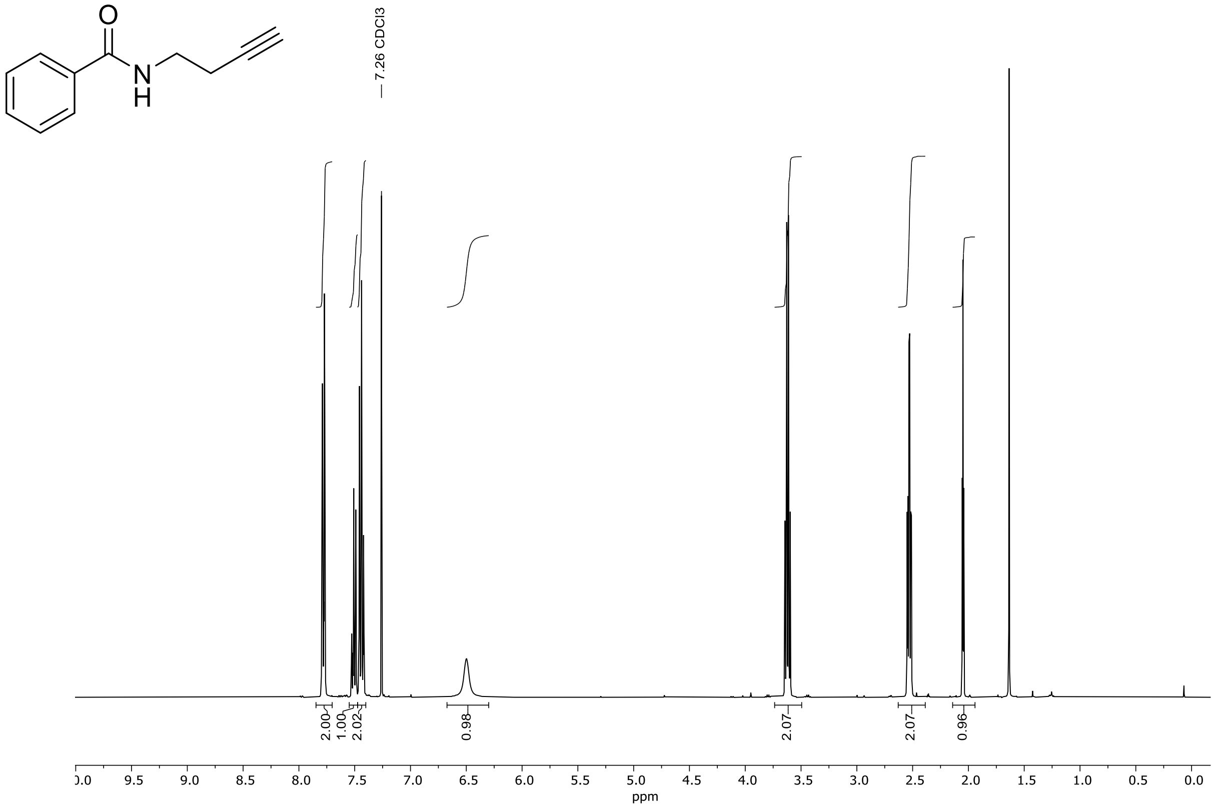

^{13}C-NMR (101MHz)

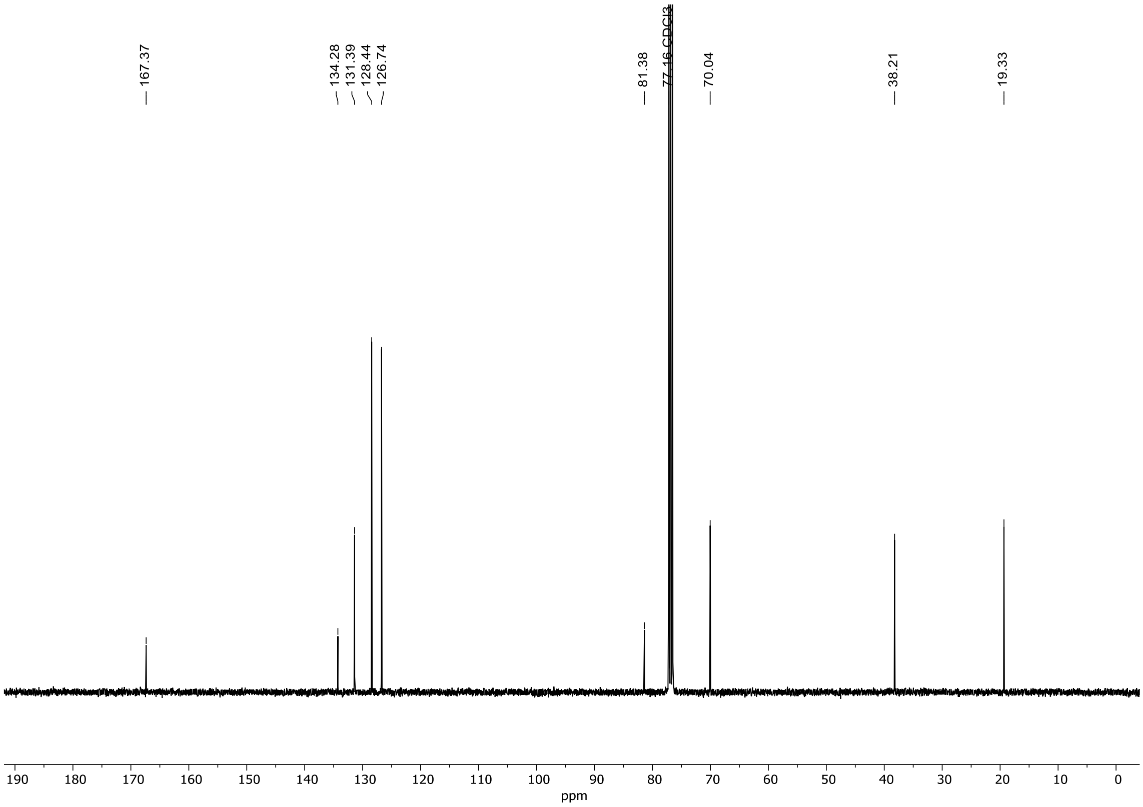

4-Methyl-N-(prop-2-yn-1-yl)benzenesulfonamide (1aa)

^{1}H-NMR (400MHz)

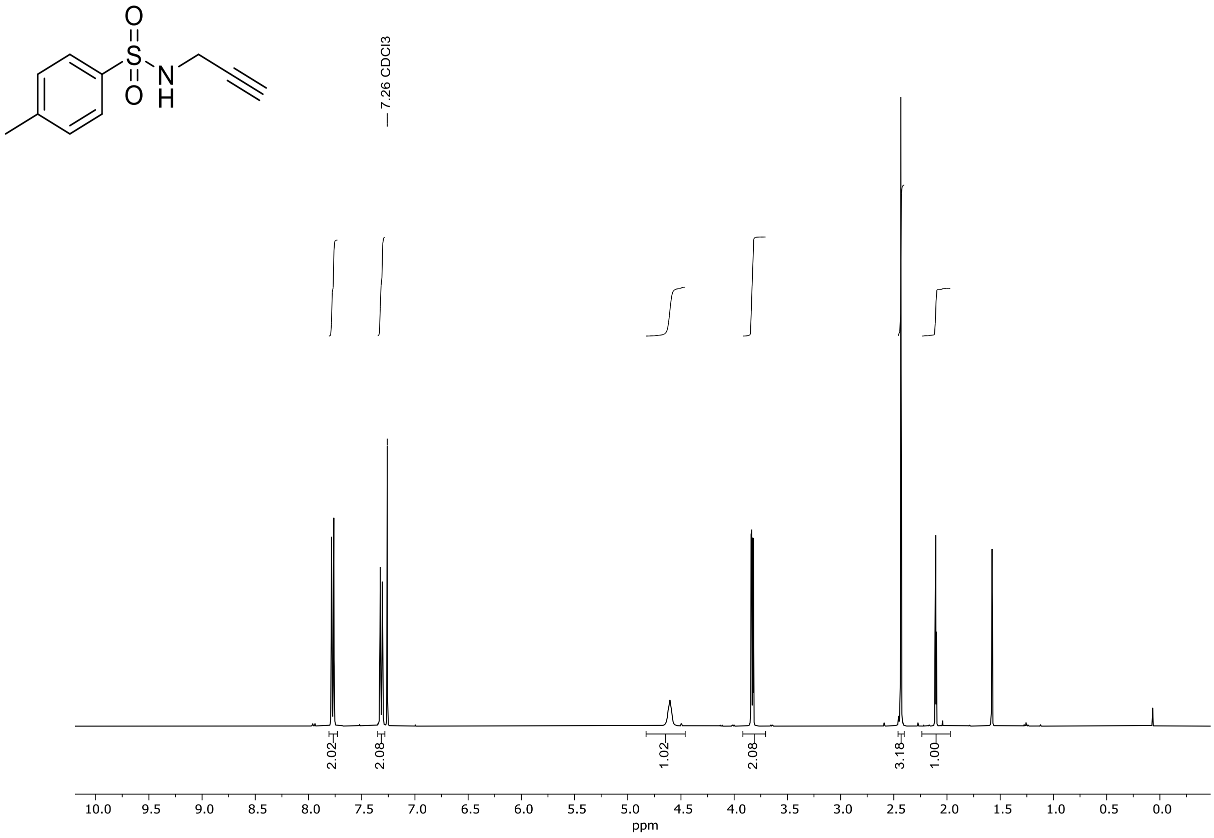

^{13}C-NMR (101MHz)

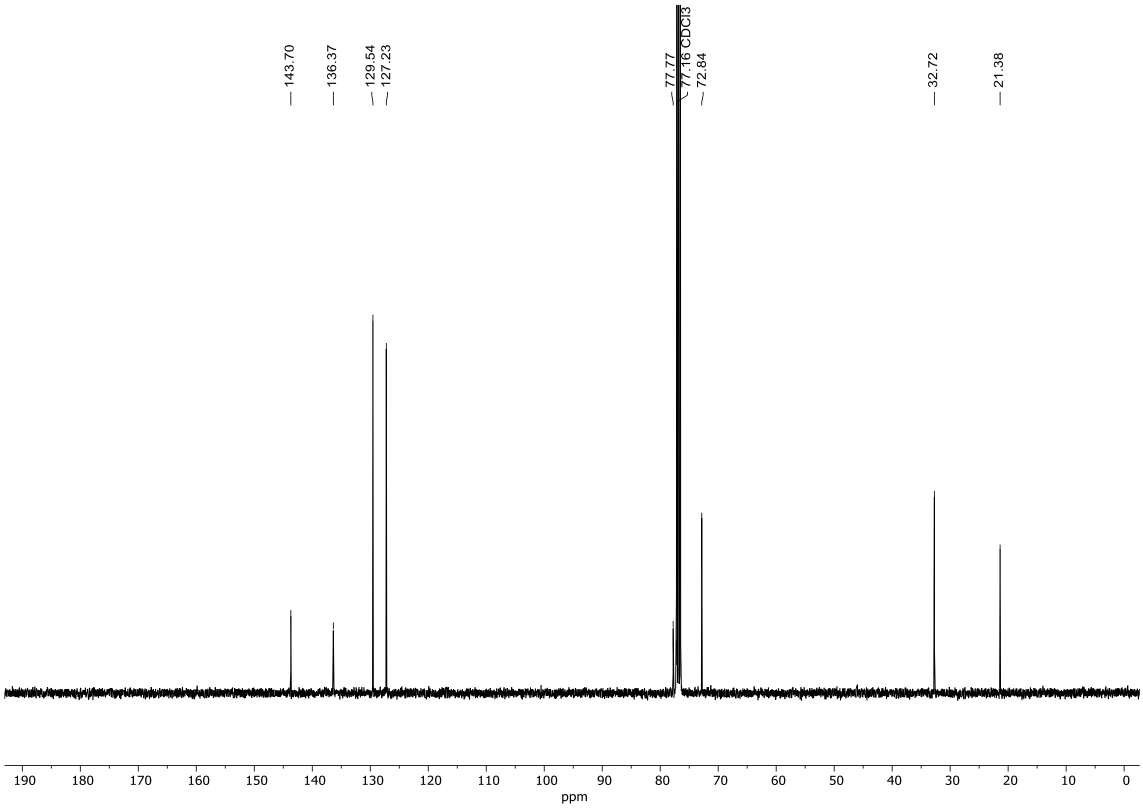

2-(Allyloxy)-1-isopropyl-4-methylcyclohexane (1ac)

^{1}H-NMR (400MHz)

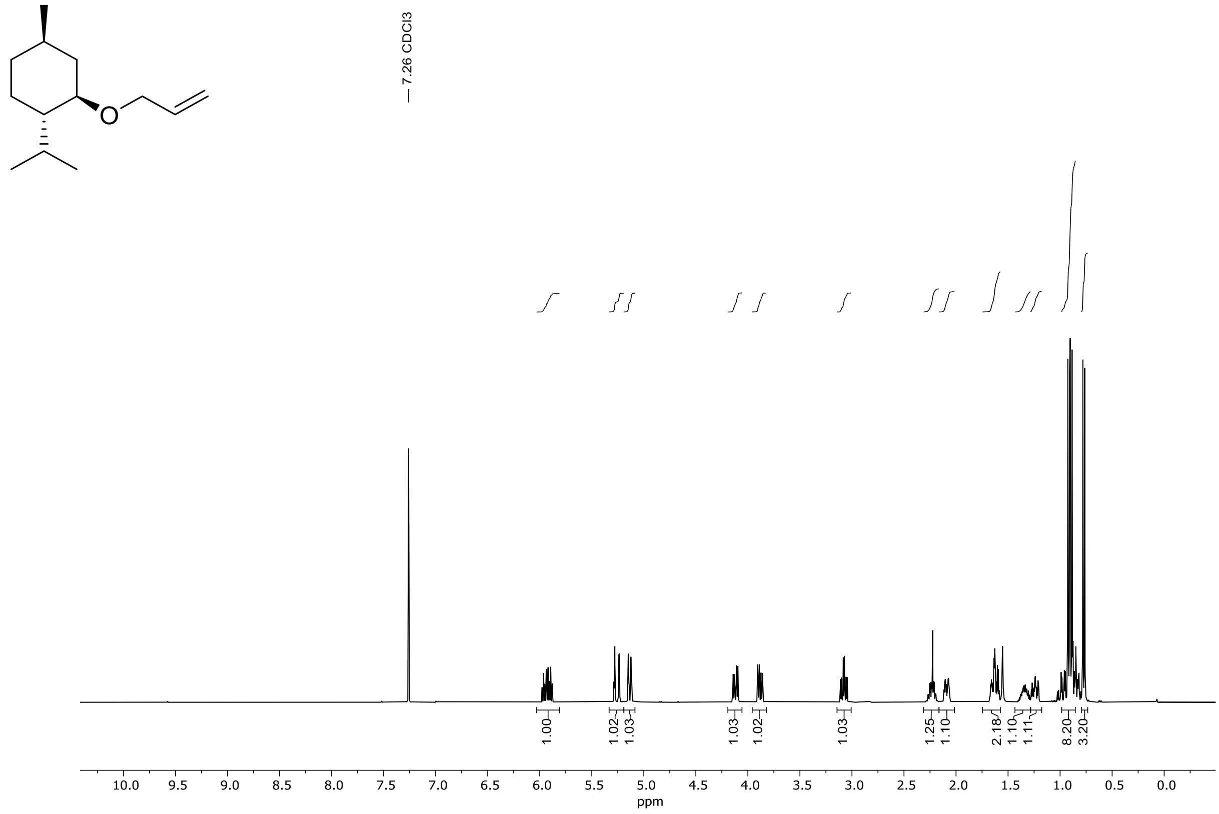

^{13}C-NMR (101MHz)

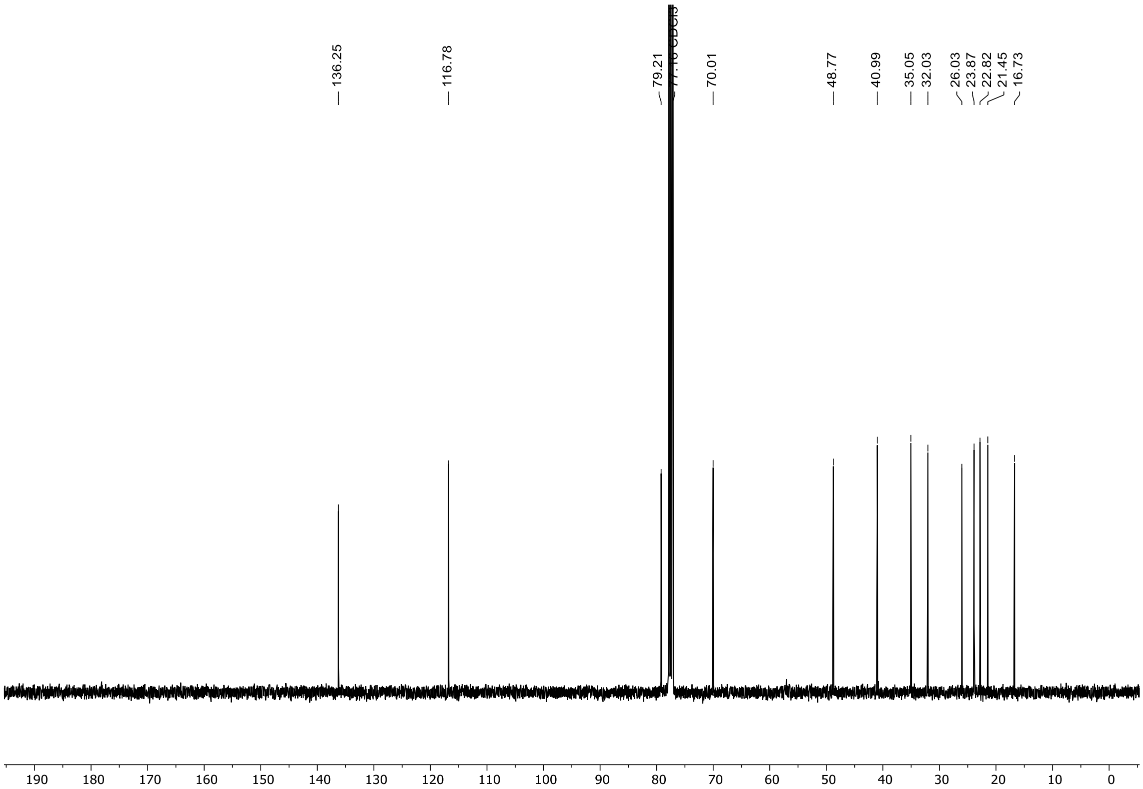

3,7-Dimethyloct-6-en-1-yl benzoate (1ad)

^{1}H-NMR (400MHz)

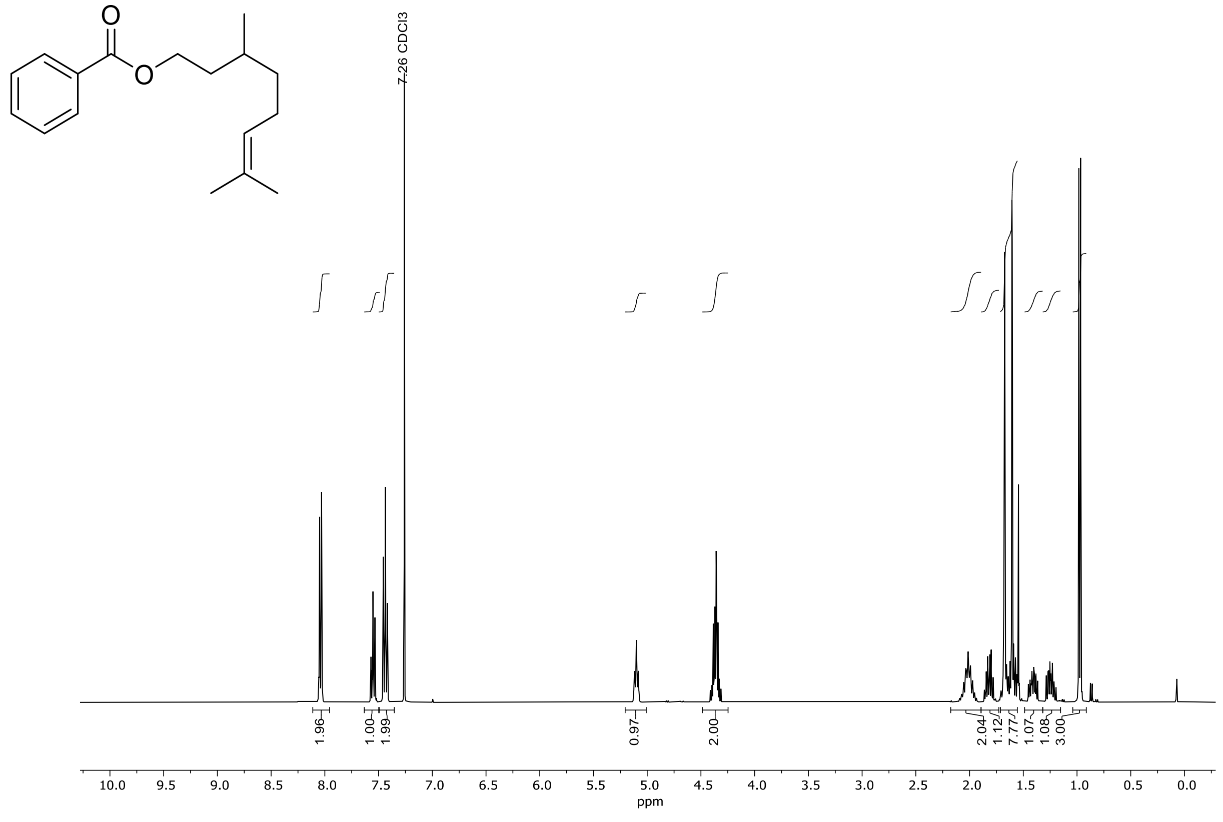

^{13}C-NMR (101MHz)

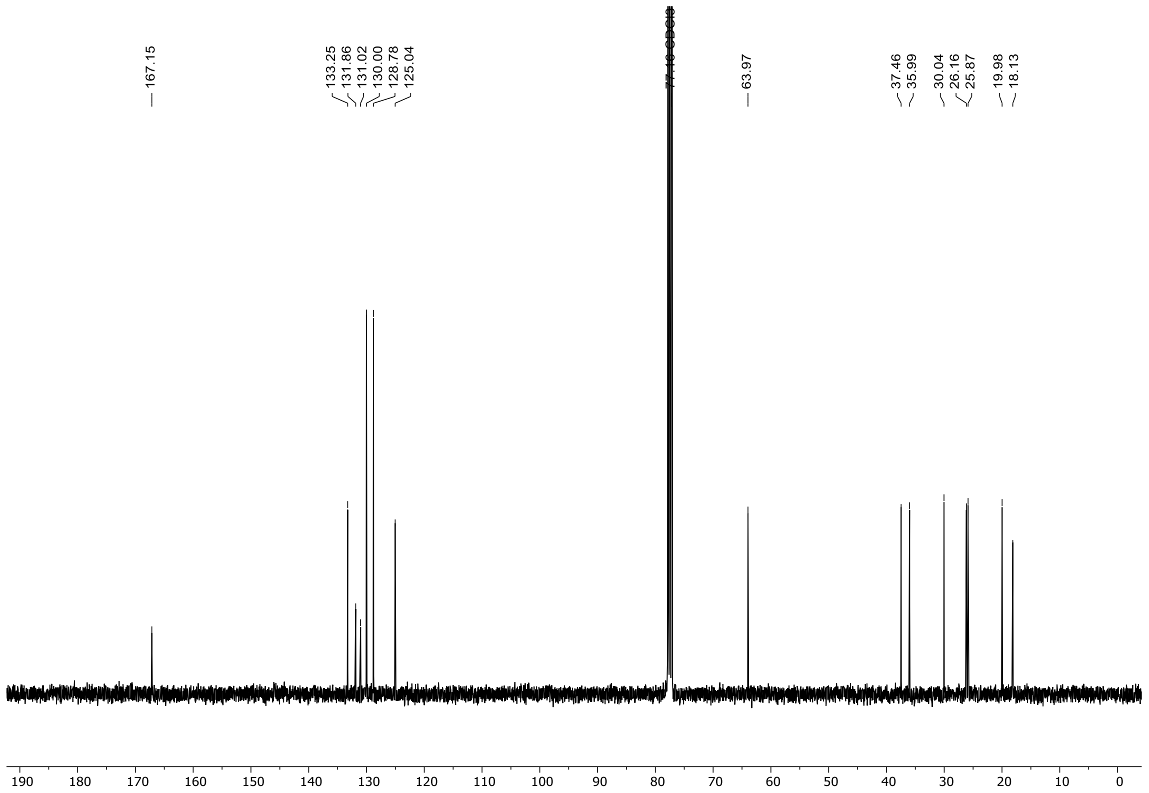

Pent-4-en-1yl 4-([1,1'-biphenyl]-4-yl)-4-oxobutanoate (1ae)

^{1}H-NMR (400MHz)

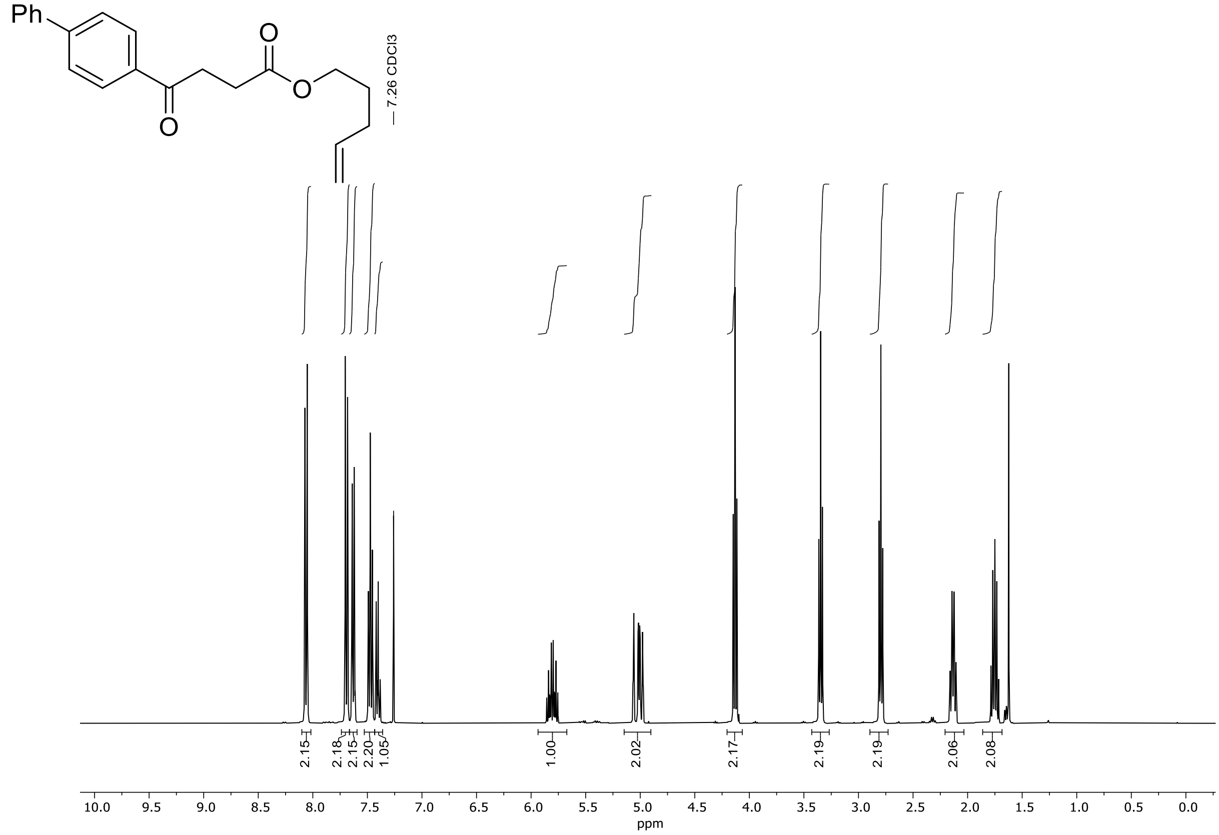

^{13}C-NMR (101MHz)

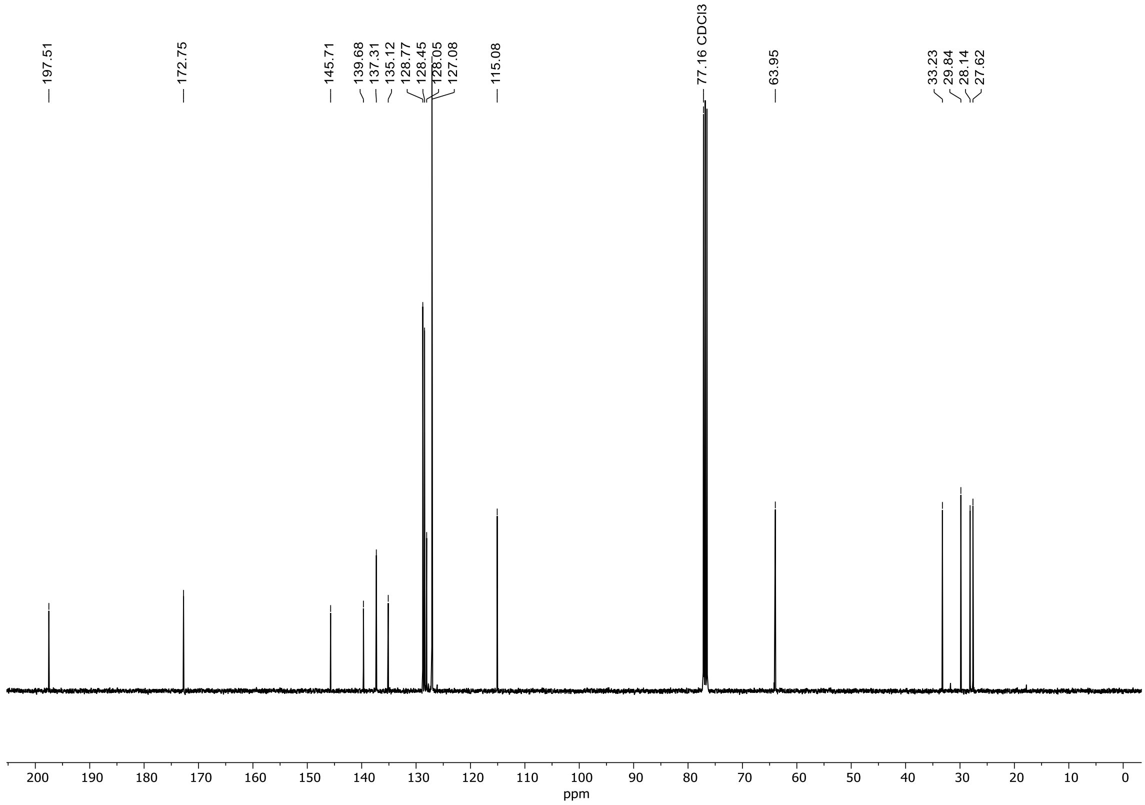

Pent-4-en-1-yl 2-(4-isobutylphenyl)propanoate (1af)

^{1}H-NMR (400MHz)

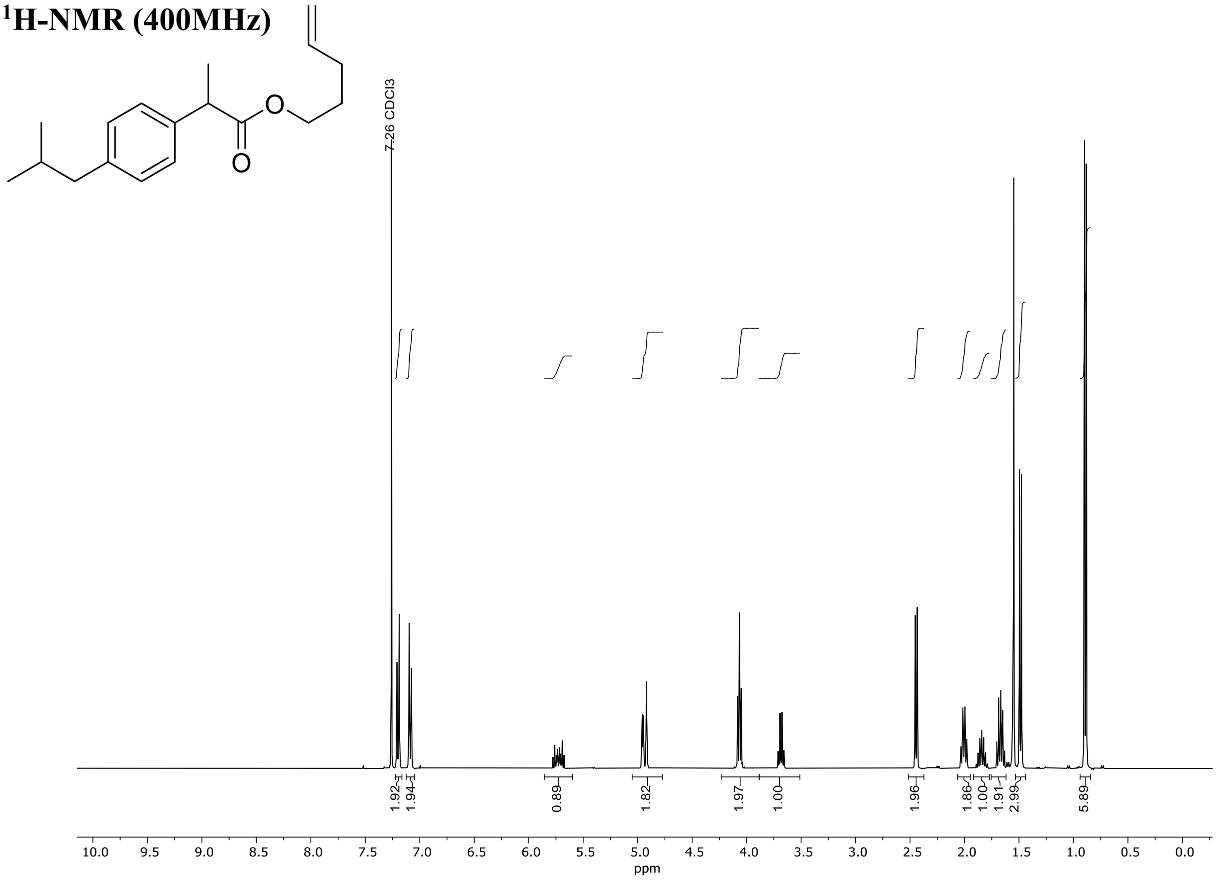

^{13}C-NMR (101MHz)

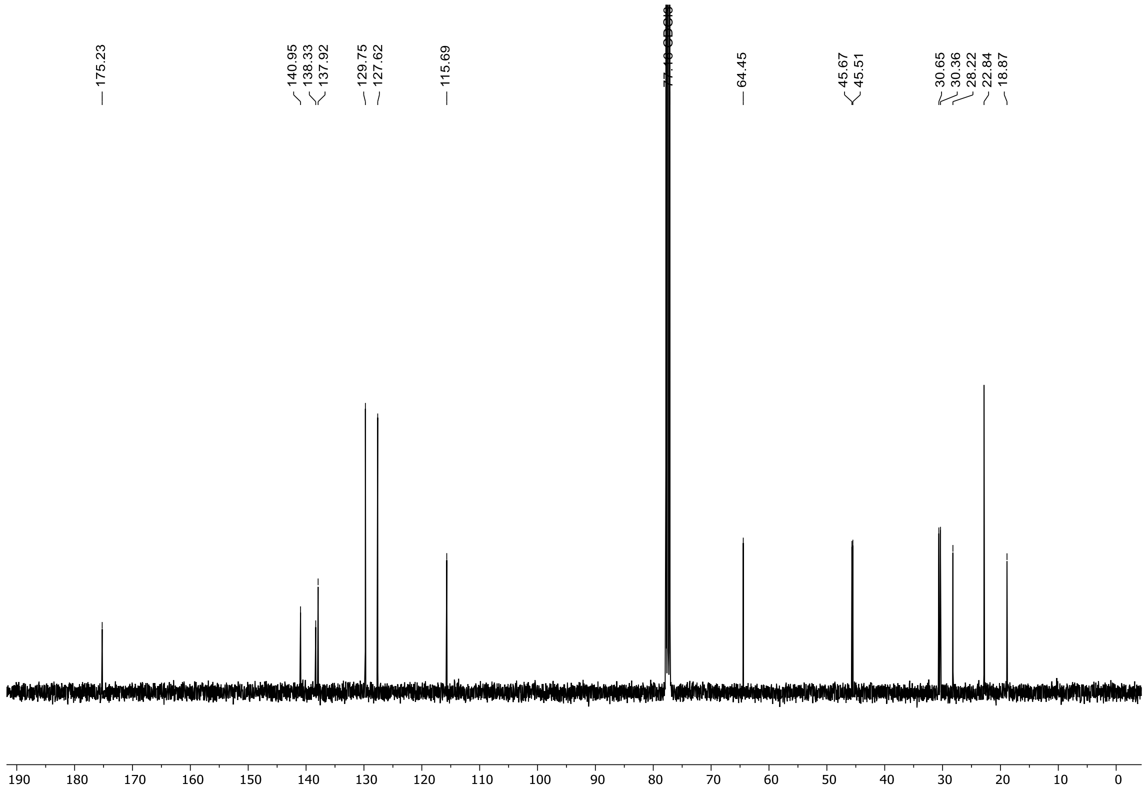

3-Methylbut-2-en-1-yl acetyl-*L*-phenylalaninate (1ag)

^{1}H-NMR (400MHz)

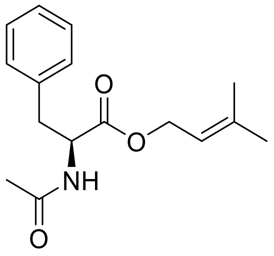

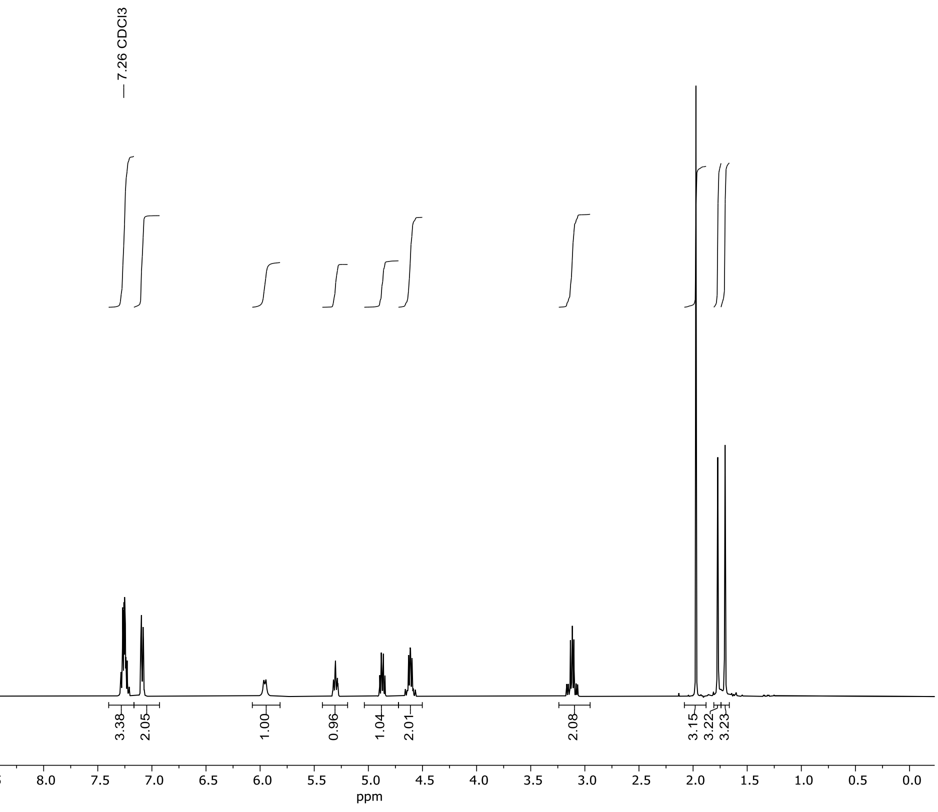

^{13}C-NMR (101MHz)

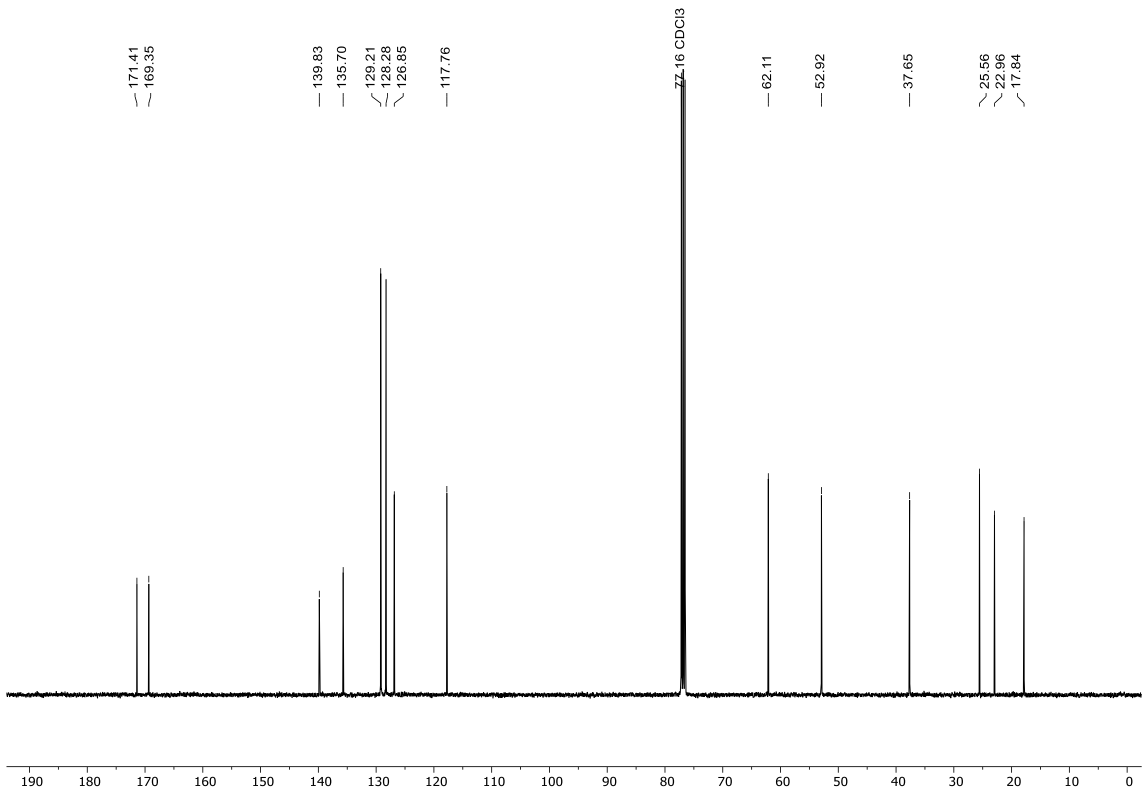

N,*N*-Diallyl 4-methylbenzenesulfonamide (1ah)

^{1}H-NMR (400MHz)

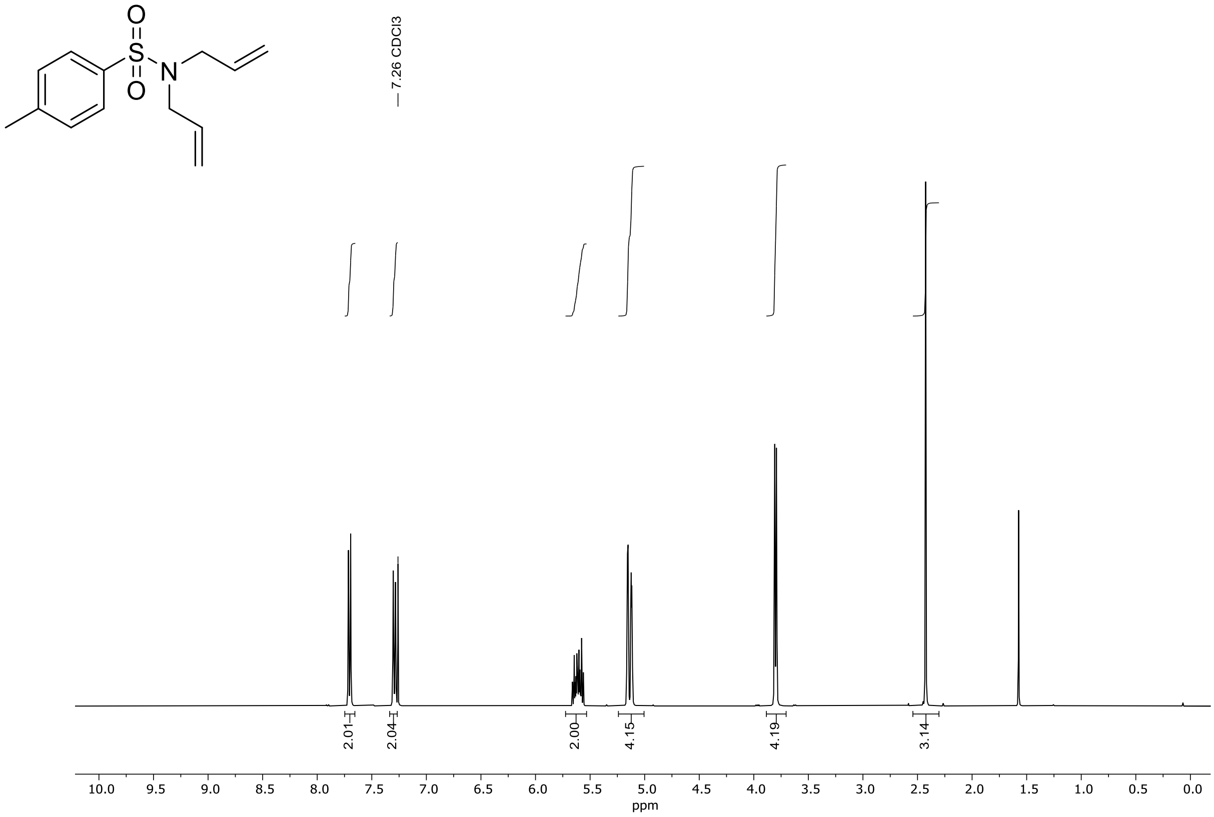

^{13}C-NMR (101MHz)

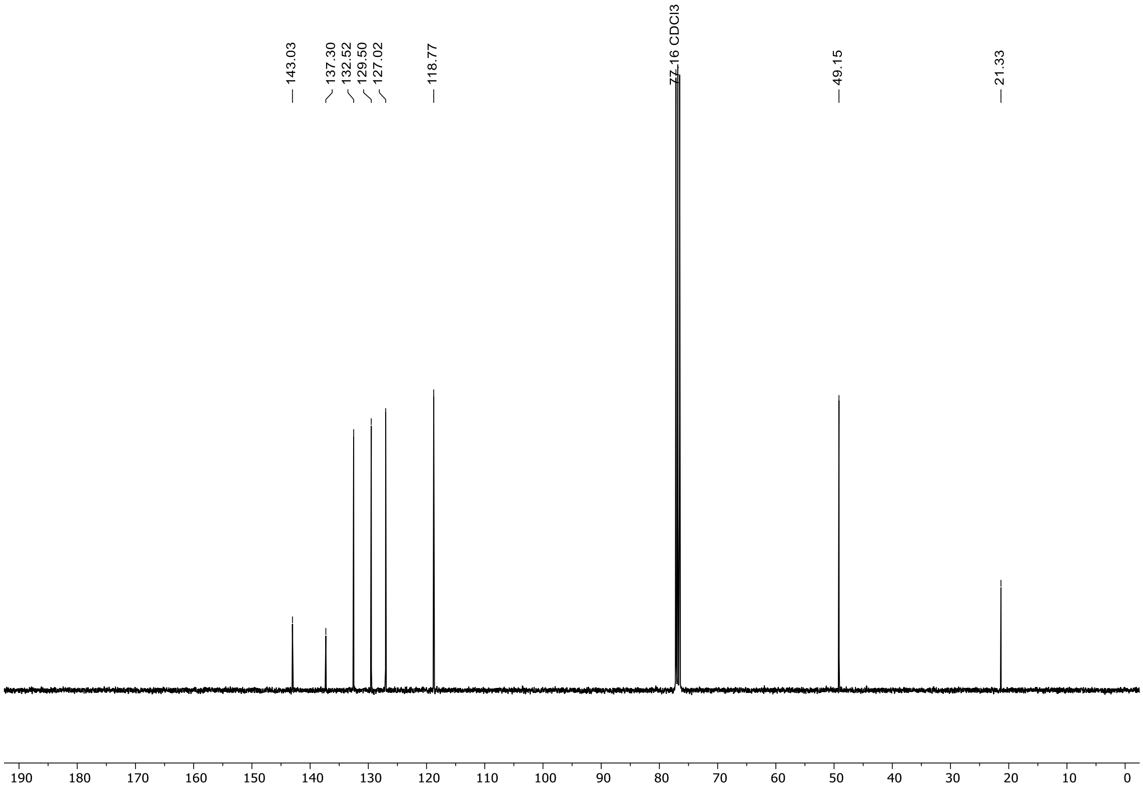

4-Bromo-6,6,6-trichlorohexyl benzoate (3)

^{1}H-NMR (400MHz)

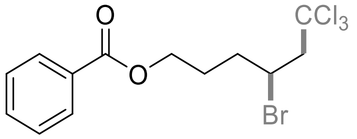

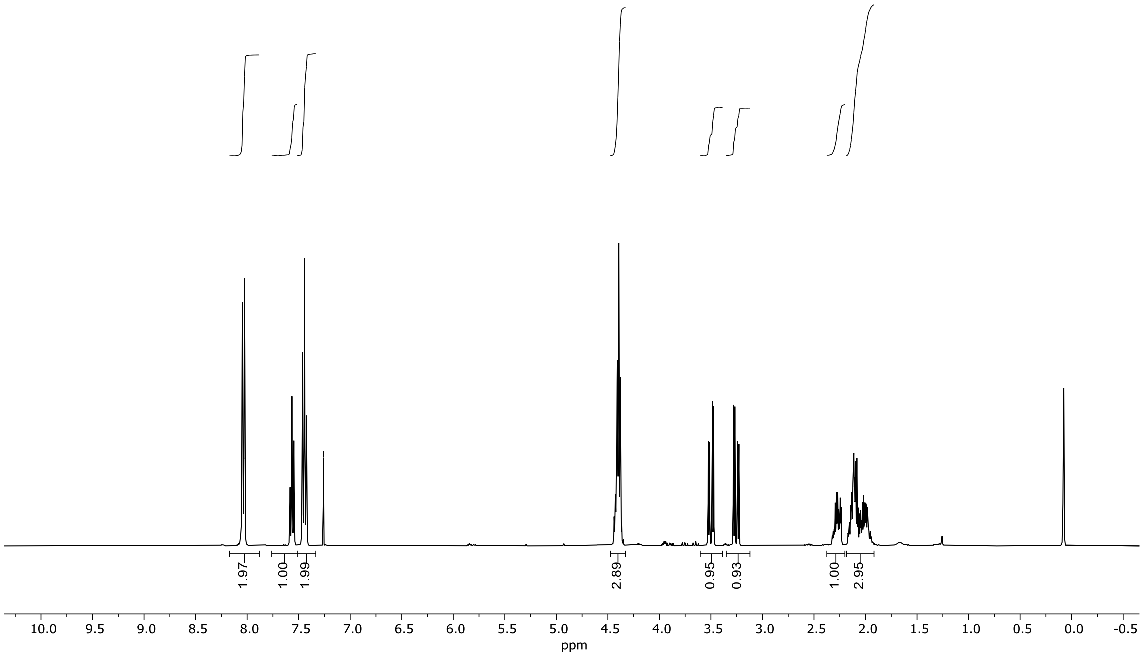

^{13}C-NMR (101MHz)

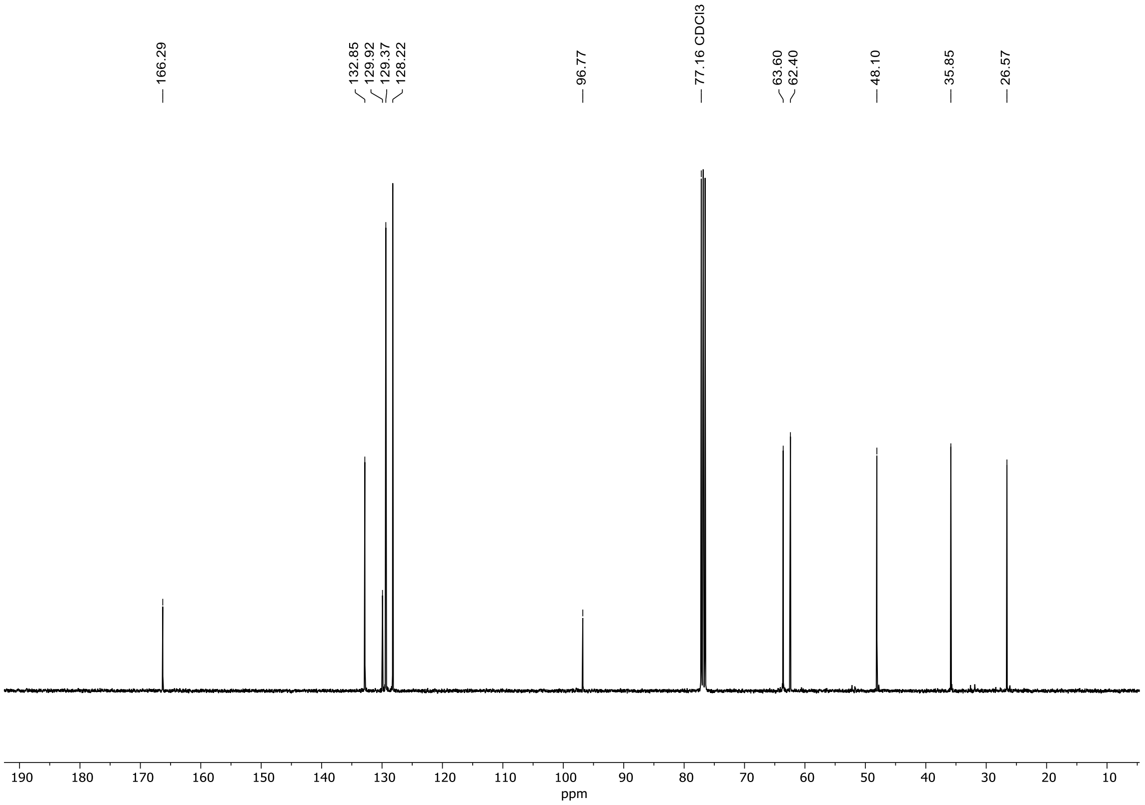

4,6,6,6-Tetrabromohexyl benzoate (4)

^{1}H-NMR (400MHz)

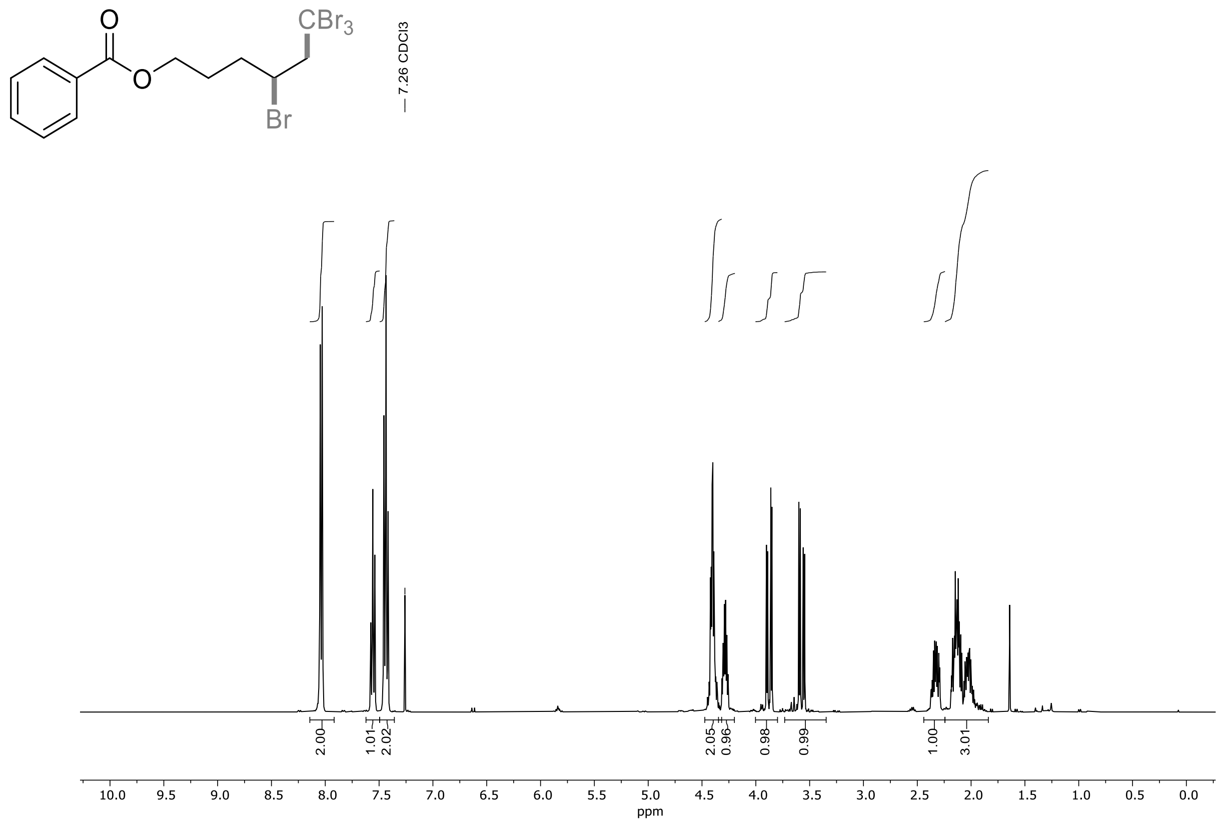

^{13}C-NMR (101MHz)

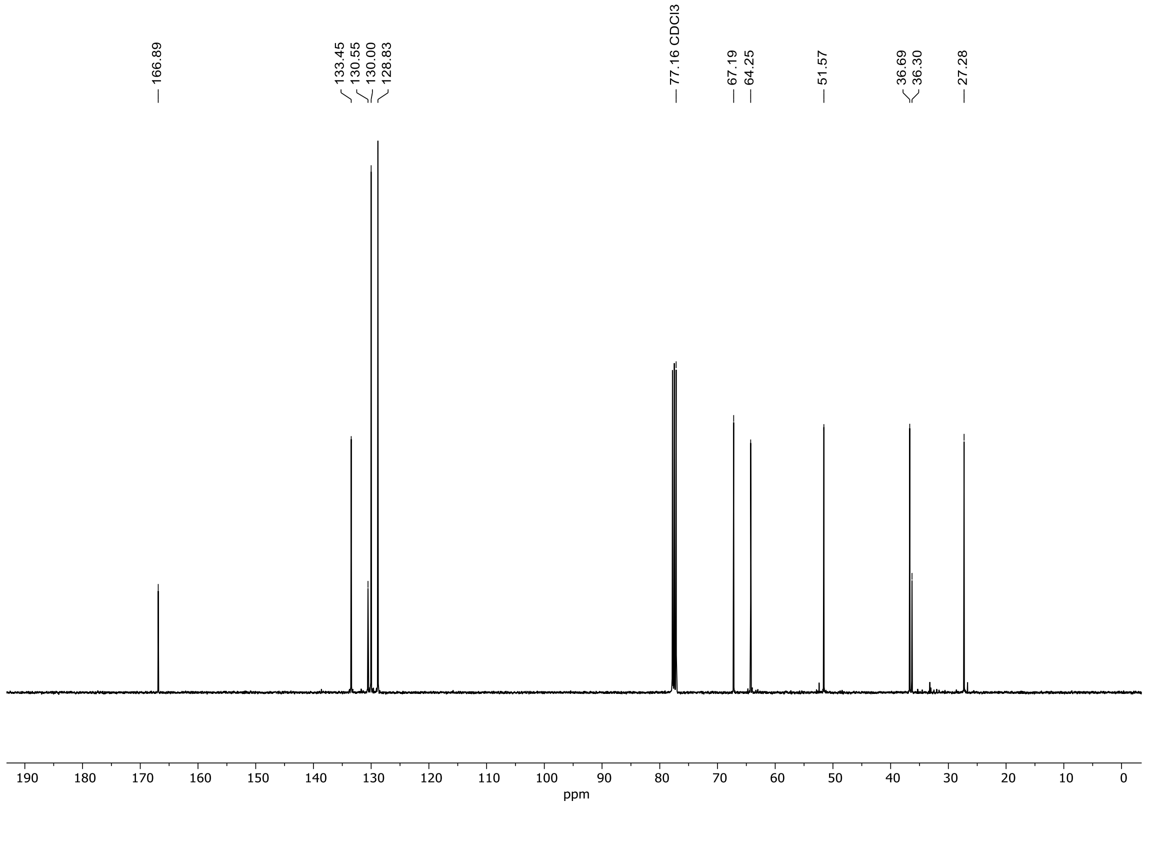

4-Bromo-6-cyanohexyl benzoate (5)

^{1}H-NMR (400MHz)

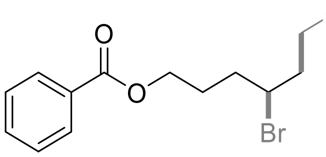

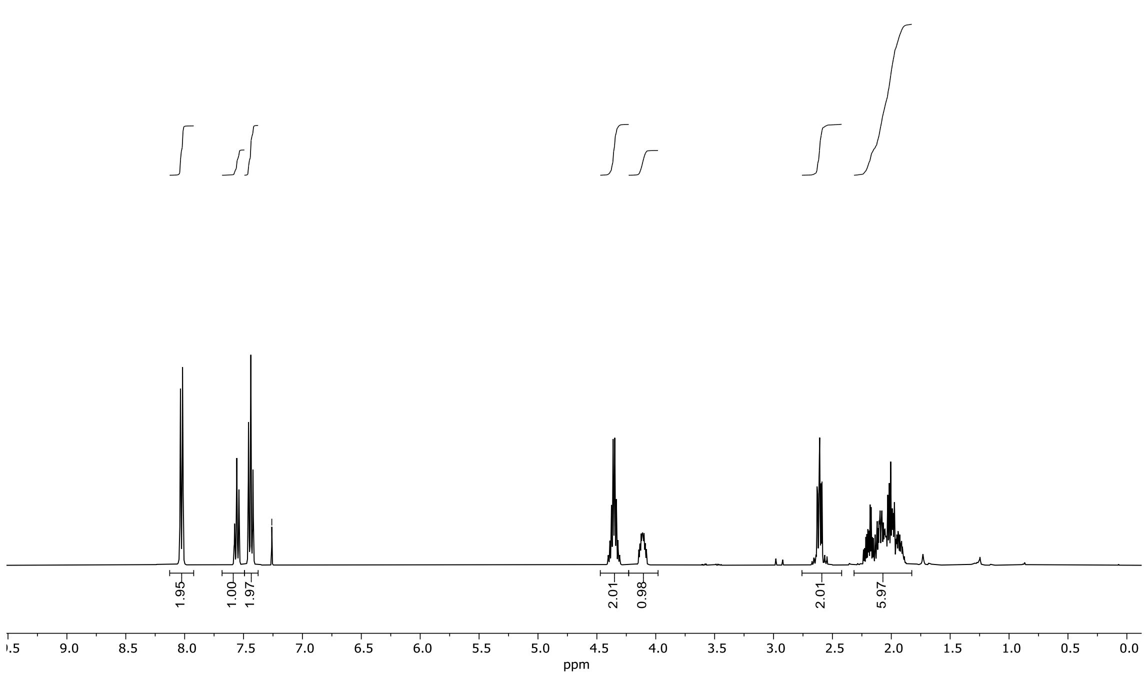

^{13}C-NMR (101MHz)

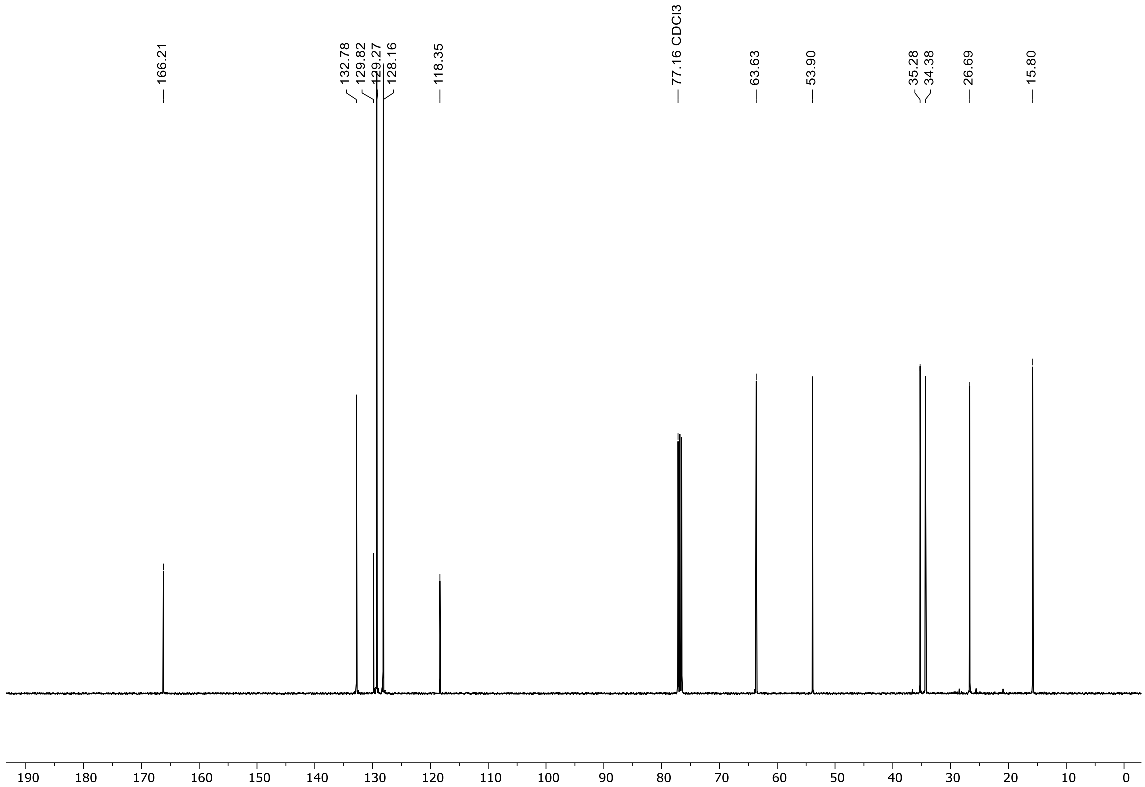

Diethyl 2-(5-(benzoyloxy)-2-bromopentyl)malonate (6)

^{1}H-NMR (400MHz)

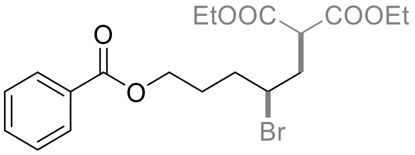

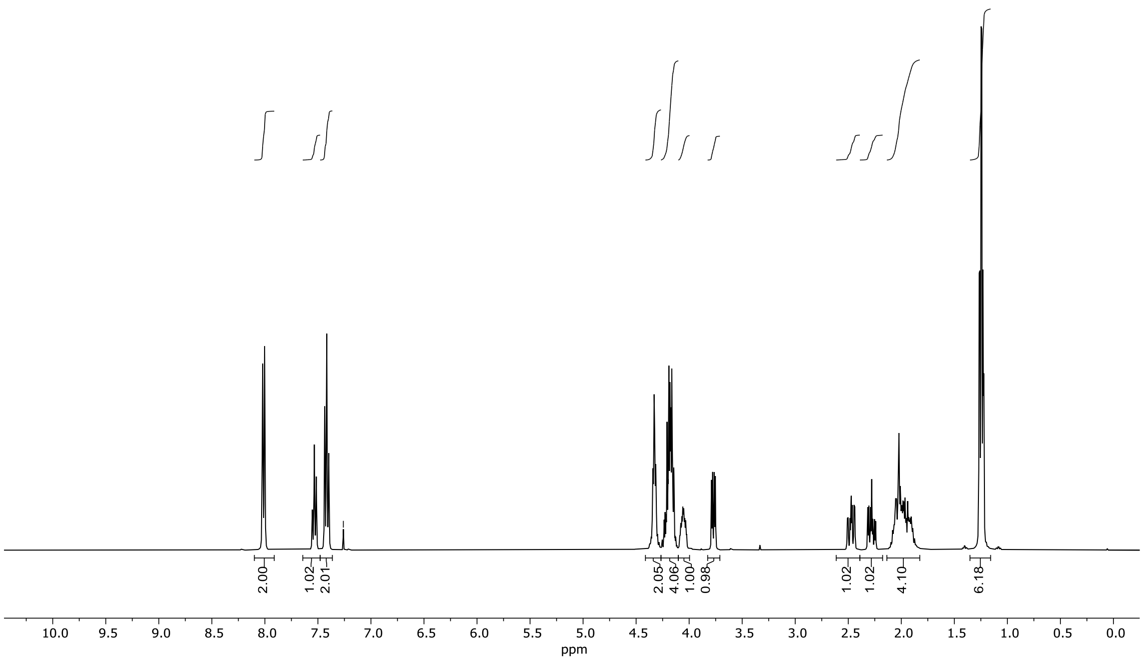

^{13}C-NMR (101MHz)

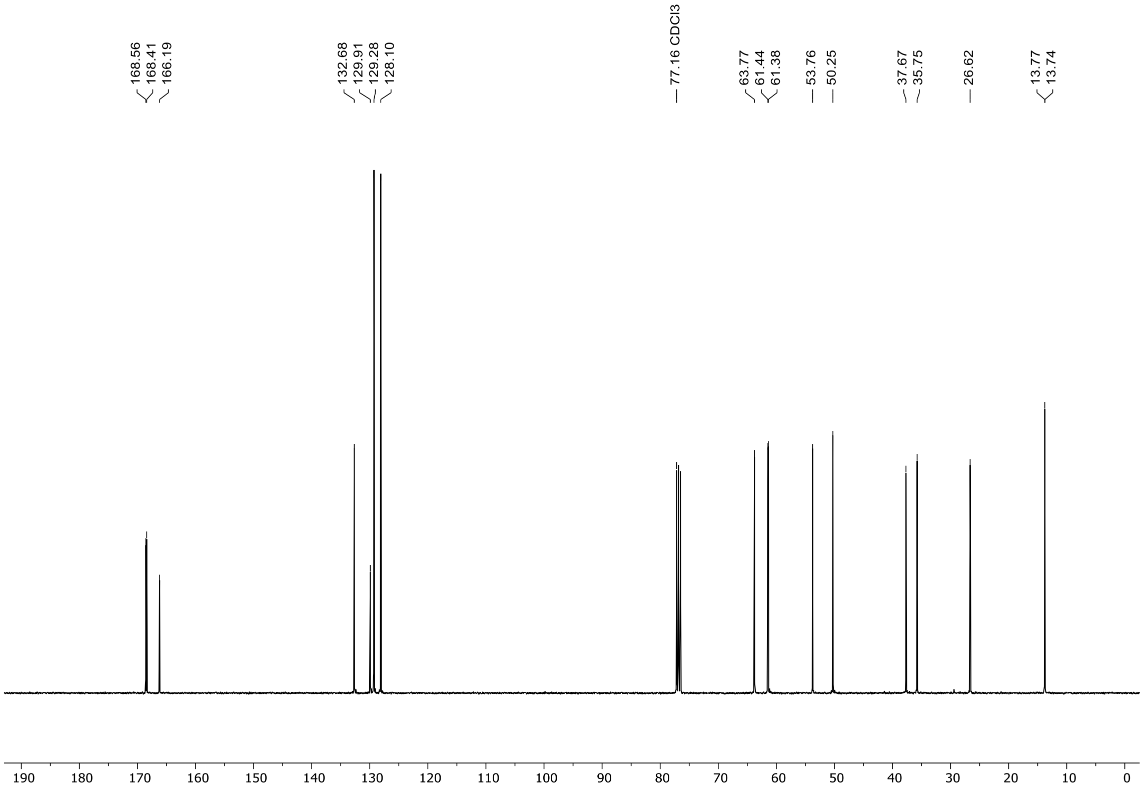

4-Bromo-7-ethoxy-6,6-difluoro-7-oxoheptyl benzoate (7)

^{1}H-NMR (400MHz)

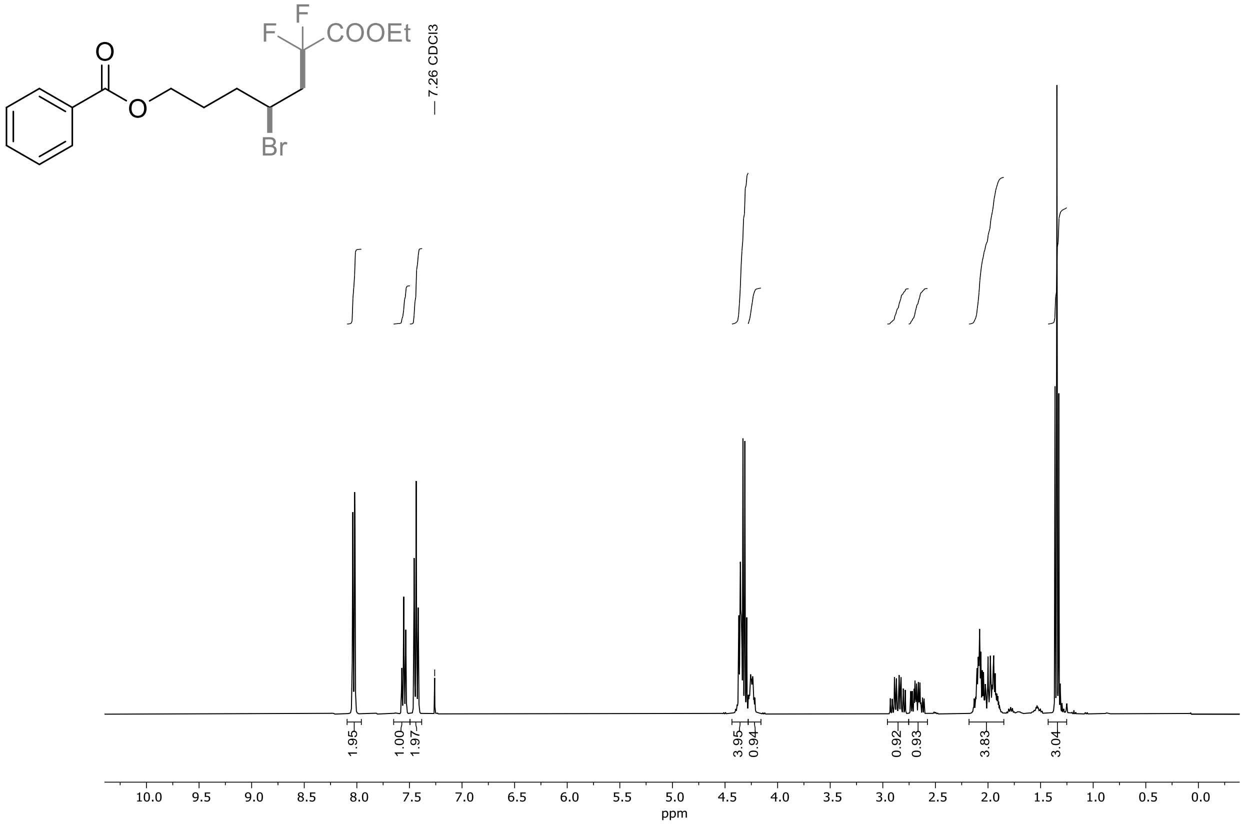

^{13}C-NMR (101MHz)

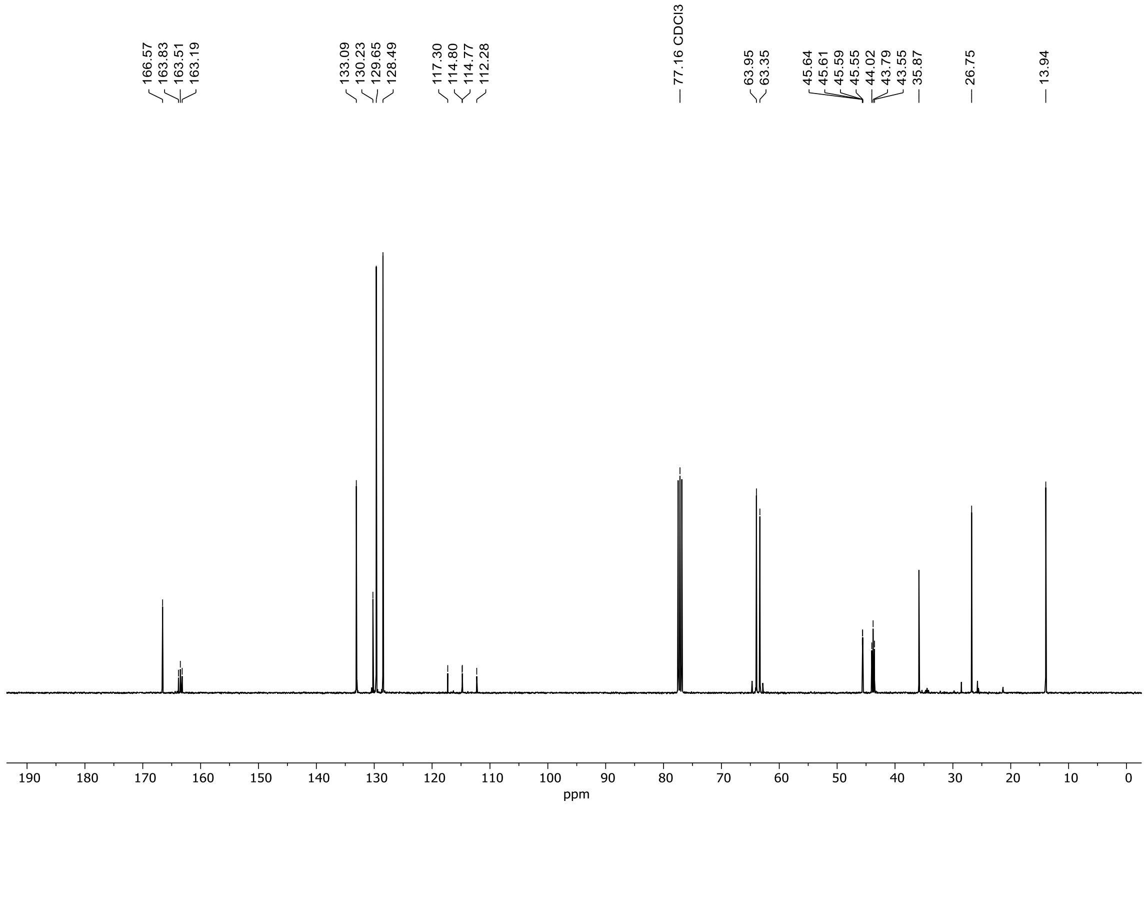

4-Bromo-7-ethoxy-7-oxoheptyl benzoate (8)

^{1}H-NMR (400MHz)

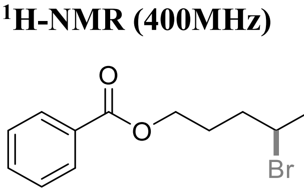

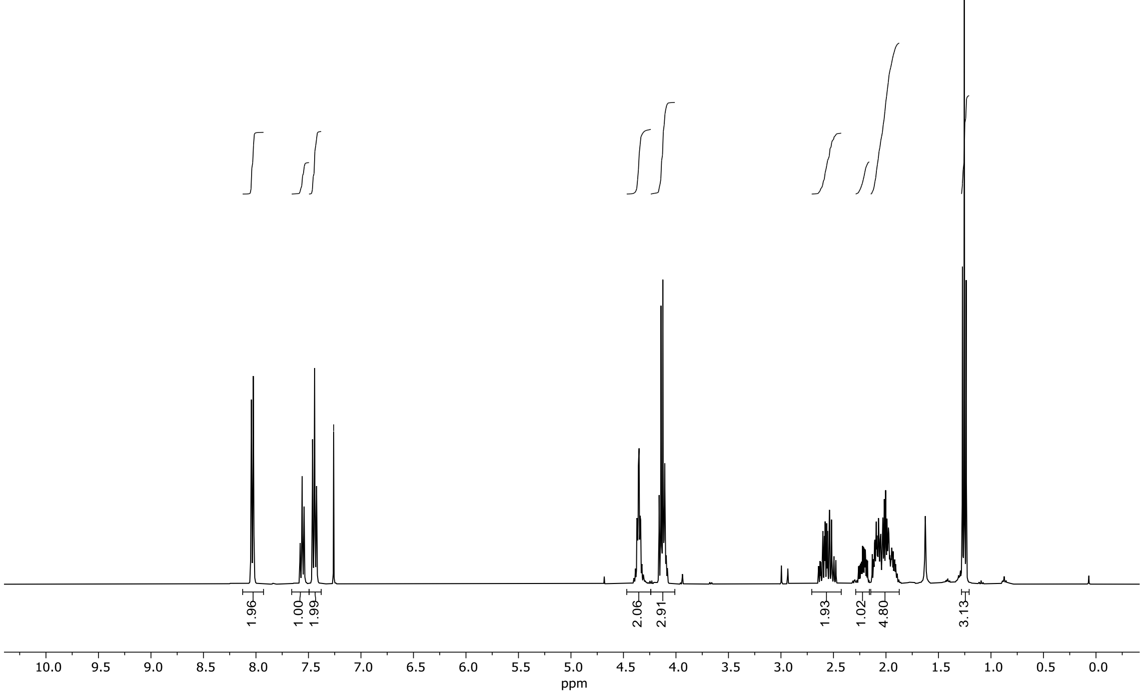

^{13}C-NMR (101MHz)

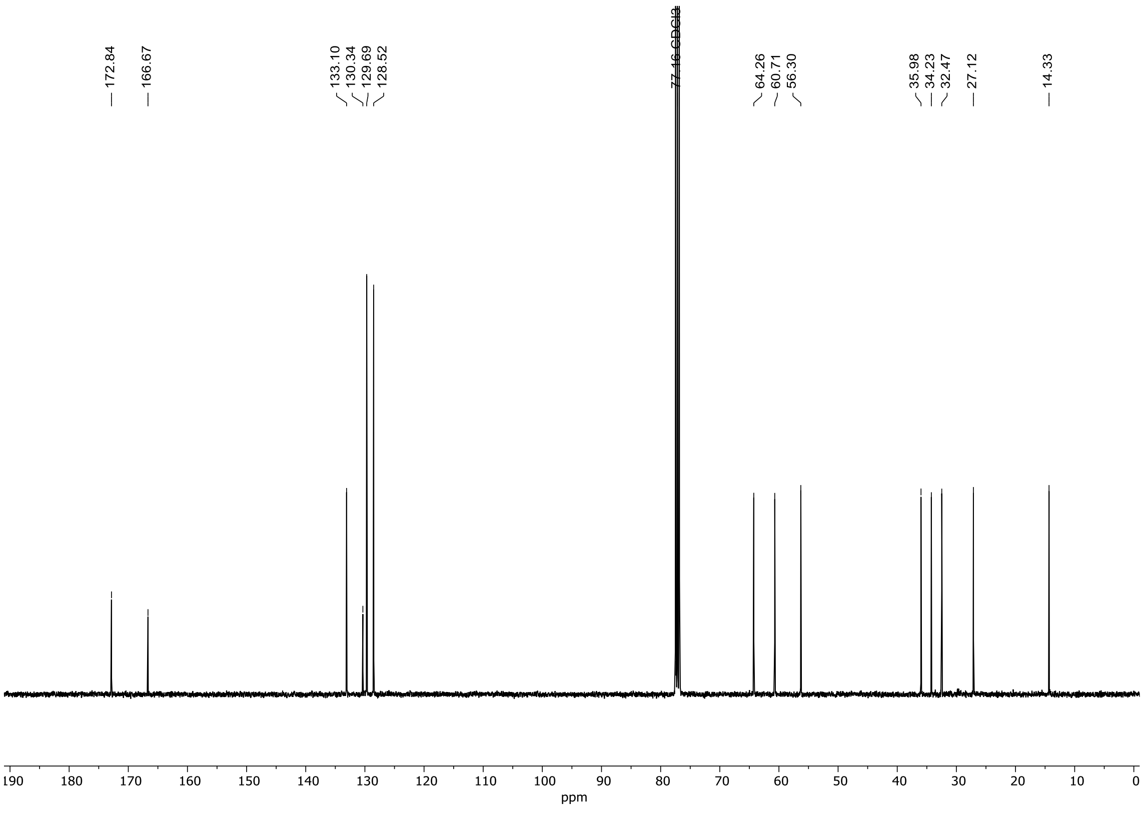

4-Bromo-6-nitrohexyl benzoate (9)

^{1}H-NMR (400MHz)

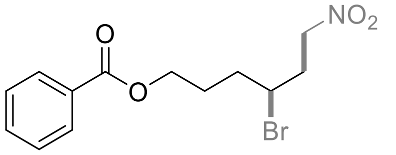

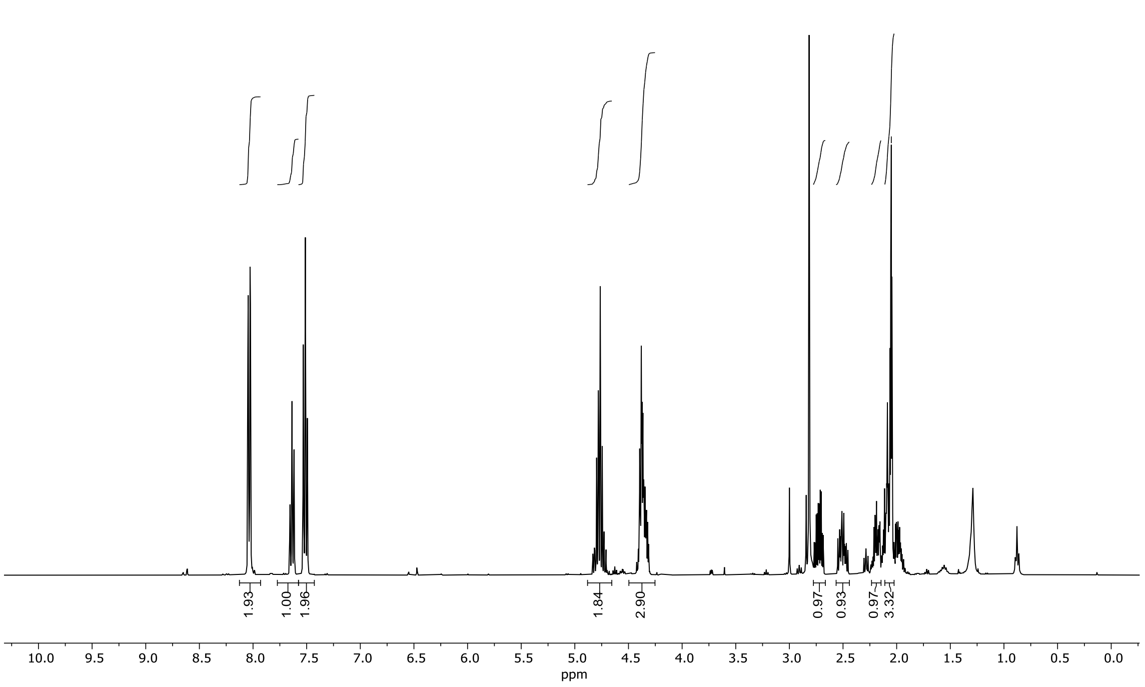

^{13}C-NMR (101MHz)

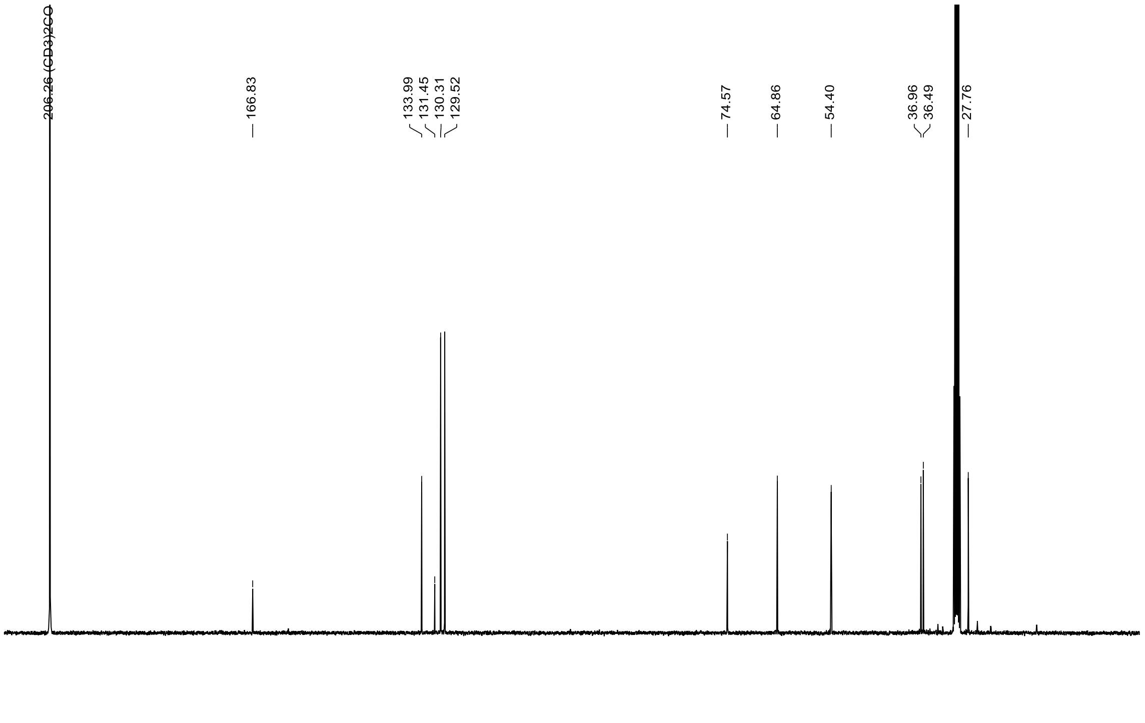

4-Bromo-7-oxo-7phenylheptyl benzoate (10)

^{1}H-NMR (400MHz)

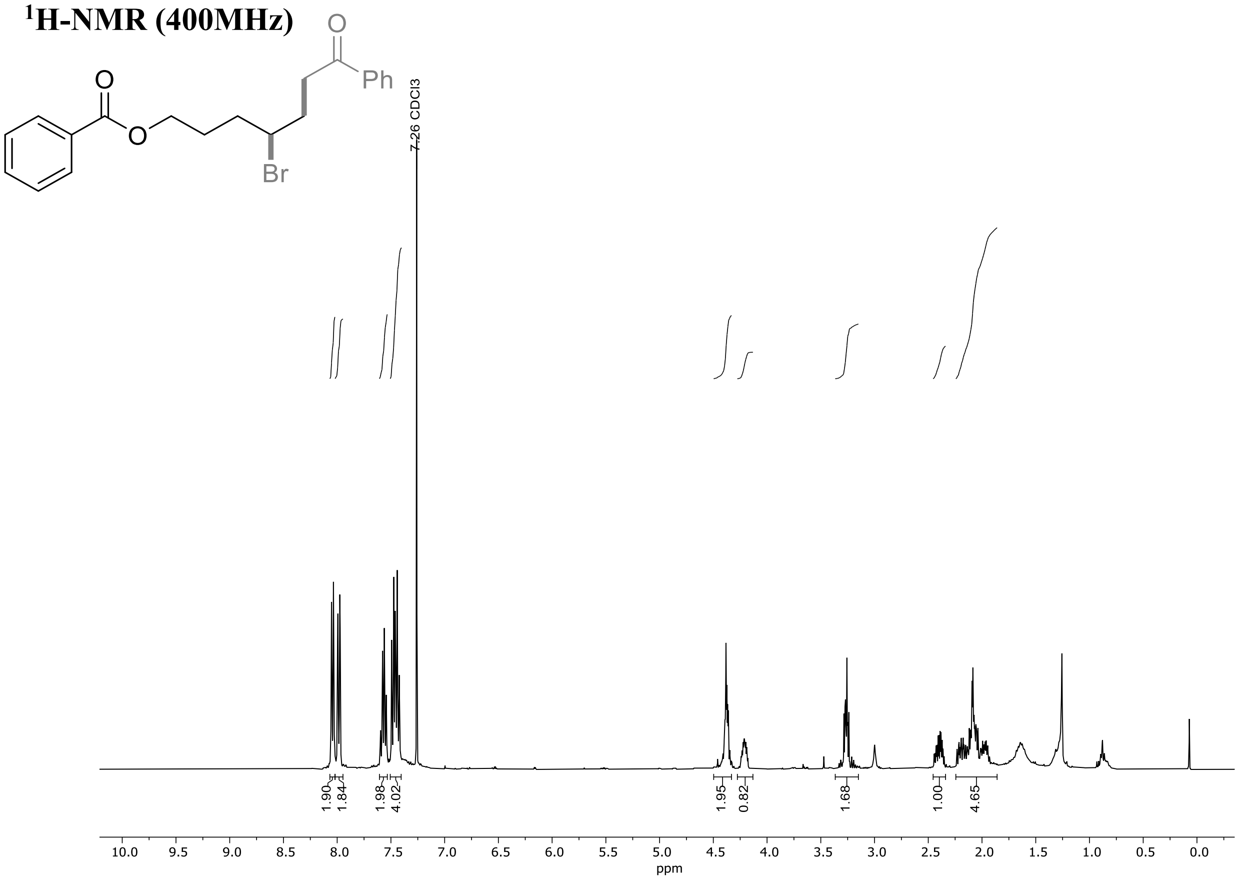

^{13}C-NMR (101MHz)

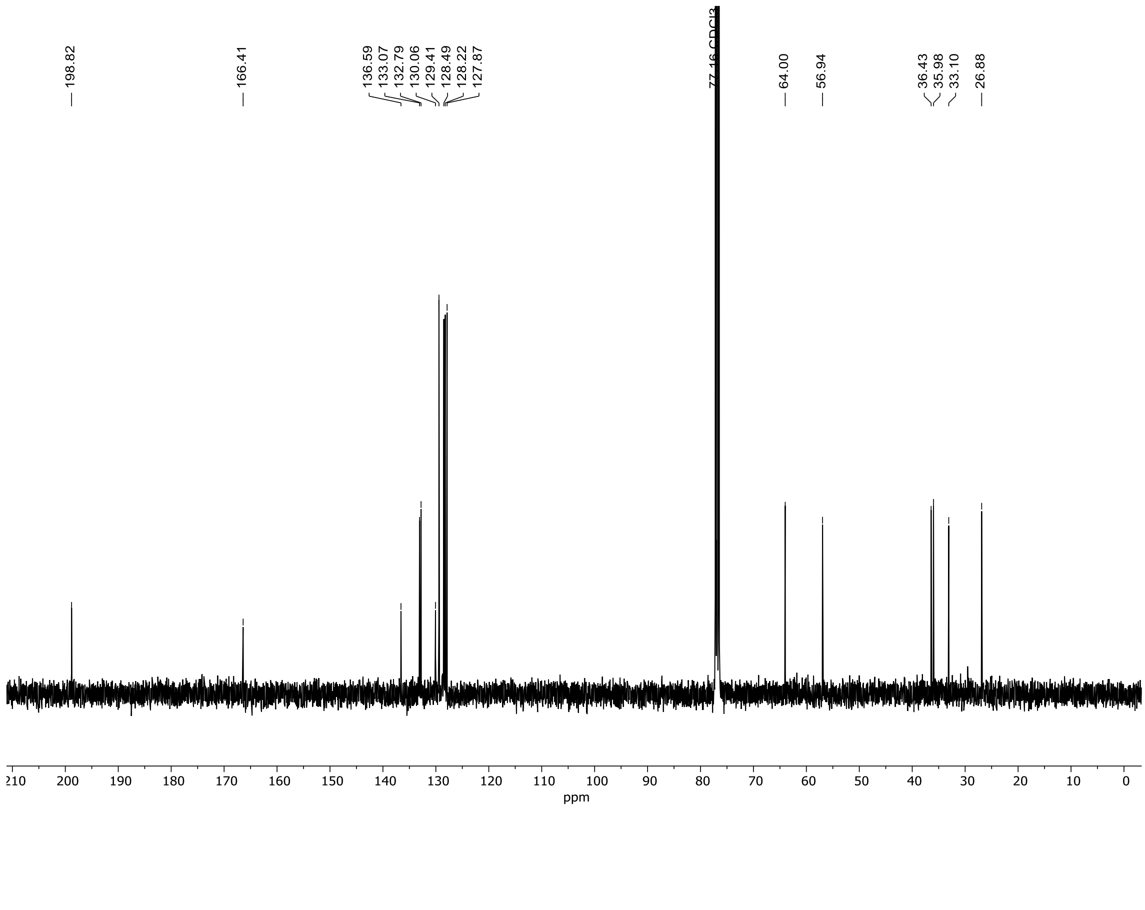

6,6,7,7,8,8,9,9,10,10,11,11,11-Tridecafluoro-4-iodoundecyl benzoate (11)

^{1}H-NMR (400MHz)

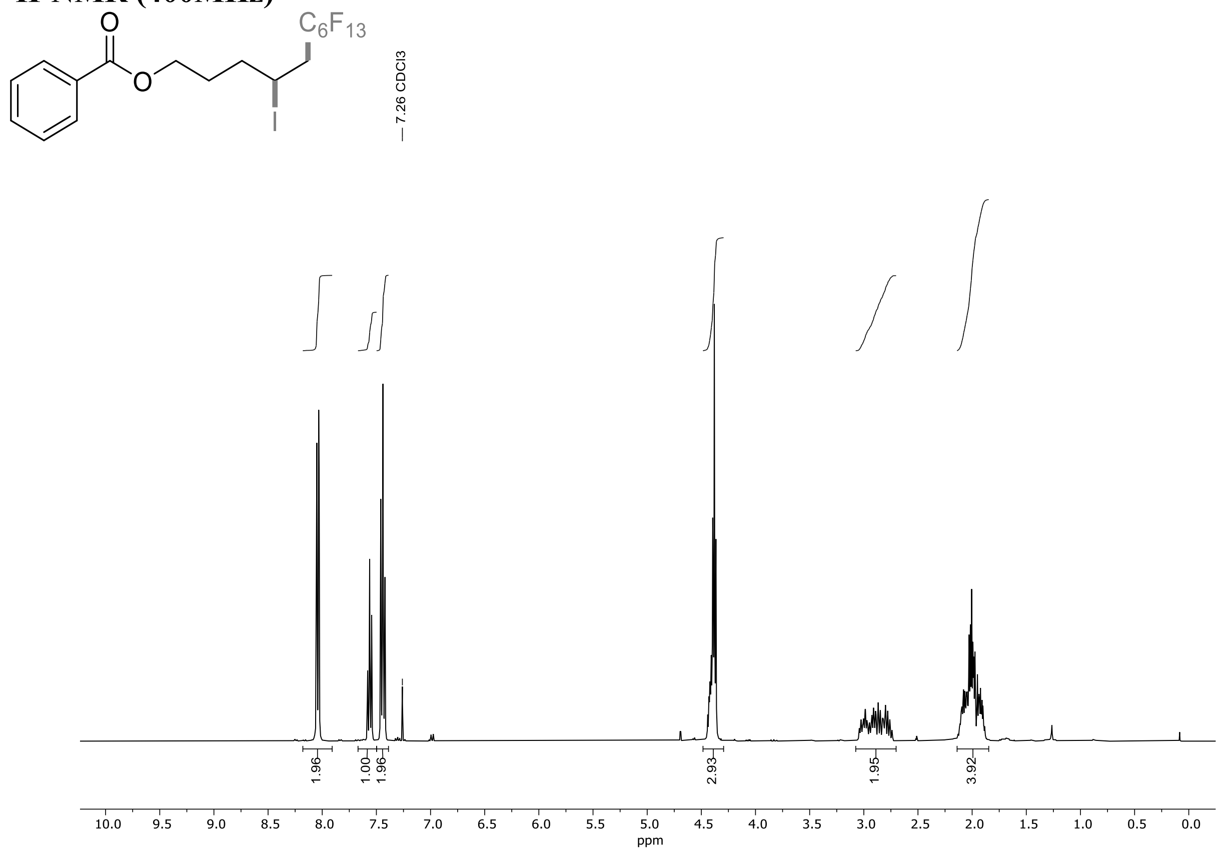

^{13}C-NMR (101MHz)

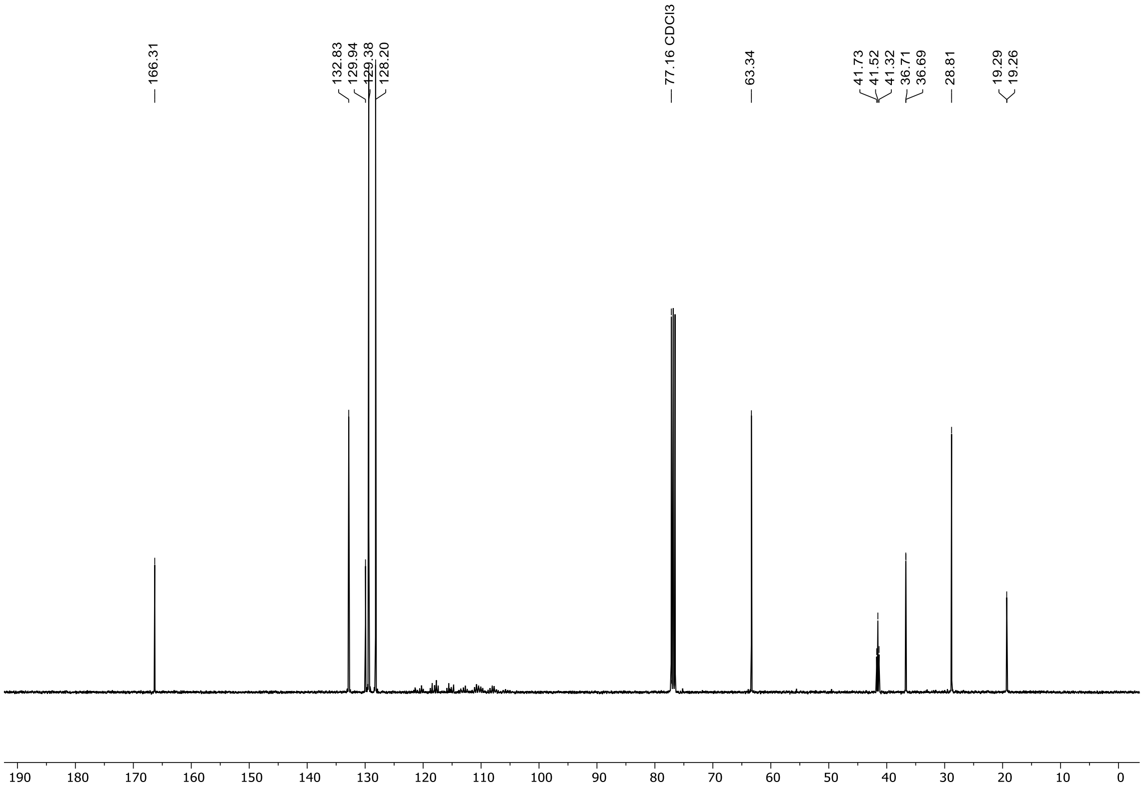

4-Bromo-6,6,6-trichlorohexyl 4-methylbenzenesulfonate (12)

^{1}H-NMR (400MHz)

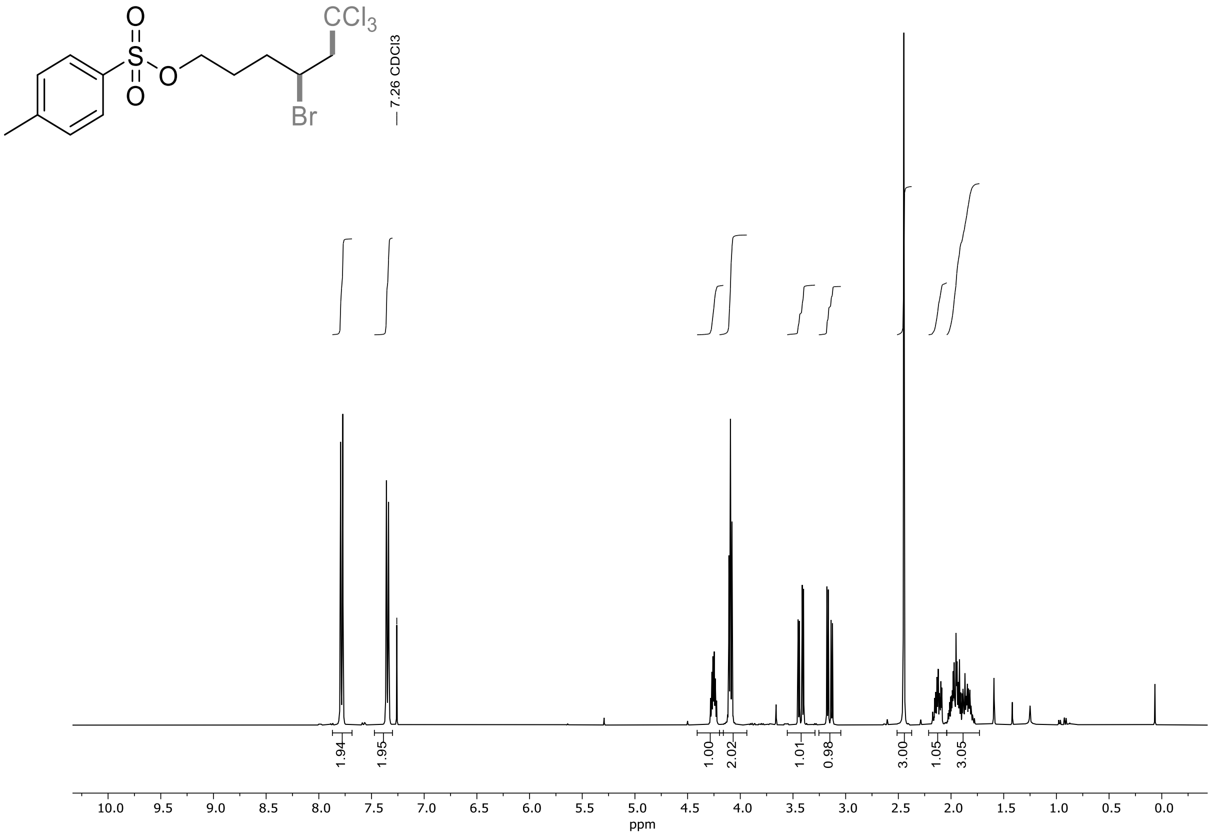

^{13}C-NMR (101MHz)

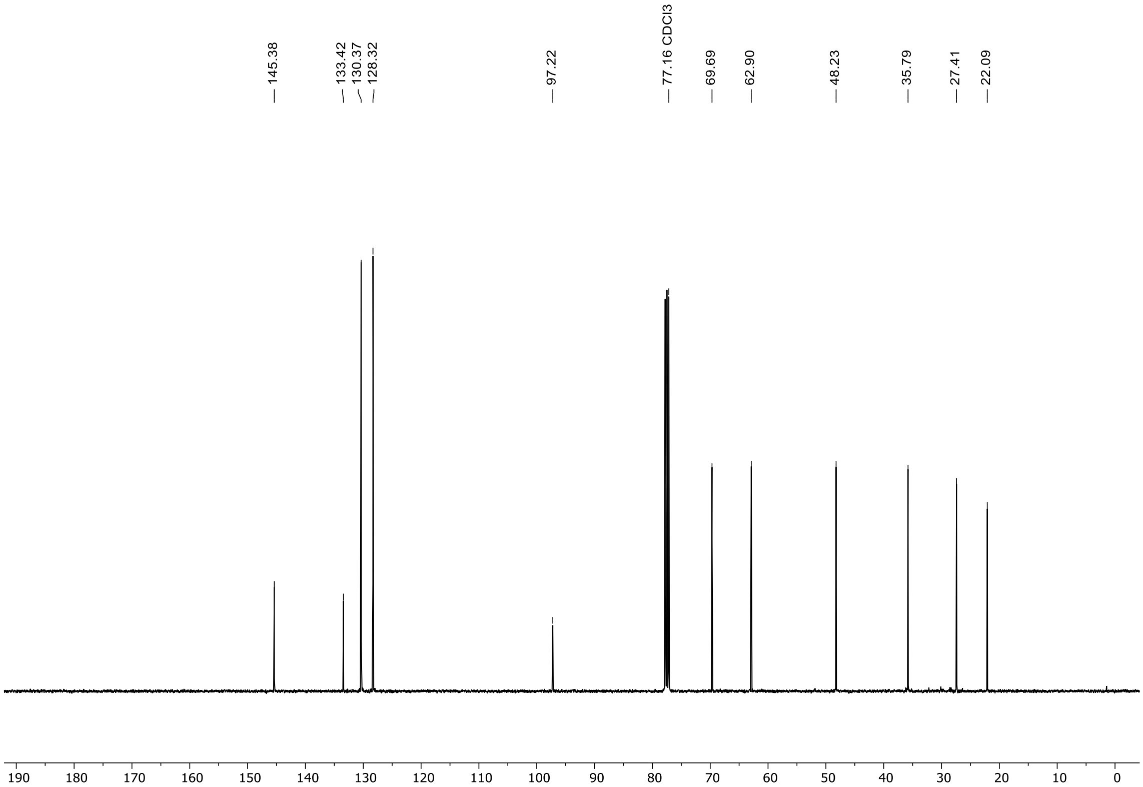

4-Bromo-6-cyanohexyl 4-methylbenzenesulfonate (13)

^{1}H-NMR (400MHz)

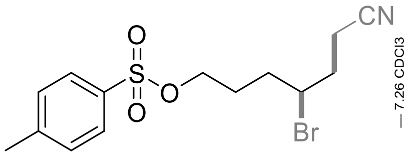

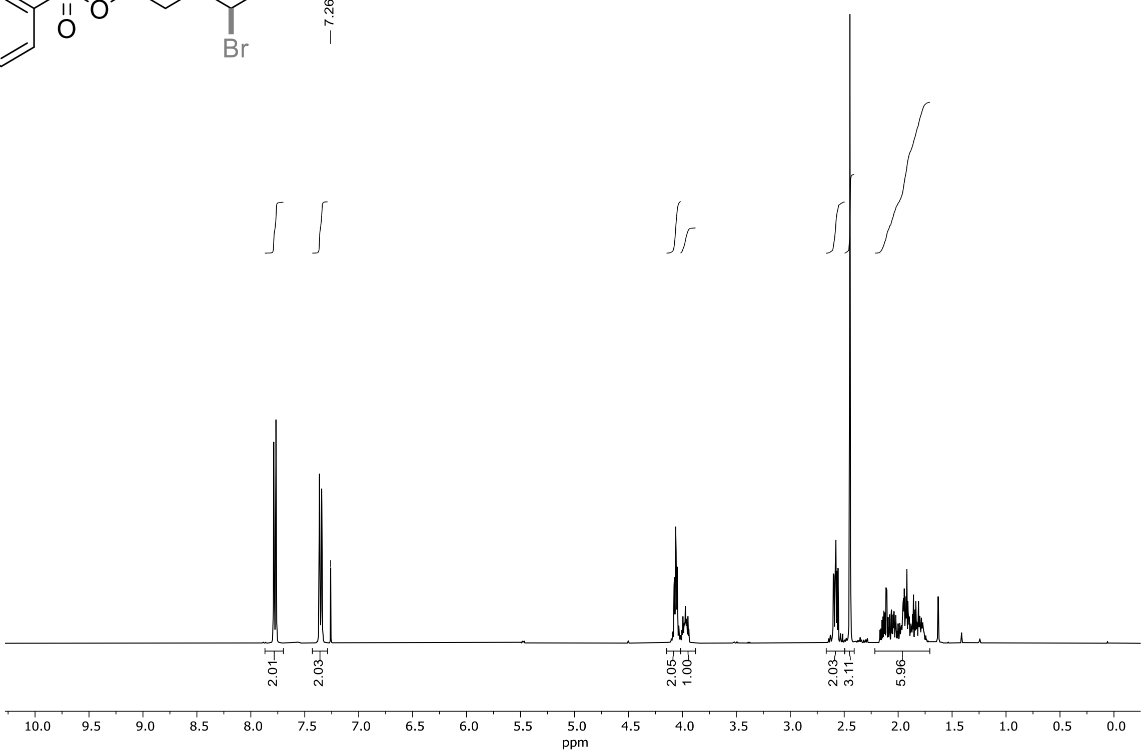

^{13}C-NMR (101MHz)

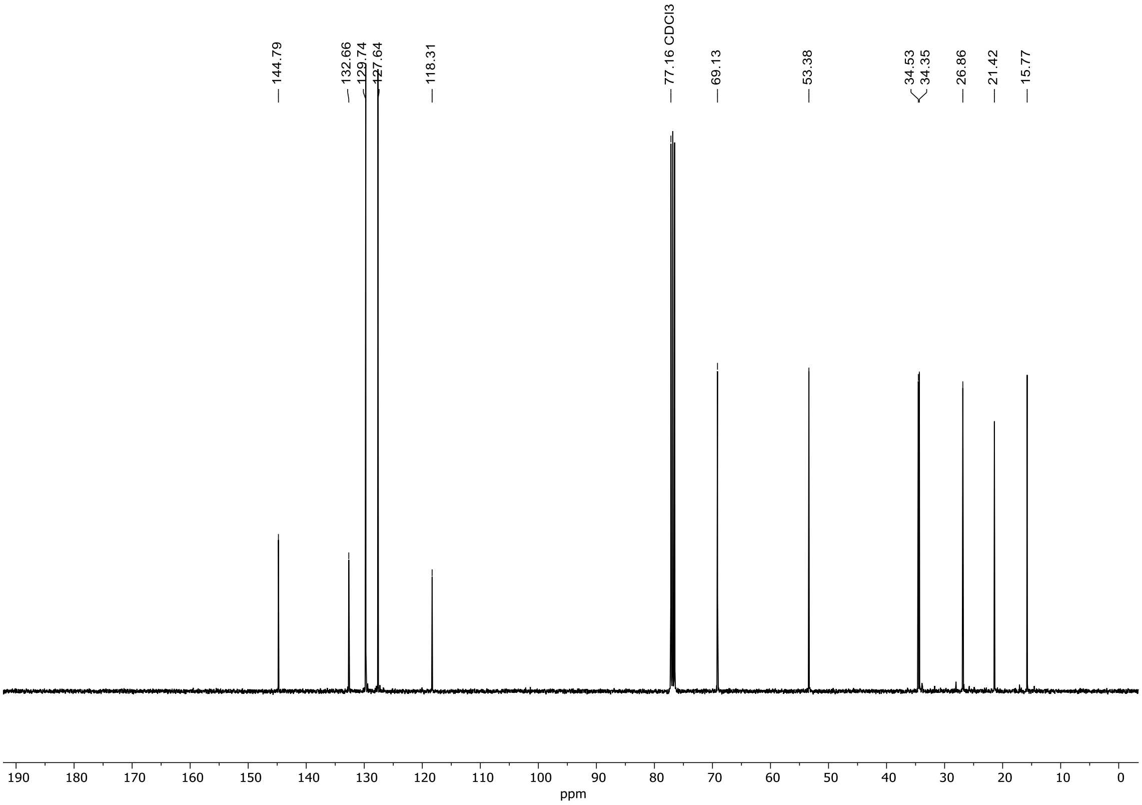

Diethyl 2-(2-bromo-5-(tosyloxy)pentyl)malonate (14)

^{1}H-NMR (400MHz)

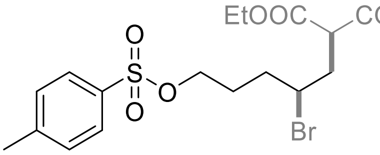

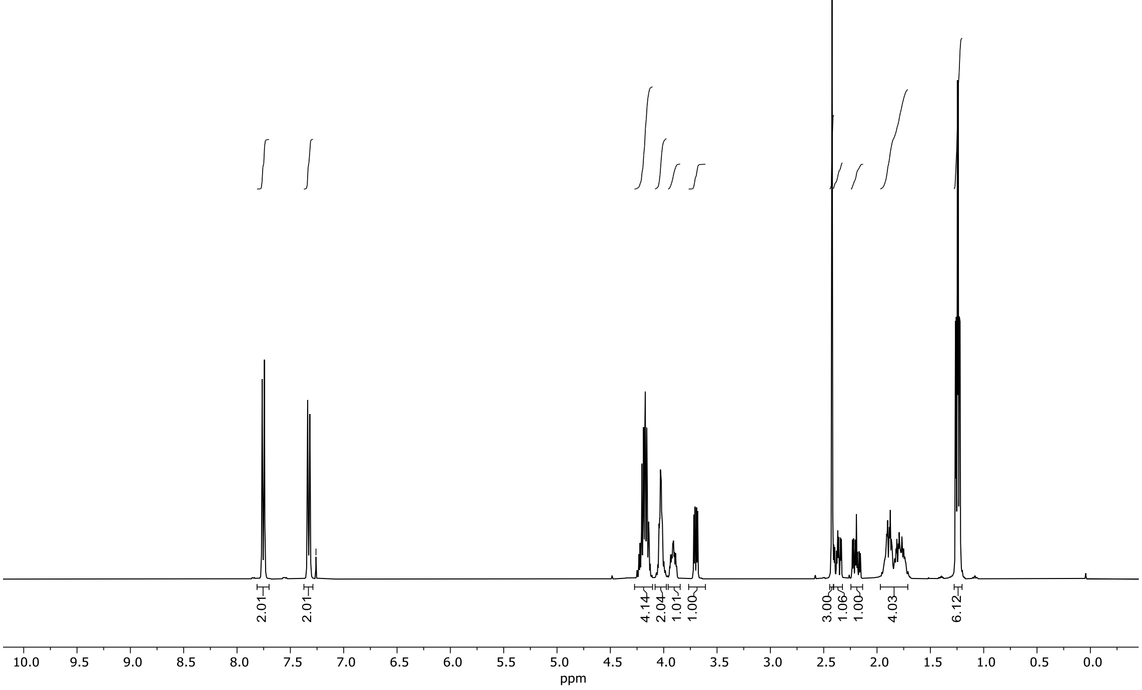

^{13}C-NMR (101MHz)

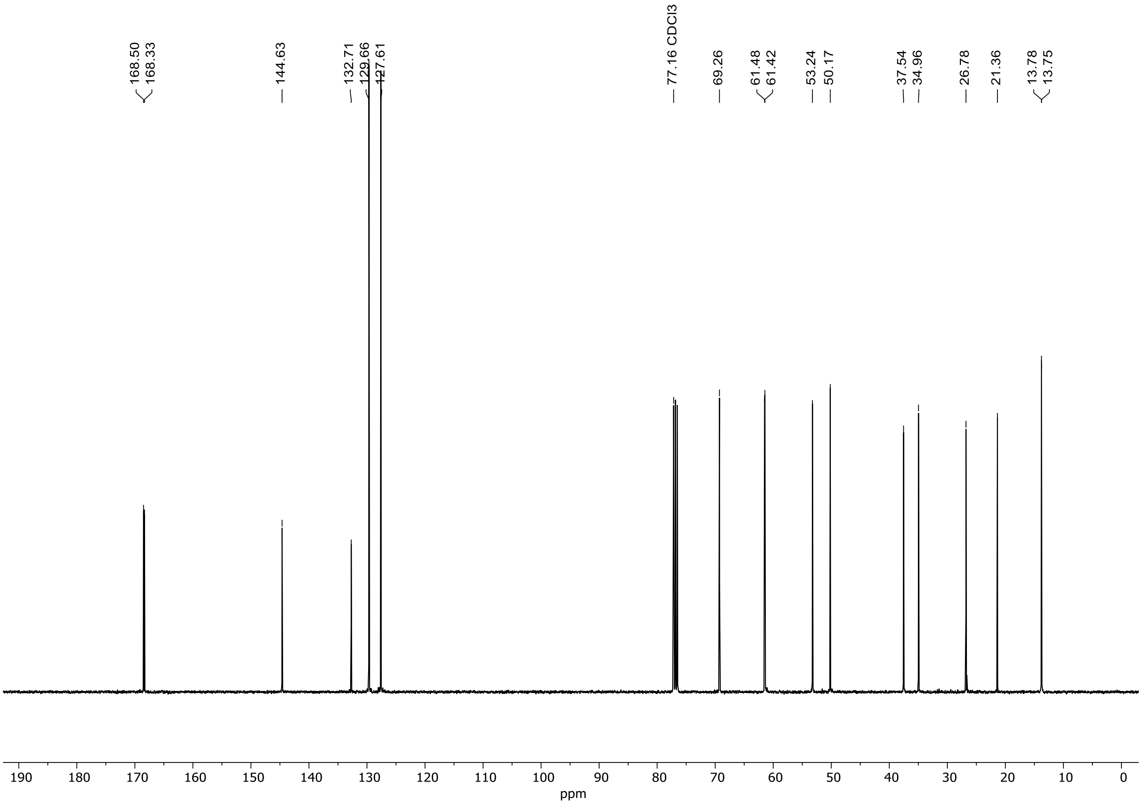

Ethyl 4-bromo-2,2-difluoro-7-(tosyloxy)heptanoate (15)

^{1}H-NMR (400MHz)

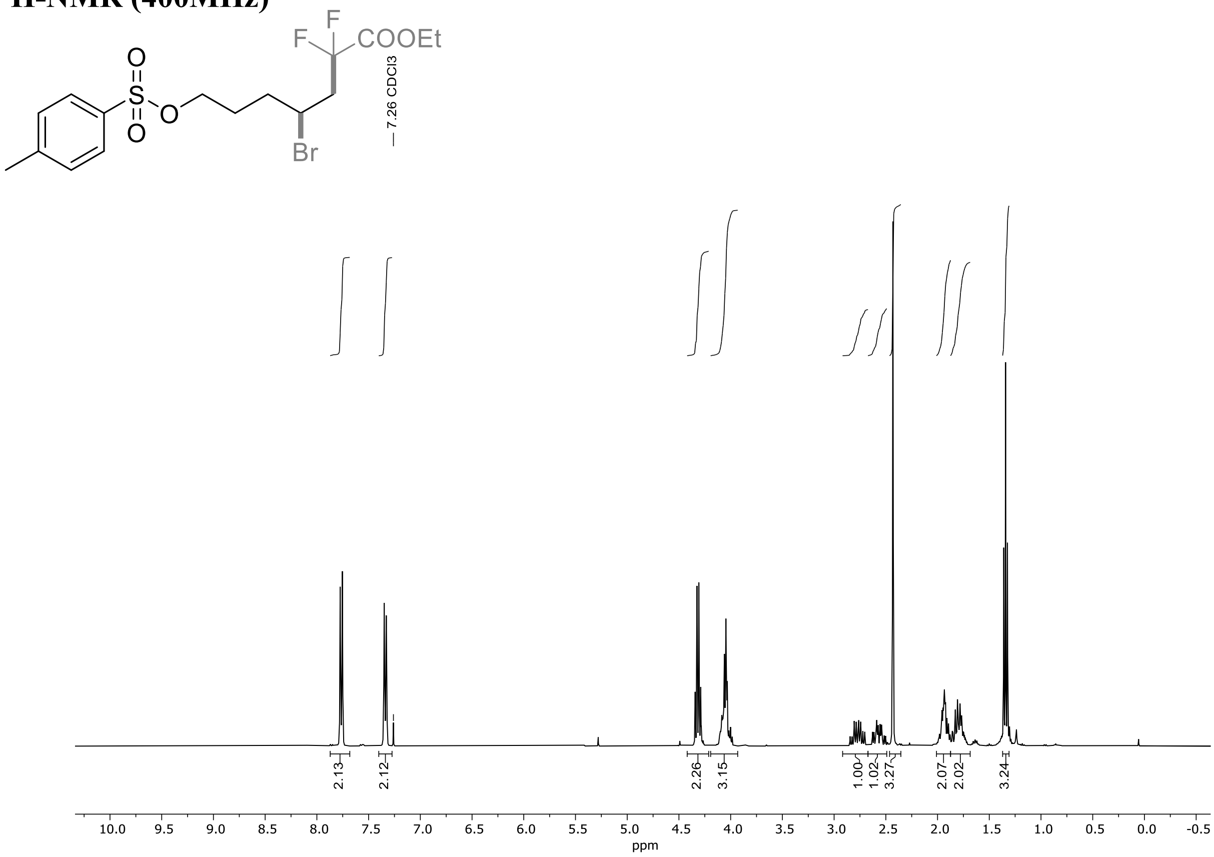

^{13}C-NMR (101MHz)

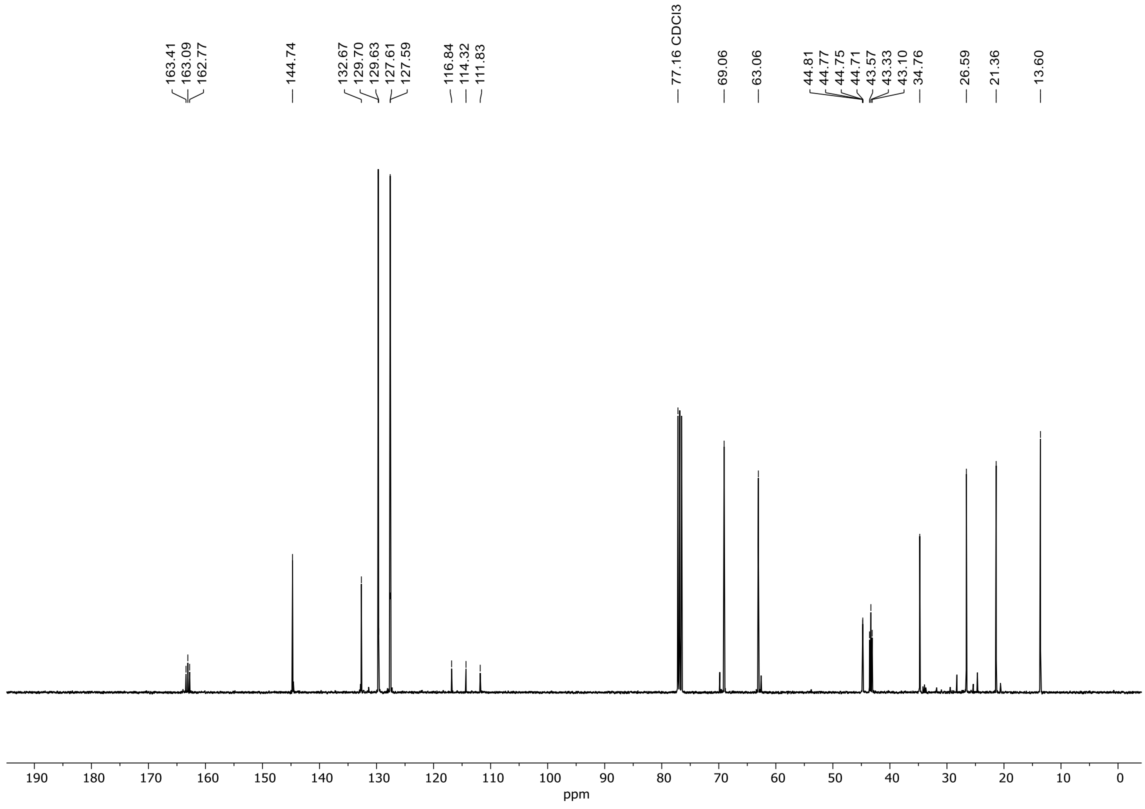

2-Bromo-4,4,4-trichlorobutyl benzoate (16)

^{1}H-NMR (400MHz)

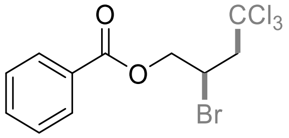

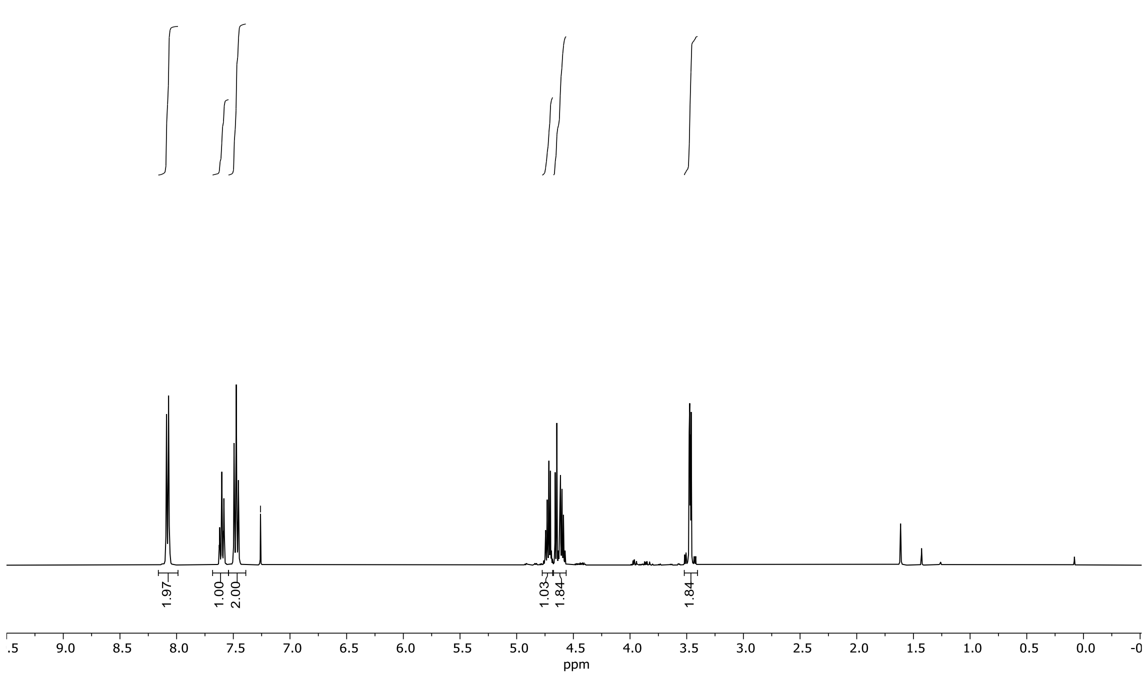

^{13}C-NMR (101MHz)

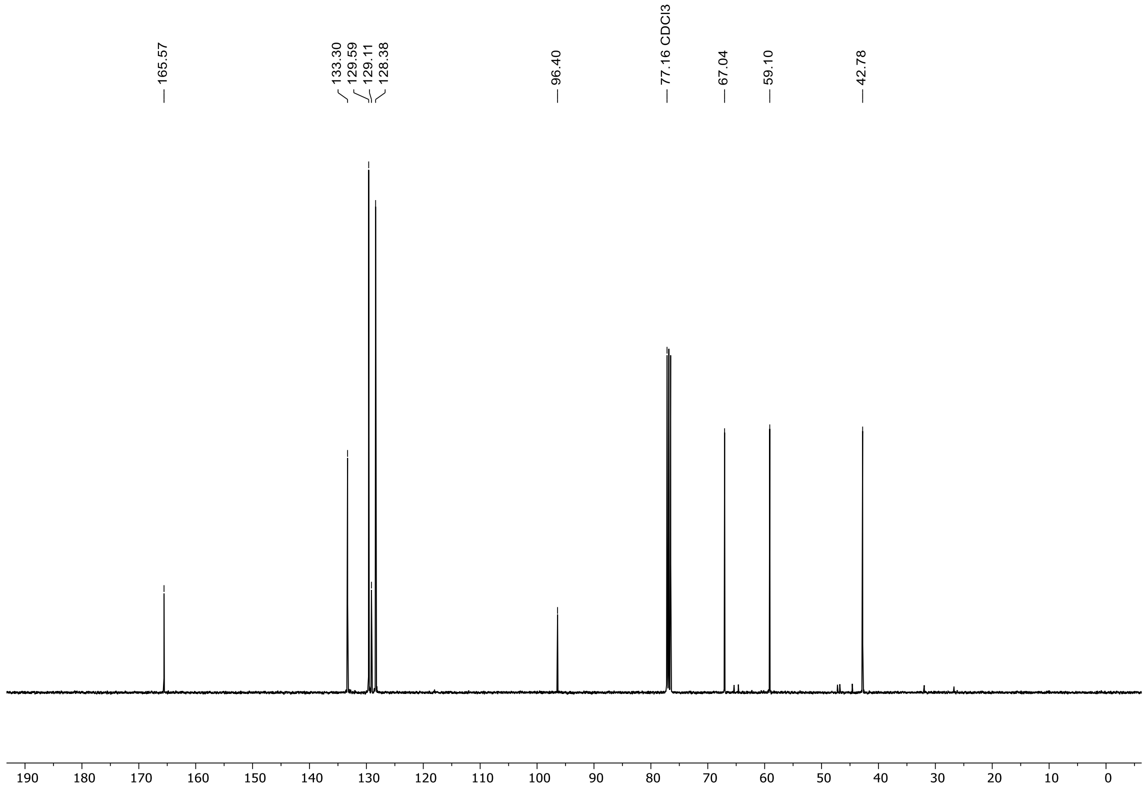

2-Bromo-4-cyanobutyl benzoate (17)

^{1}H-NMR (400MHz)

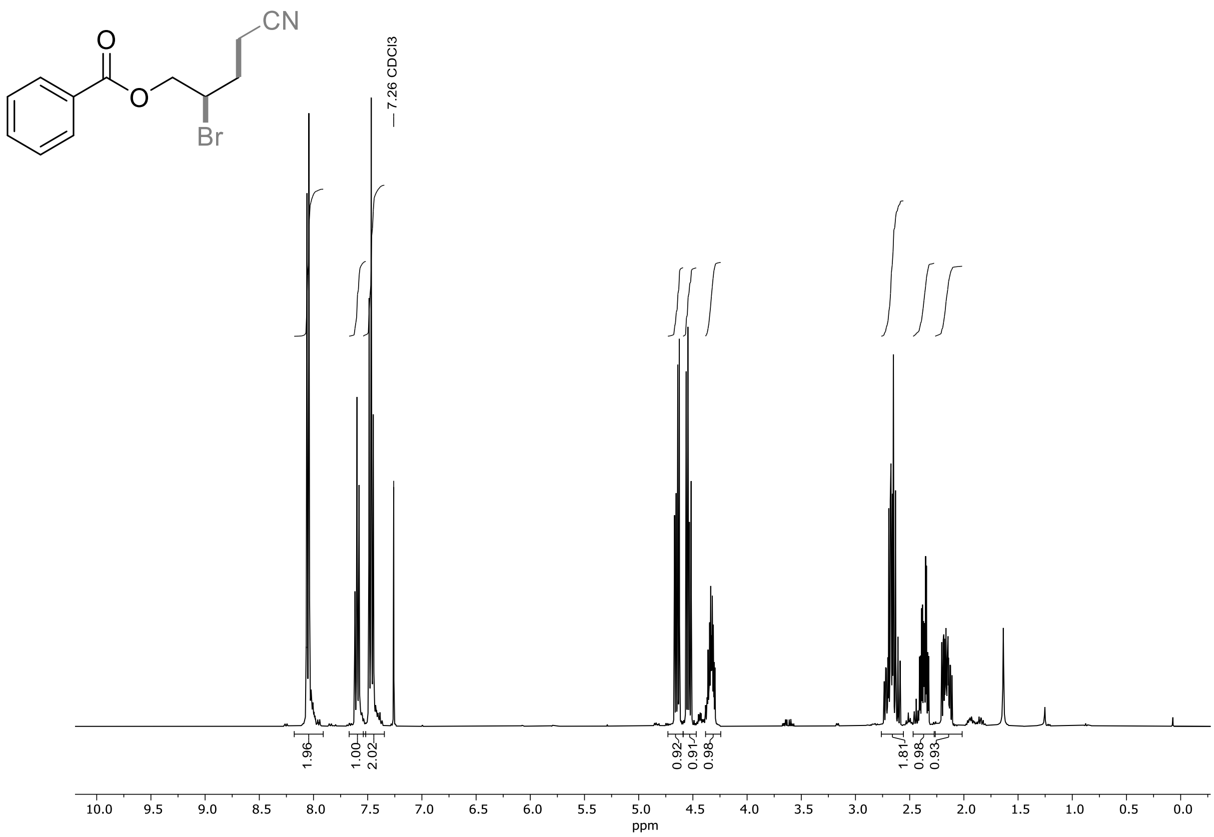

^{13}C-NMR (101MHz)

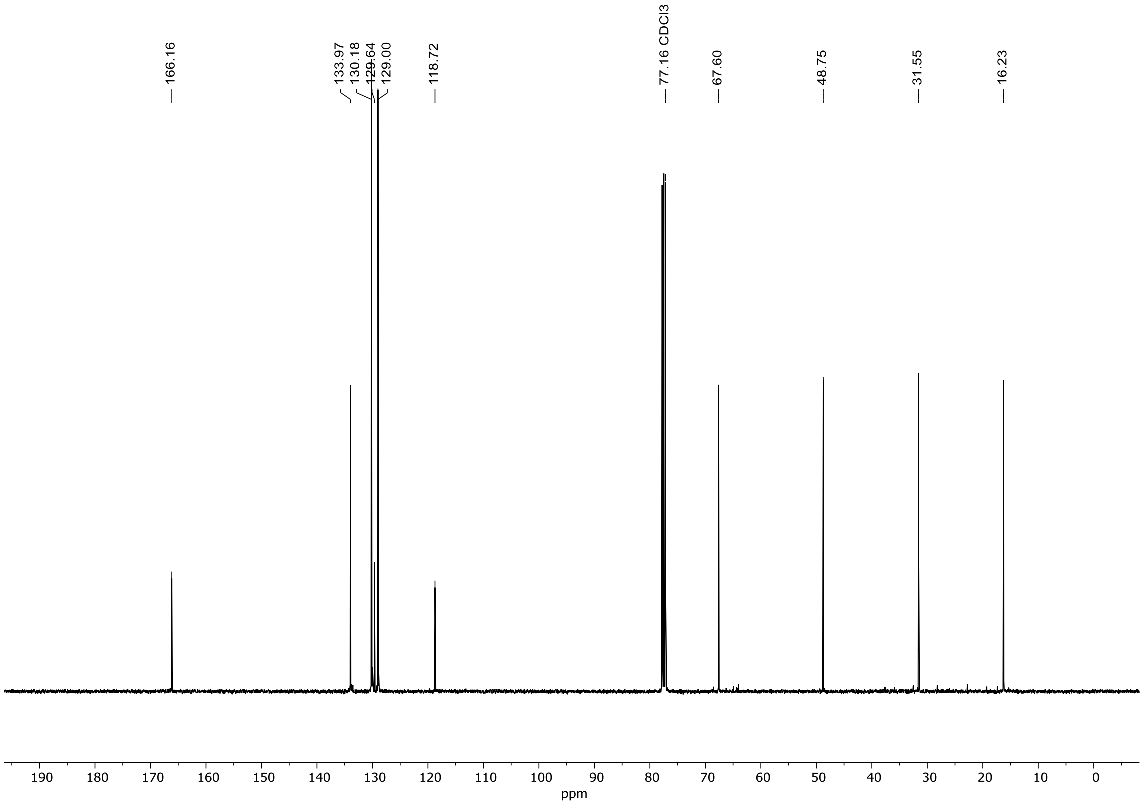

Diethyl 2-(3-(benzoyloxy)-2-bromopropyl)malonate (18)

^{1}H-NMR (400MHz)

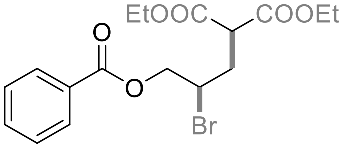

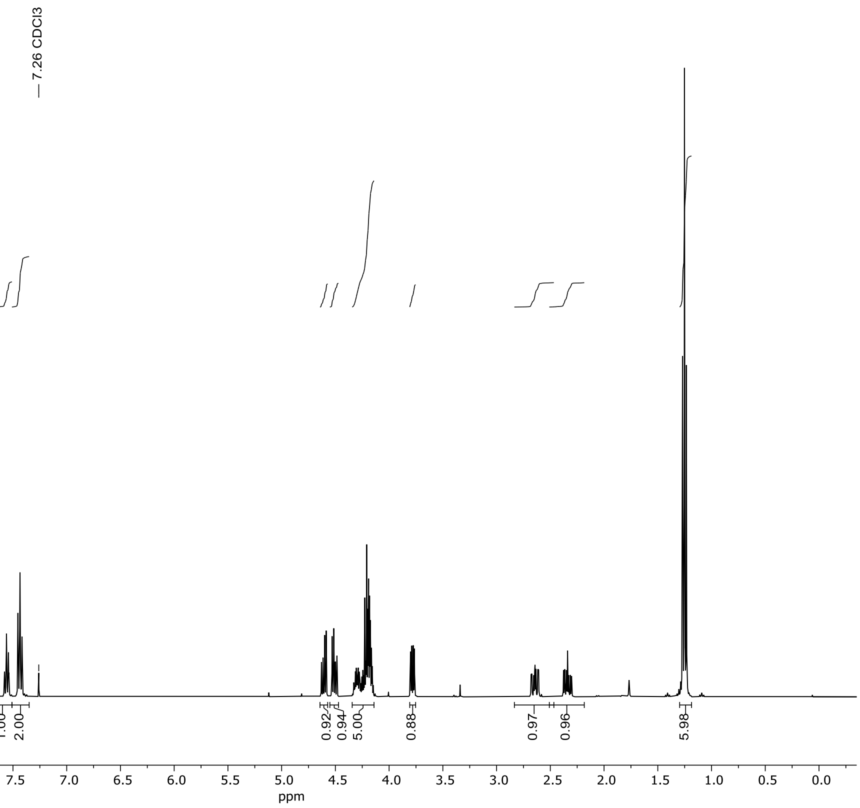

^{13}C-NMR (101MHz)

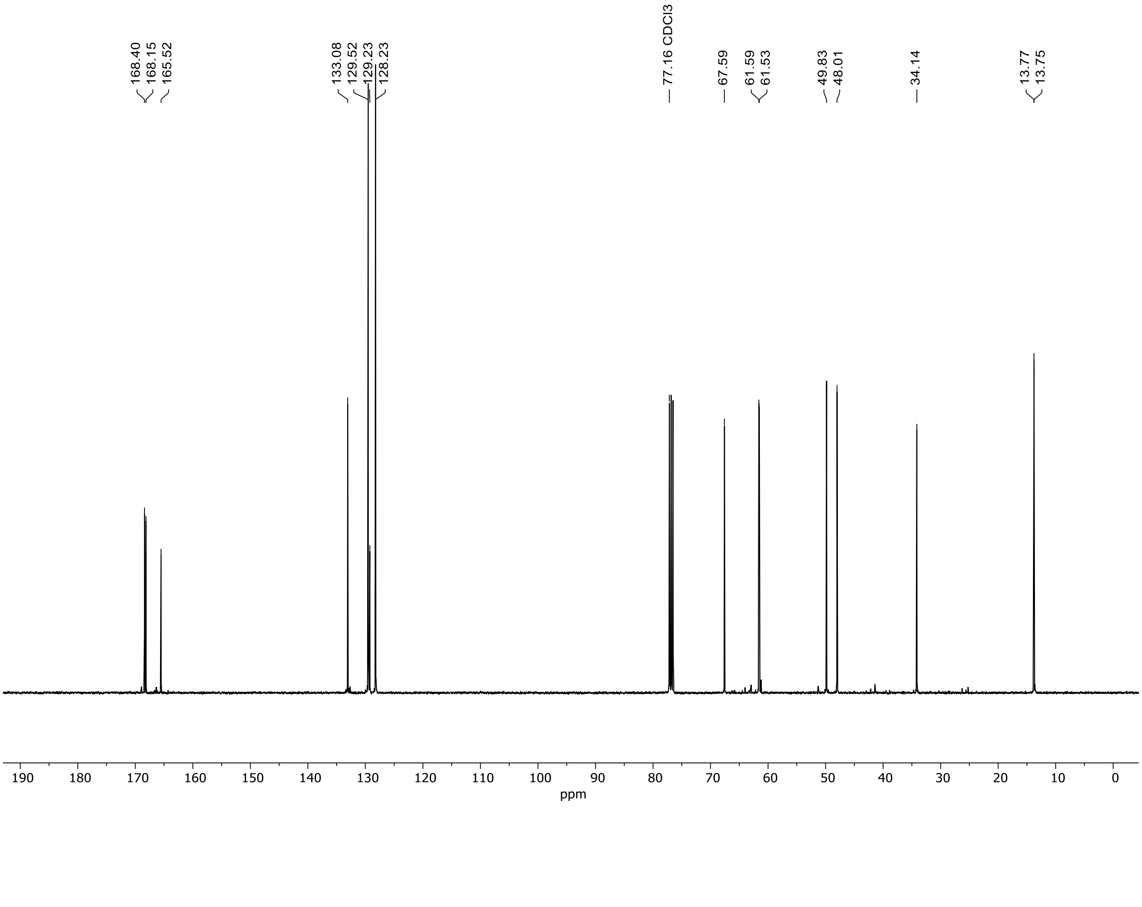

2-Bromo-5-ethoxy-4,4-difluoro-5-oxopentyl benzoate (19)

^{1}H-NMR (400MHz)

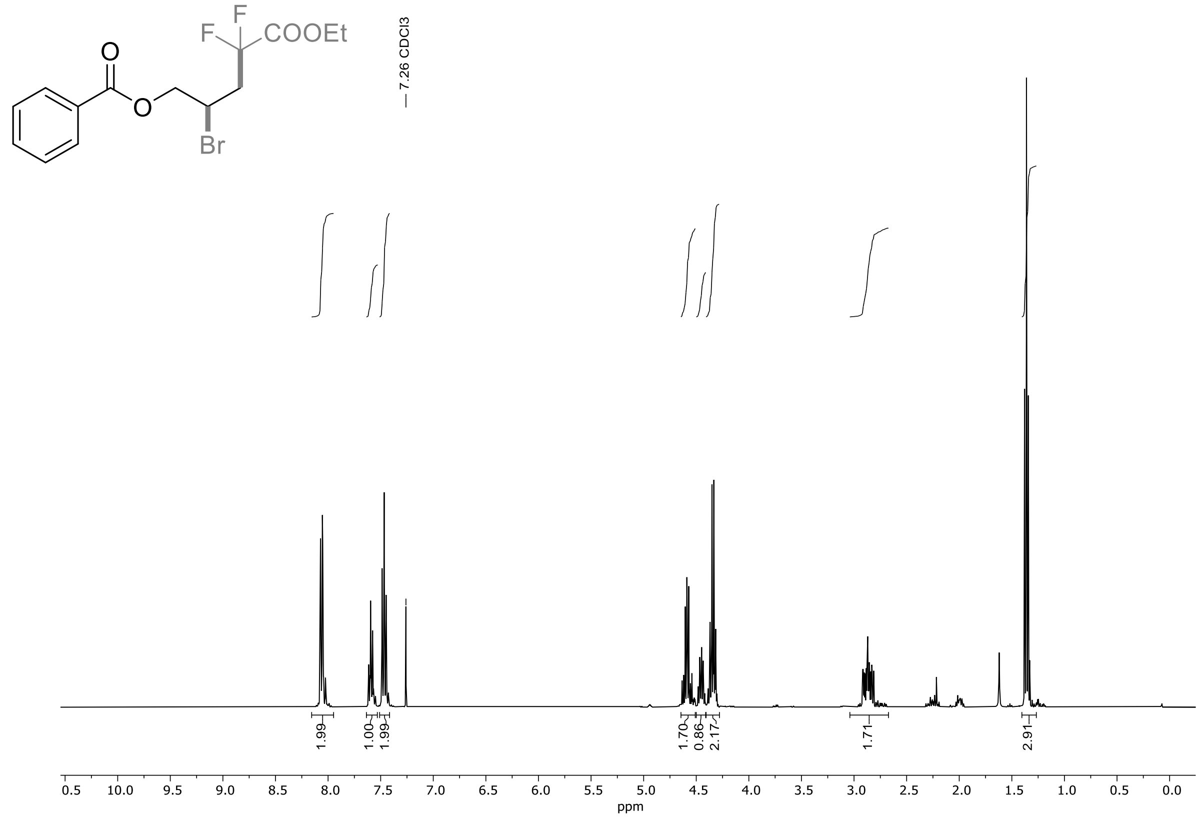

^{13}C-NMR (101MHz)

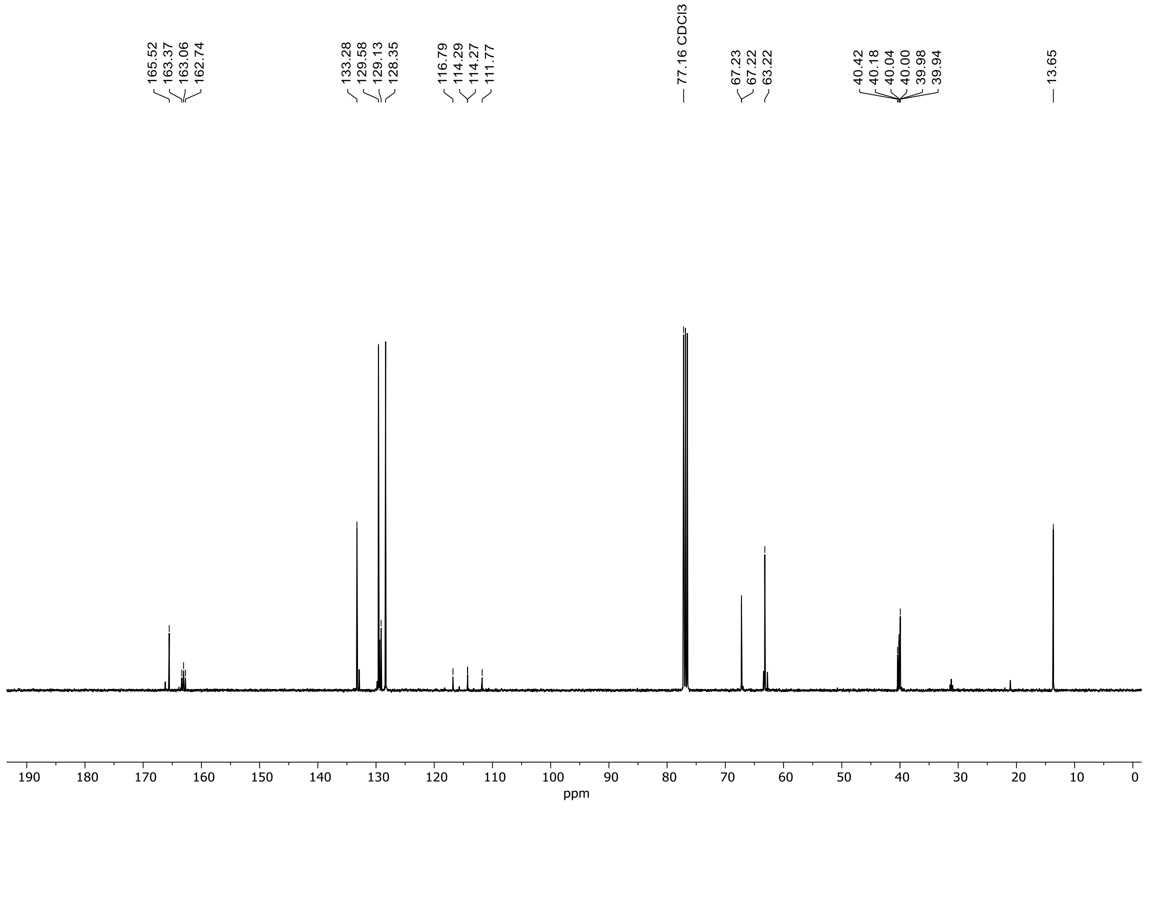

2-Bromo-4,4,4-trichloro-2-methylbutyl benzoate (20)

^{1}H-NMR (400MHz)

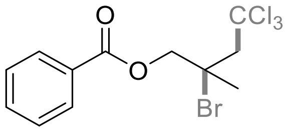

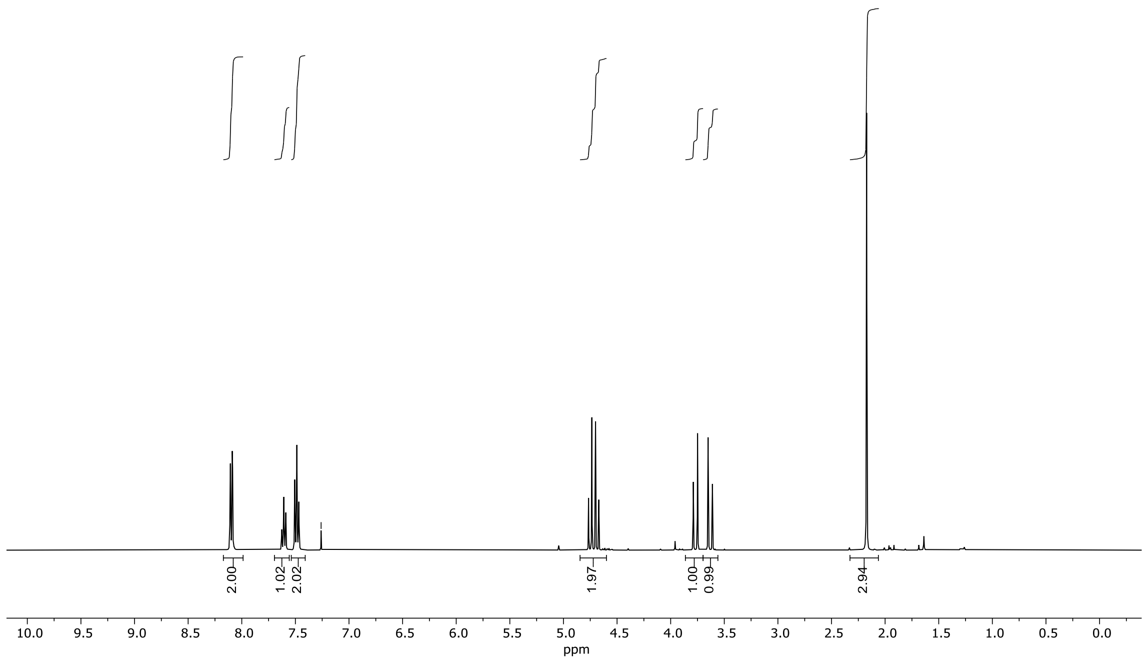

^{13}C-NMR (101MHz)

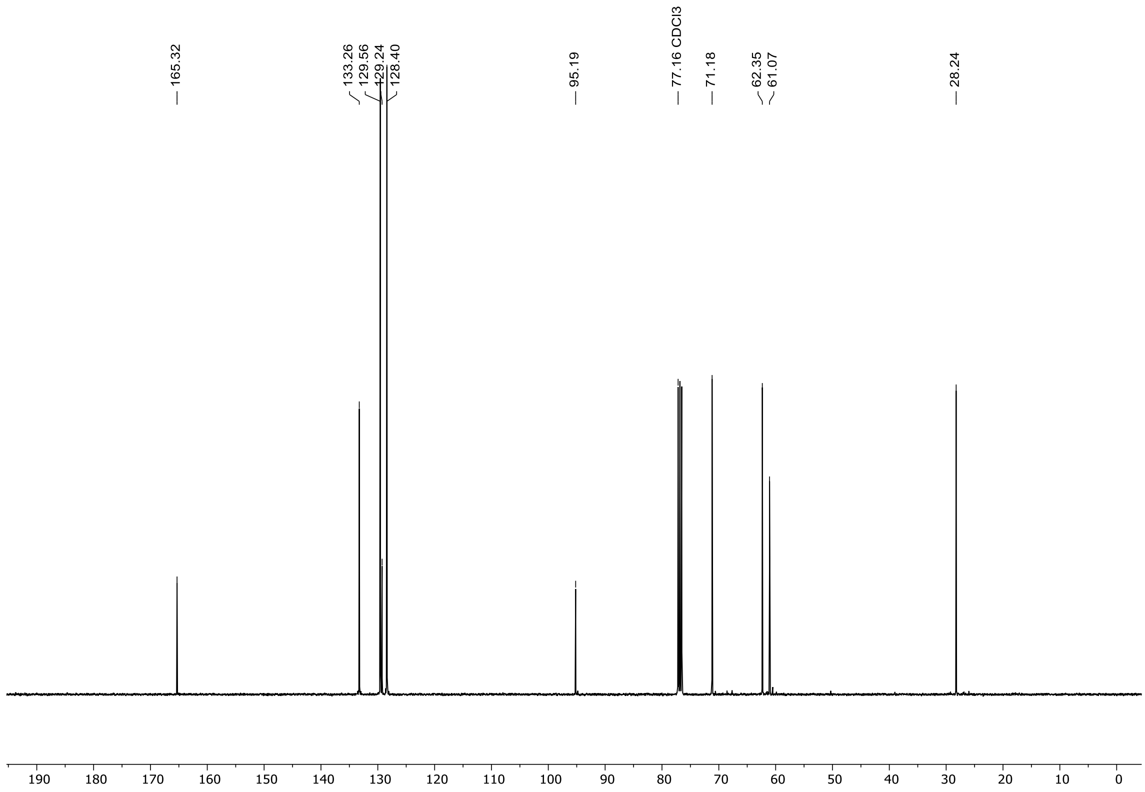

2-Bromo-4cyano-2-methylbutyl benazoate (21)

^{1}H-NMR (400MHz)

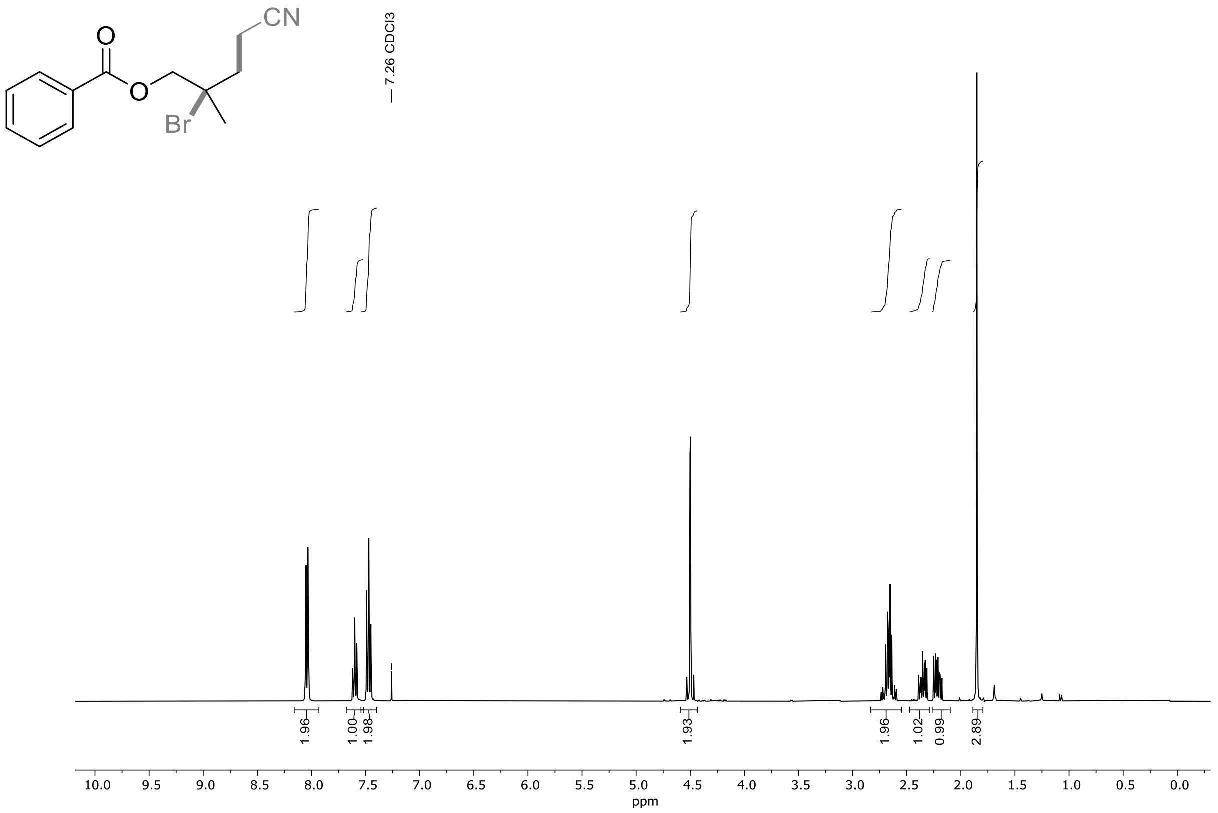

^{13}C-NMR (101MHz)

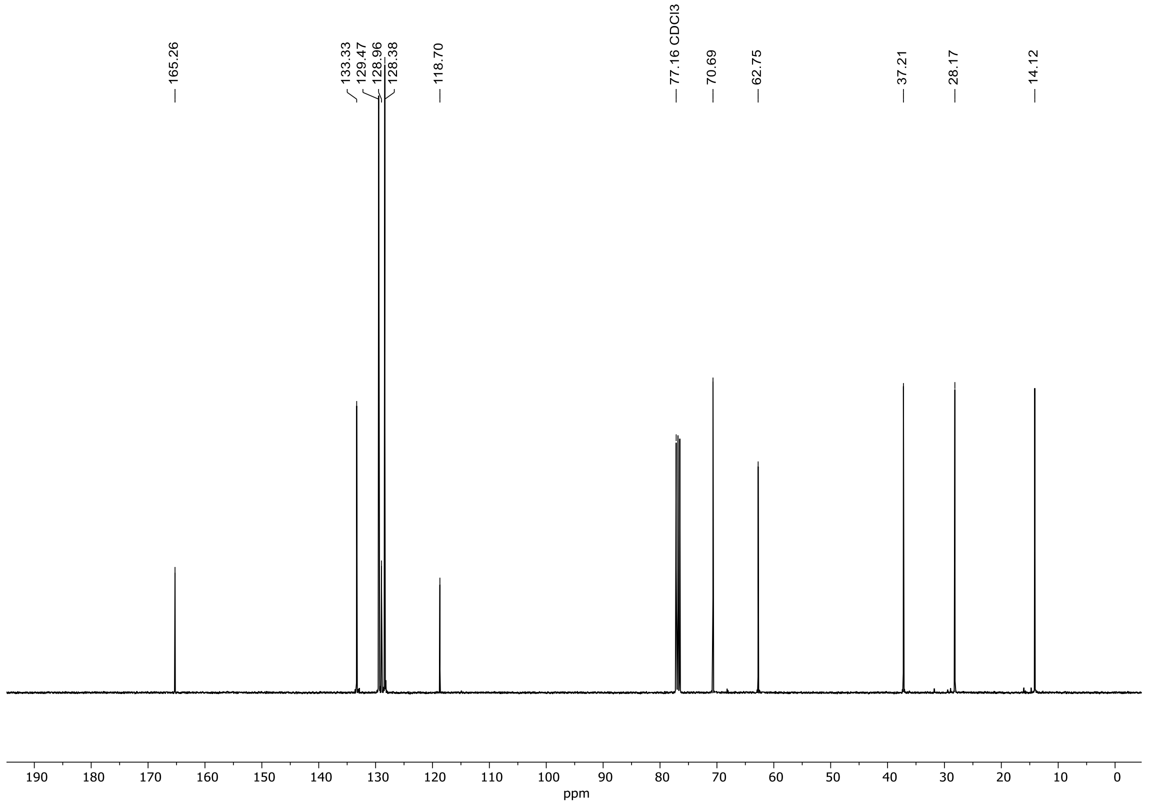

Diethyl 2-(3-(benzoyloxy)-2-bromo-2-methylpropyl)malonate (22)

^{1}H-NMR (400MHz)

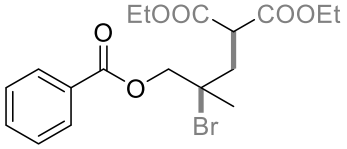

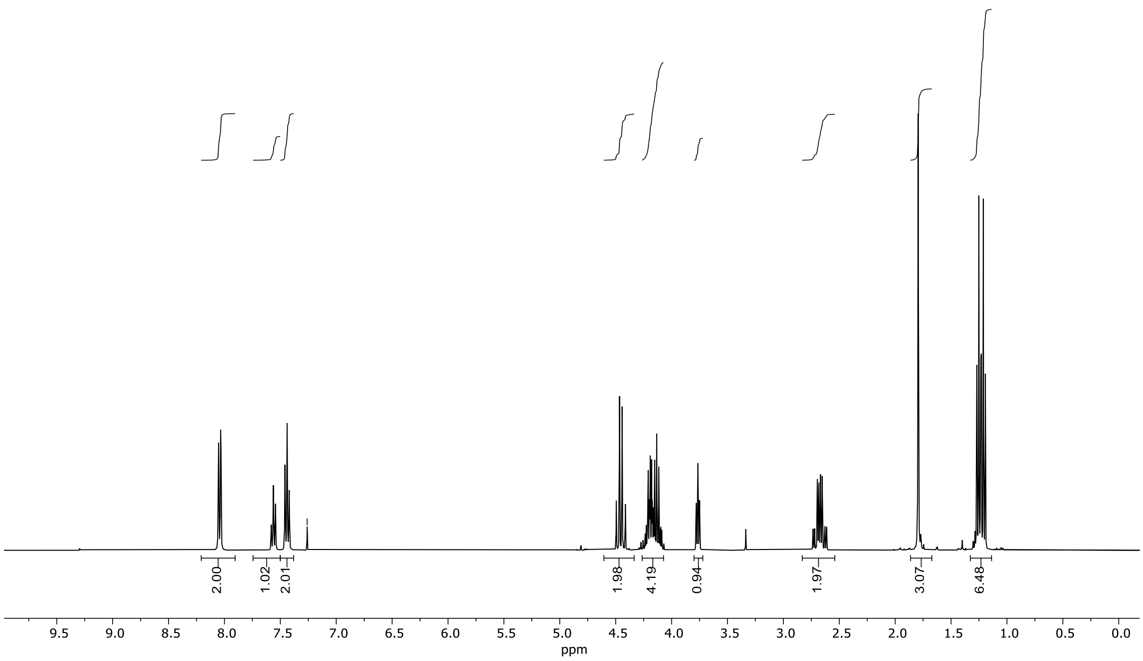

^{13}C-NMR (101MHz)

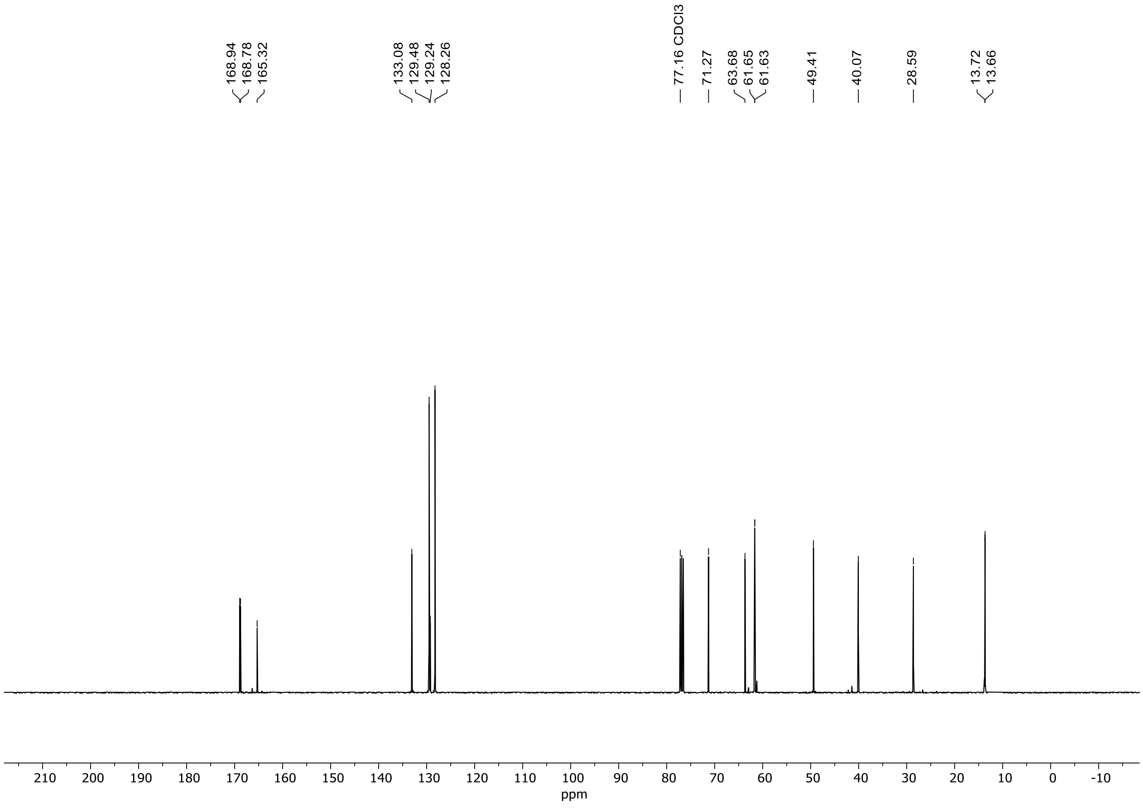

3-Bromo-2-(cyanomethyl)-3-methylbutyl benzoate (23)

^{1}H-NMR (400MHz)

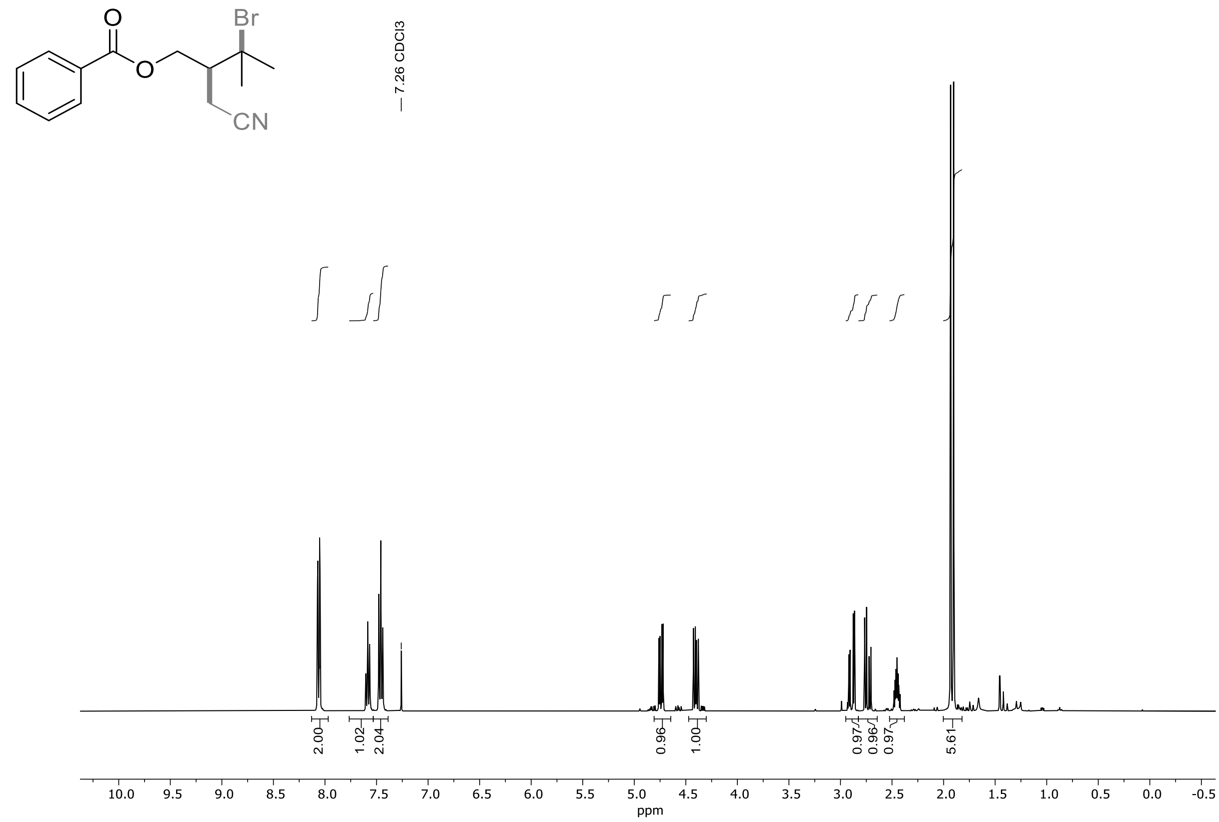

^{13}C-NMR (101MHz)

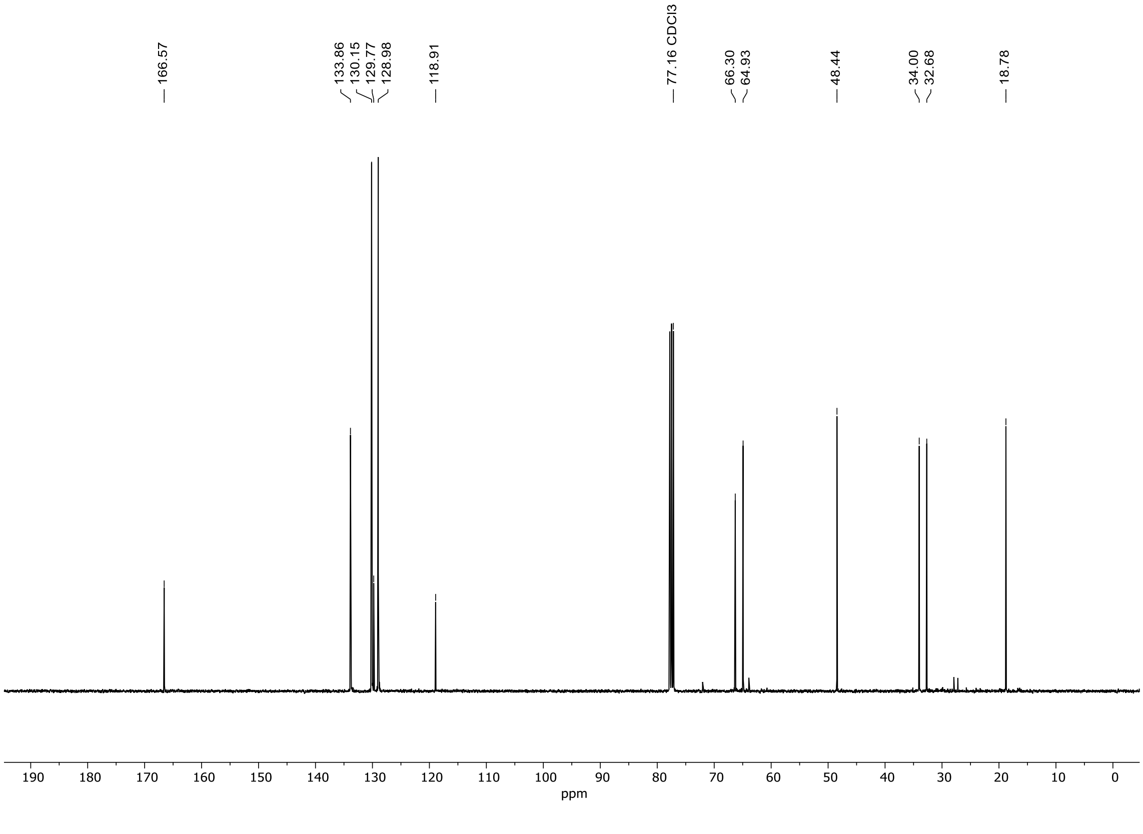

2-Bromo(phenyl)methyl-3,3,3-trichloropropyl benzoate (24)

^{1}H-NMR (400MHz)

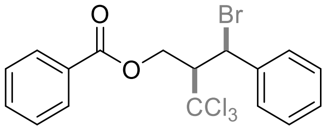

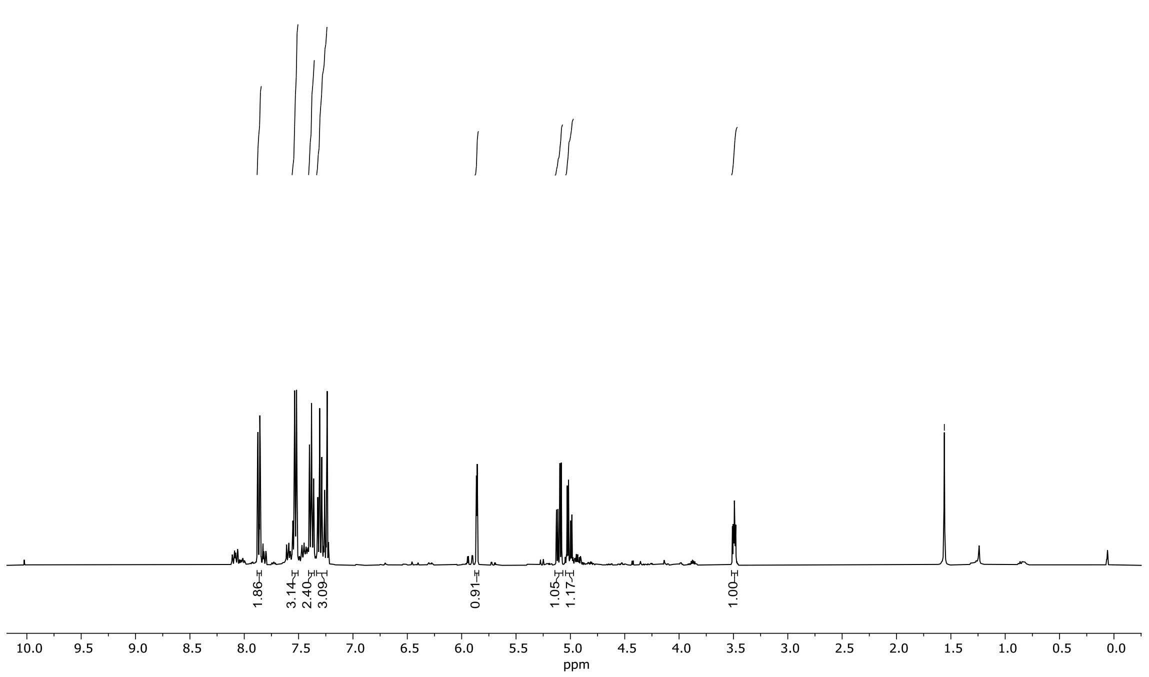

^{13}C-NMR (101MHz)

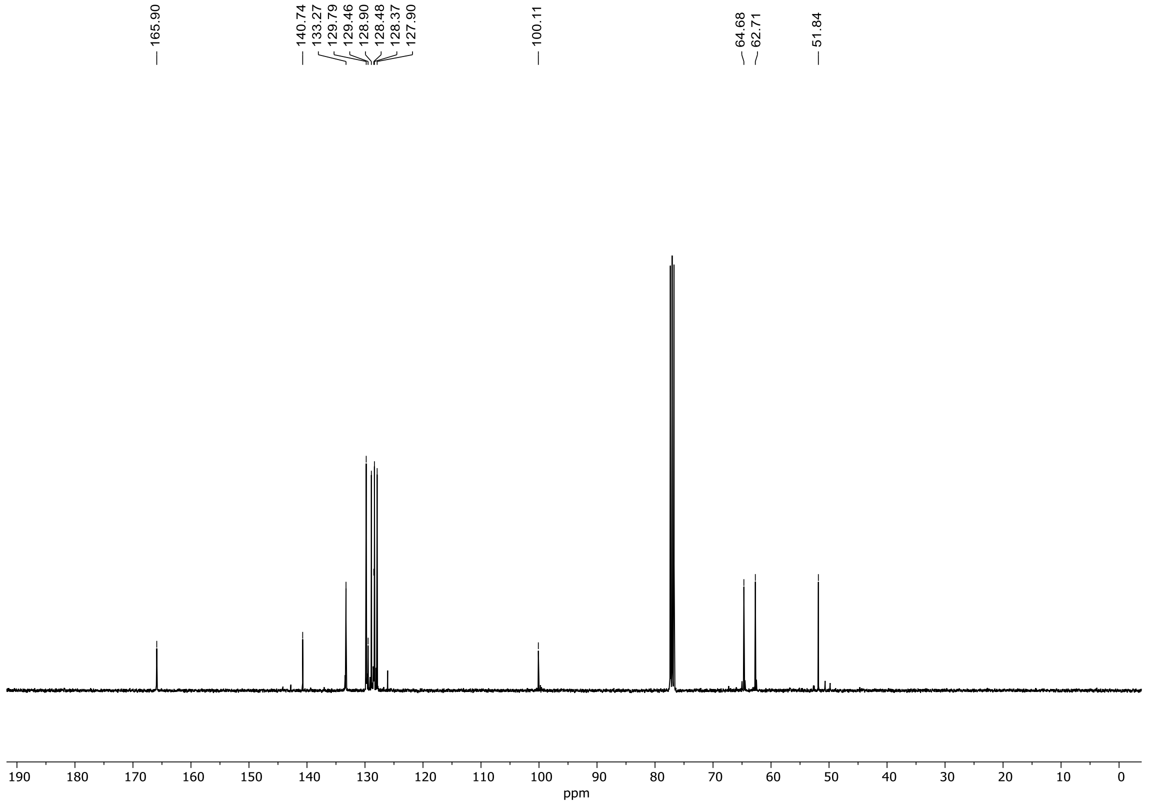

3-Bromo-2-(cyanomethyl)-3-phenylpropyl benzoate (25)

^{1}H-NMR (400MHz)

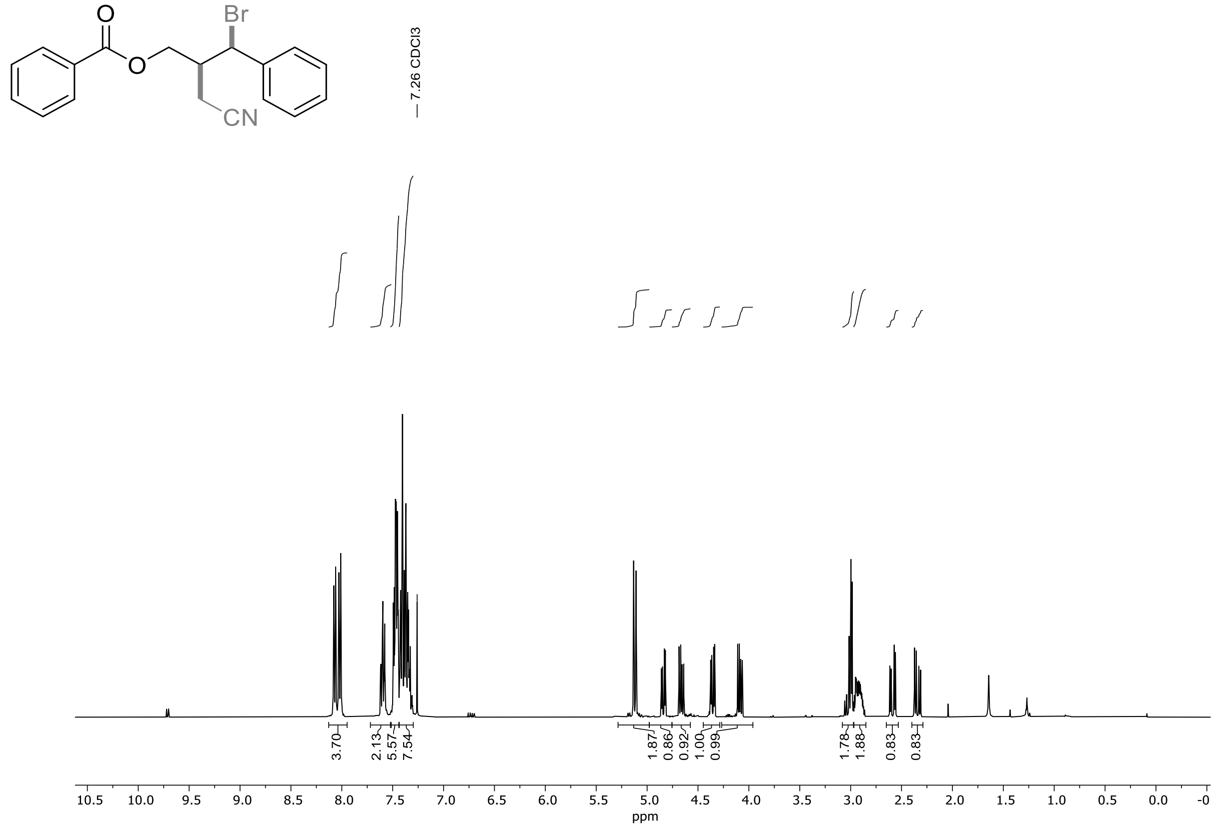

^{13}C-NMR (101MHz)

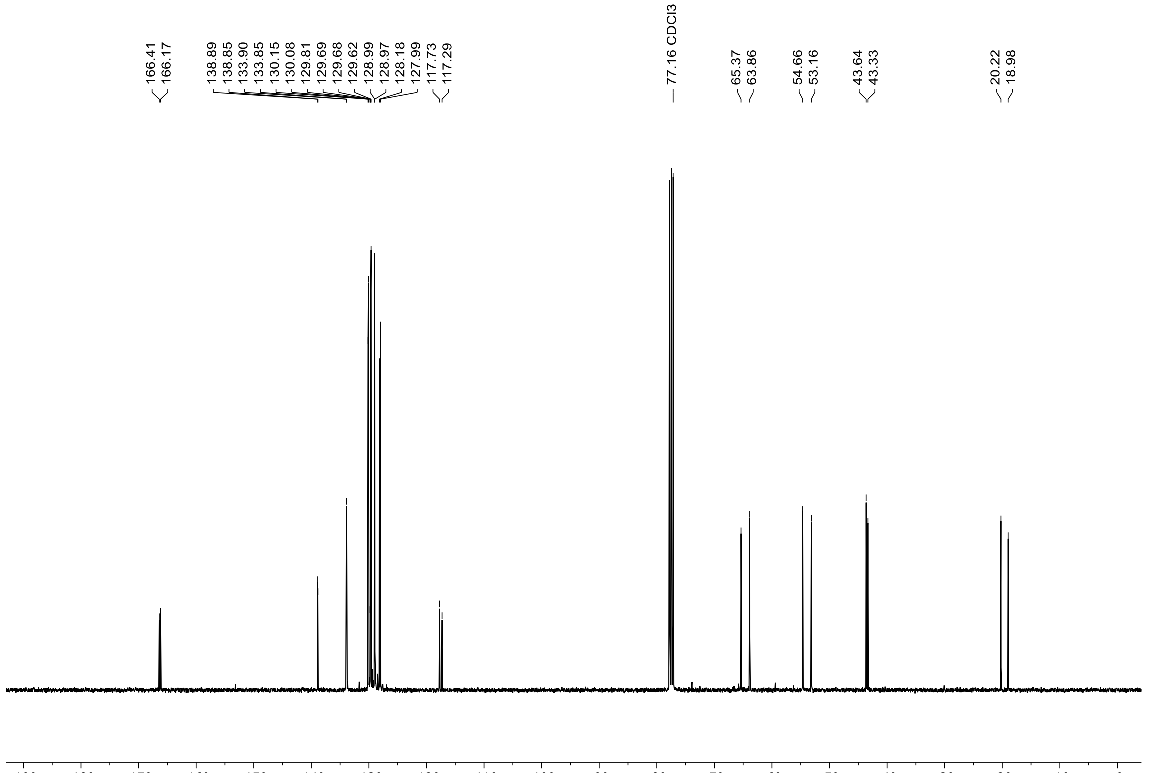

Mixture of 2-bromo-4-cyano-3-methylbutyl benzoate (26) and 3-bromo-2-(cyanomethyl)butyl benzoate (26')

^{1}H-NMR (400MHz)

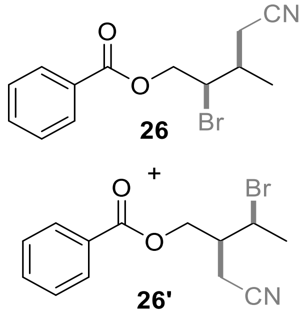

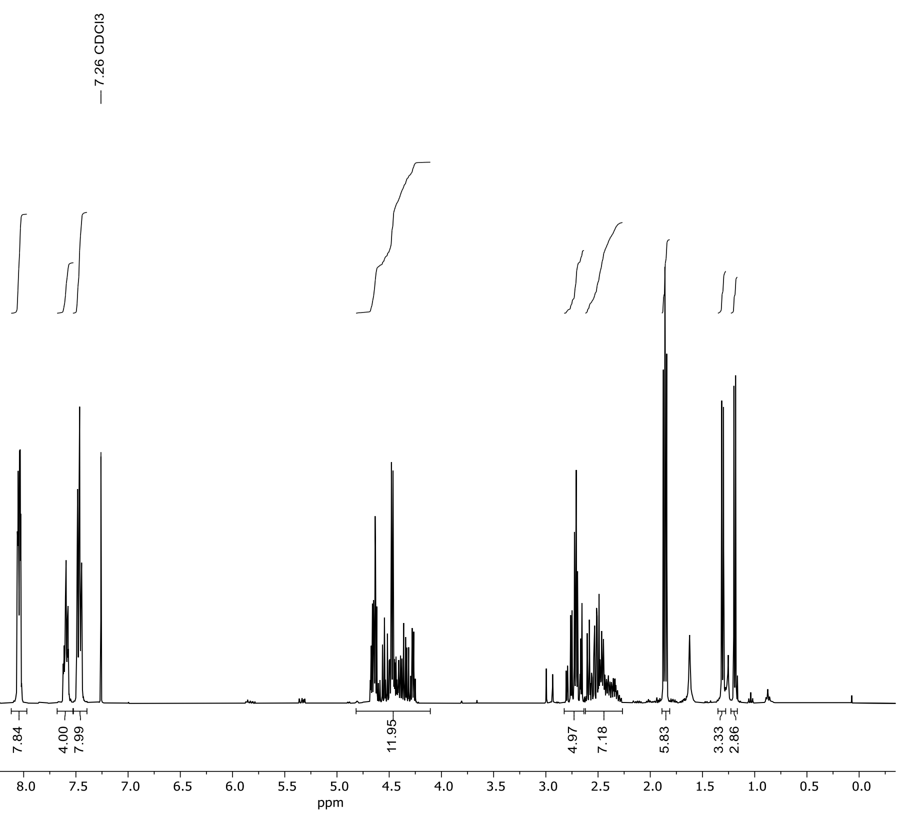

^{13}C-NMR (101MHz)

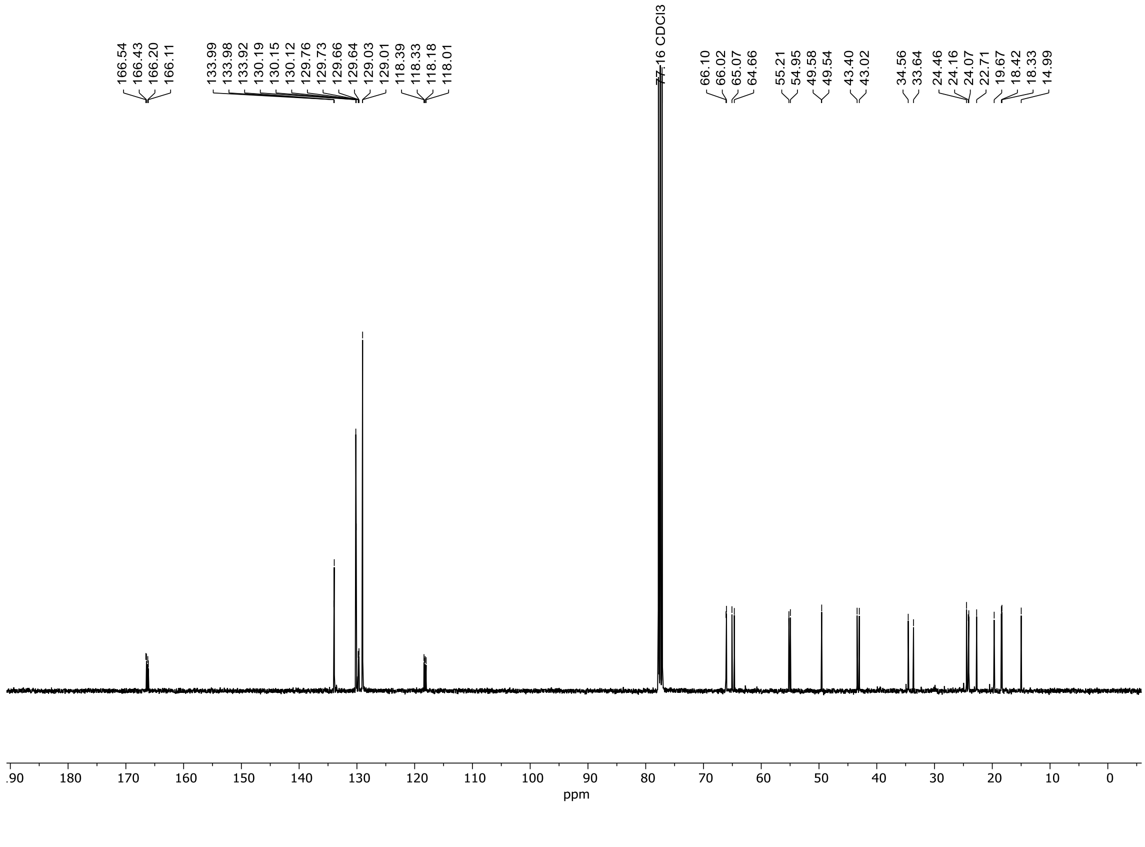

N-(4-Bromo-6,6,6-trichlorohexyl)benzamide (27)

^{1}H-NMR (400MHz)

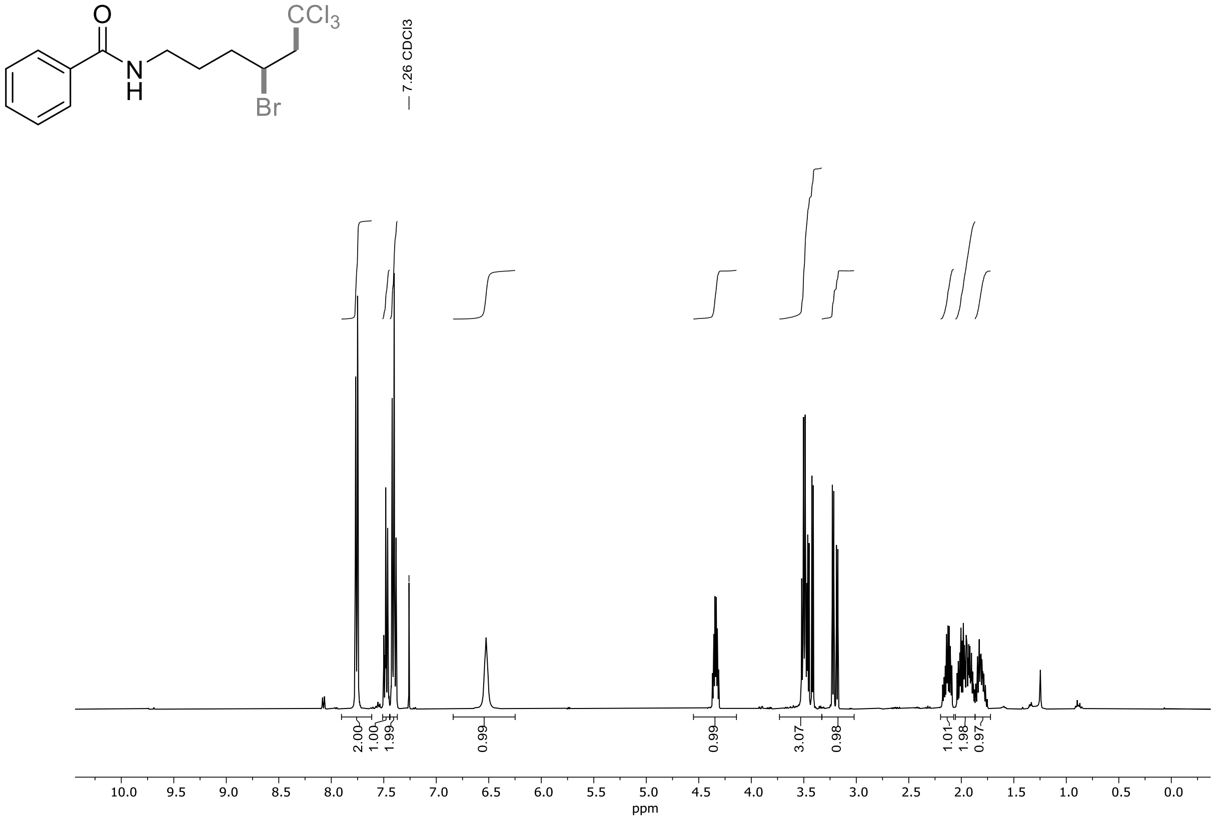

^{13}C-NMR (101MHz)

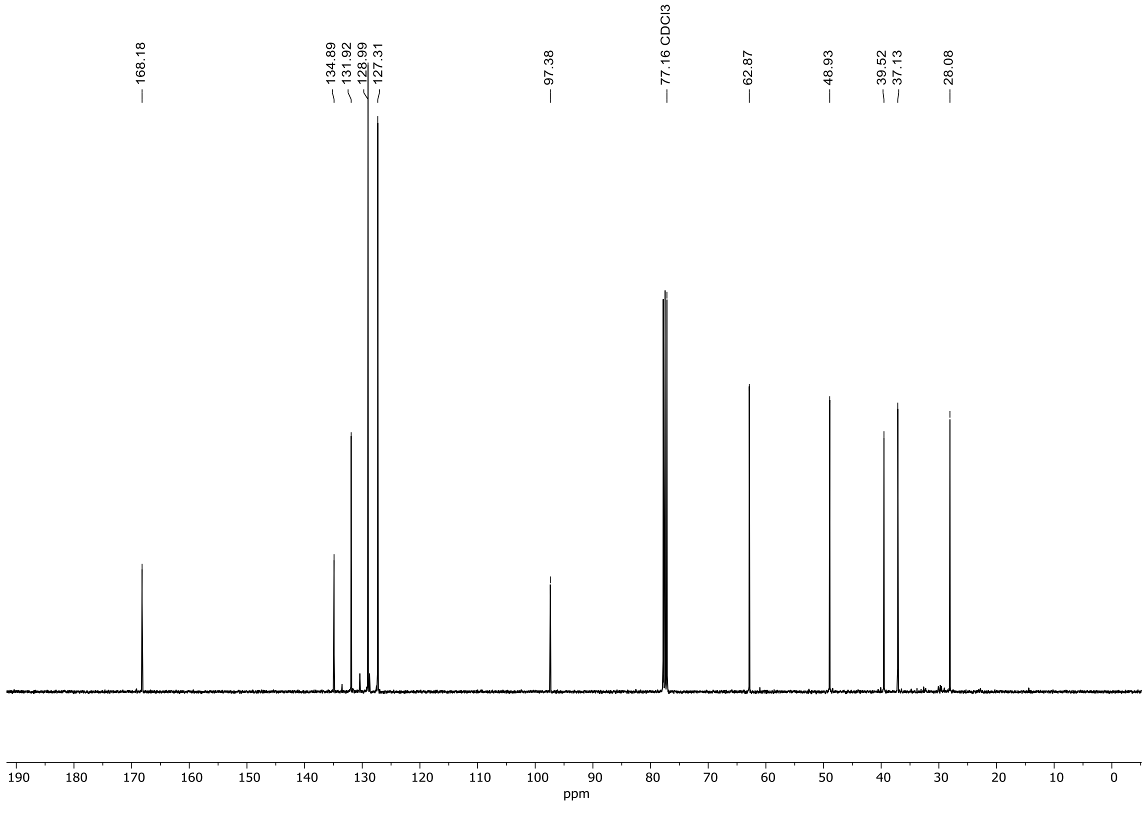

N-(4-Bromo-6-cyanohexyl)benzamide (28)

^{1}H-NMR (400MHz)

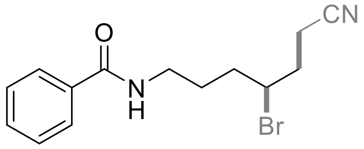

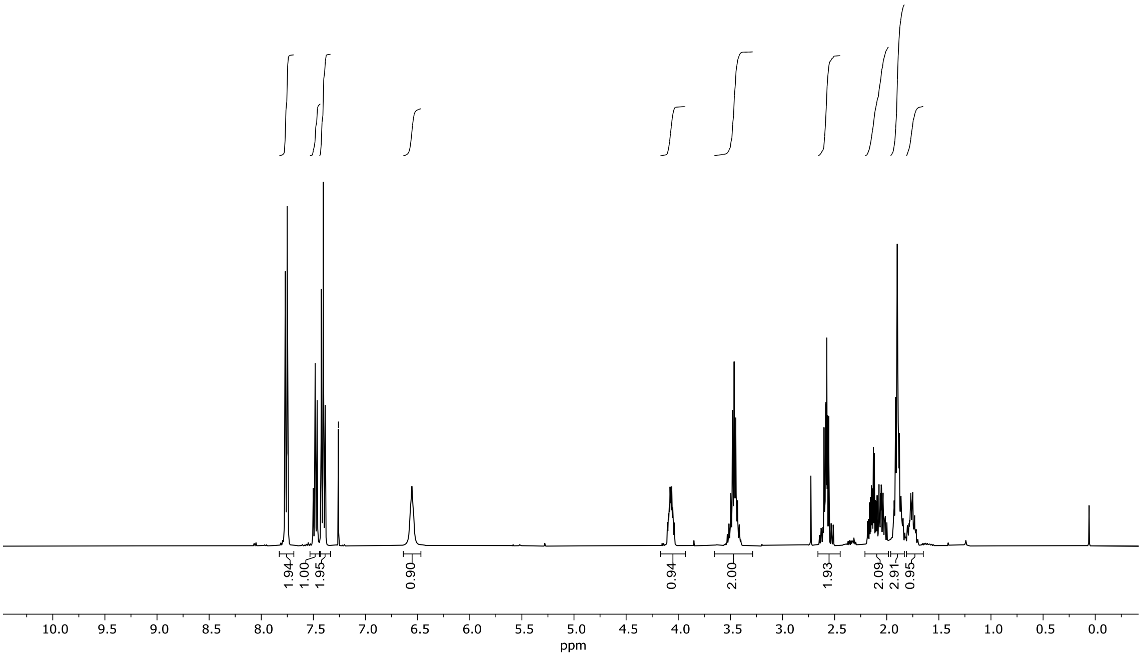

^{13}C-NMR (101MHz)

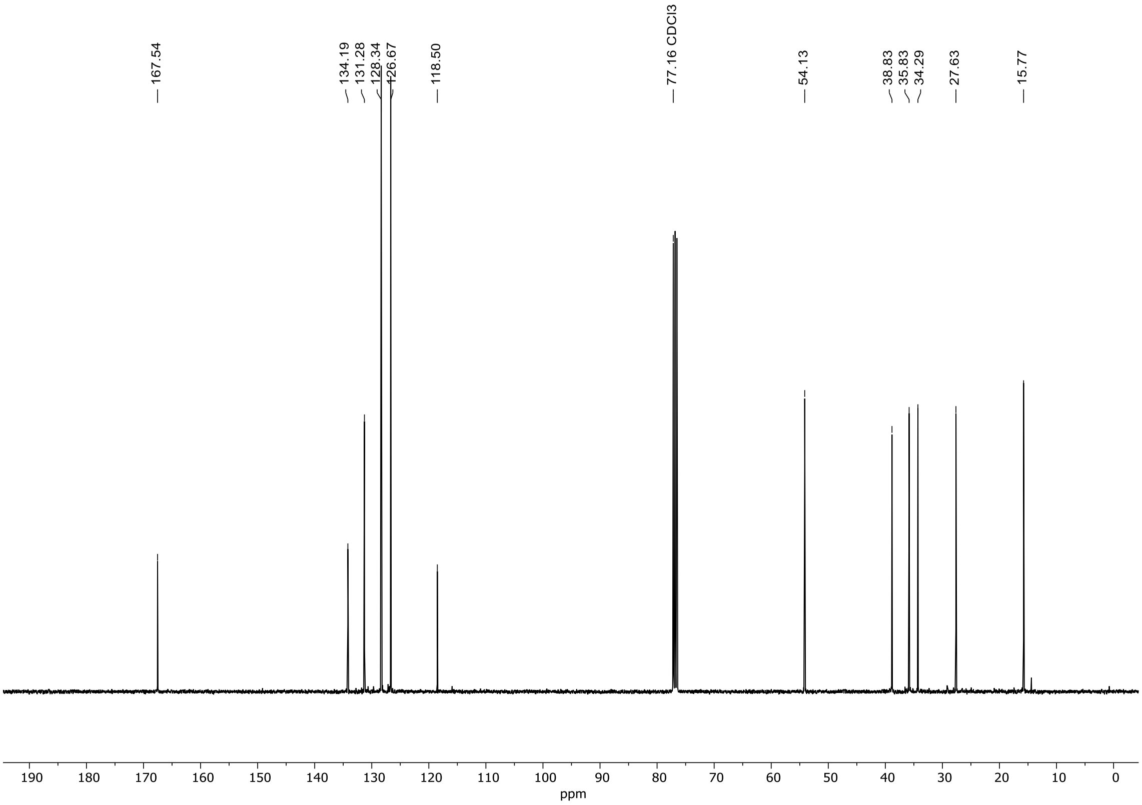

Diethyl 2-(5-benzamido-2-bromopentyl)malonate (29)

^{1}H-NMR (400MHz)

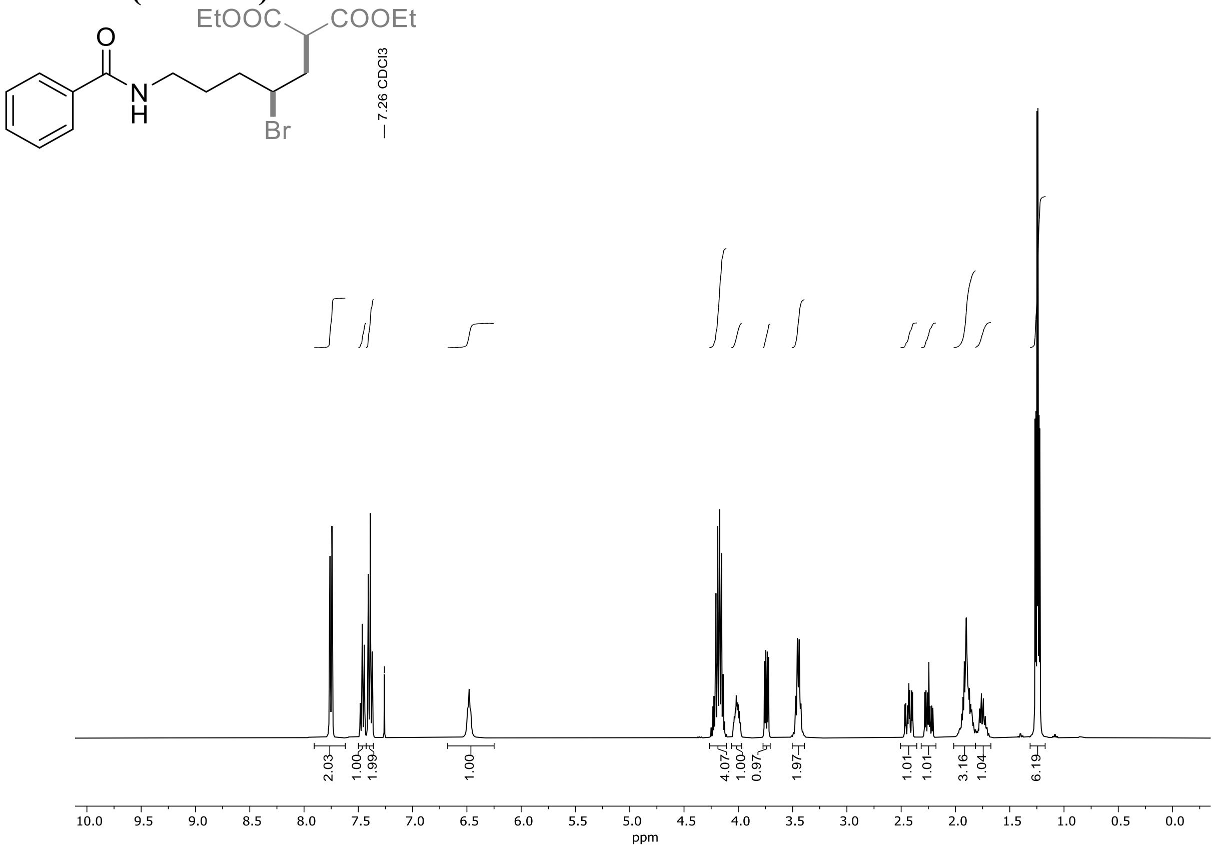

^{13}C-NMR (101MHz)

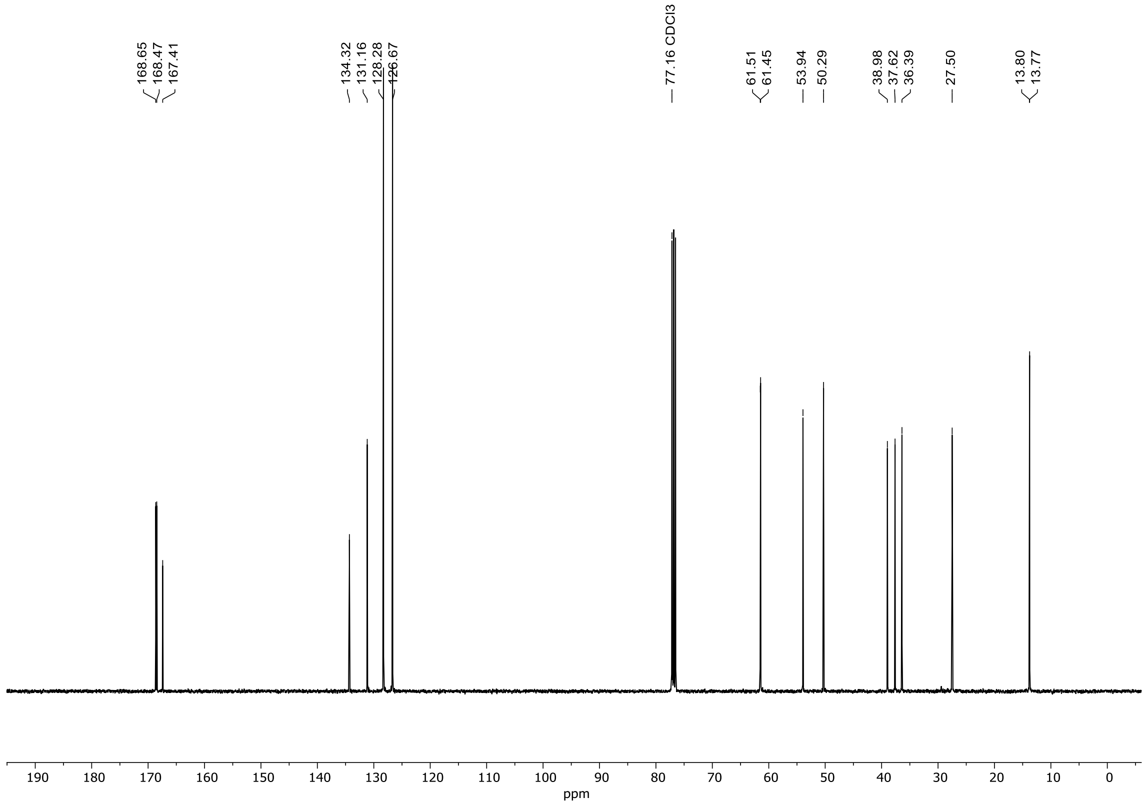

Ethyl 7- benzamido-4-bromo-2,2-difluoroheptanoate (30)

^{1}H-NMR (400MHz)

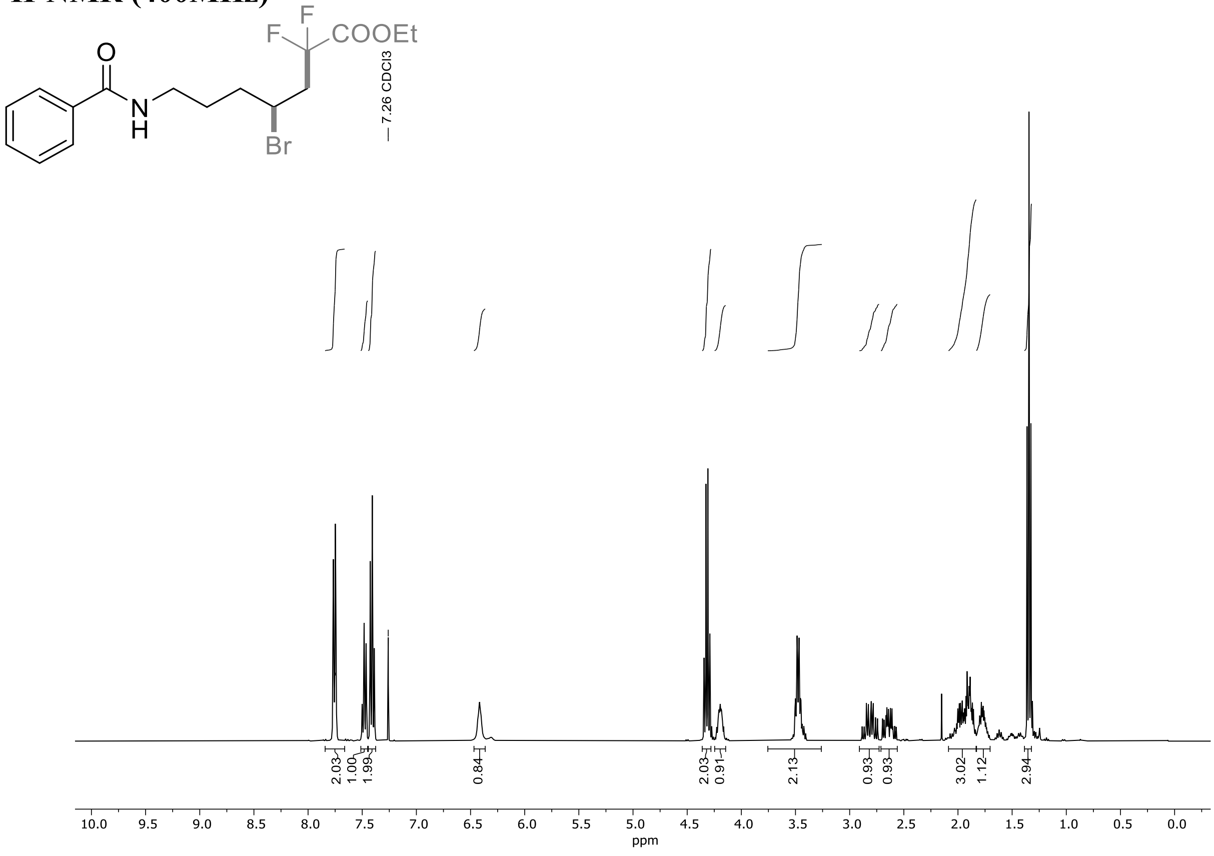

^{13}C-NMR (101MHz)

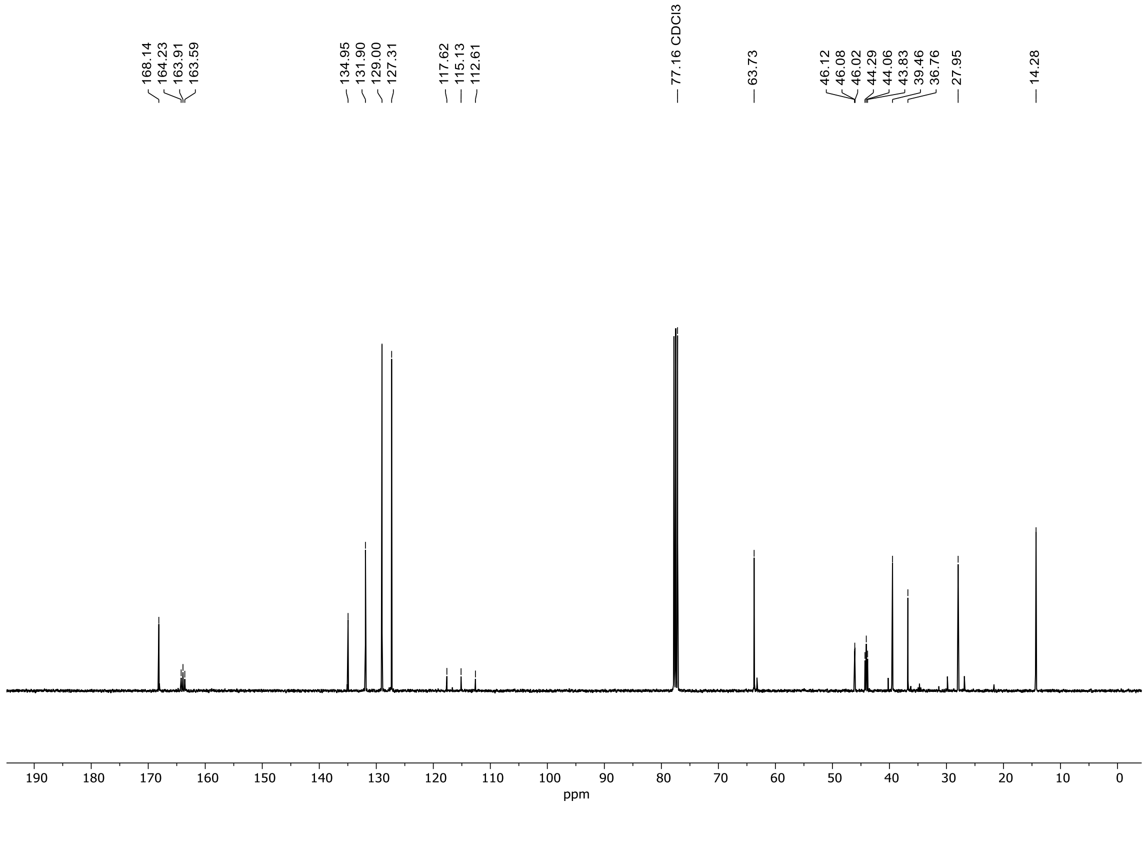

N-(2-Bromo-4,4,4-trichlorobutyl)benzamide (31)

^{1}H-NMR (400MHz)

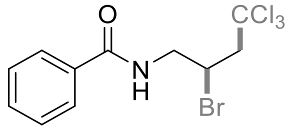

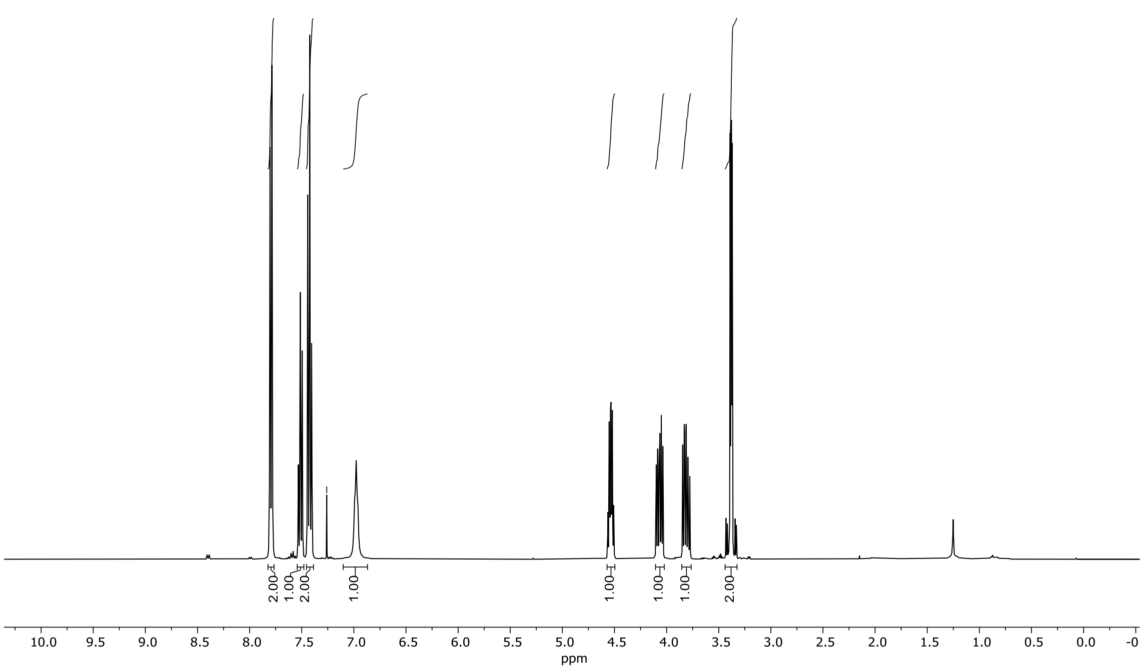

^{13}C-NMR (101MHz)

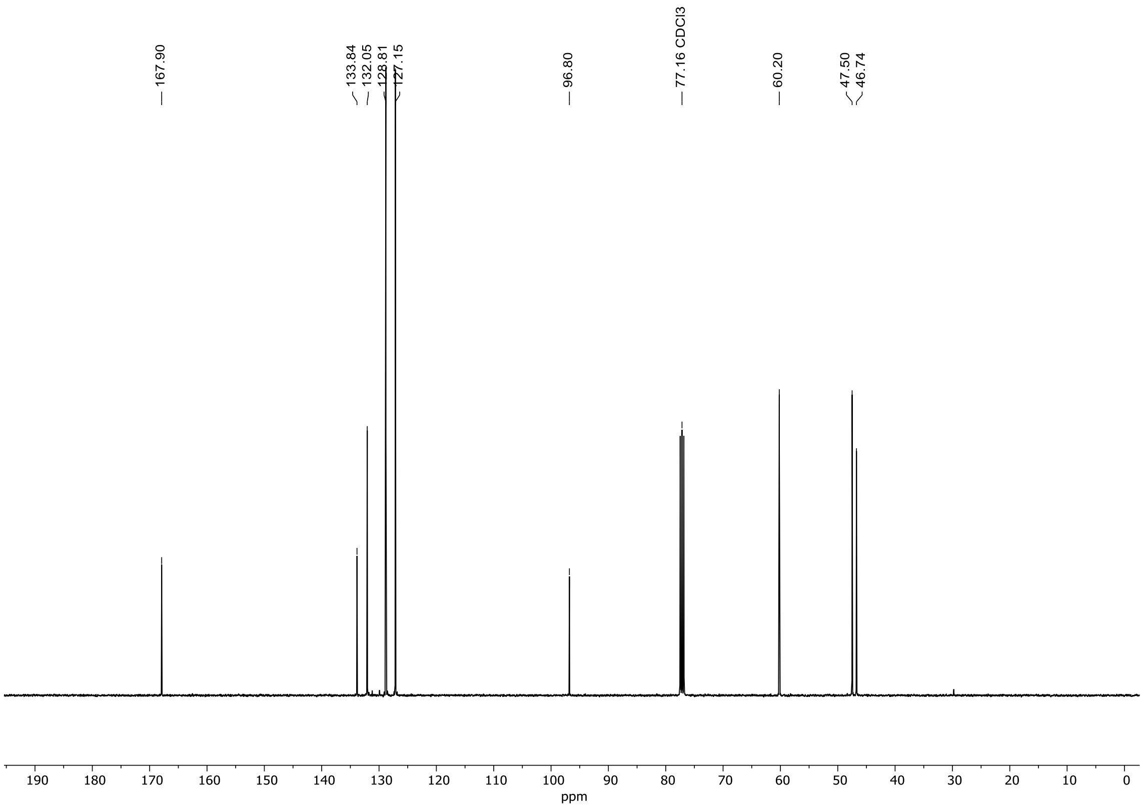

Diethyl 2-(3-benzamido-2-bromopropyl)malonate (32)

^{1}H-NMR (400MHz)

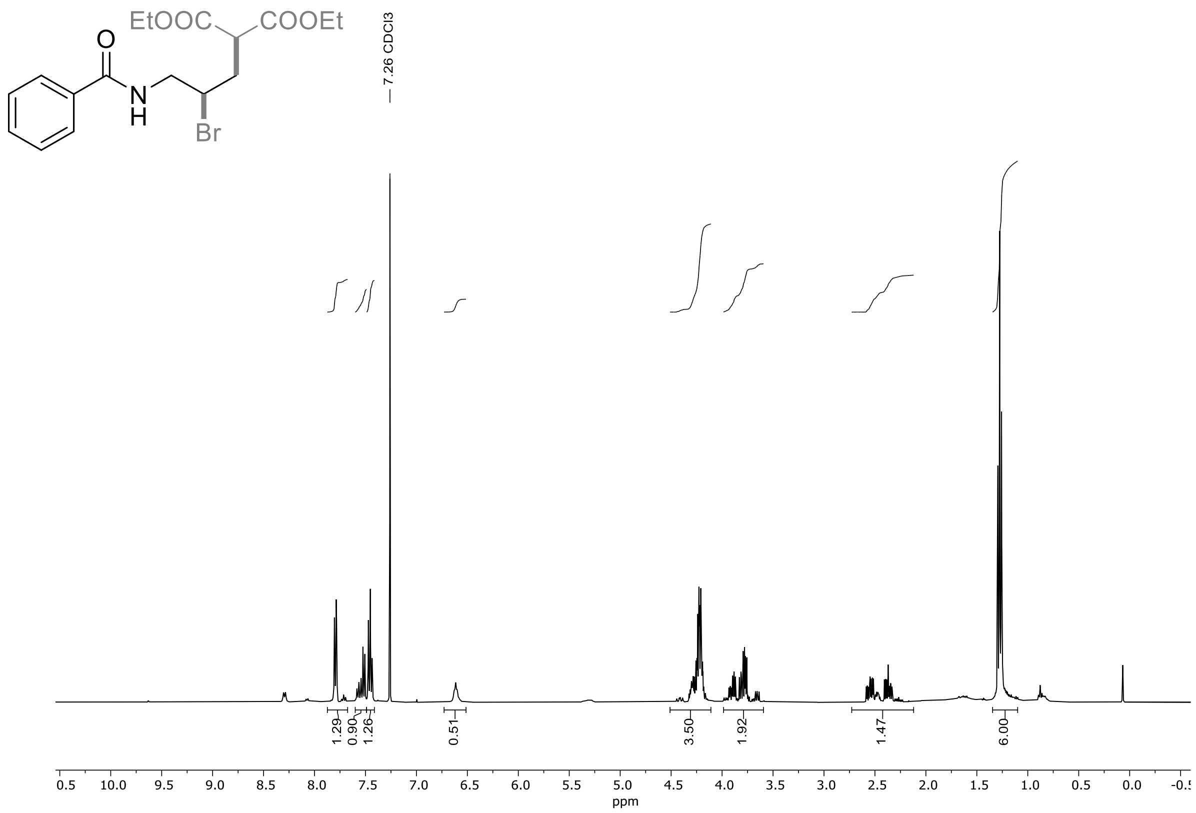

^{13}C-NMR (101MHz)

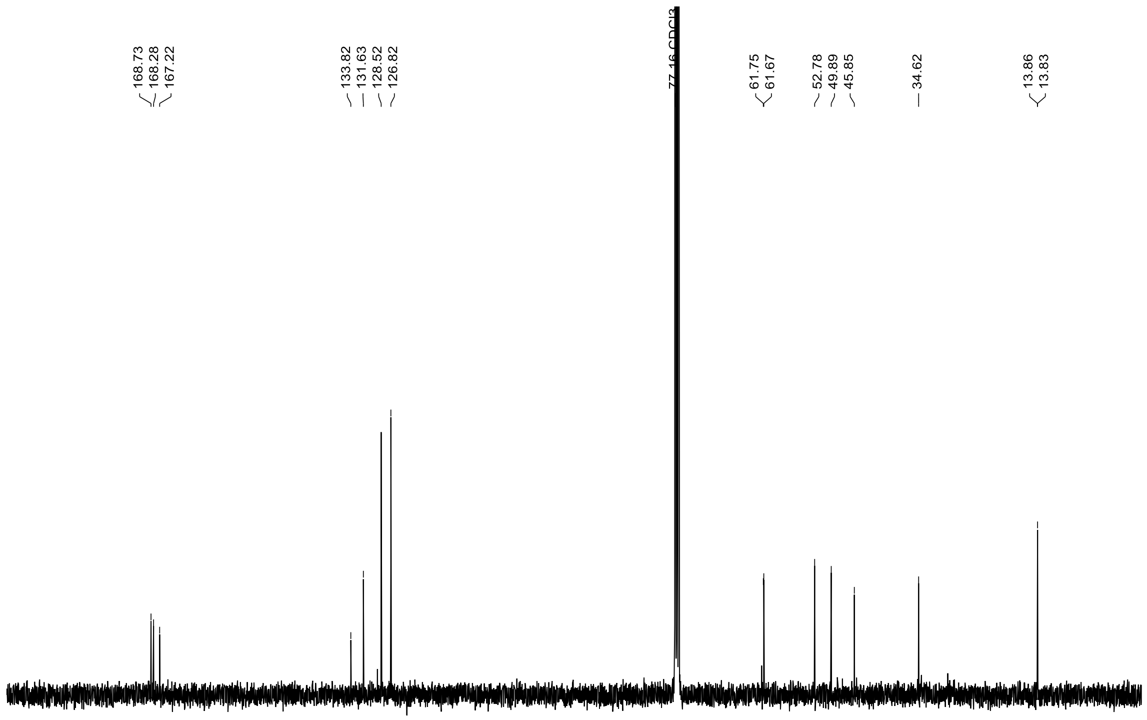

N-(2-Bromo-4,4,4-trichlorobutyl)-1-naphtamide (33)

^{1}H-NMR (400MHz)

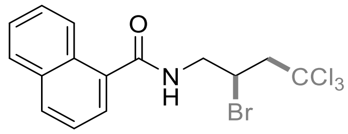

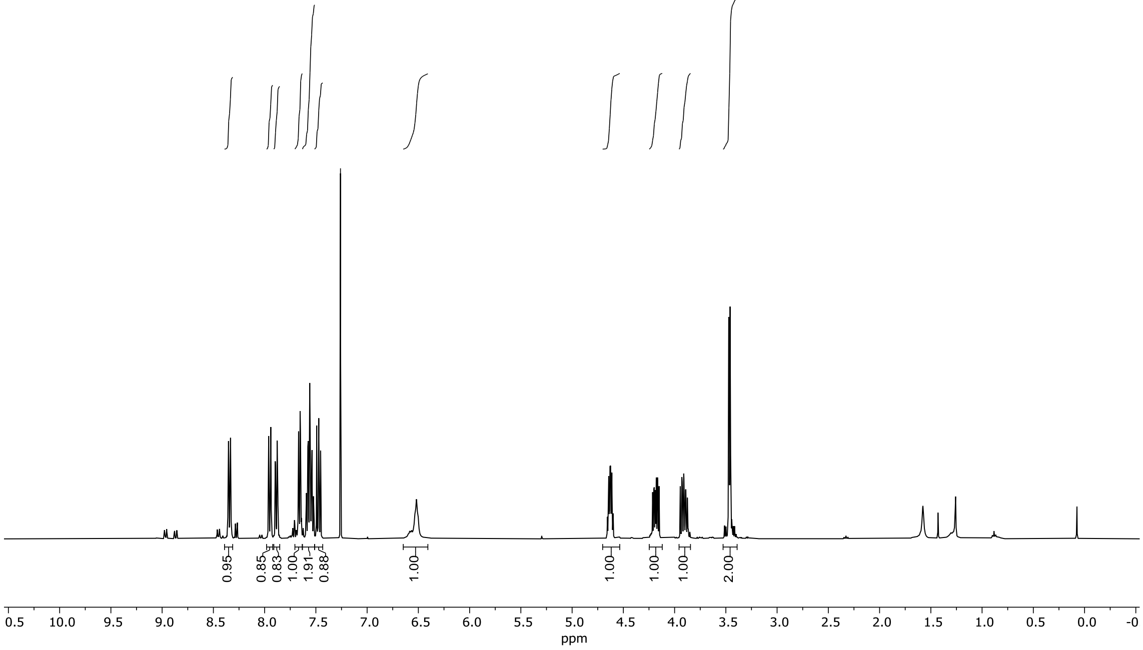

^{13}C-NMR (101MHz)

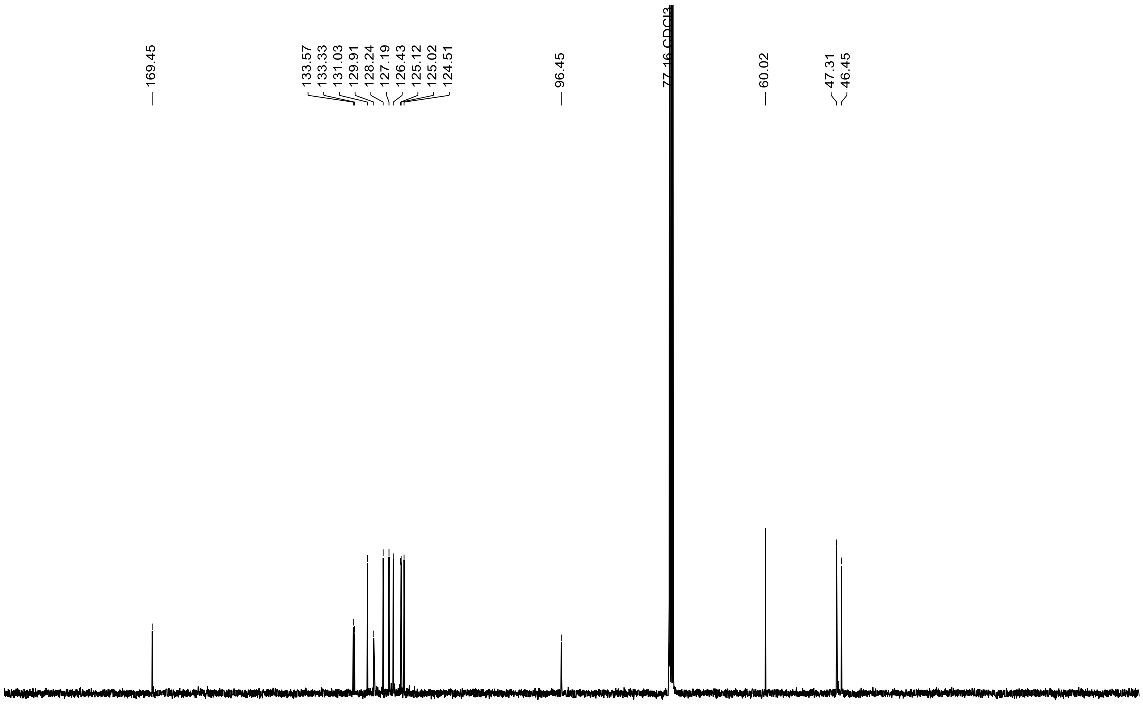

Diethyl 2-(3-(1-naphtamido)-2-bromoprpyl)malonate (34)

^{1}H-NMR (400MHz)

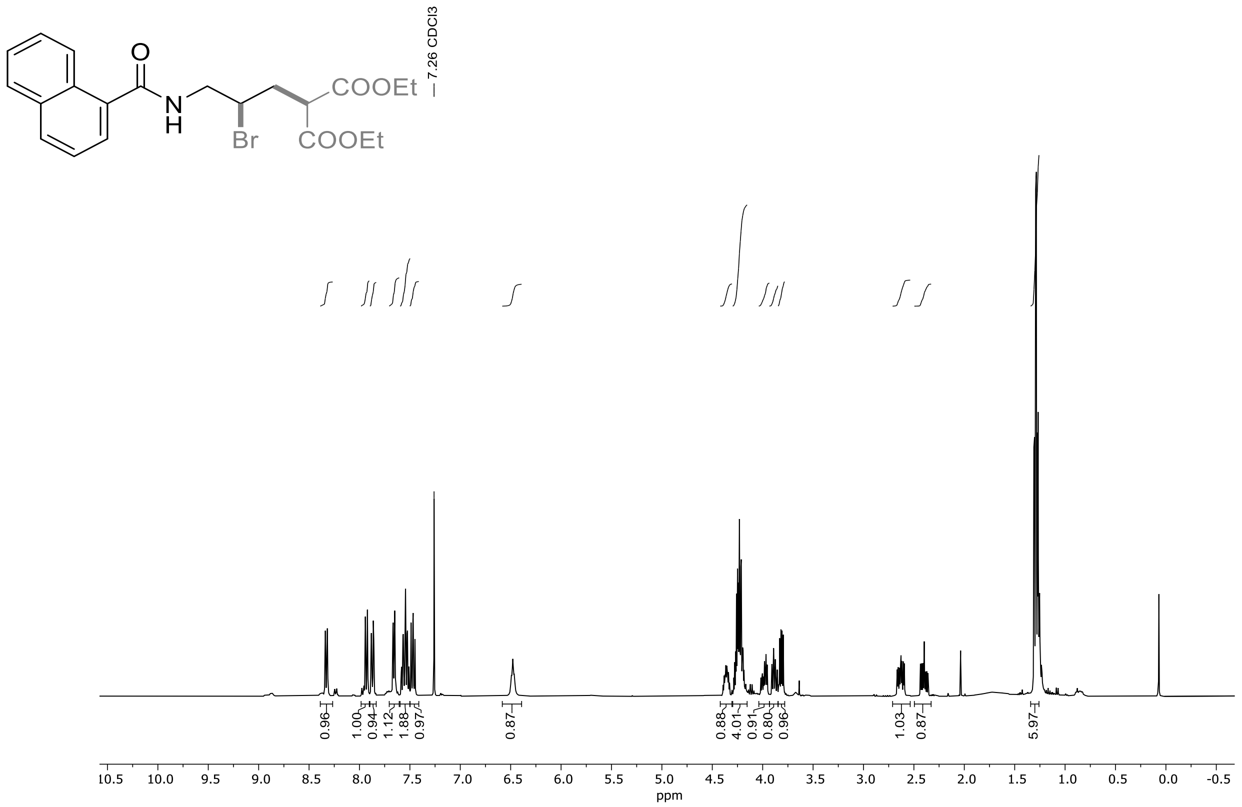

^{13}C-NMR (101MHz)

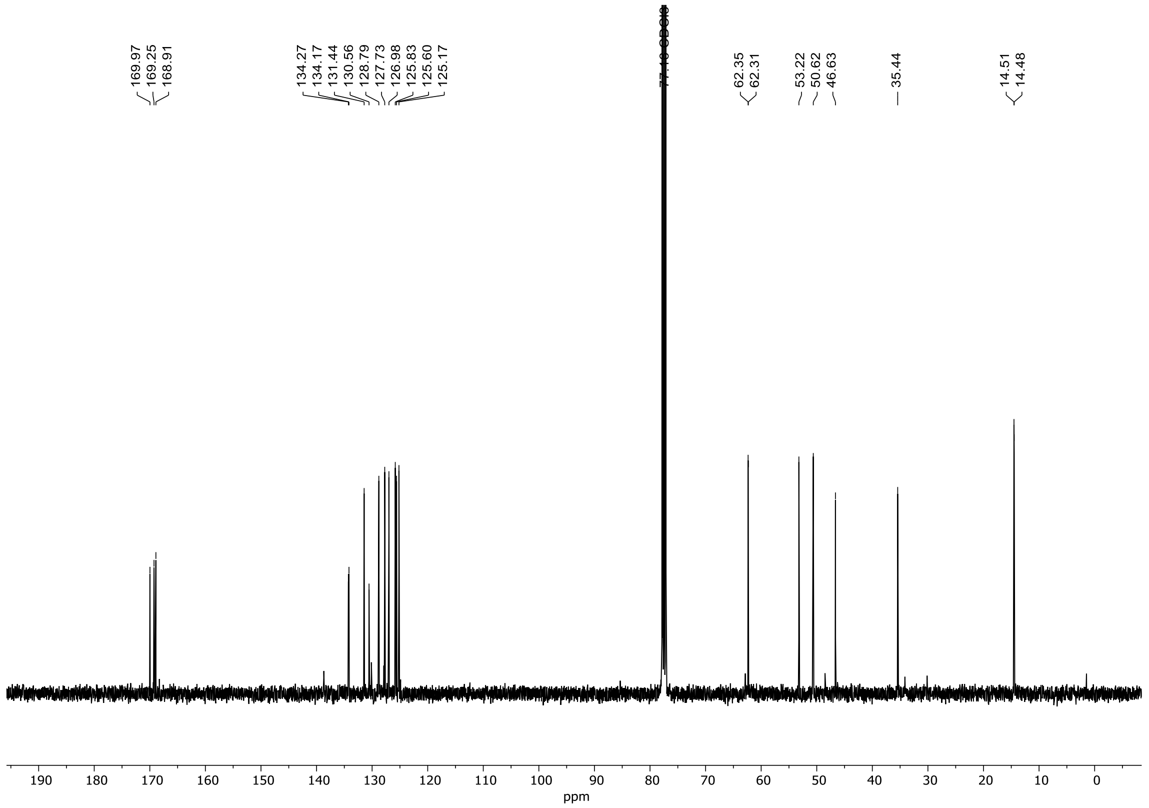

N-(2-Bromo-4,4,4-trichlorobutyl)-4-methylbenzenesulfonamide (35)

^{1}H-NMR (400MHz)

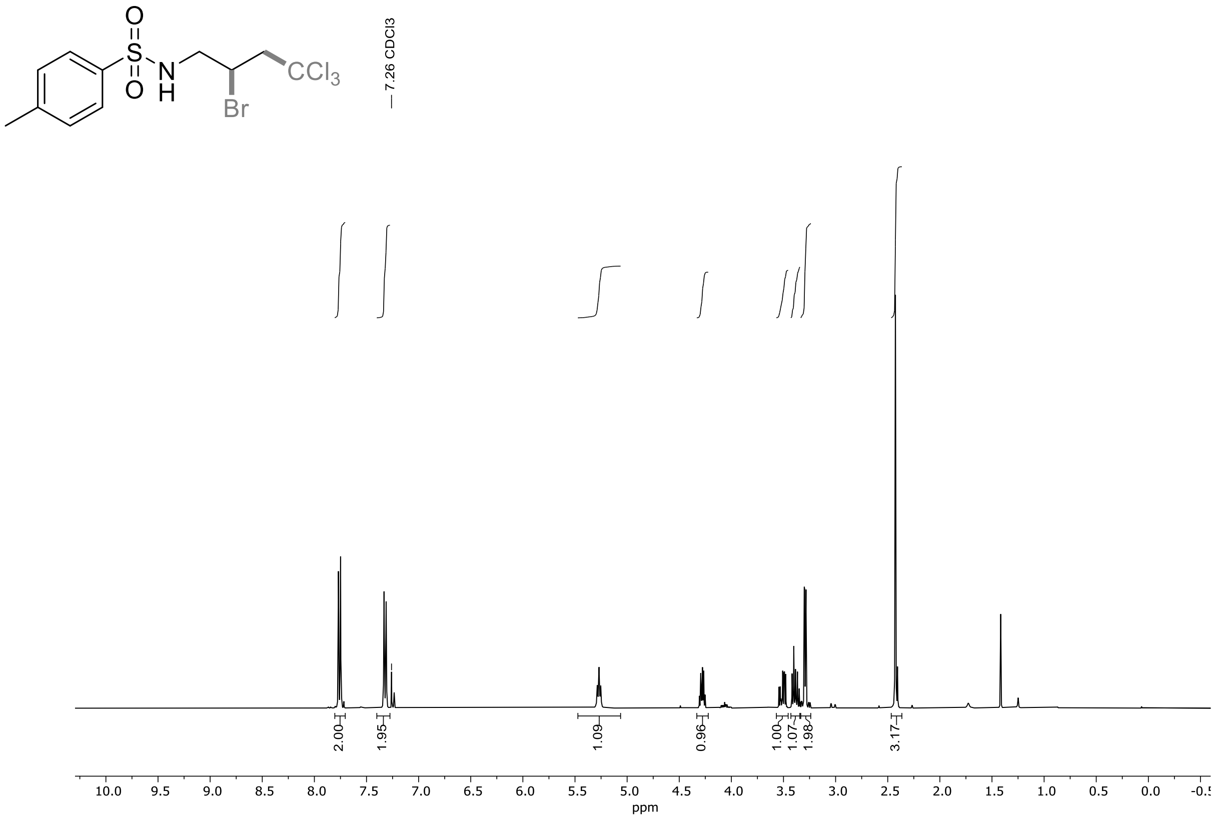

^{13}C-NMR (101MHz)

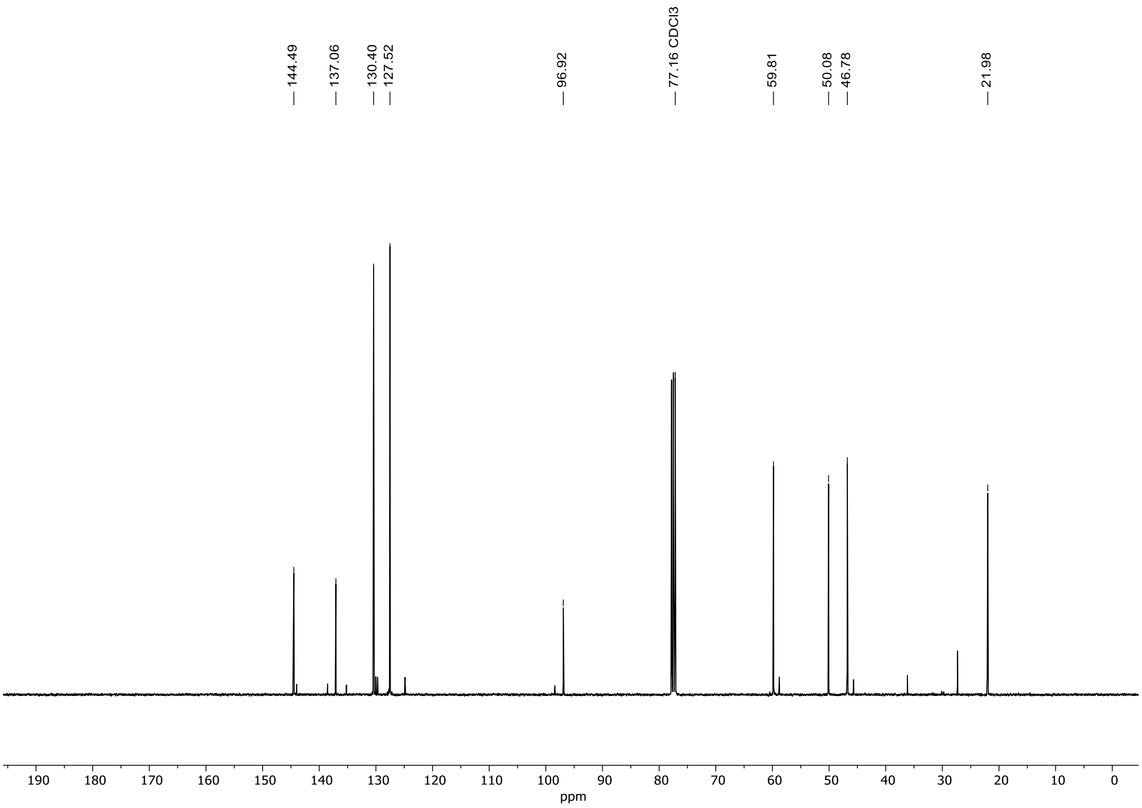

Diethyl 2-(2-bromo-3-((4-methylphenyl)sulfonamido)propyl) malonate (36)

^{1}H-NMR (400MHz)

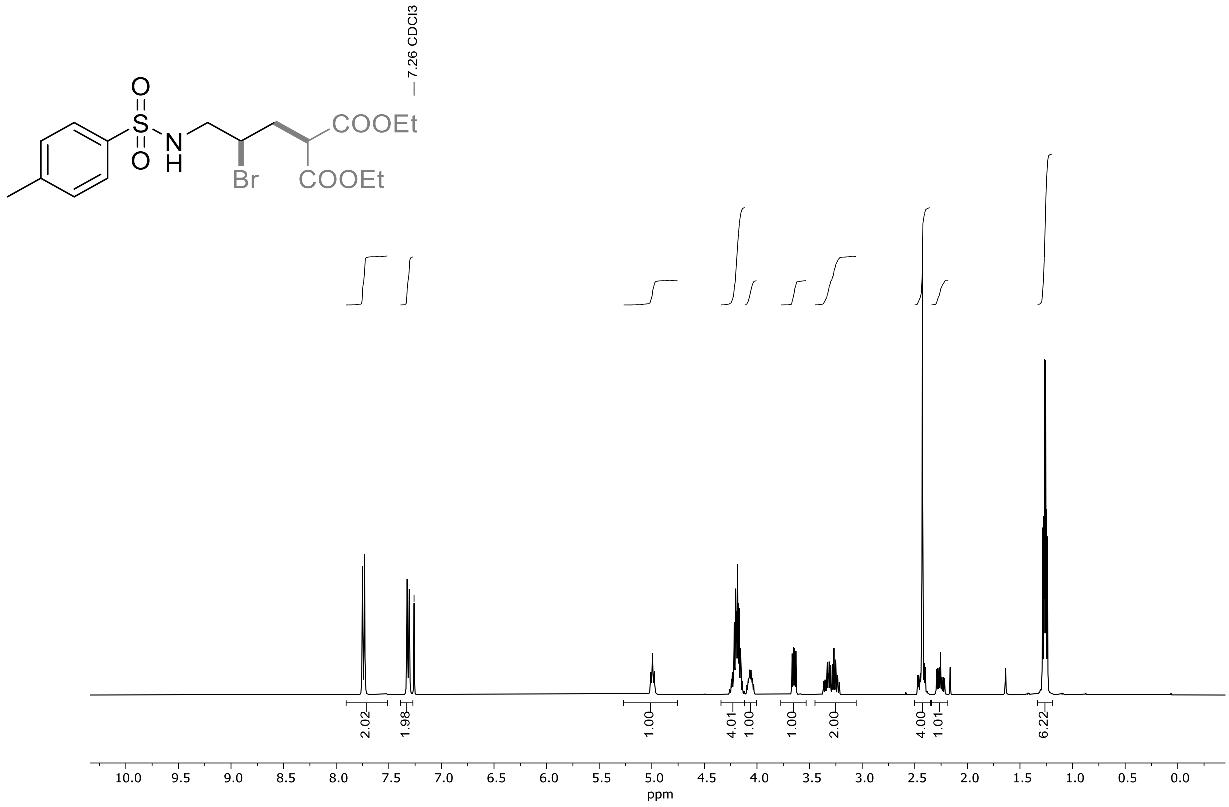

^{13}C-NMR (101MHz)

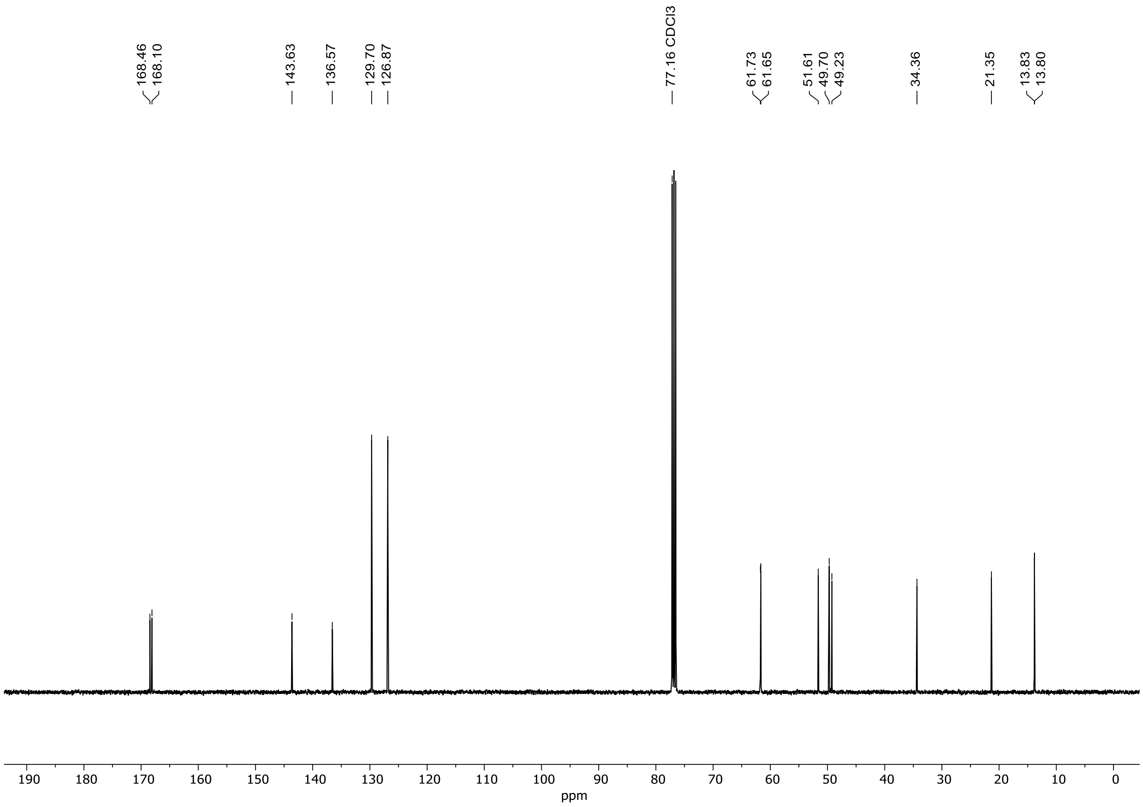

1-(2-Bromo-4,4,4-trichlorobutyl)pyrrolidin-2-one (37)

^{1}H-NMR (400MHz)

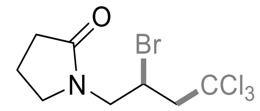

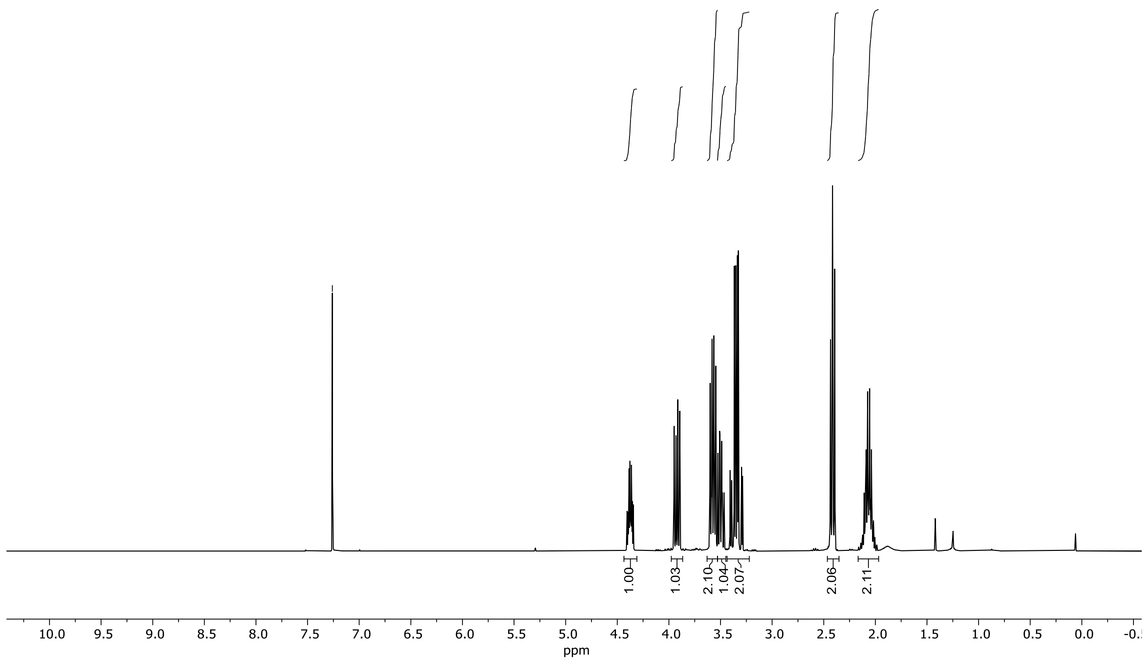

^{13}C-NMR (101MHz)

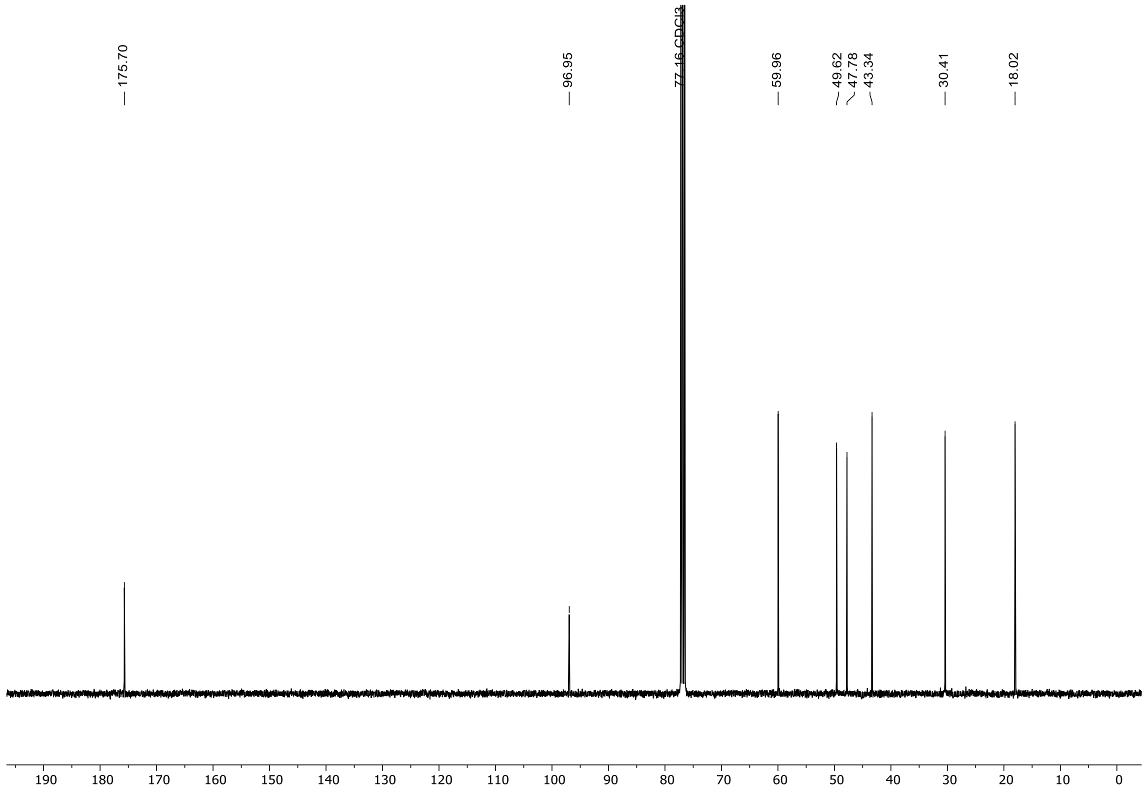

Diethyl 2-(2-bromo-3-(2-oxopyrrolidin-1-yl)propyl)malonate (38)

^{1}H-NMR (400MHz)

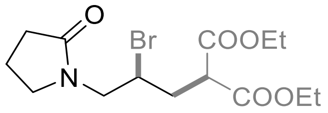

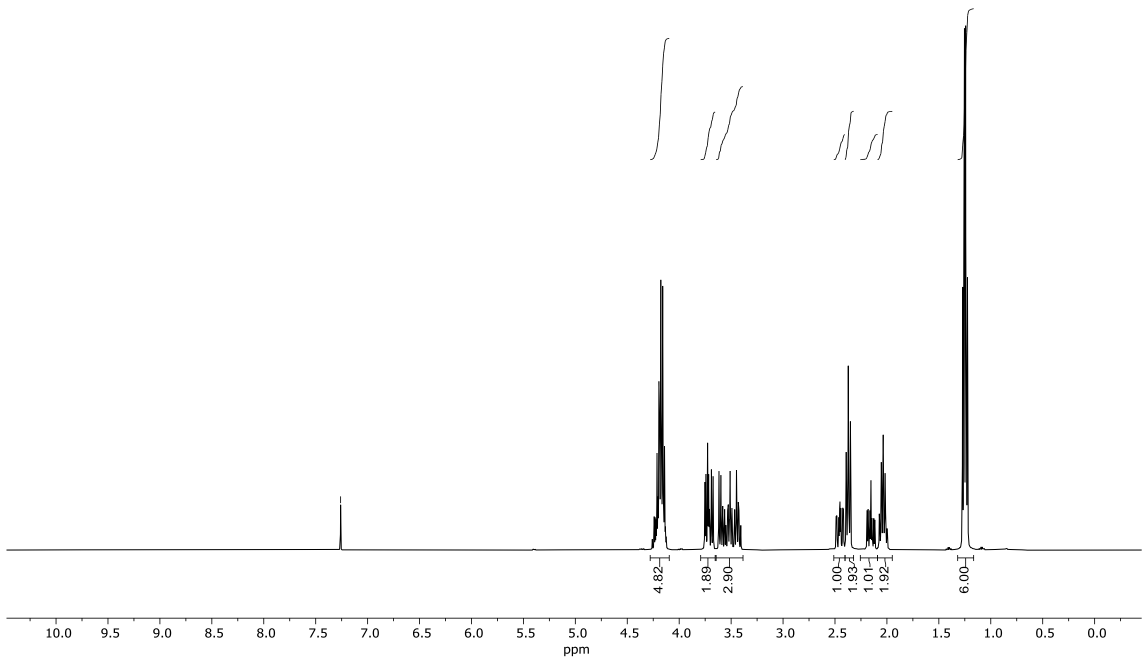

^{13}C-NMR (101MHz)

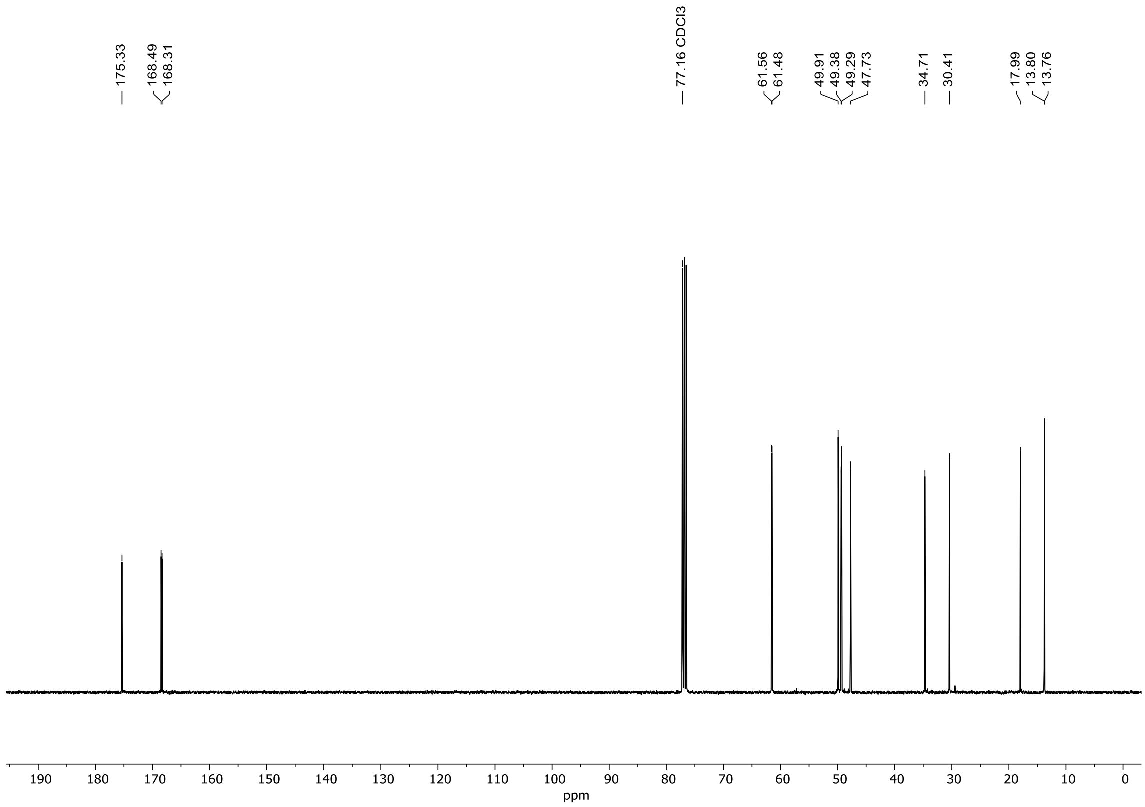

N-(2-Bromo-4,4,4-trichloro-2-methylbutyl)benzamide (39)

^{1}H-NMR (400MHz)

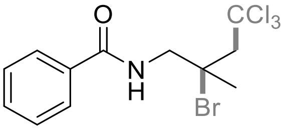

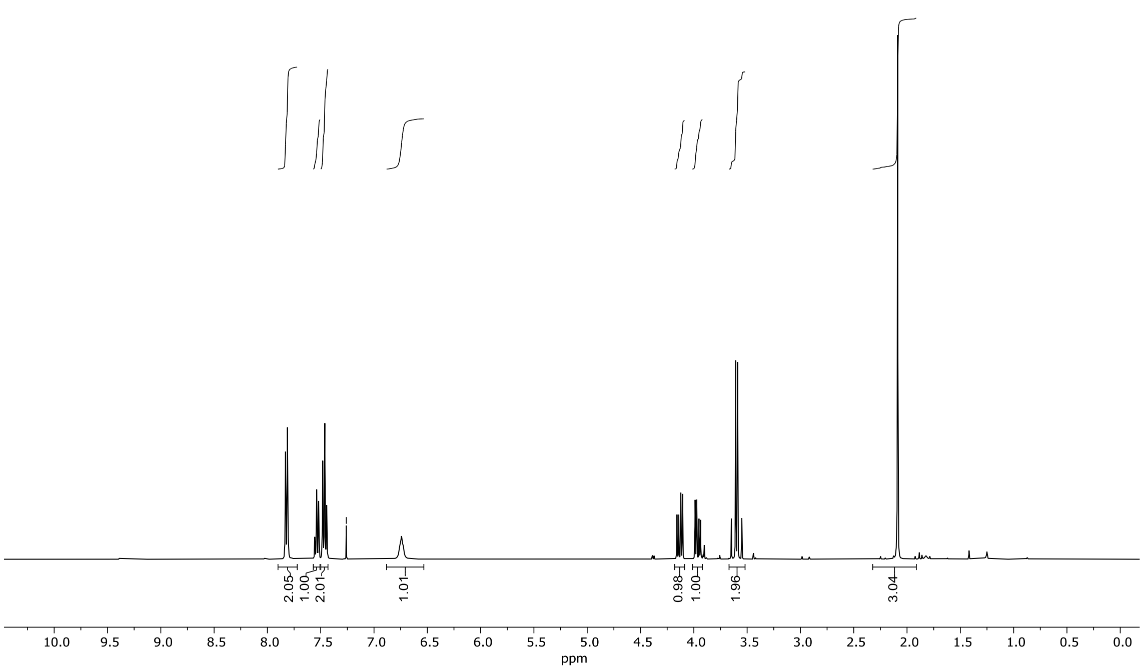

^{13}C-NMR (101MHz)

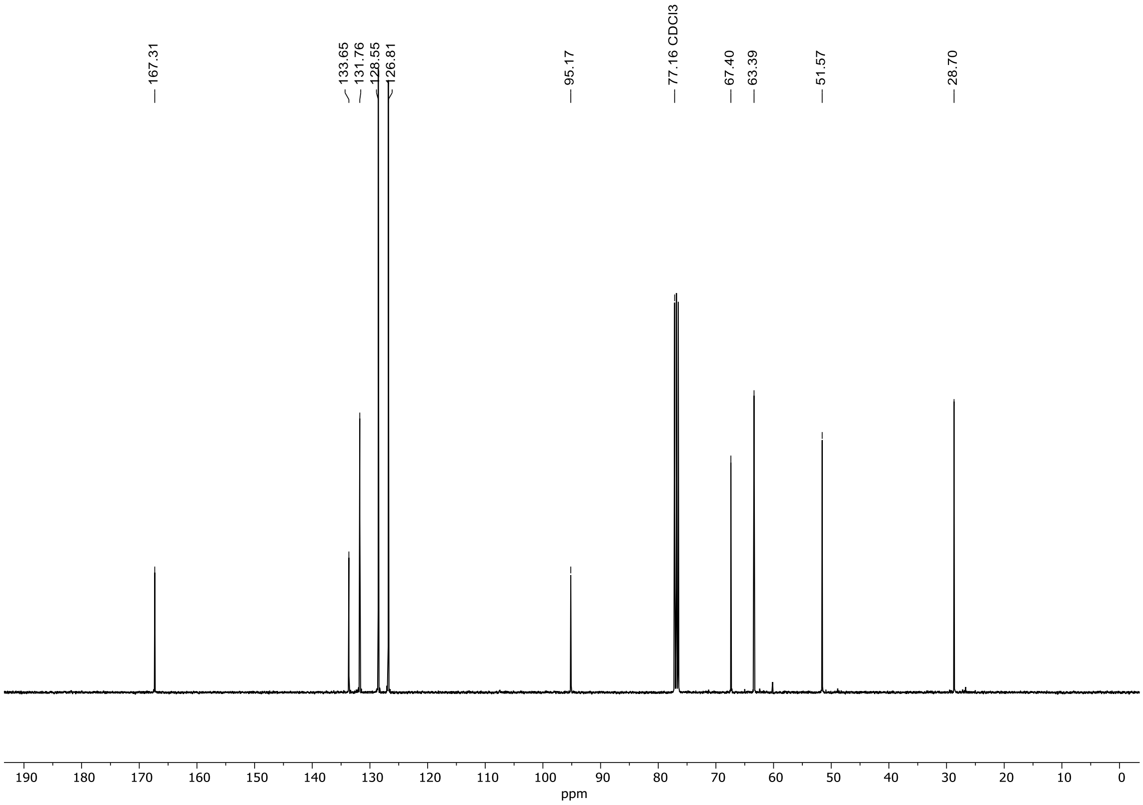

N-(2-Bromo-4-cyano-2-methylbutyl)benzamide (40)

^{1}H-NMR (400MHz)

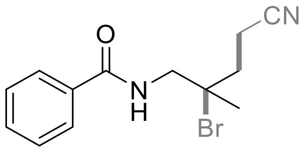

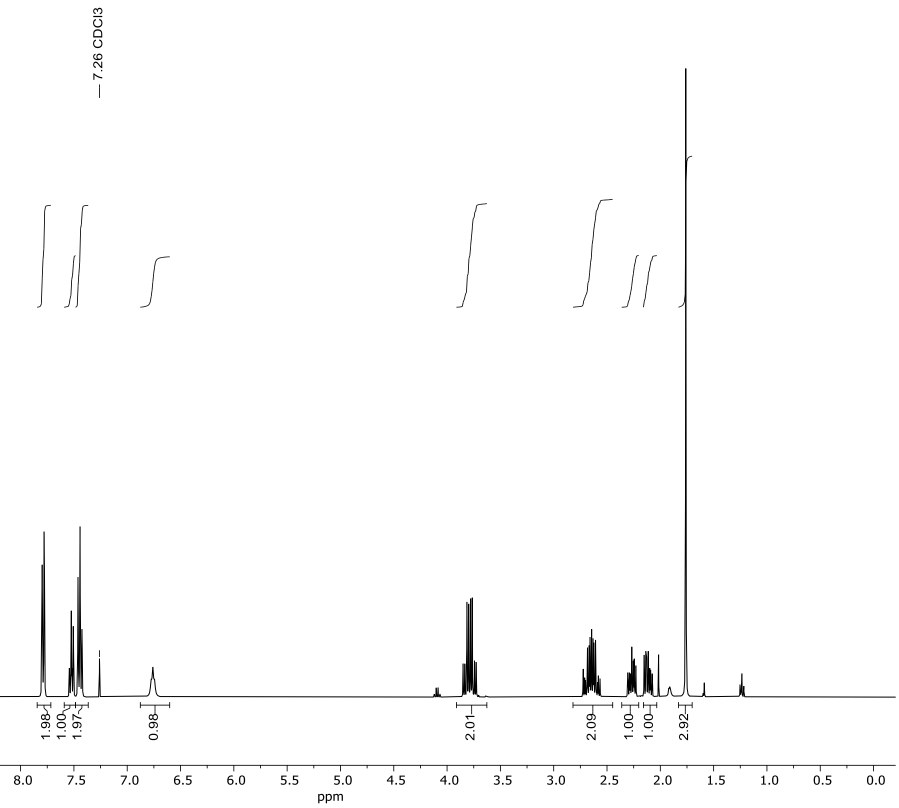

^{13}C-NMR (101MHz)

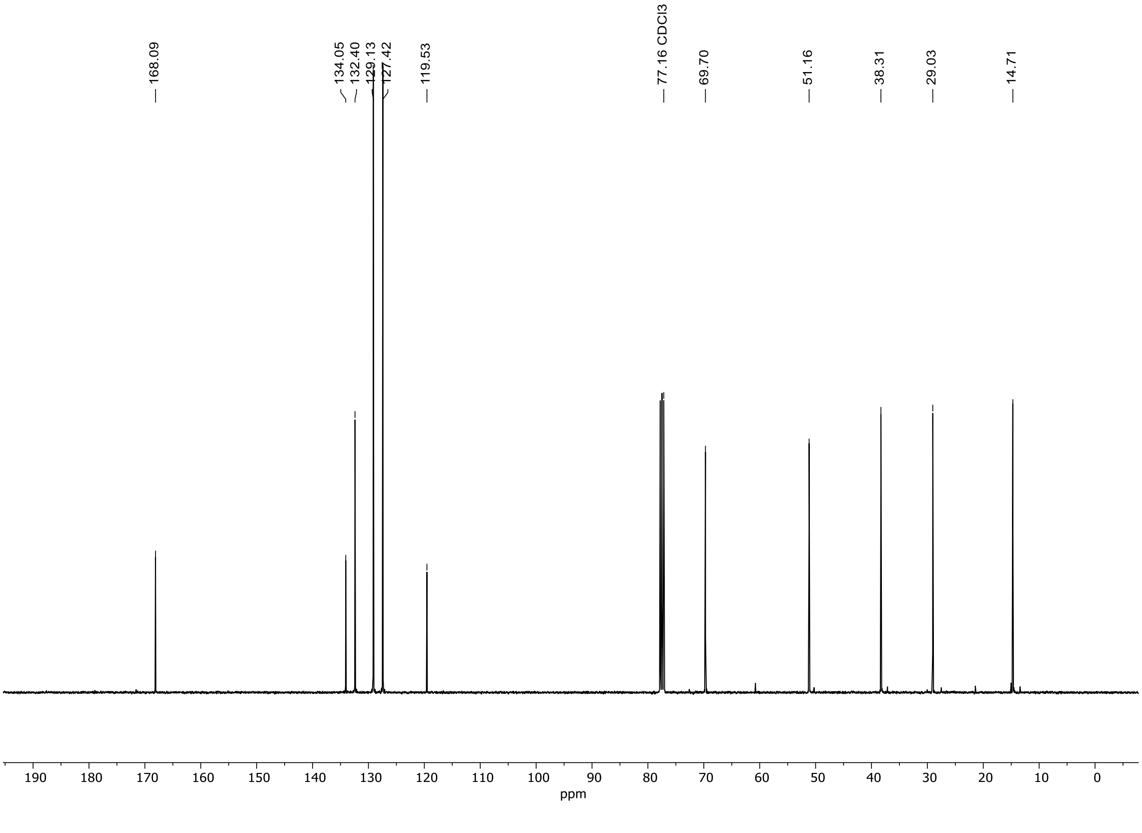

Diethyl 2-(3-benzamido-2-bromo-2-methylpropyl)malonate (41)

^{1}H-NMR (400MHz)

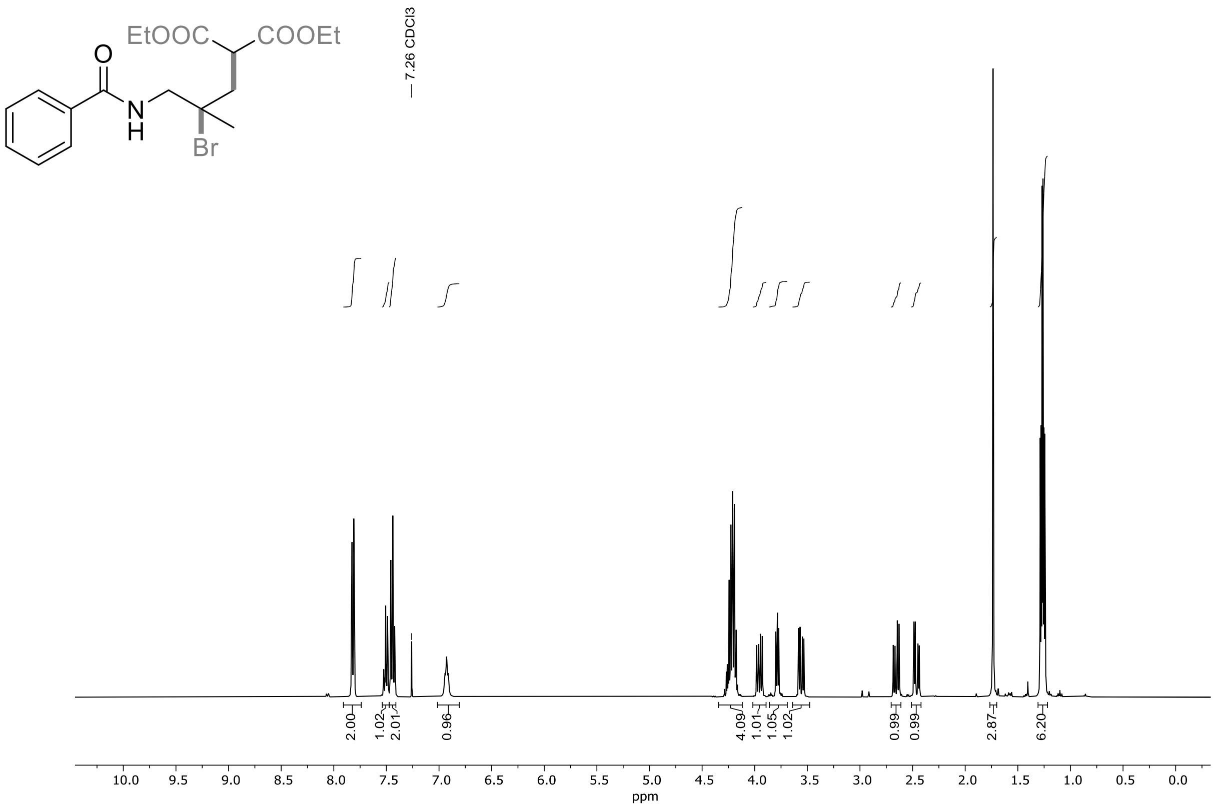

^{13}C-NMR (101MHz)

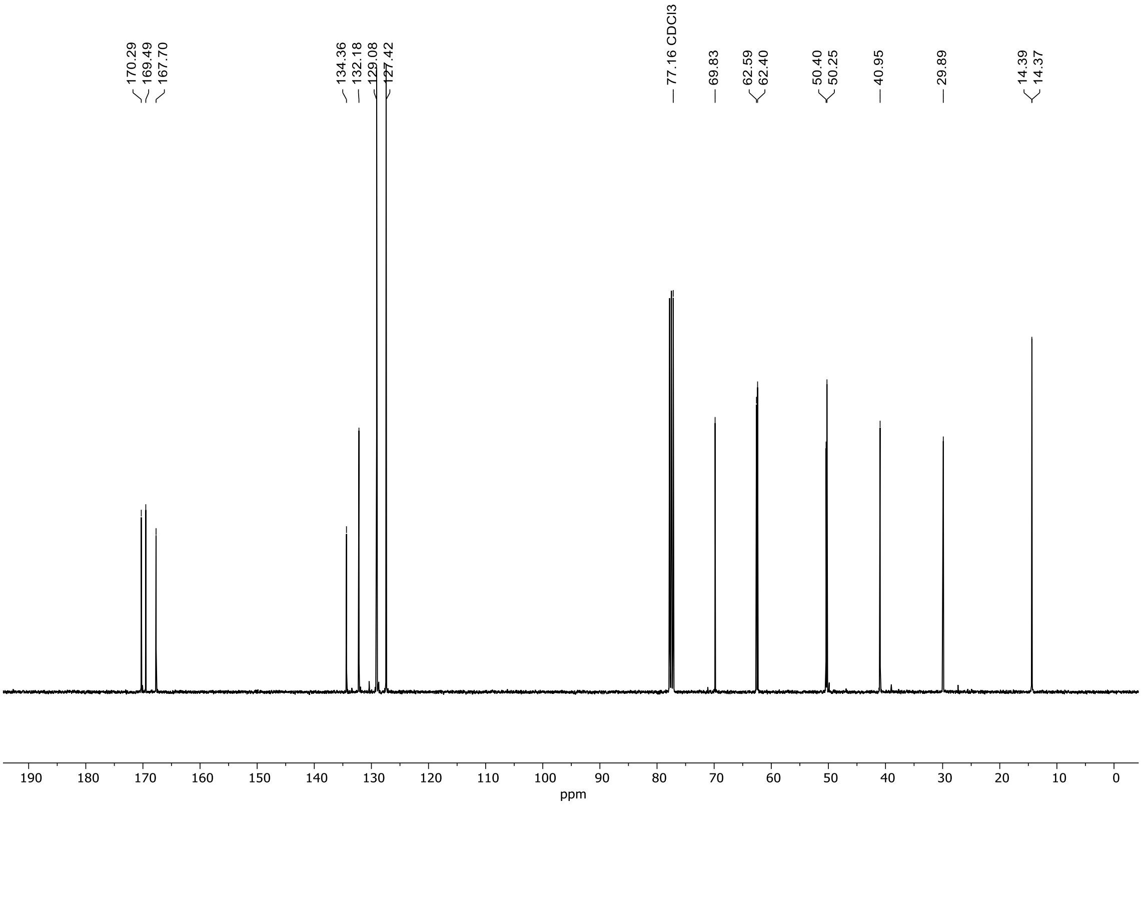

4-Bromo-4-methyl-3-((2-oxopyrrolidin-1-yl)methyl)pentanenitrile (42)

^{1}H-NMR (400MHz)

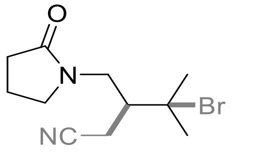

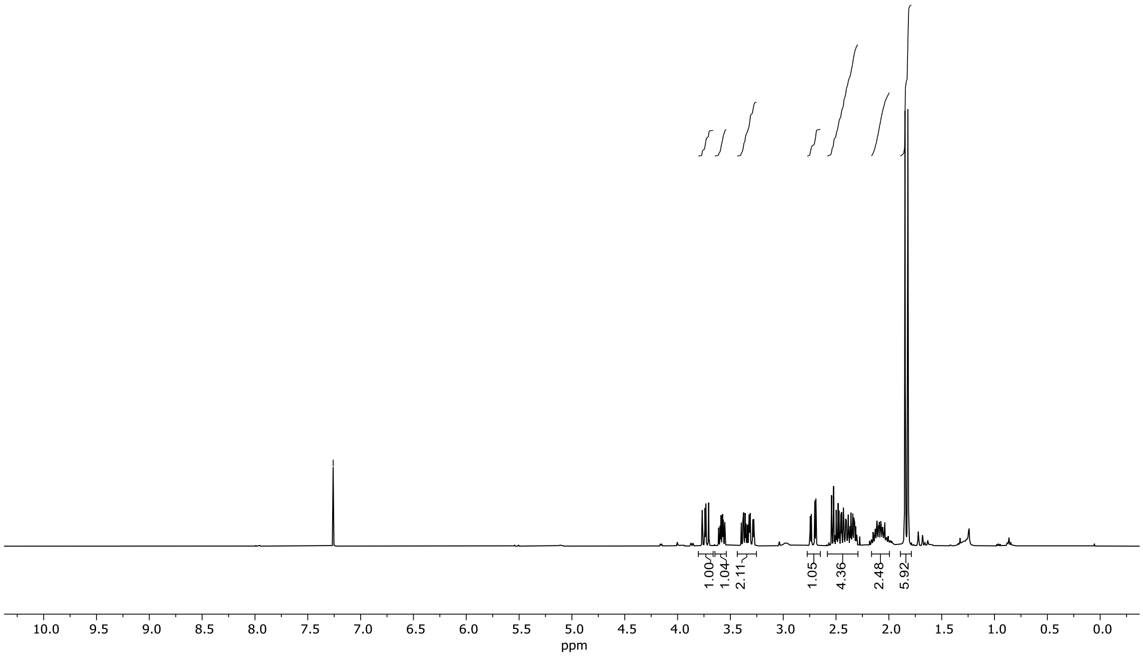

^{13}C-NMR (101MHz)

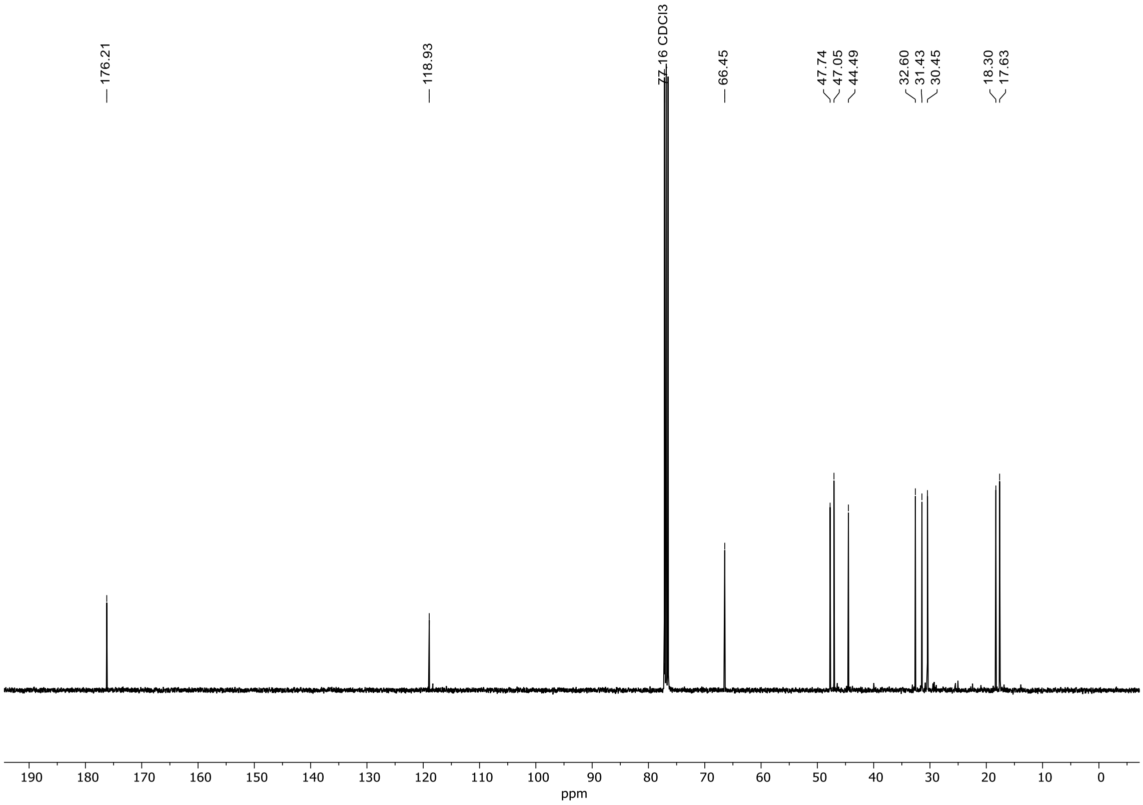

(2-Bromo-4,4,4-trichlorobutyl)benzene (43)

^{1}H-NMR (400MHz)

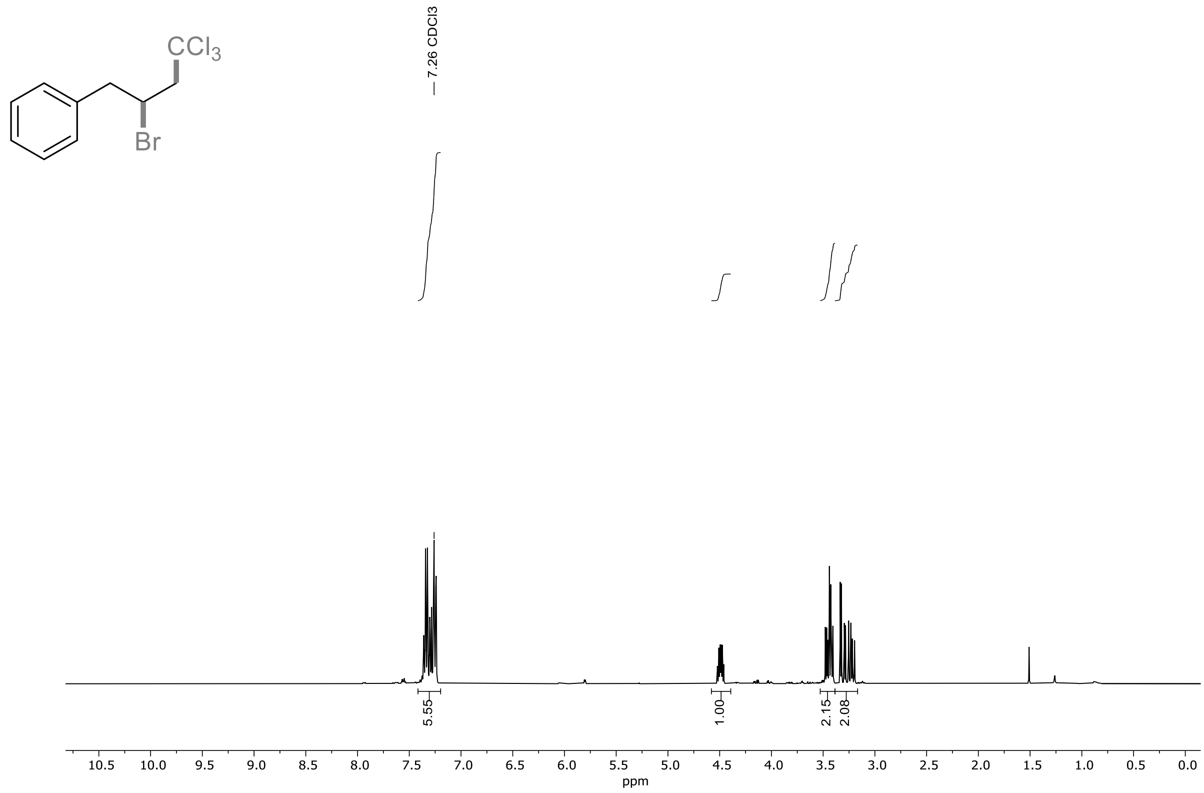

^{13}C-NMR (101MHz)

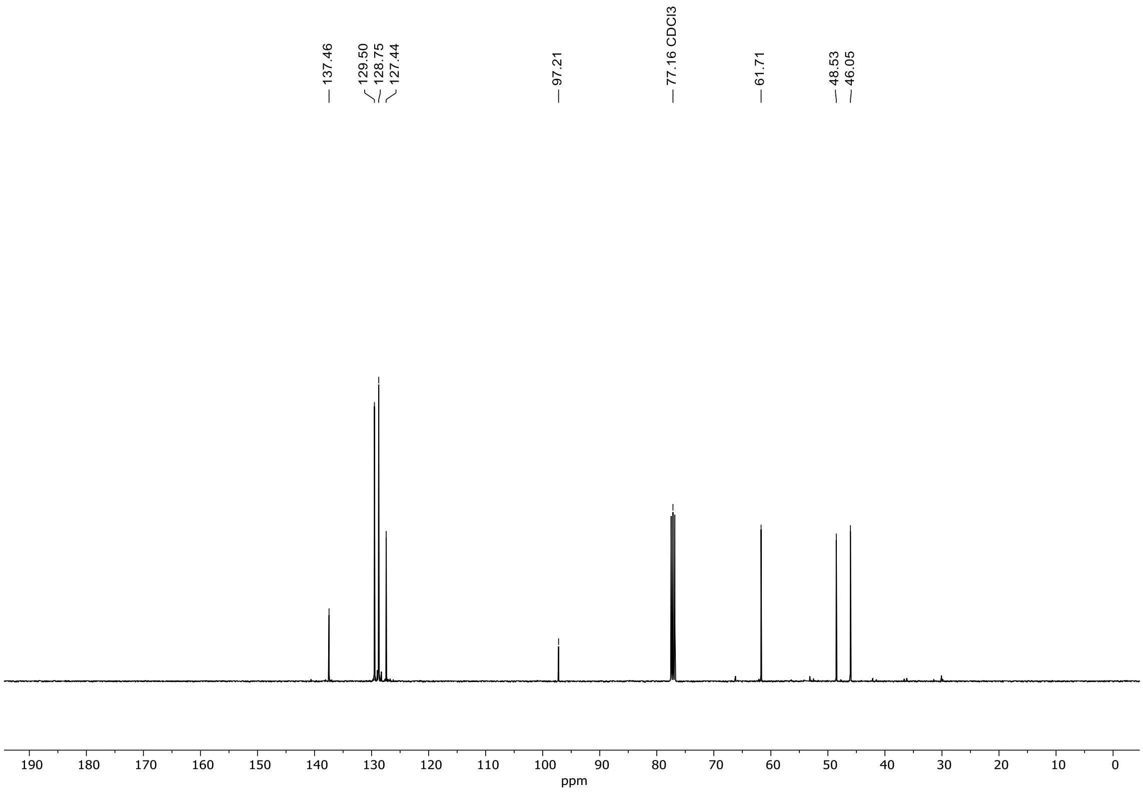

(3-Bromo-5,5,5-trichloropentyl)benzene (44)

^{1}H-NMR (400MHz)

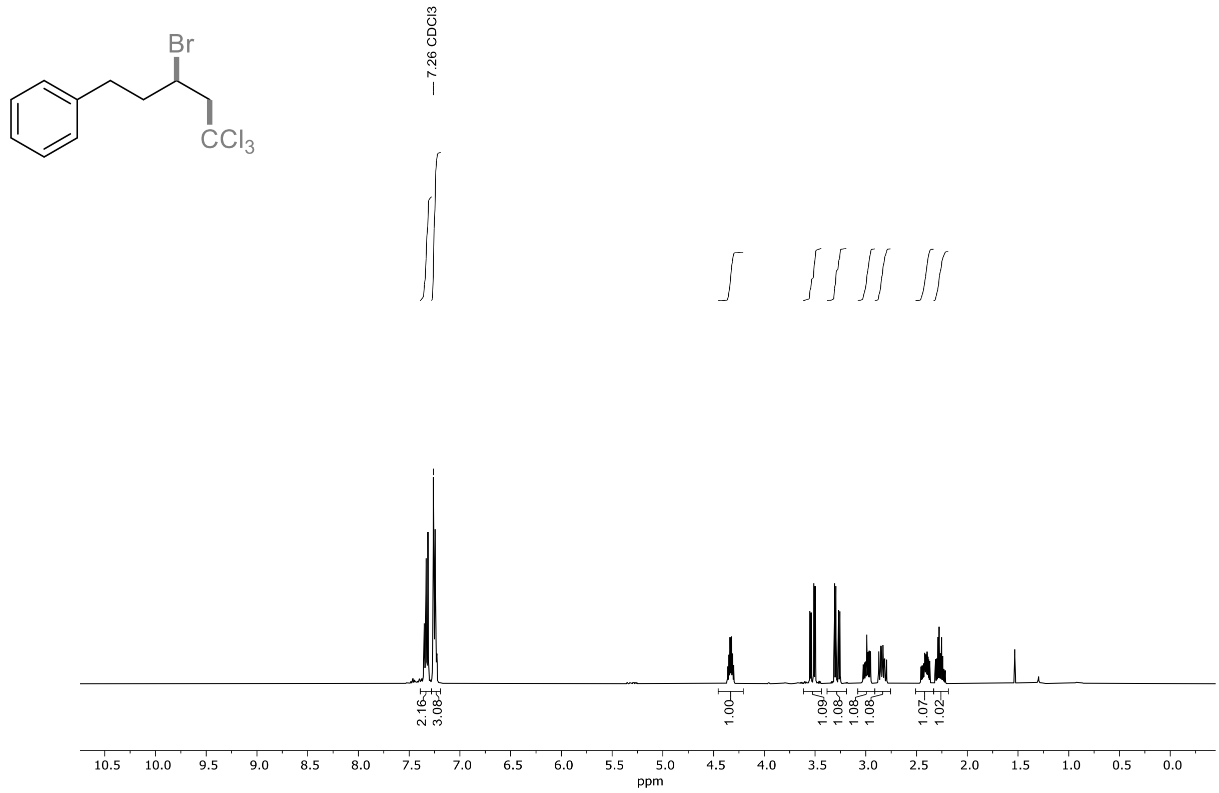

^{13}C-NMR (101MHz)

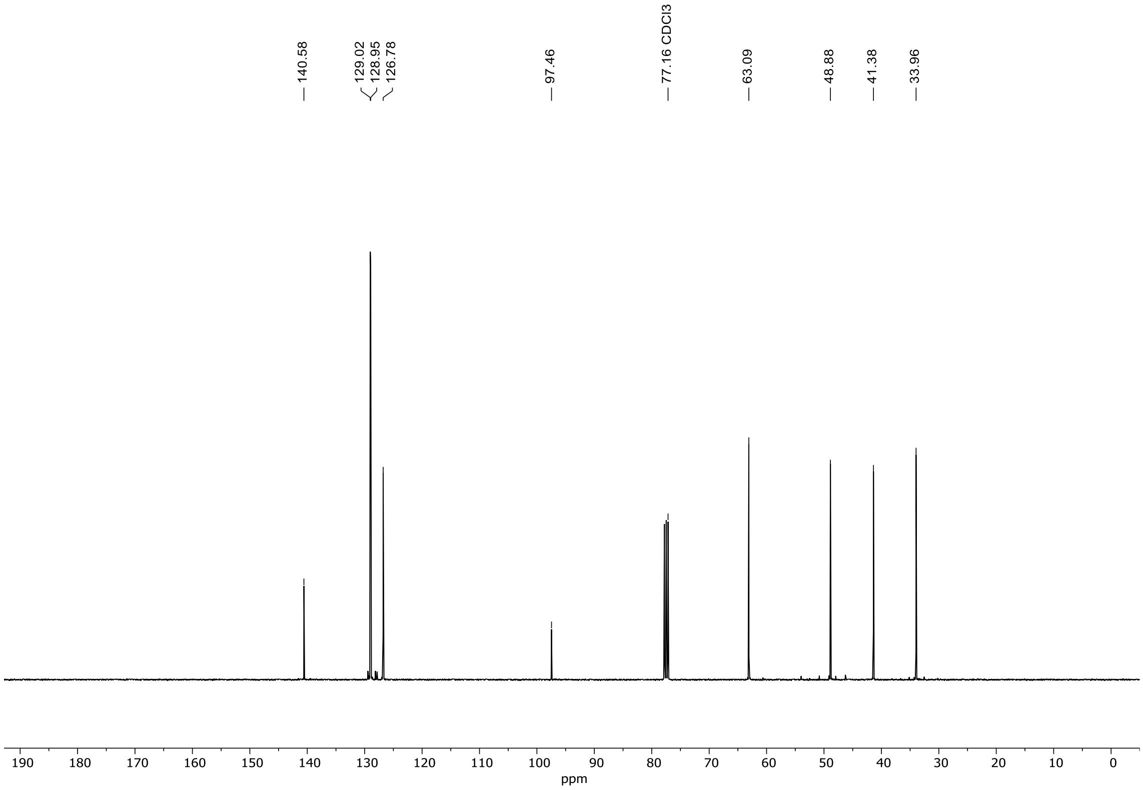

***trans*-1-Bromo-2-(trichloromethyl)-2,3-dihydro-1*H*-indene (45)**

^{1}H-NMR (400MHz)

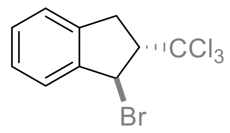

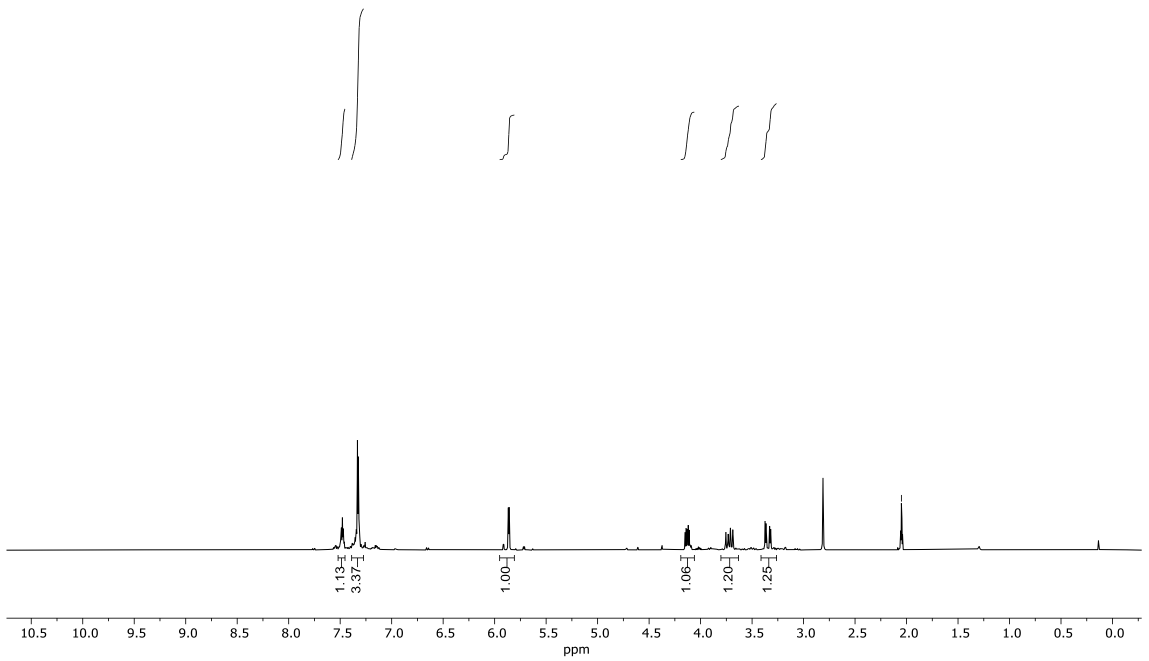

^{13}C-NMR (101MHz)

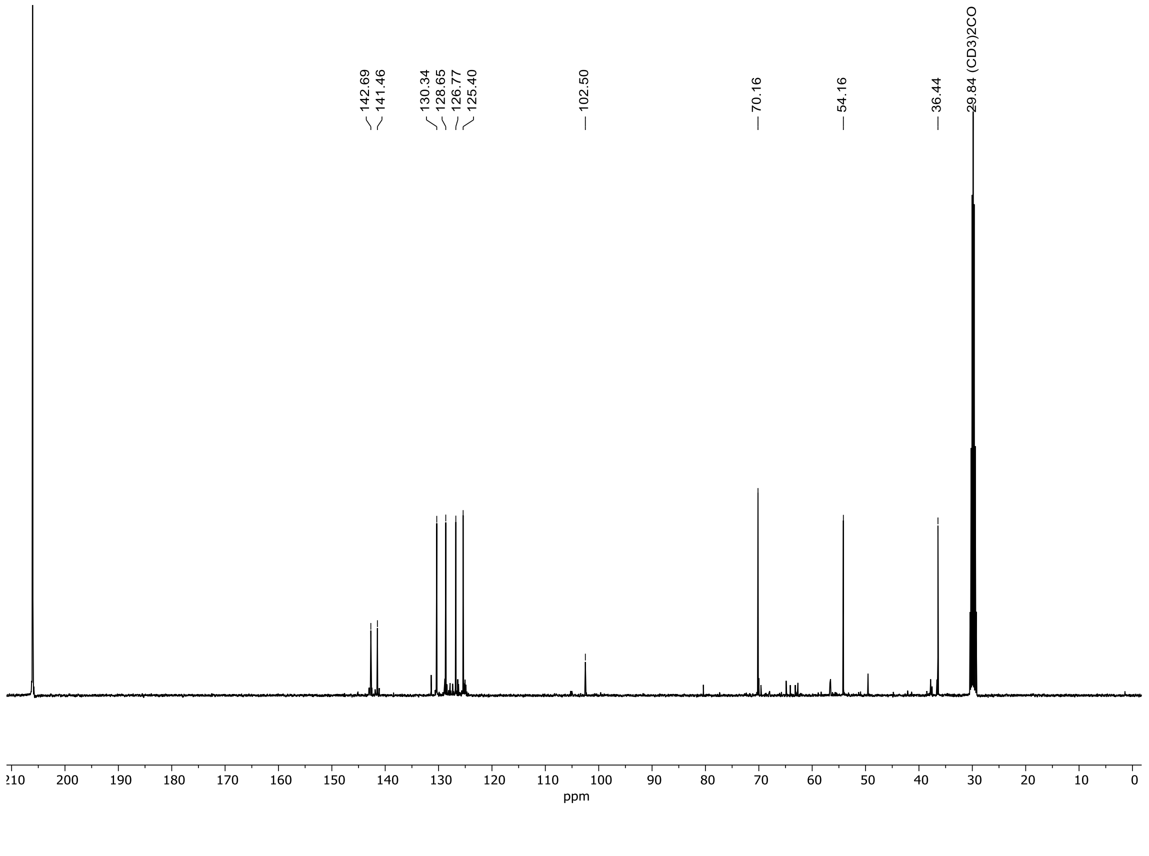

(*Z*)-2-Bromo-4,4,4-trichlorobut-2-en-1-yl benzoate (46)

^{1}H-NMR (400MHz)

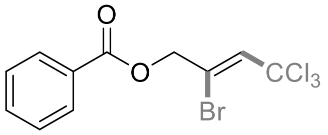

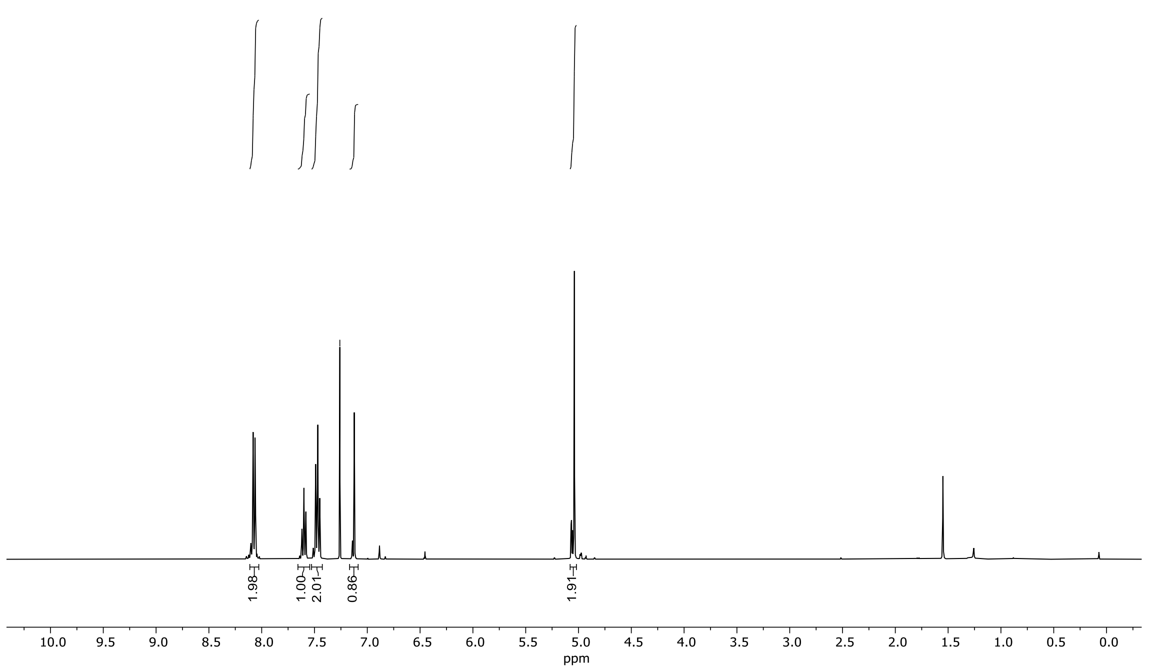

^{13}C-NMR (101MHz)

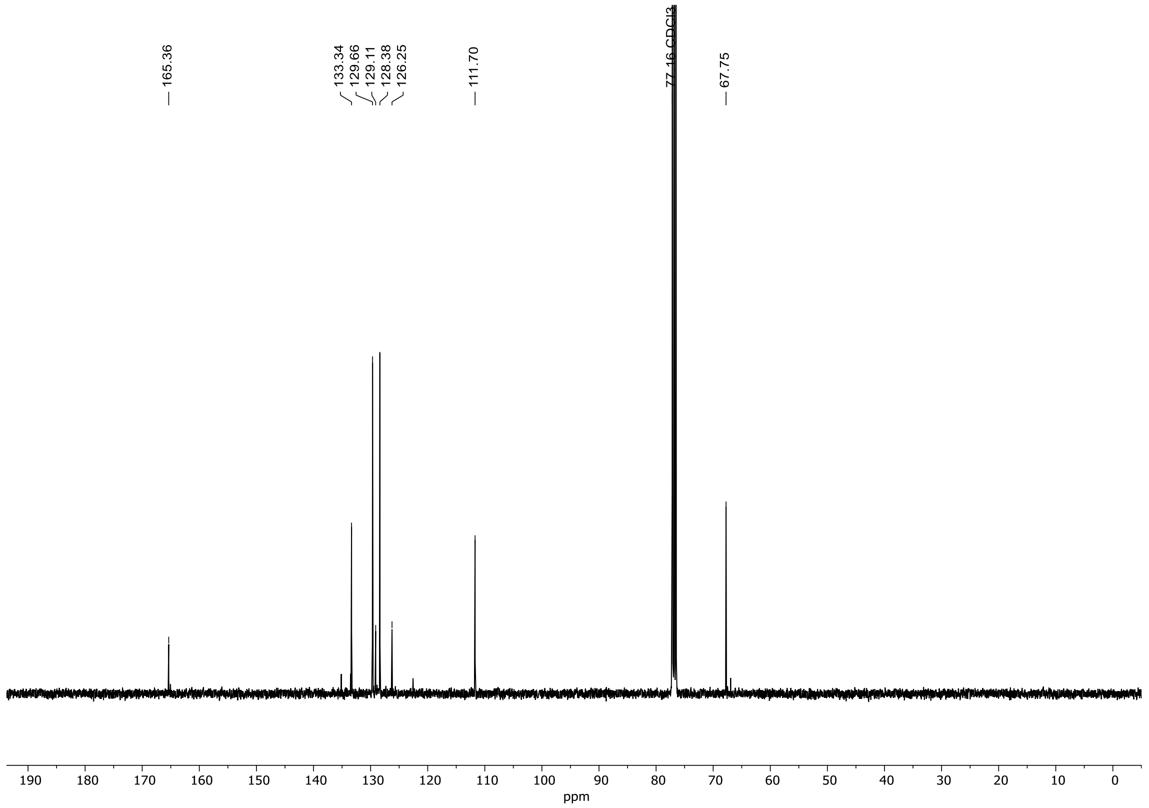

(Z)-2-Bromo-4cyanobut-2-en-1-yl benzoate (47)

^{1}H-NMR (400MHz)

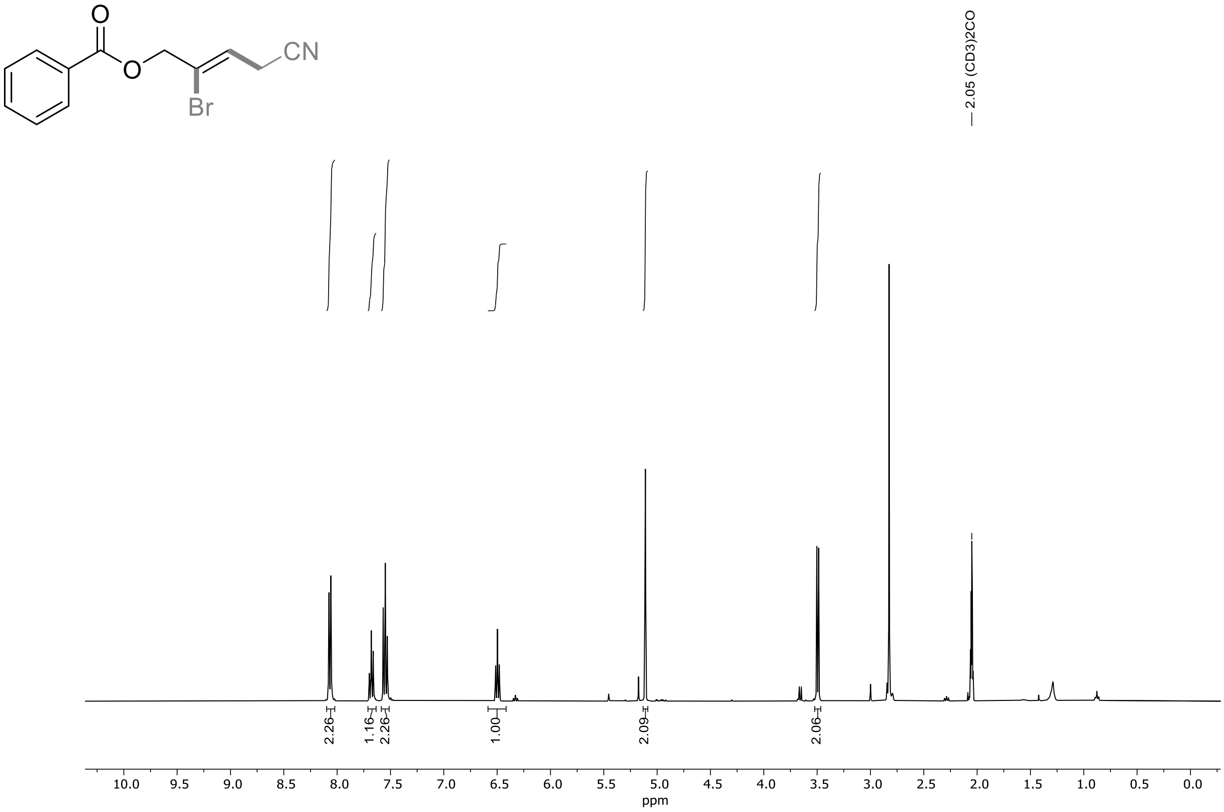

^{13}C-NMR (101MHz)

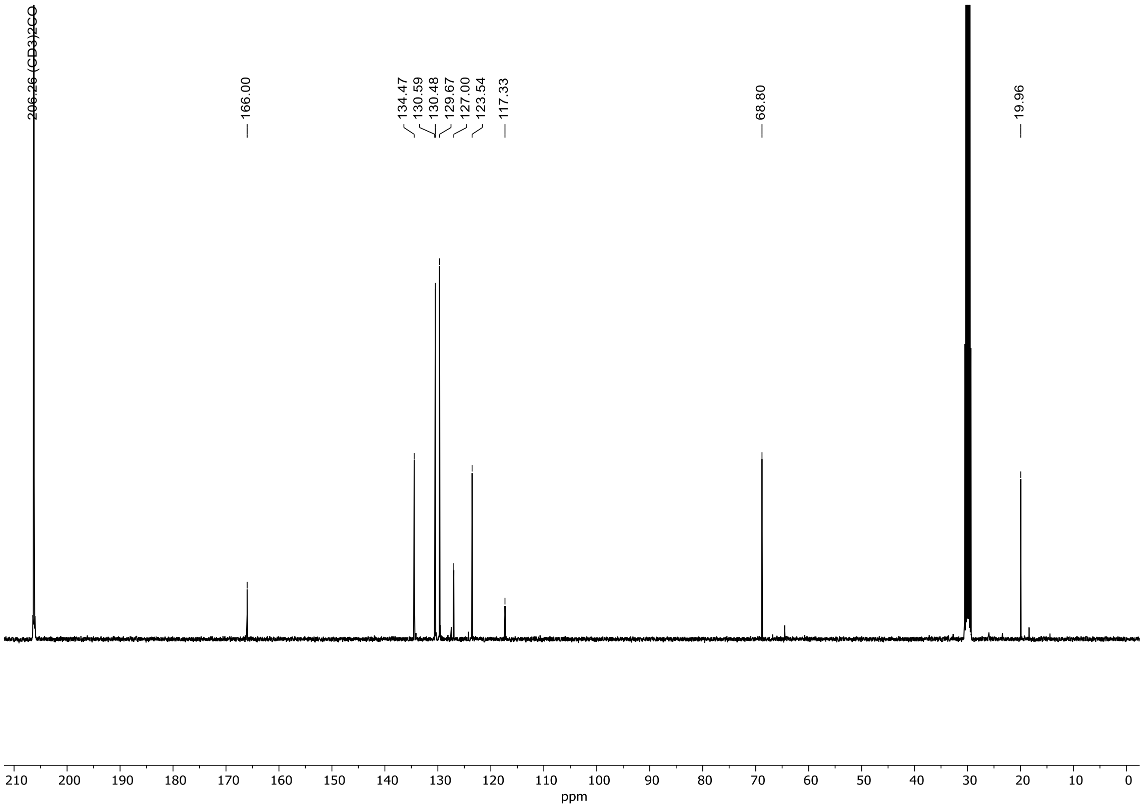

NOESY analysis for compound (*Z*)-2-bromo-4cyanobut-2-en-1-yl benzoate (47)

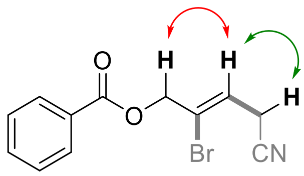

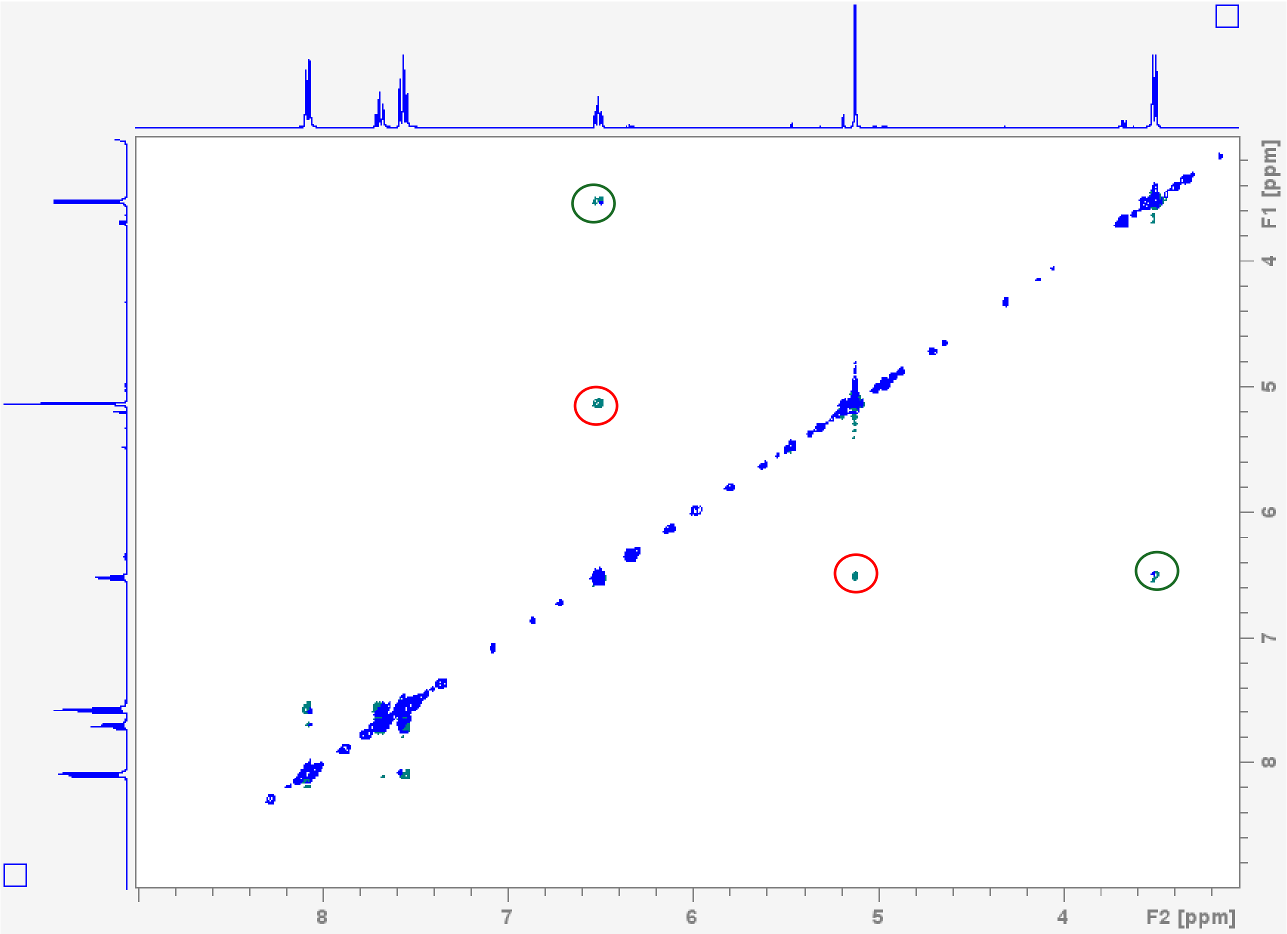

3-Bromo-5,5,5-trichloropent-3-en-1-yl benzoate (48)

^{1}H-NMR (400MHz)

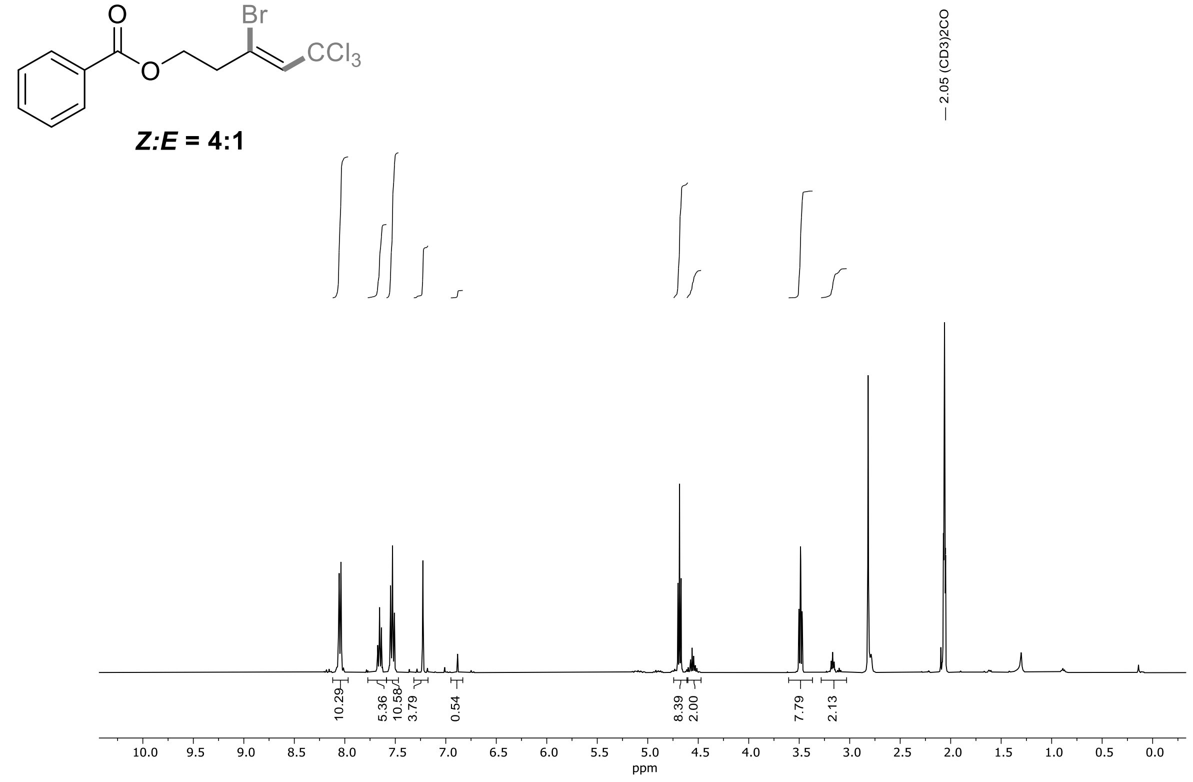

^{13}C-NMR (101MHz)

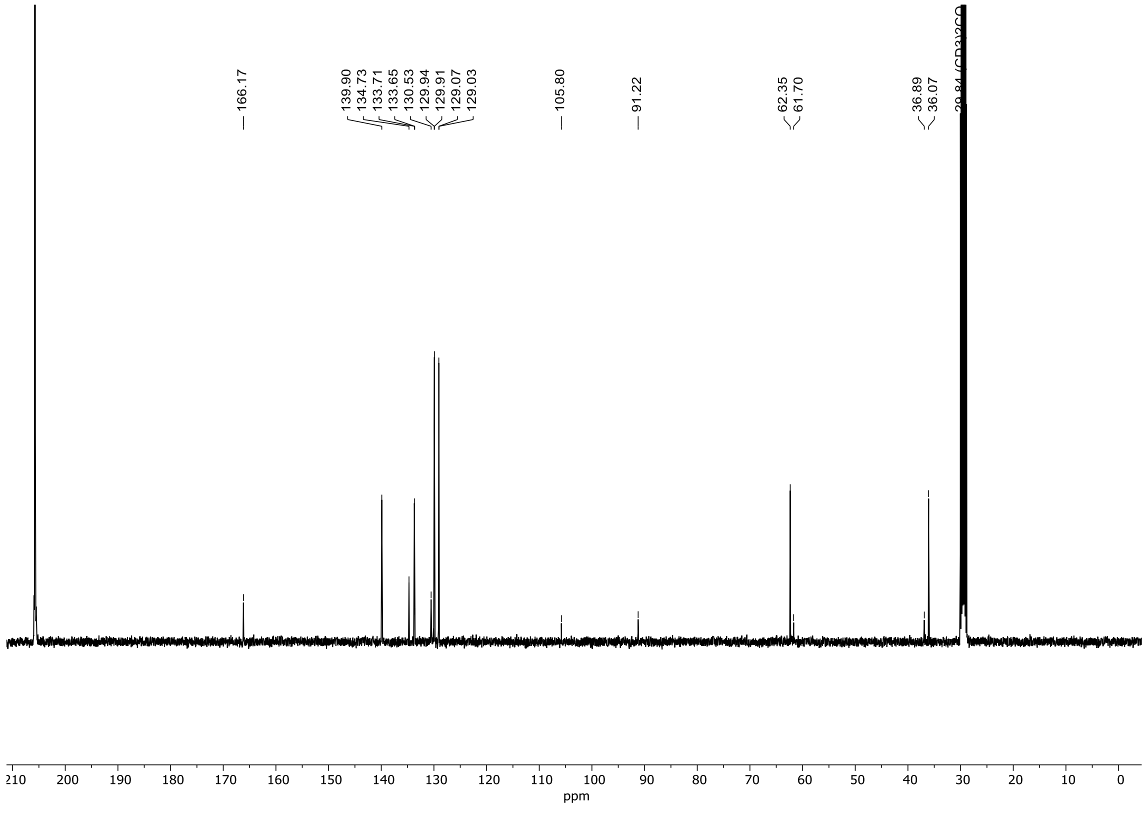

3-Bromo-5-cyanopent-3-en-1yl benzoate (49)

^{1}H-NMR (400MHz)

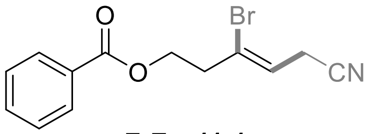

***Z:E* = 11:1**

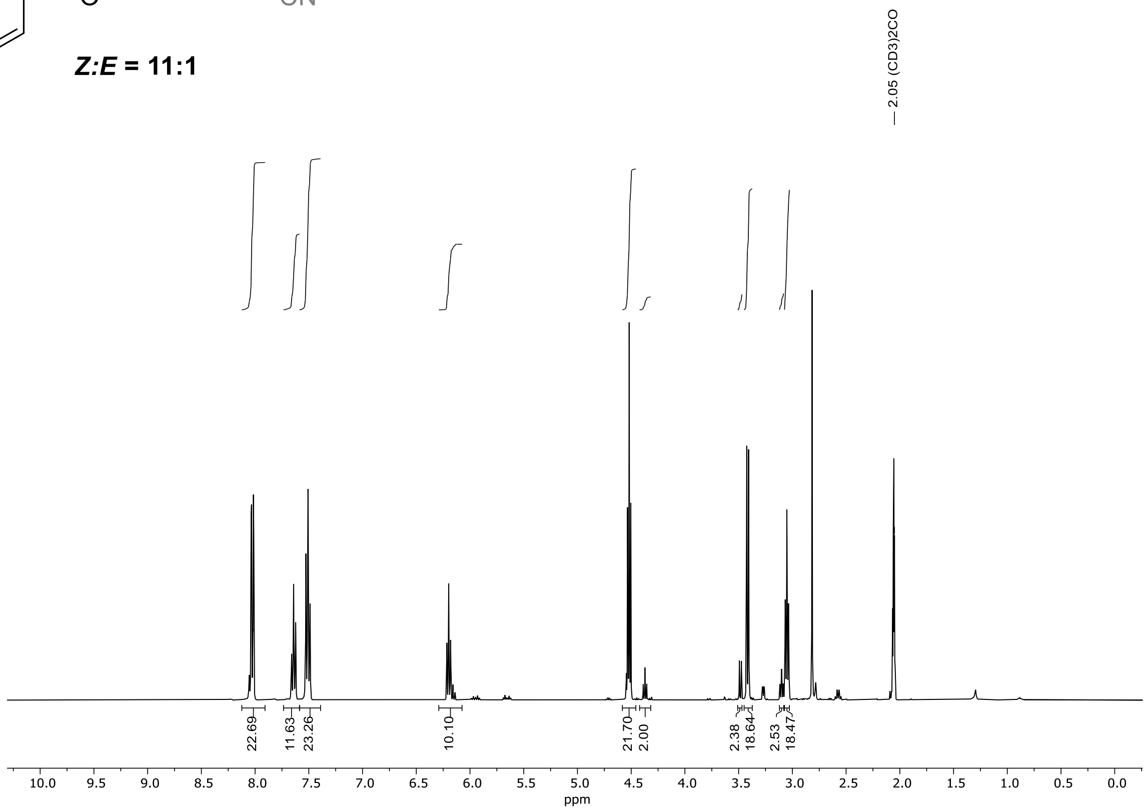

^{13}C-NMR (101MHz)

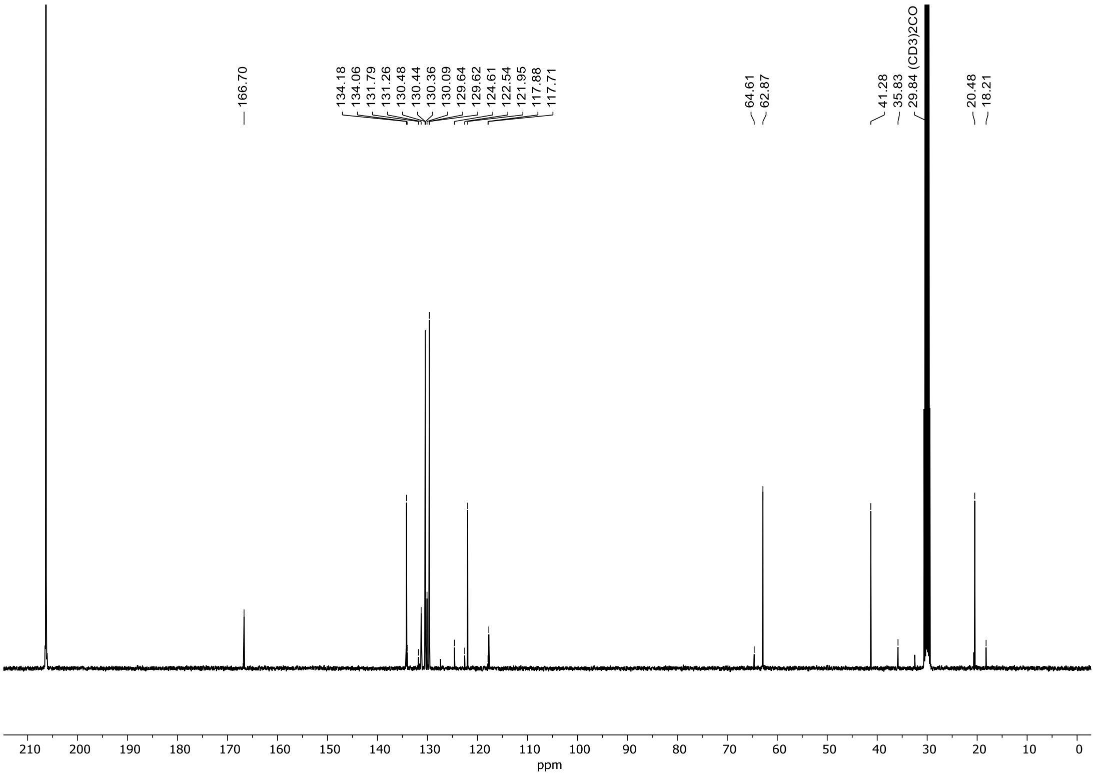

NOESY analysis for compound 3-bromo-5-cyanopent-3-en-1yl benzoate (49)

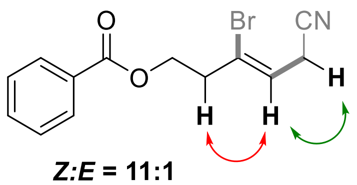

Z:E **= 11:1**

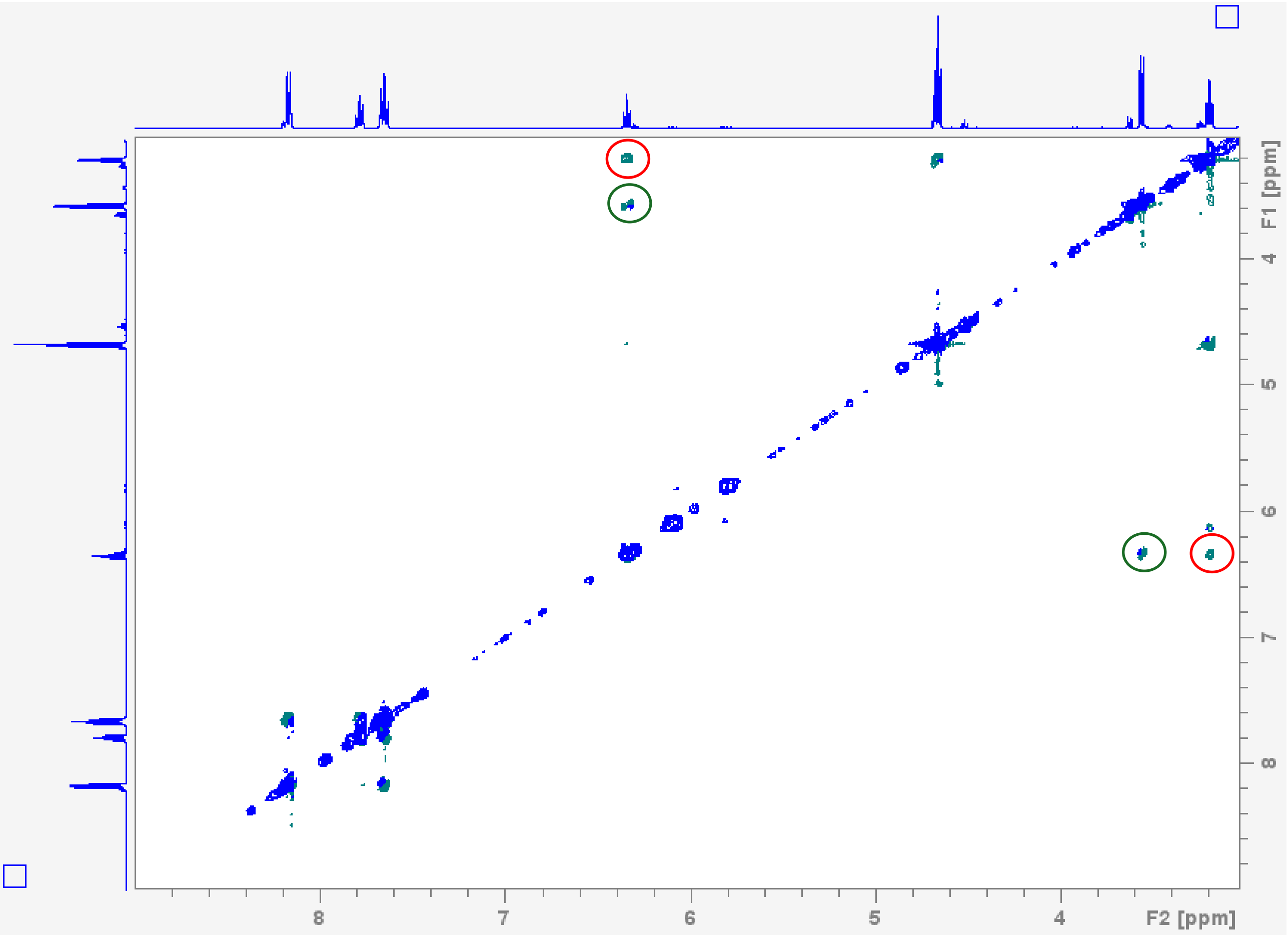

(*Z*)-3-bromo-2-(tricholoromethyl)but-2-en-1-yl benzoate (50)

^{1}H-NMR (400MHz)

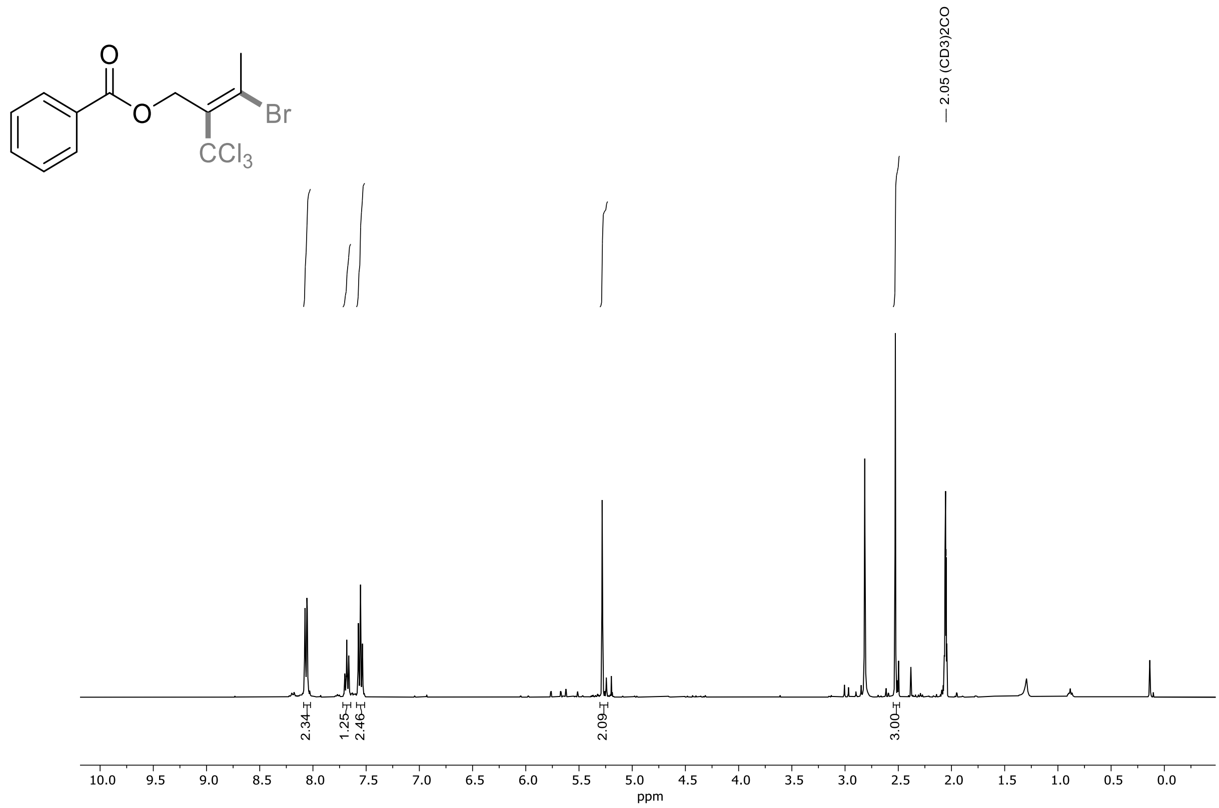

^{13}C-NMR (101MHz)

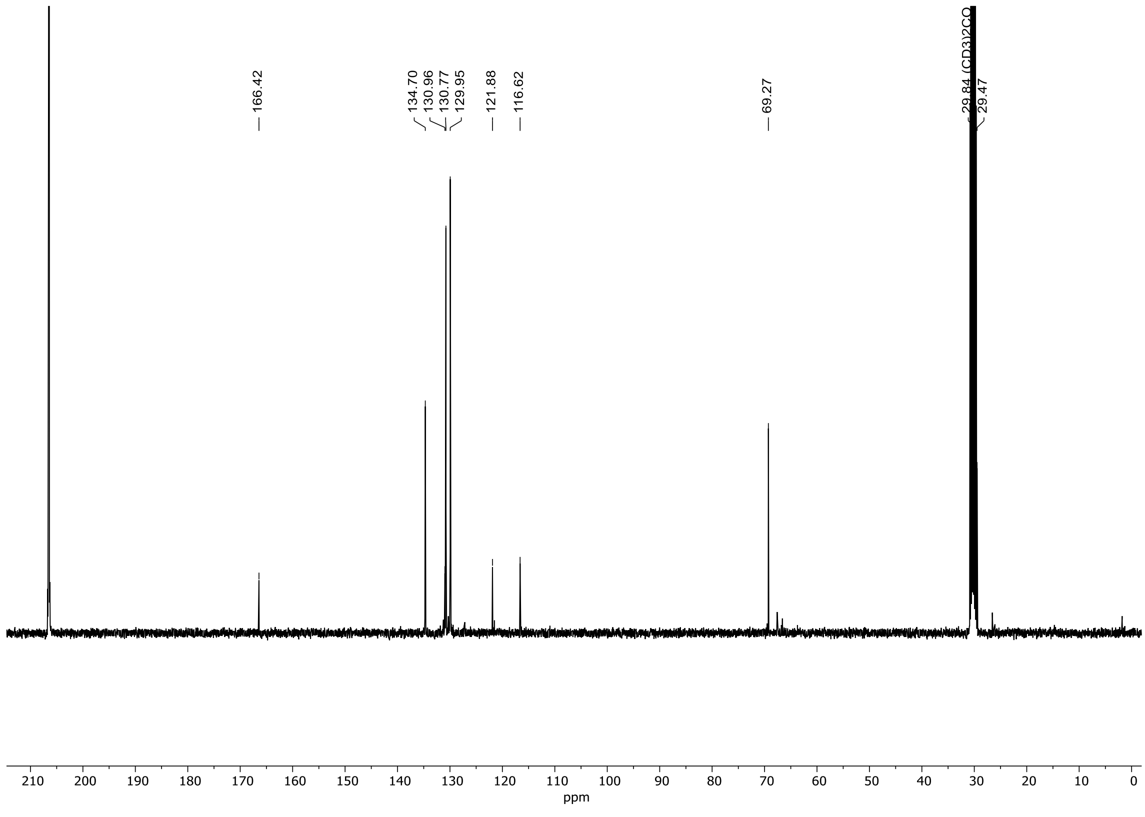

(*E*)-3-Bromo-2-(cyanomethyl)but-2-en-1-yl benzoate (51)

^{1}H-NMR (400MHz)

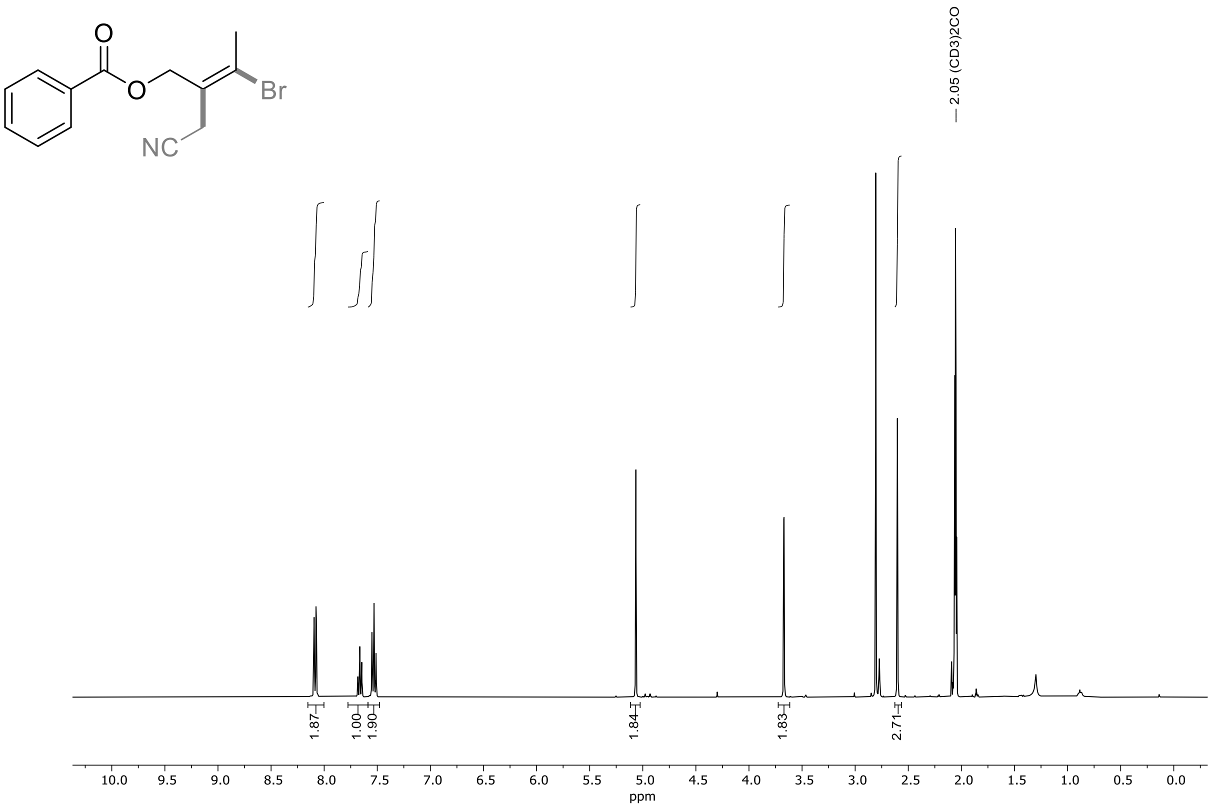

^{13}C-NMR (101MHz)

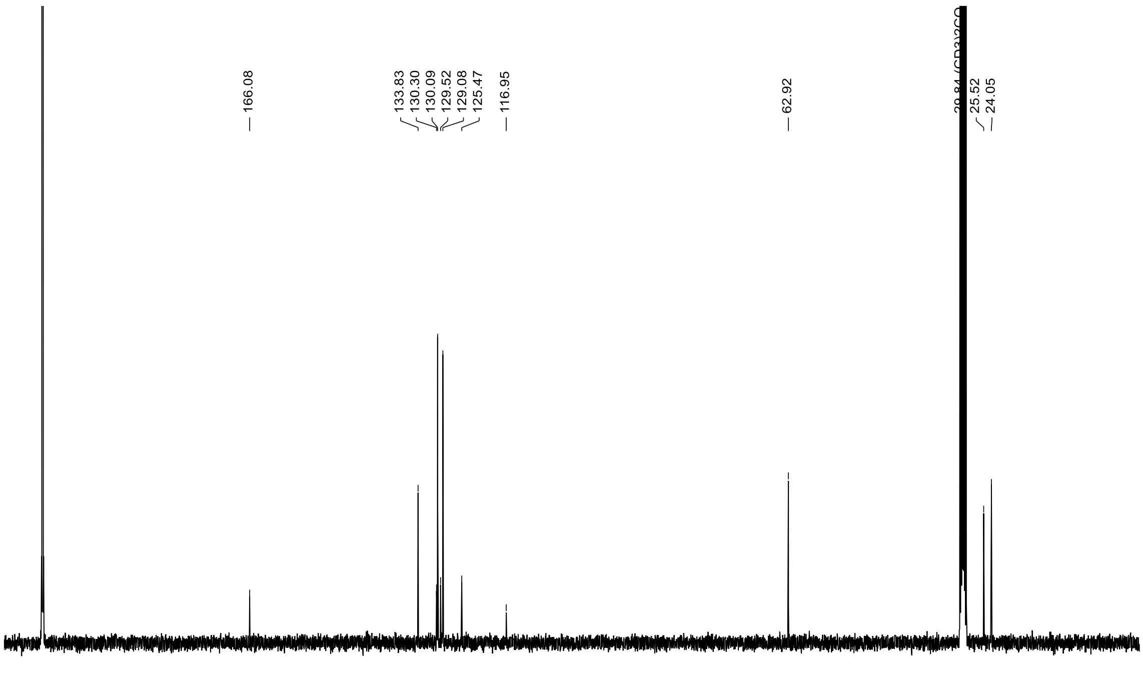

NOESY analysis for compound (*E*)-3-Bromo-2-(cyanomethyl)but-2-en-1-yl benzoate (51)

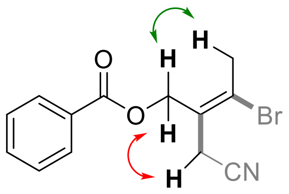

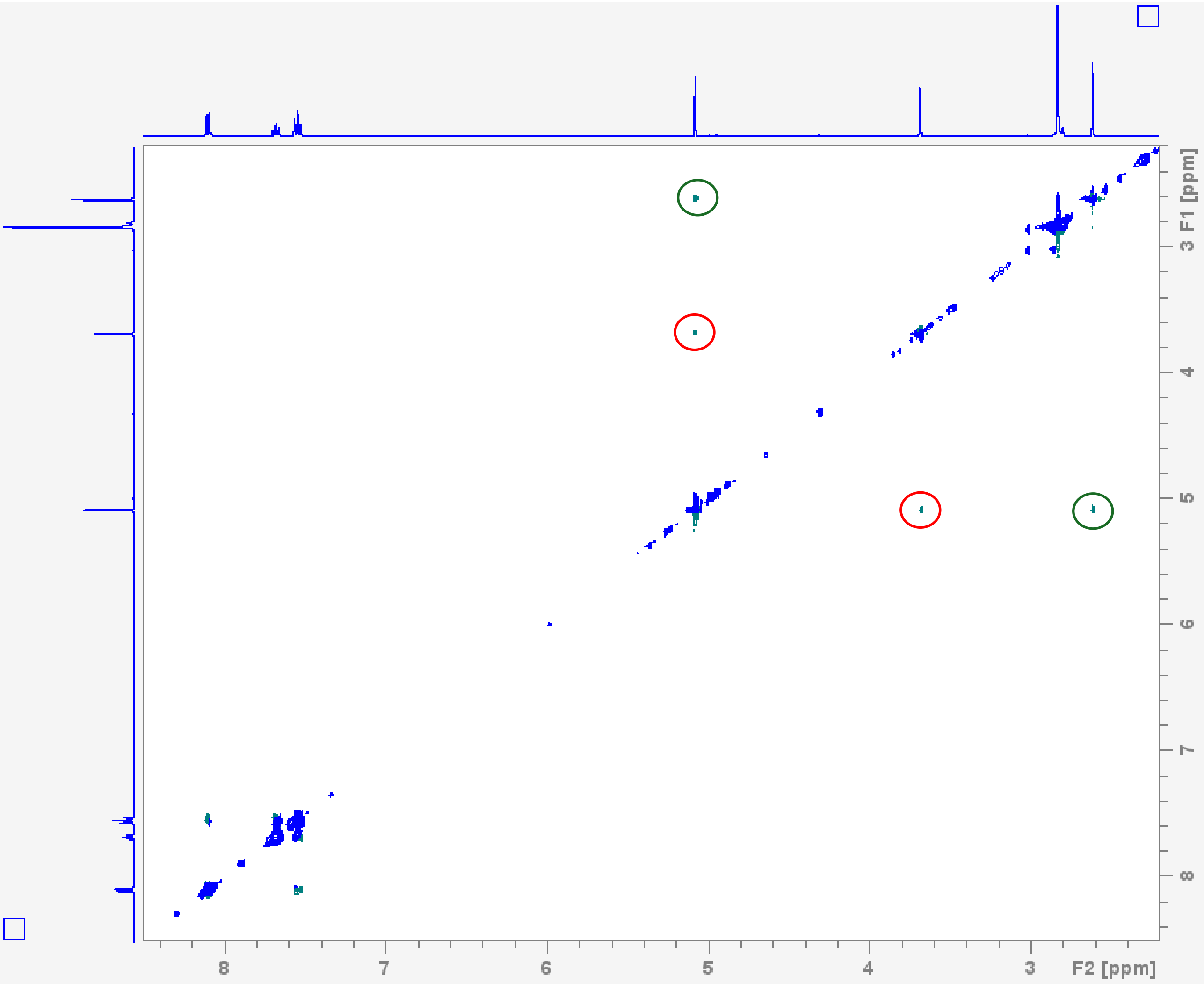

3-Bromo-2-(cyanomethyl)-3-phenylallyl benzoate (52)

^{1}H-NMR (400MHz)

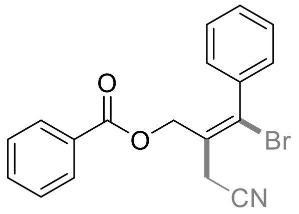

***E*:*Z* = 2:1**

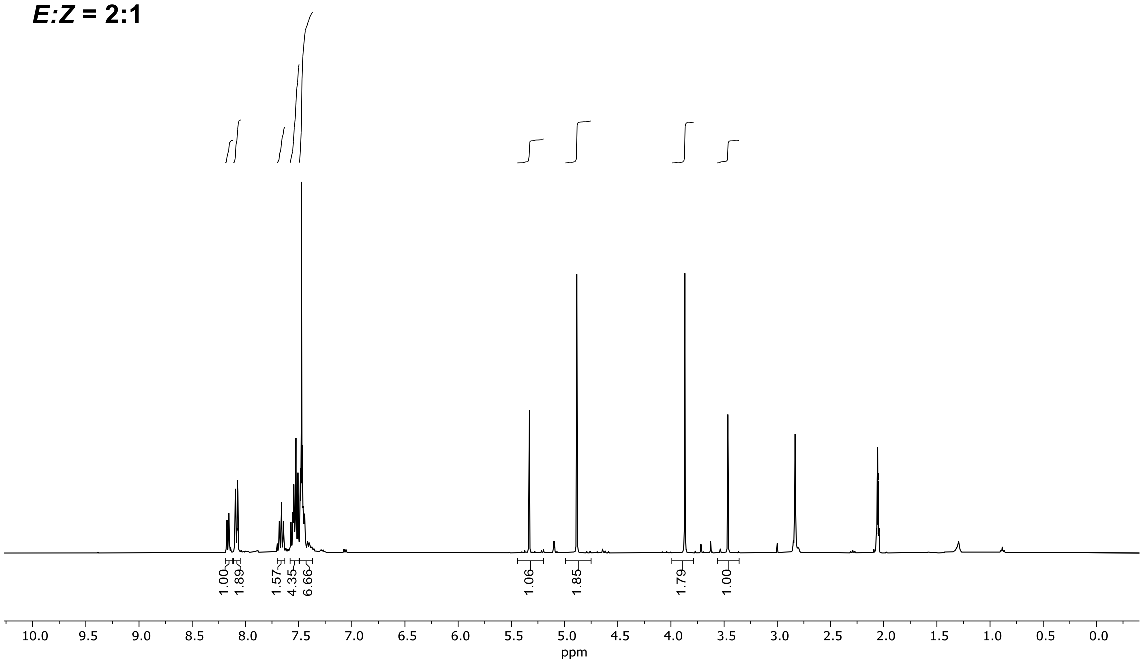

^{13}C-NMR (101MHz)

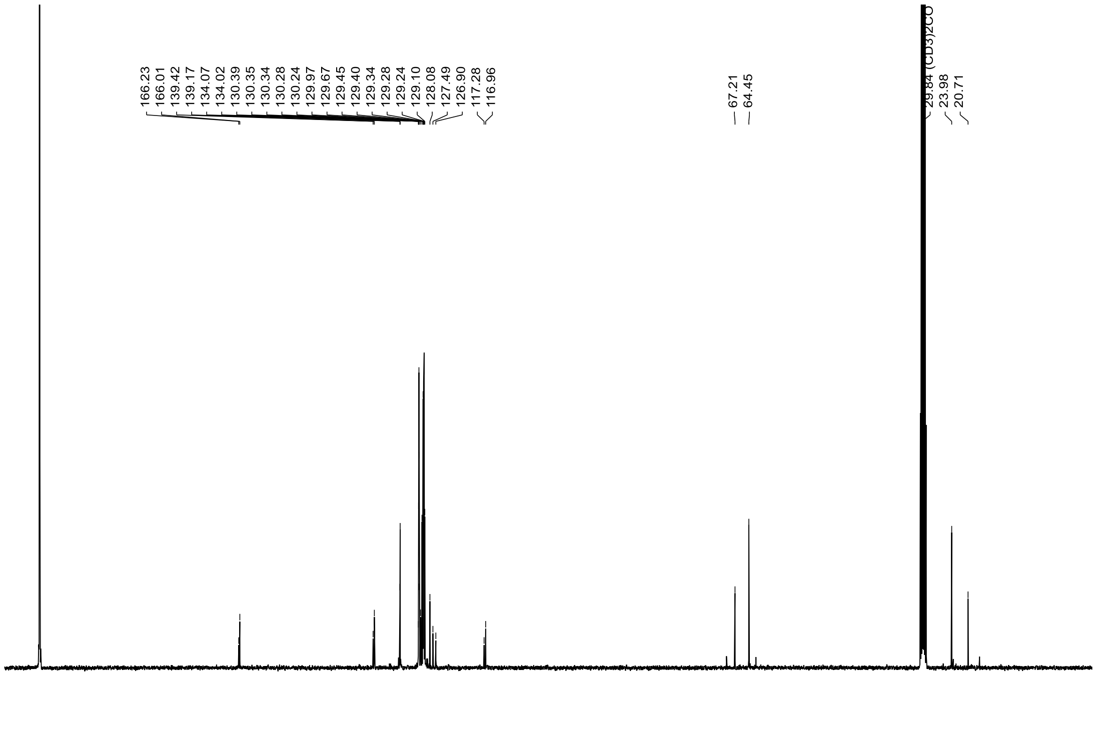

NOESY analysis for compound 3-bromo-5-cyanopent-3-en-1yl benzoate (52)

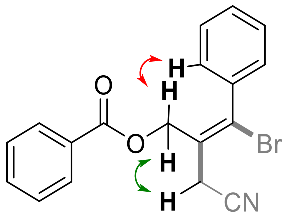

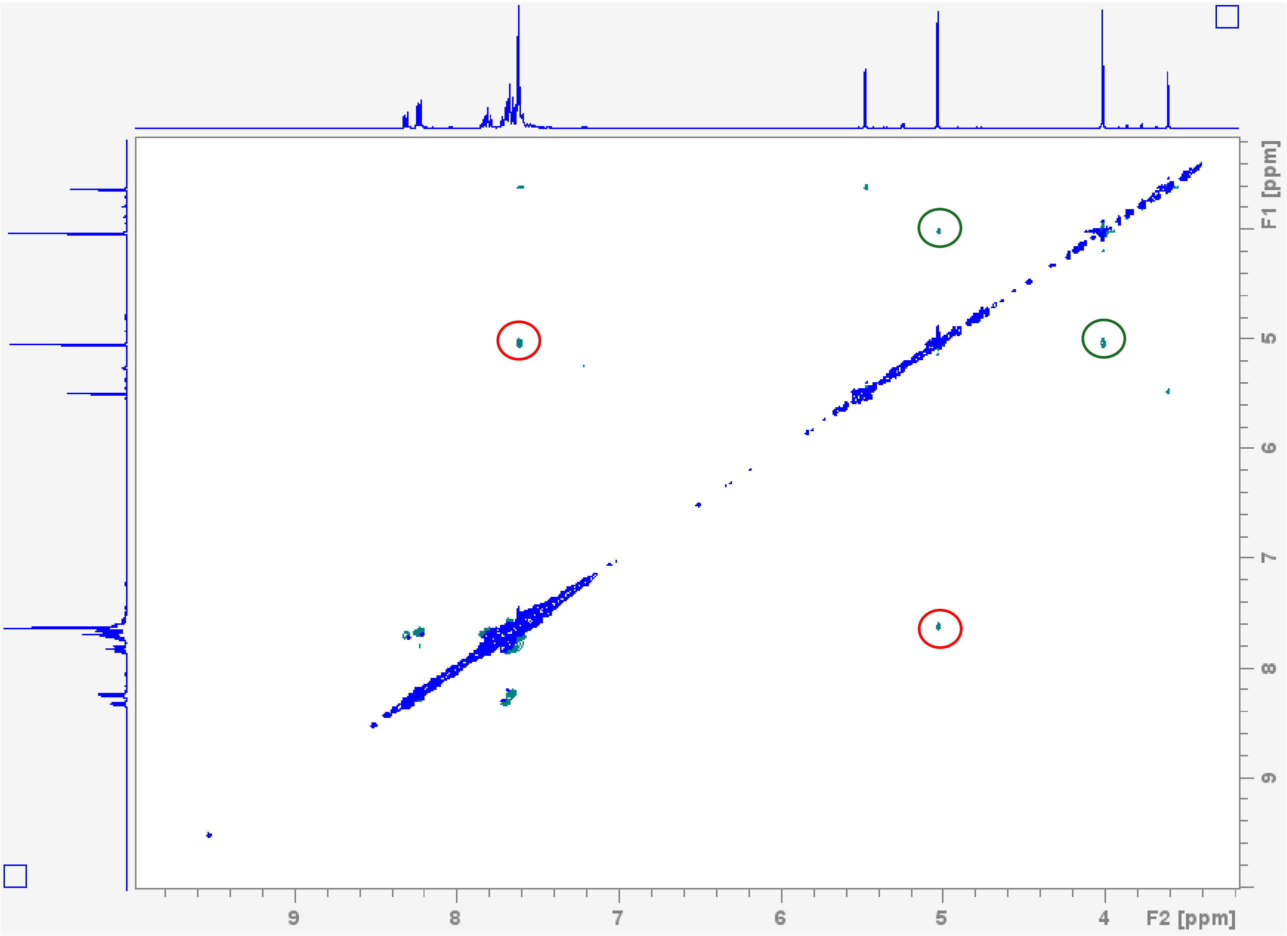

N-(2-Bromo-4cyanobut-2-en-1-yl)benzamide (53)

^{1}H-NMR (400MHz)

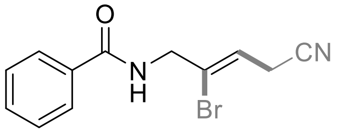

***Z*:*E* = 10:1**

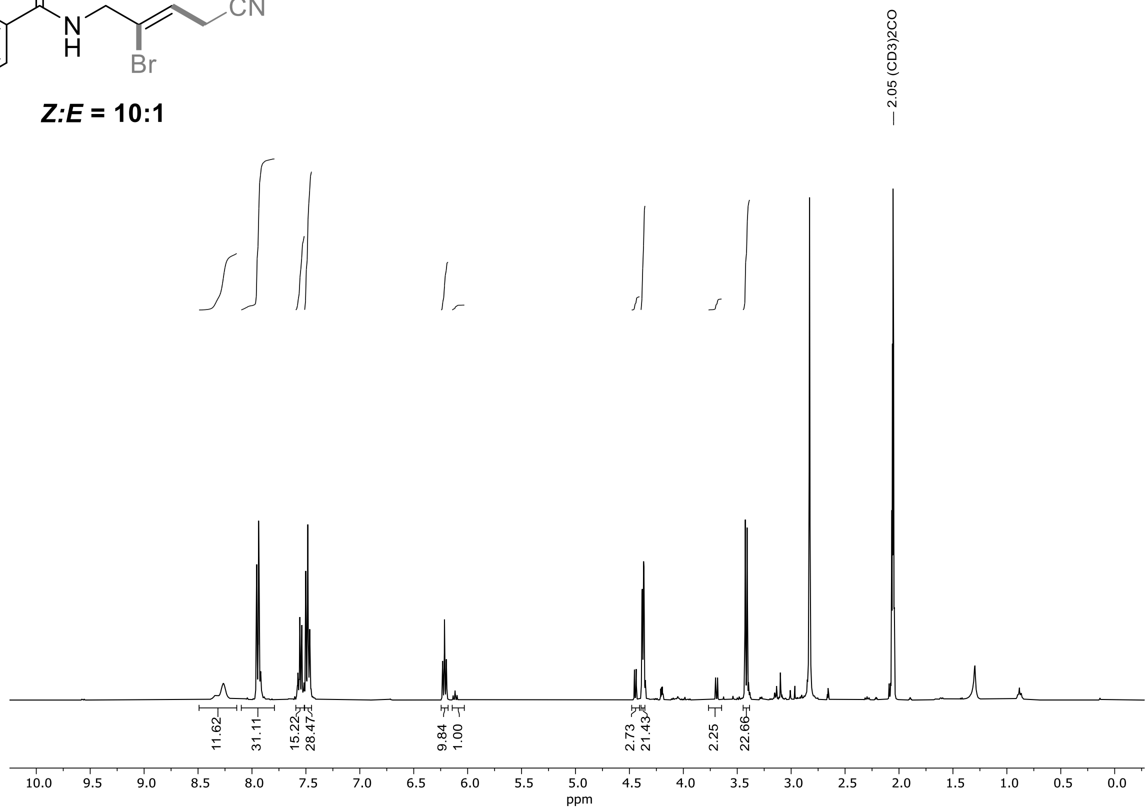

^{13}C-NMR (101MHz)

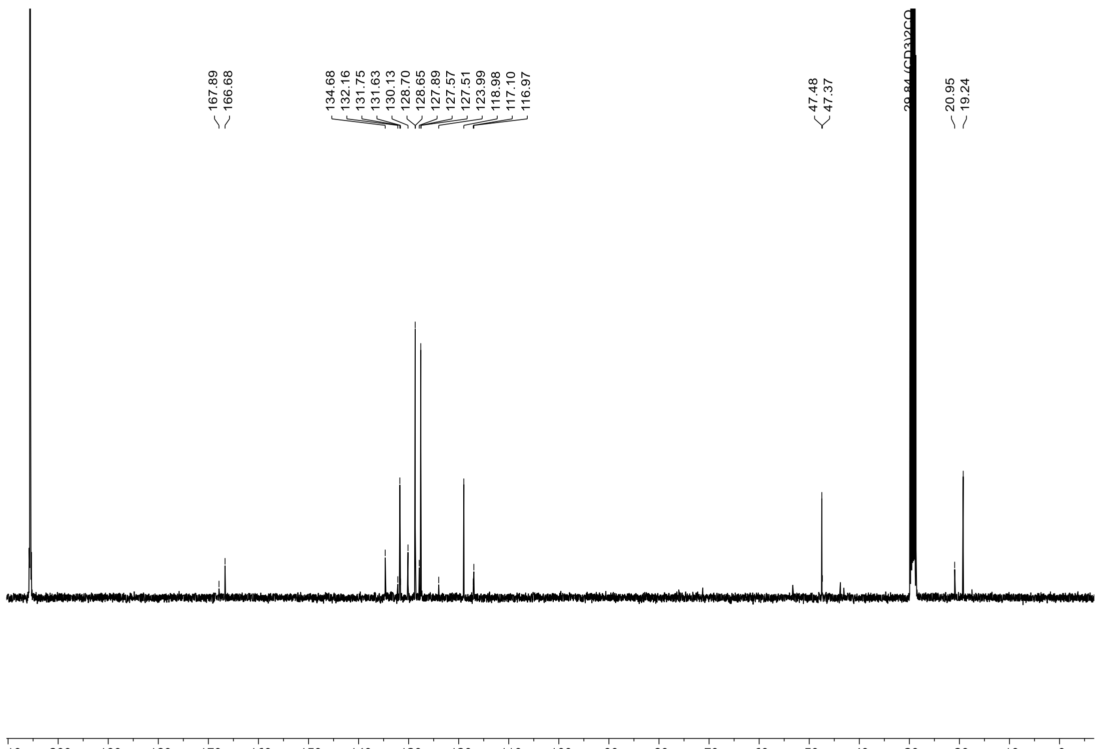

(*Z*)-*N*-(3-Bromo-5-cyanopent-3-en-1-yl)benzamide (54)

^{1}H-NMR (400MHz)

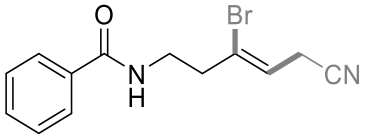

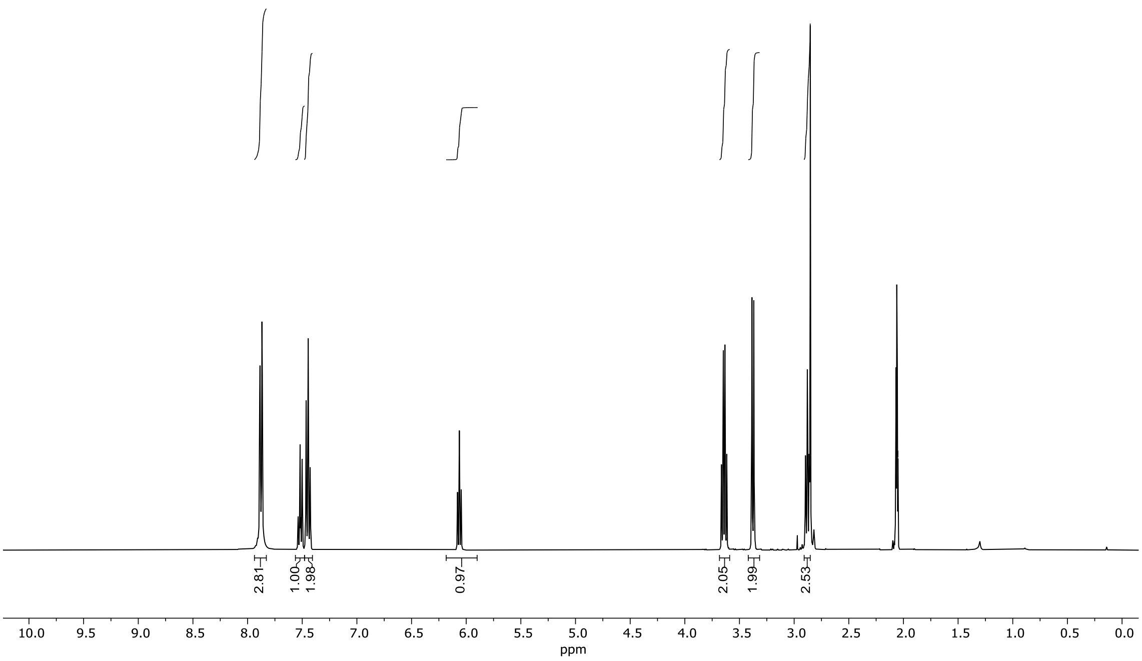

^{13}C-NMR (101MHz)

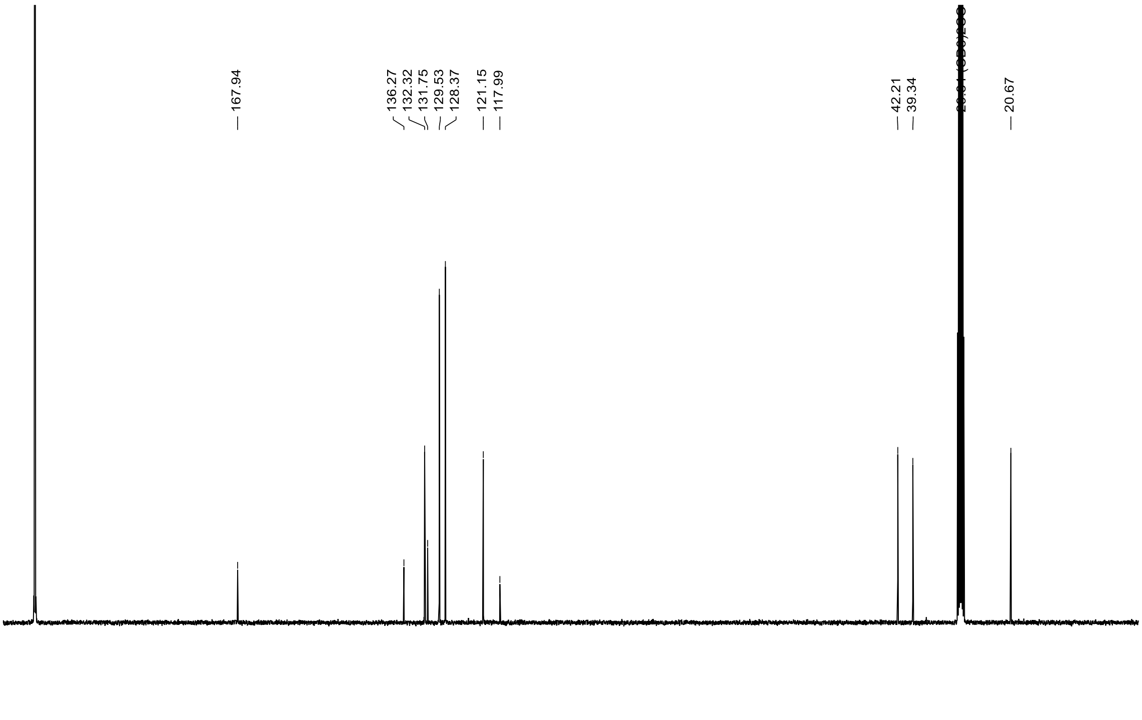

NOESY analysis for compound (*Z*)-*N*-(3-bromo-5-cyanopent-3-en-1-yl)benzamide (54)

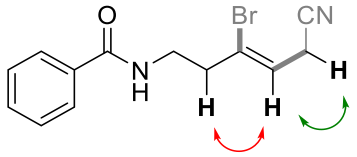

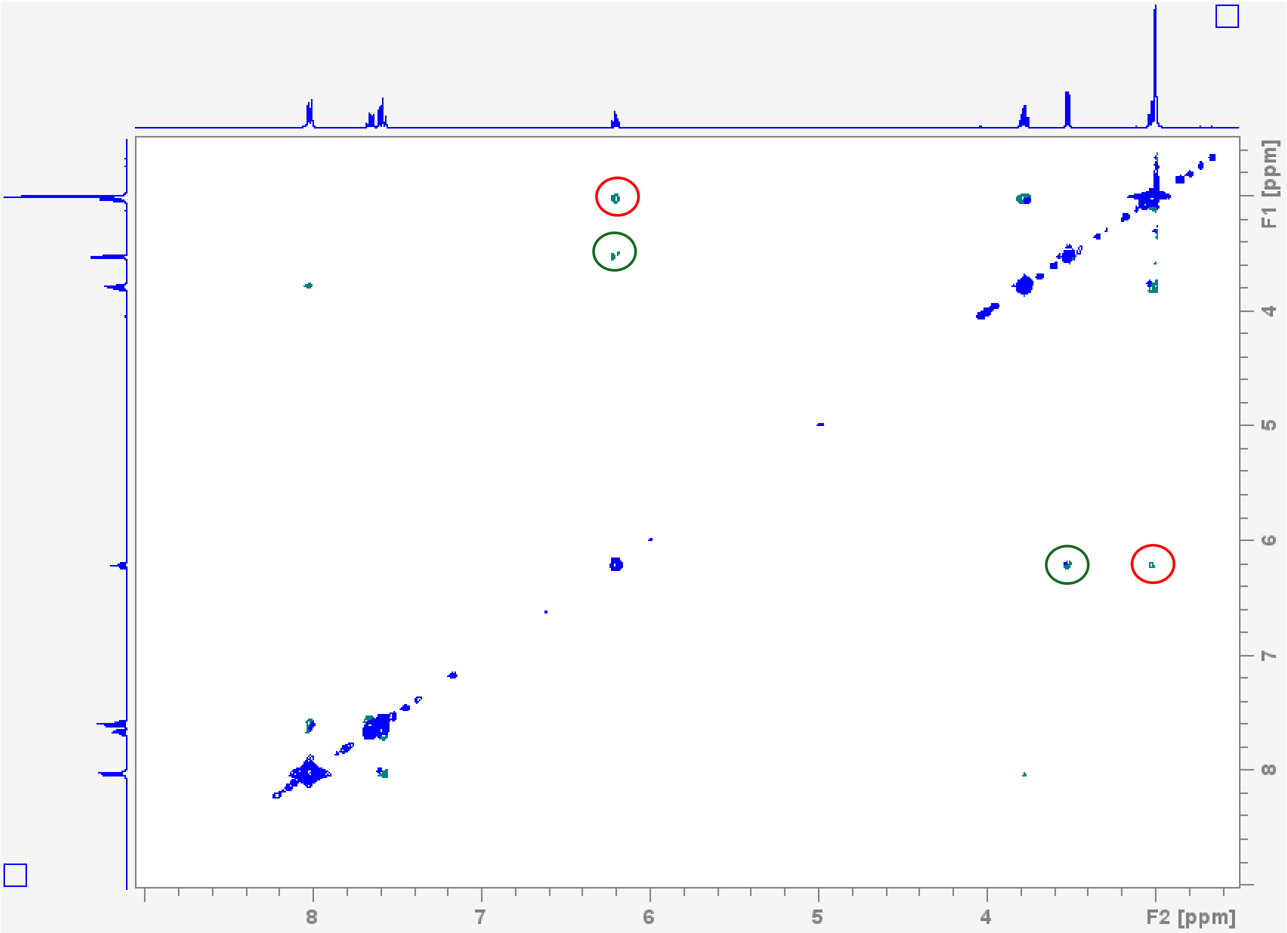

(*Z*)-*N*-(2-Bromo-4,4,4-trichlorobut-2-en-1-yl)-4-methylbenzenesulfonamide (55)

^{1}H-NMR (400MHz)

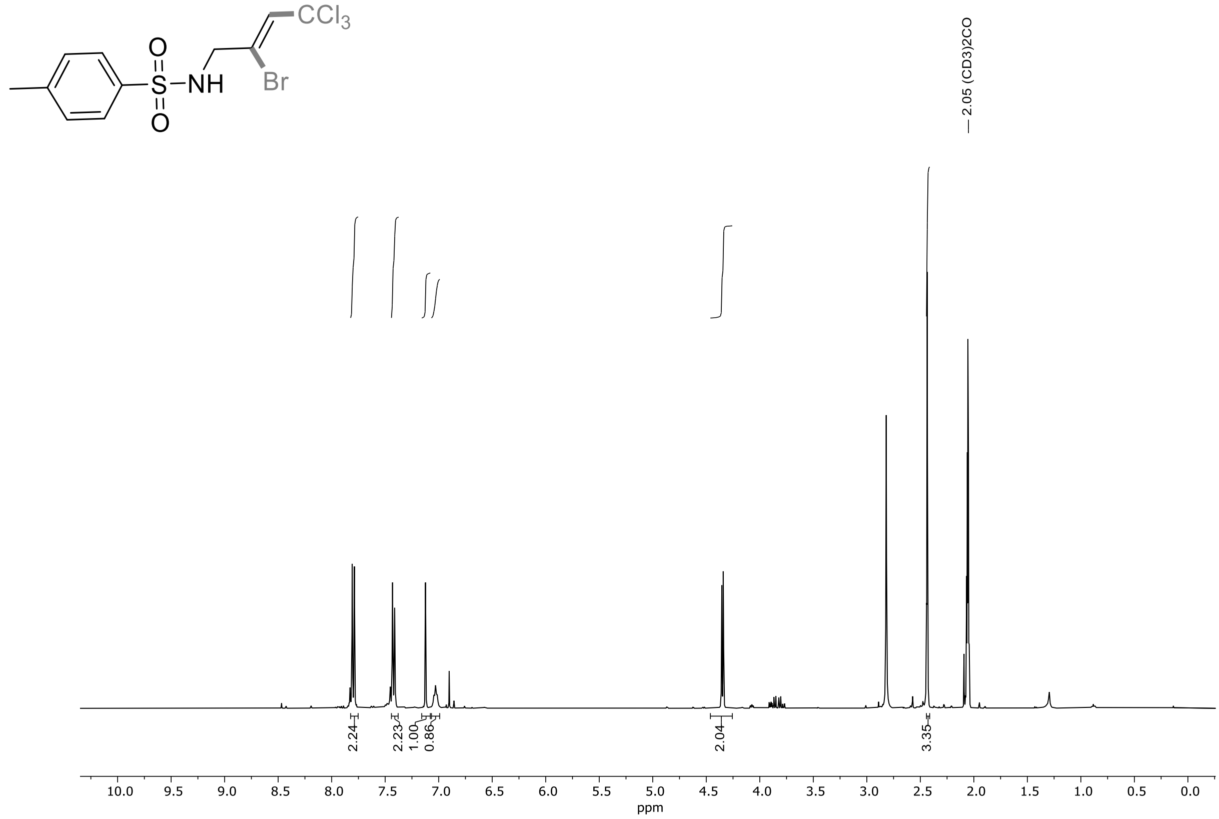

^{13}C-NMR (101MHz)

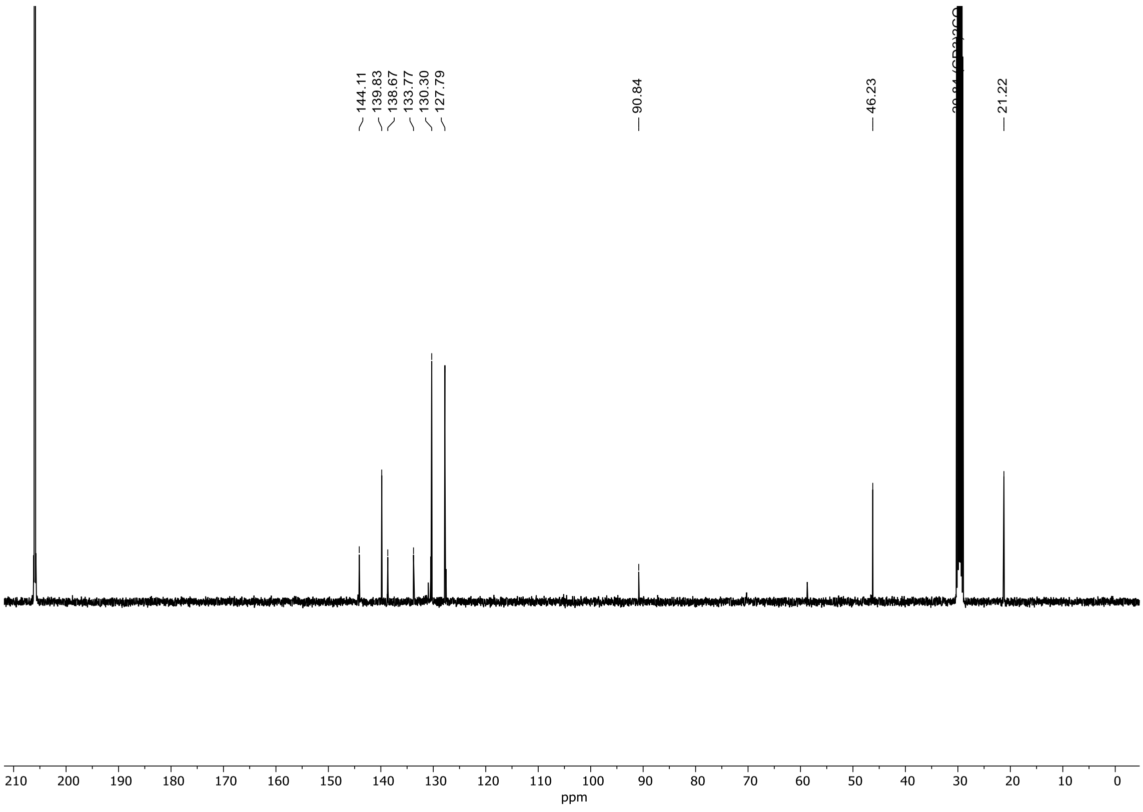

4-(2-Bromo-4,4,4-trichlorobutyl)-2-methoxyphenol (56)

^{1}H-NMR (400MHz)

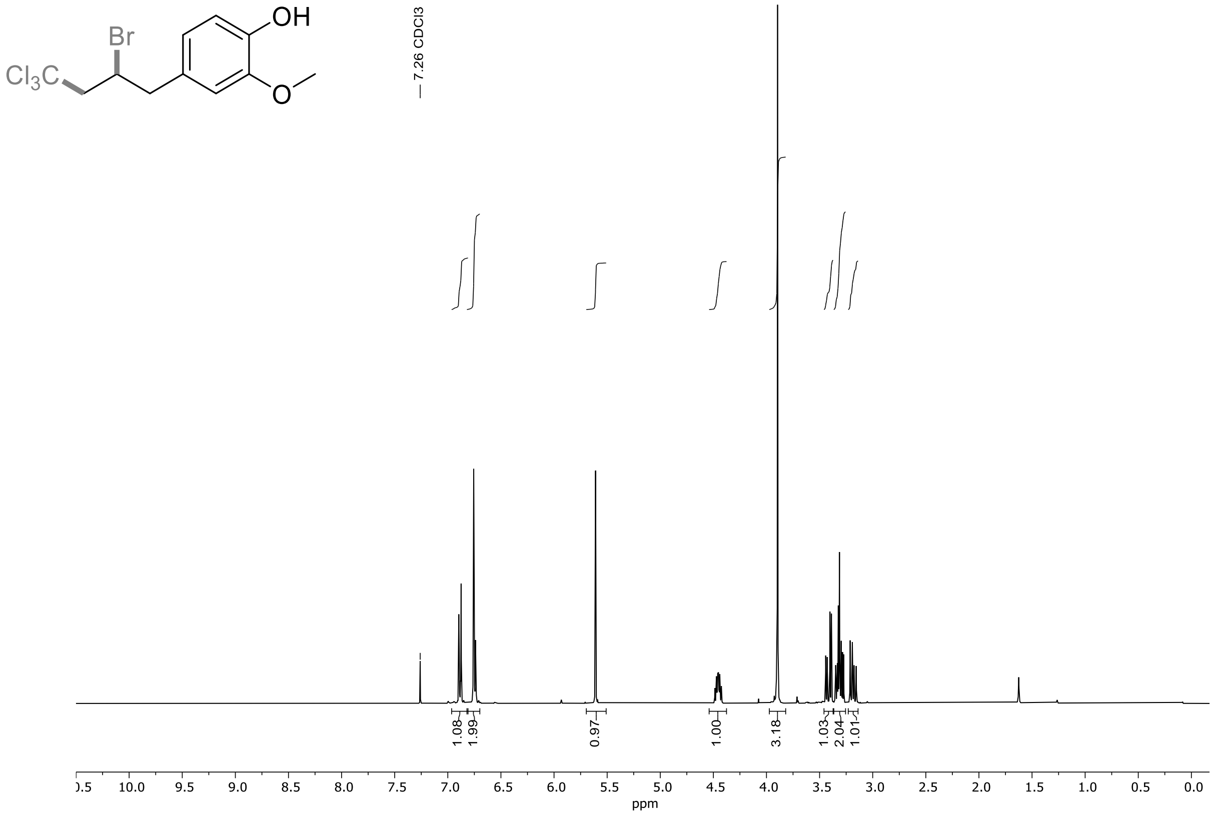

^{13}C-NMR (101MHz)

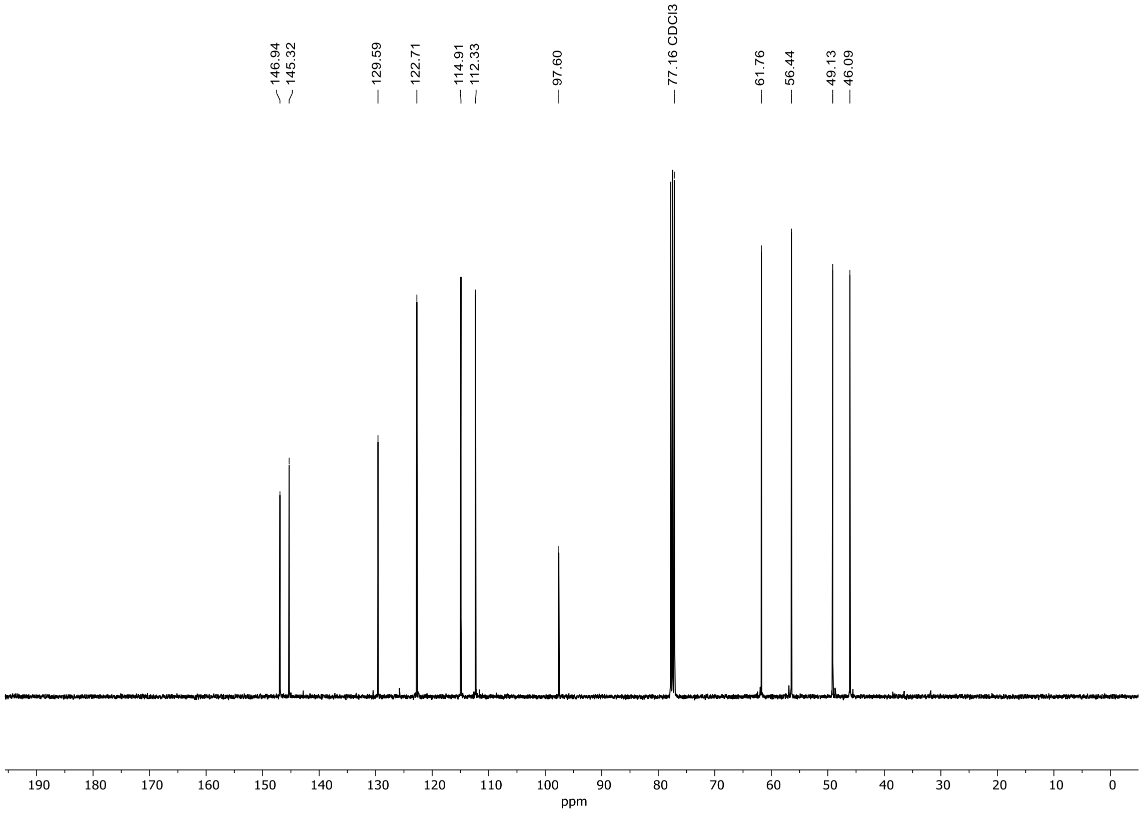

4-Bromo-5-(4-hydroxy-3-methoxyphenyl)pentanenitrile (57)

^{1}H-NMR (400MHz)

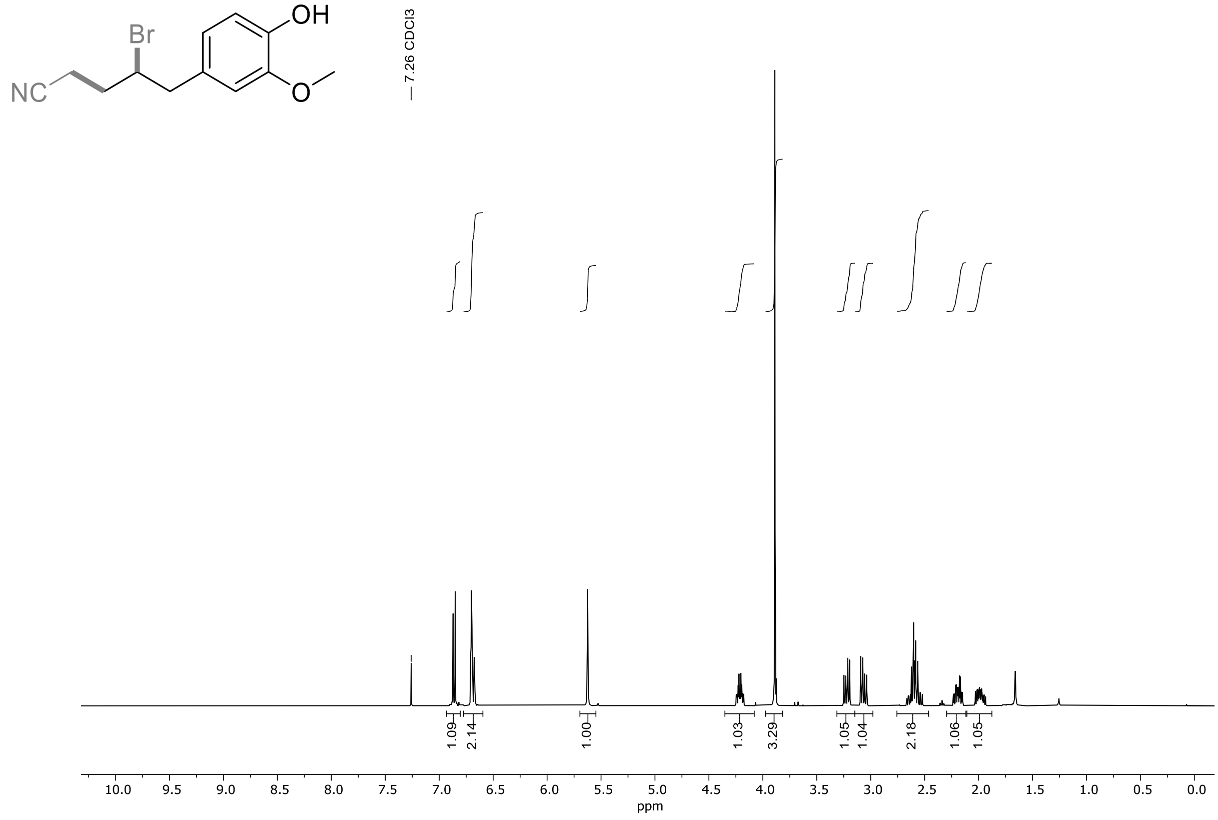

^{13}C-NMR (101MHz)

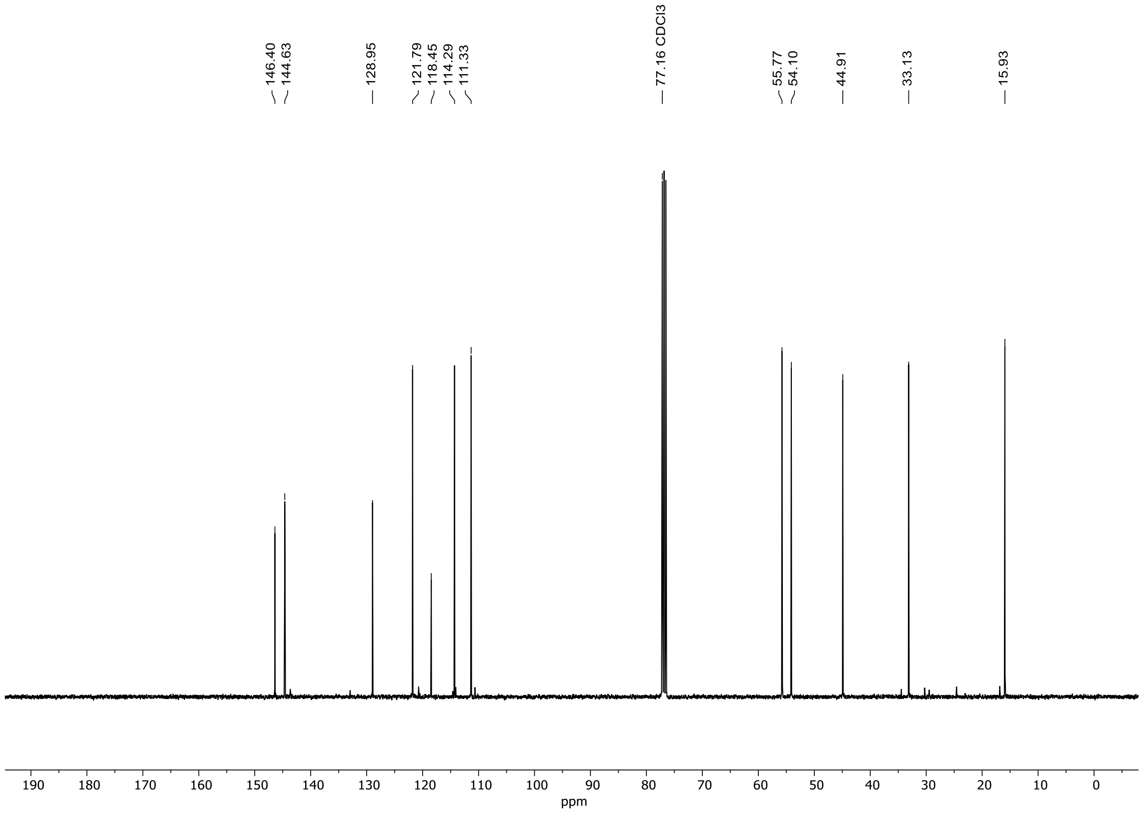

2-Bromo-4,4,4-trichlorobutoxy-1-isopropyl-4-methylcyclohexane (58)

^{1}H-NMR (400MHz)

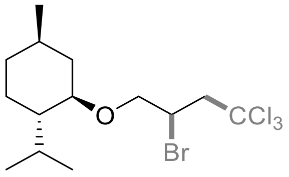

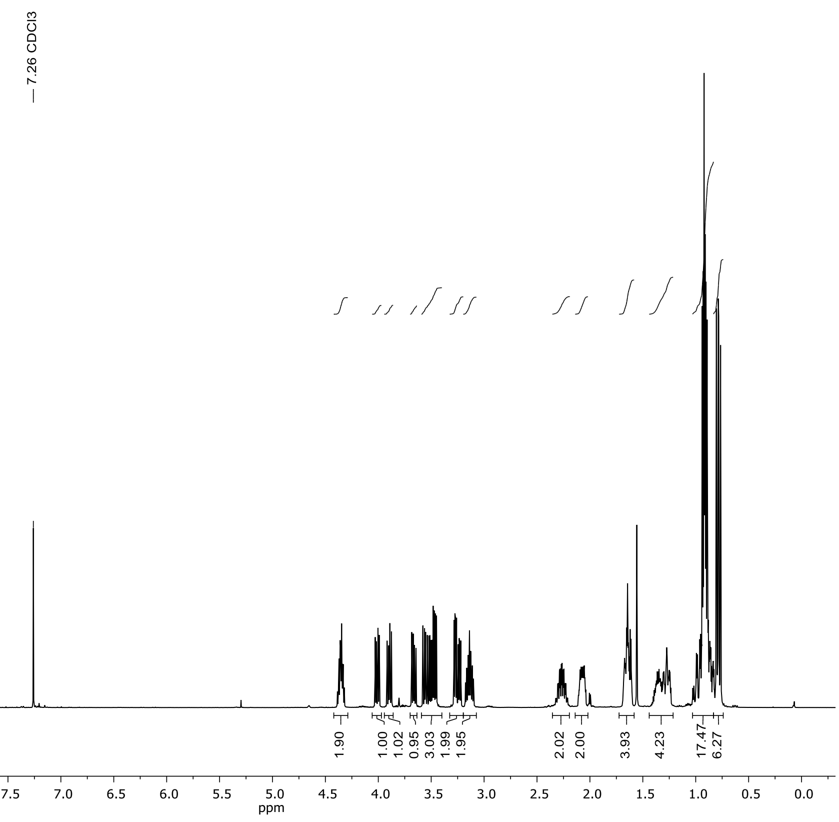

^{13}C-NMR (101MHz)

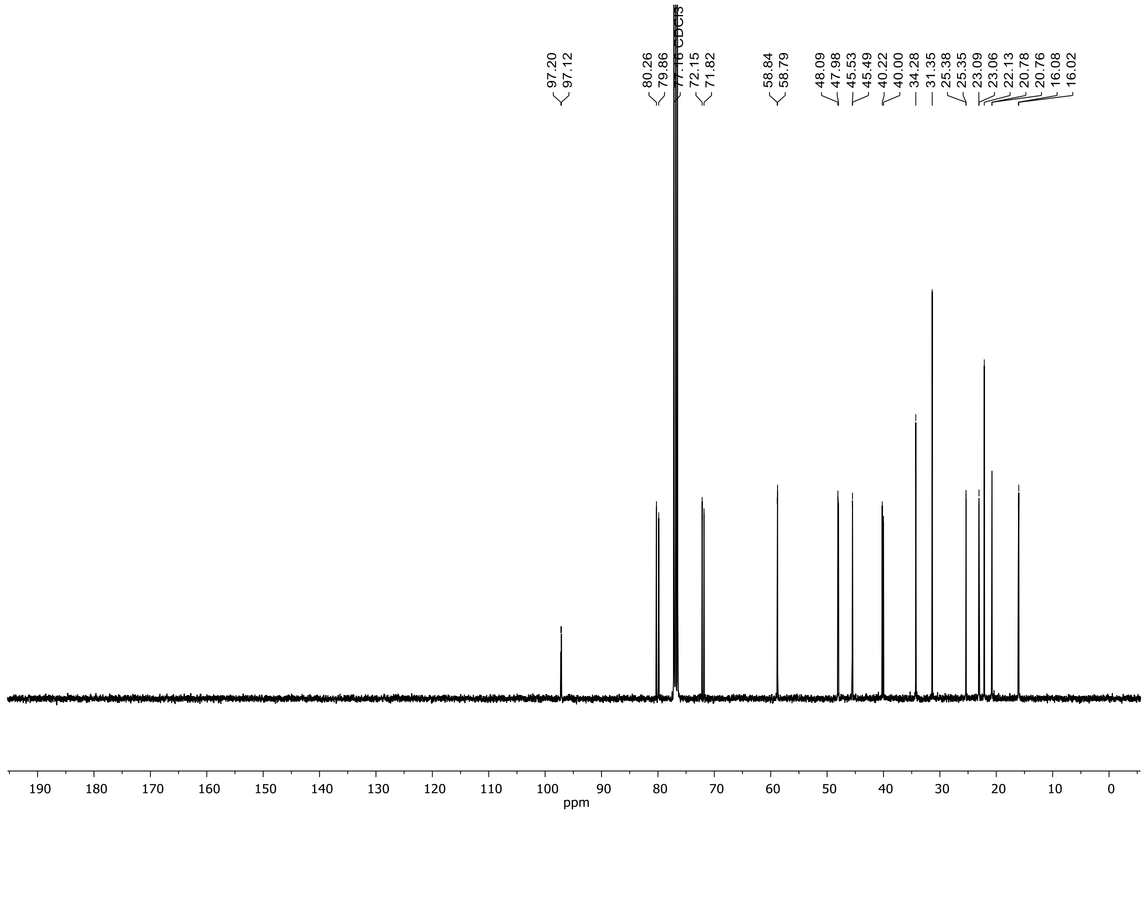

4-Bromo-5-((methylcyclohexyl)oxy)pentanenitrile (59)

¹H-NMR (400MHz)

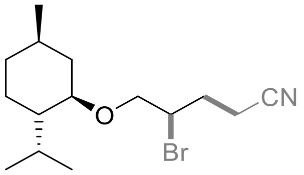

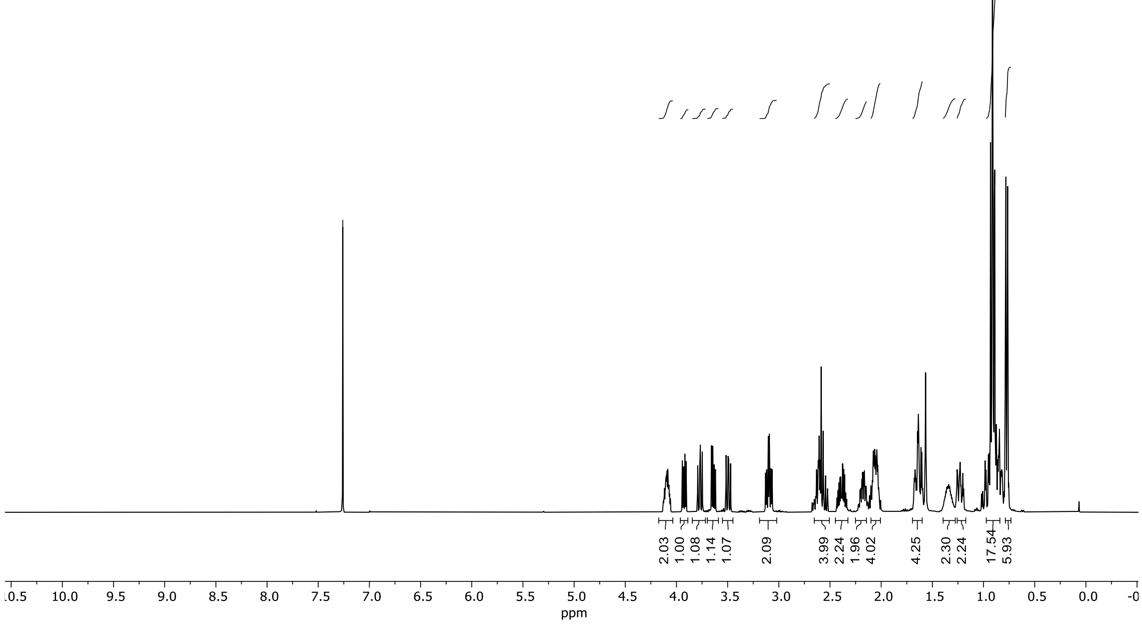

¹³C-NMR (101MHz)

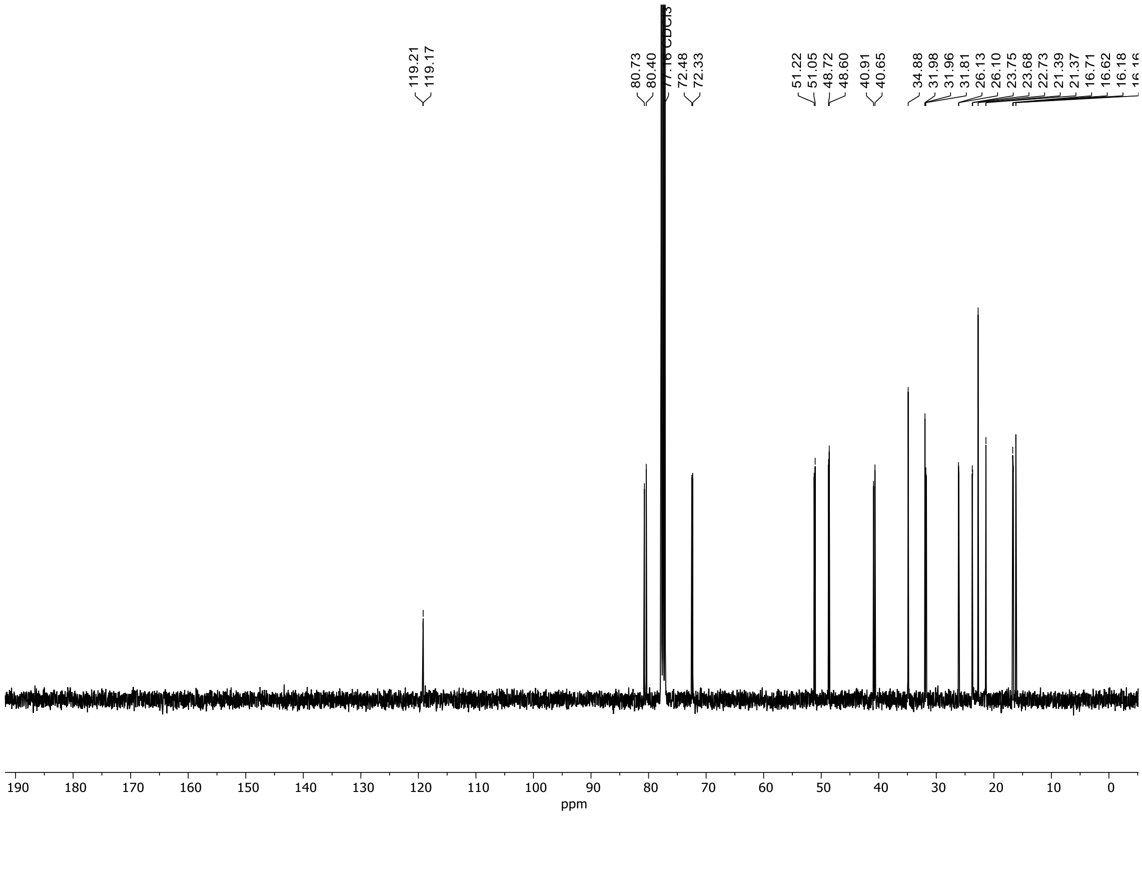

7-Bromo-6-(cyanomethyl)-3,7-dimethyloctyl benzoate (60)

^{1}H-NMR (400MHz)

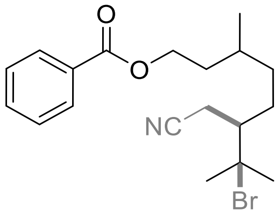

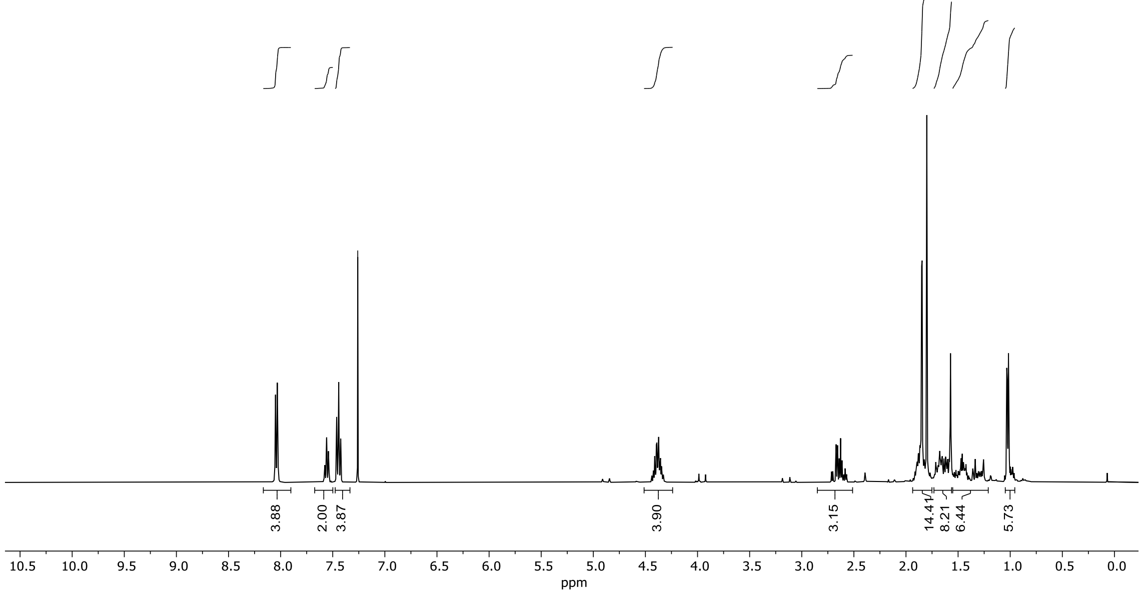

^{13}C-NMR (101MHz)

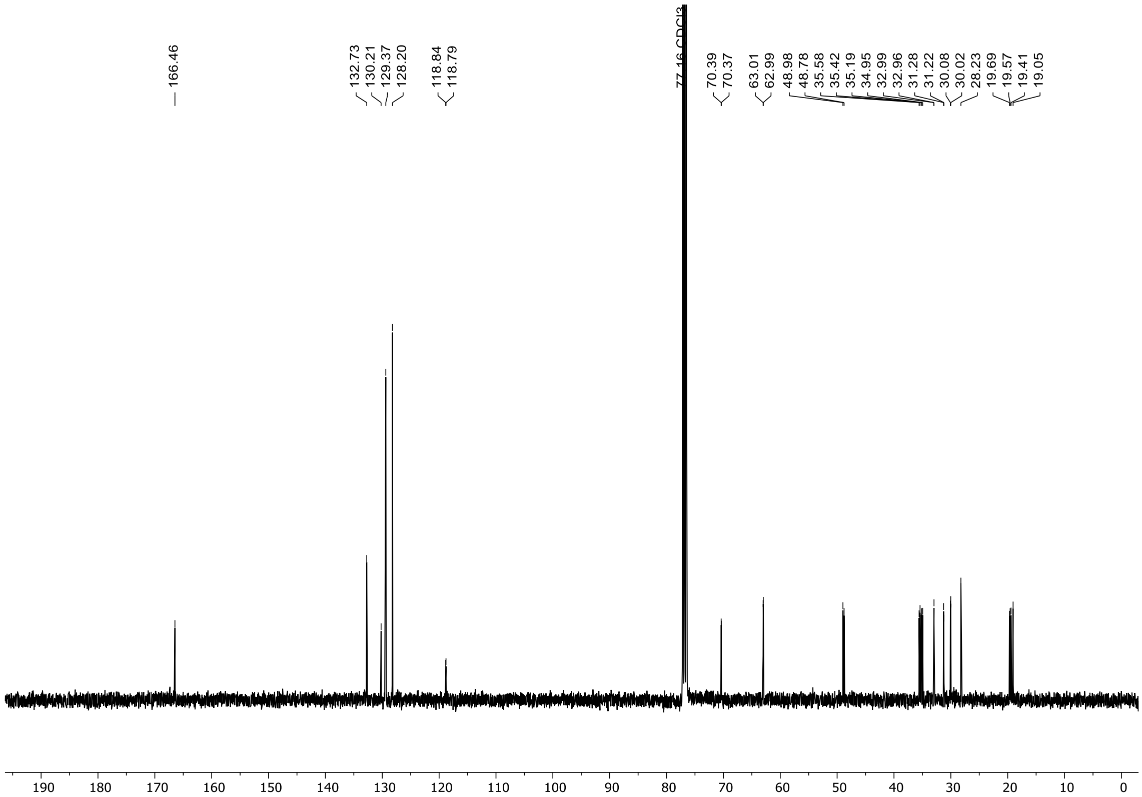

4-Bromo-6,6,6-trichlorohexyl 4-([1,1'-biphenyl]-4-yl)-4-oxobutanoate (61)

^{1}H-NMR (400MHz)

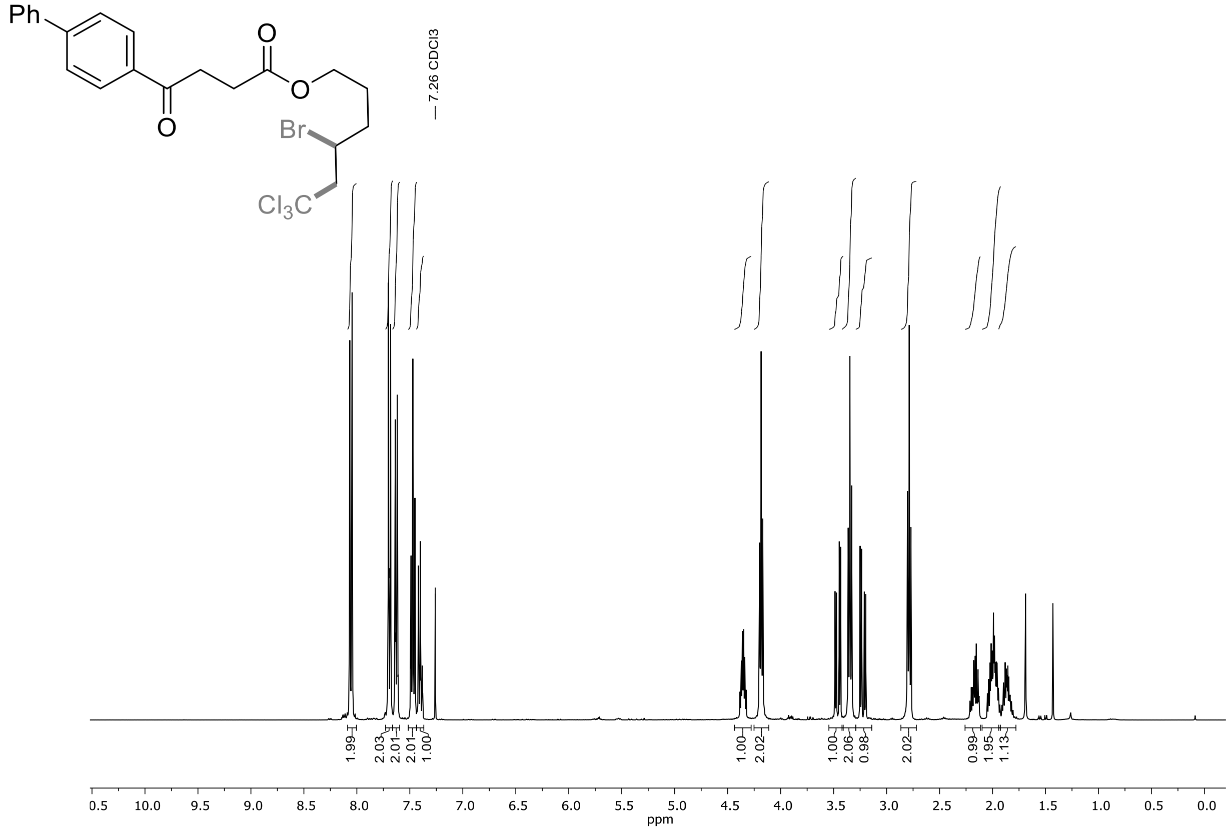

^{13}C-NMR (101MHz)

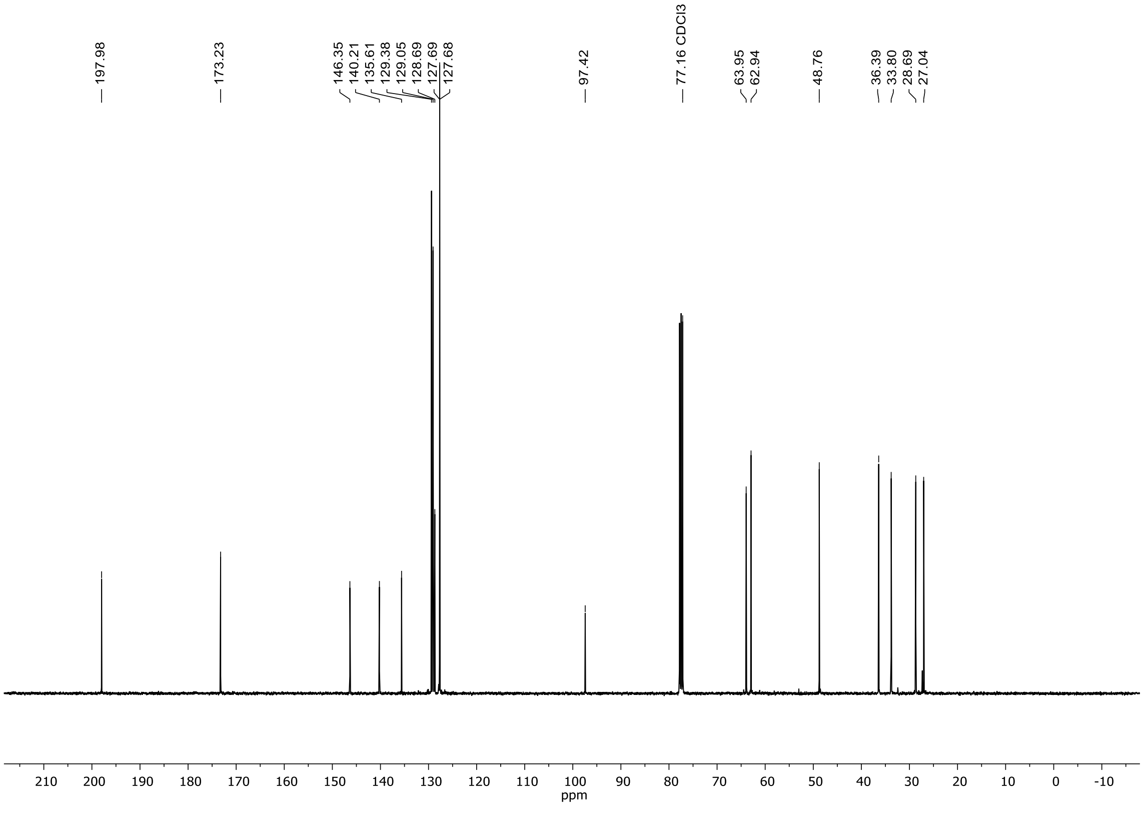

4-Bromo-6-cyanohexyl 4-([1,1’-biphenyl]-4-yl)-4-oxobutanoate (62)

^{1}H-NMR (400MHz)

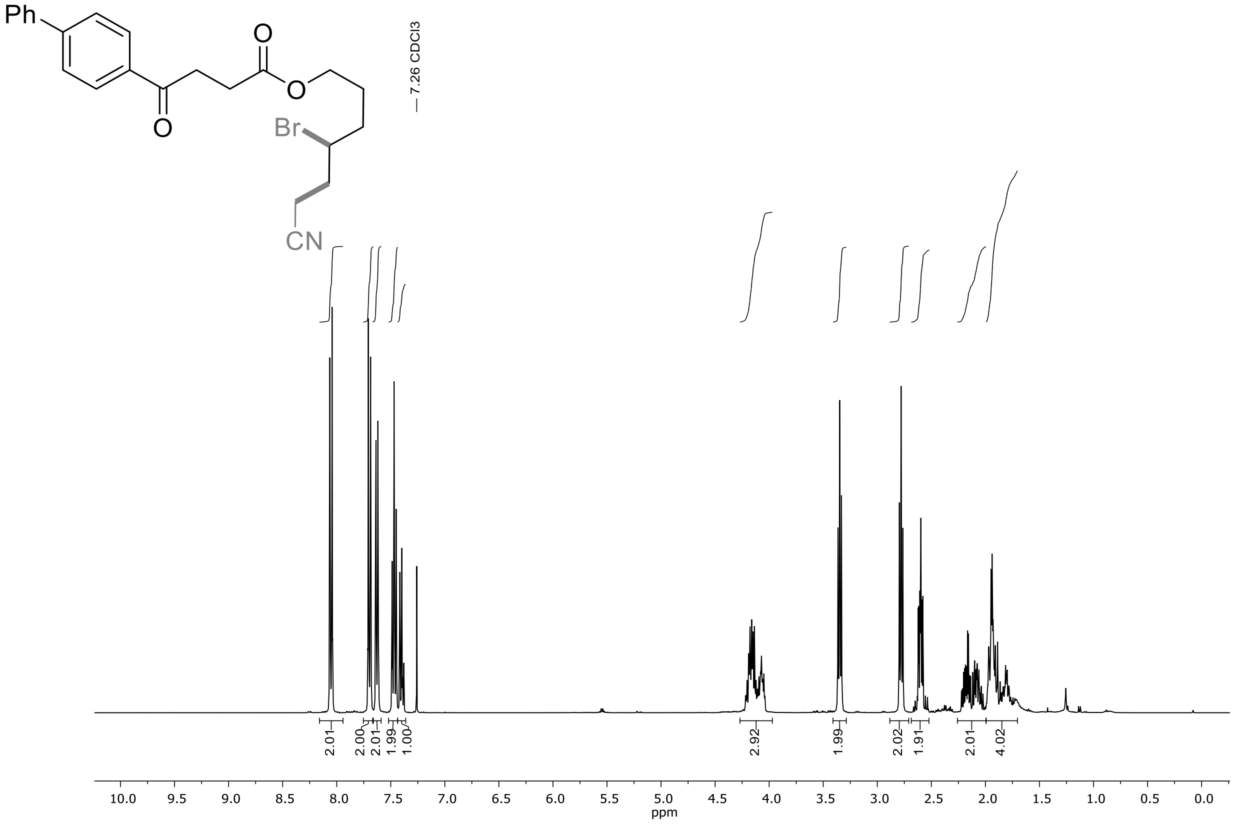

^{13}C-NMR (101MHz)

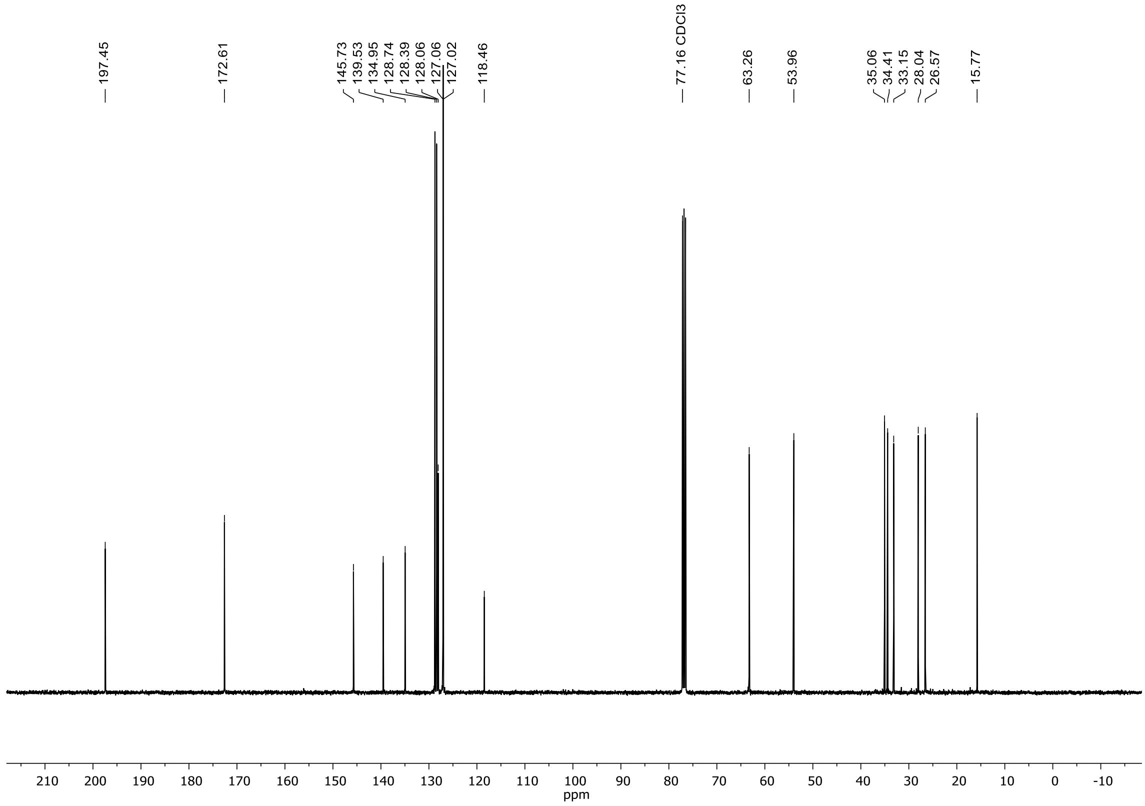

4-Bromo-6,6,6-trichlorohexyl 2-(4-isobutylphenyl)propanoate (63)

^{1}H-NMR (400MHz)

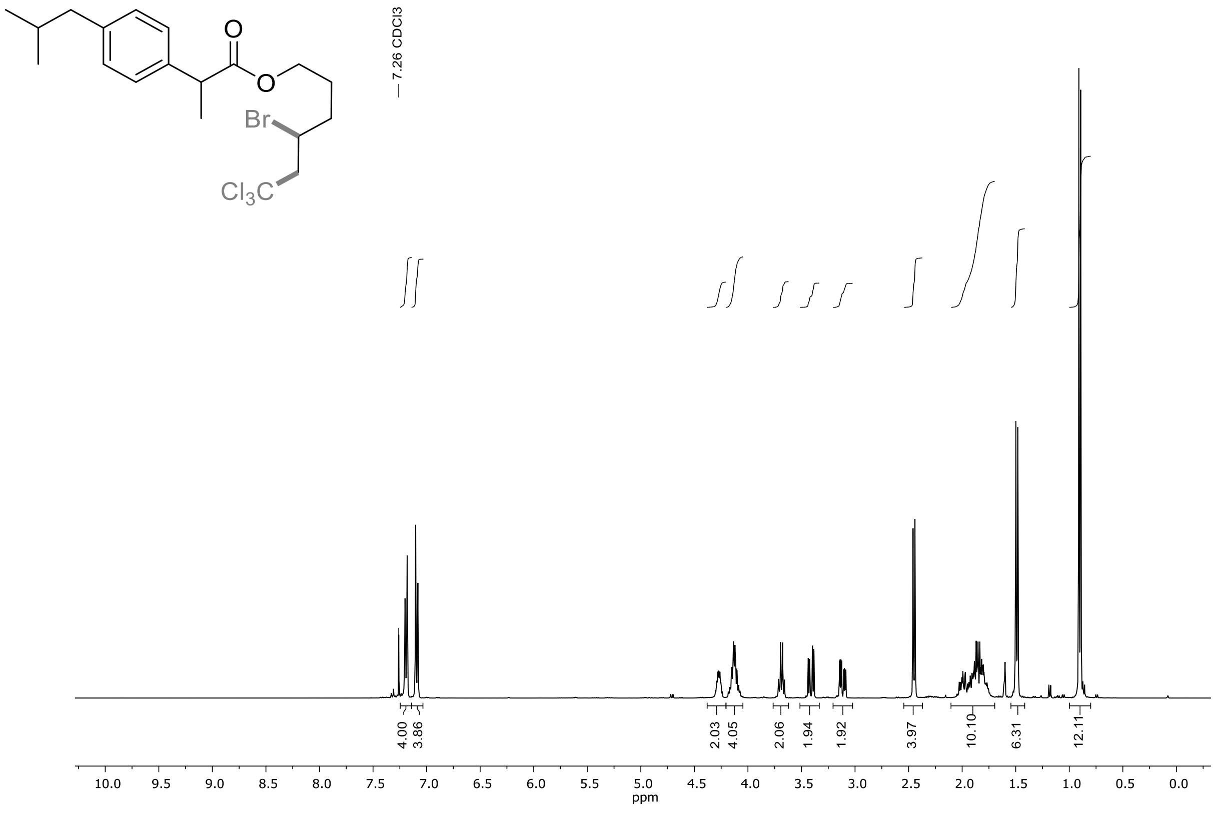

^{13}C-NMR (101MHz)

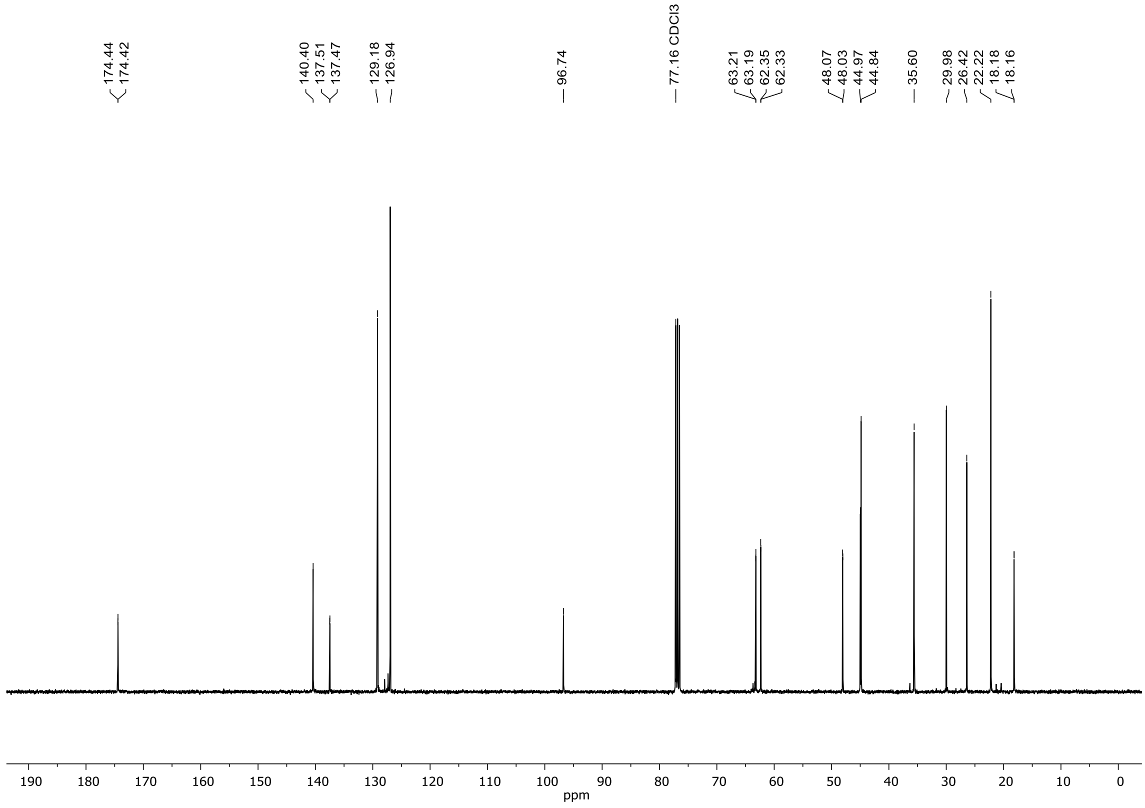

4-Bromo-6-cyanohexyl 2-(4-isobutylphenyl)propanoate (64)

^{1}H-NMR (400MHz)

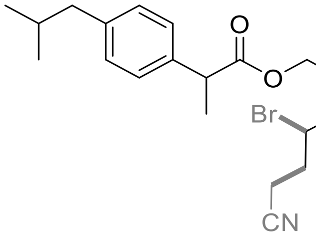

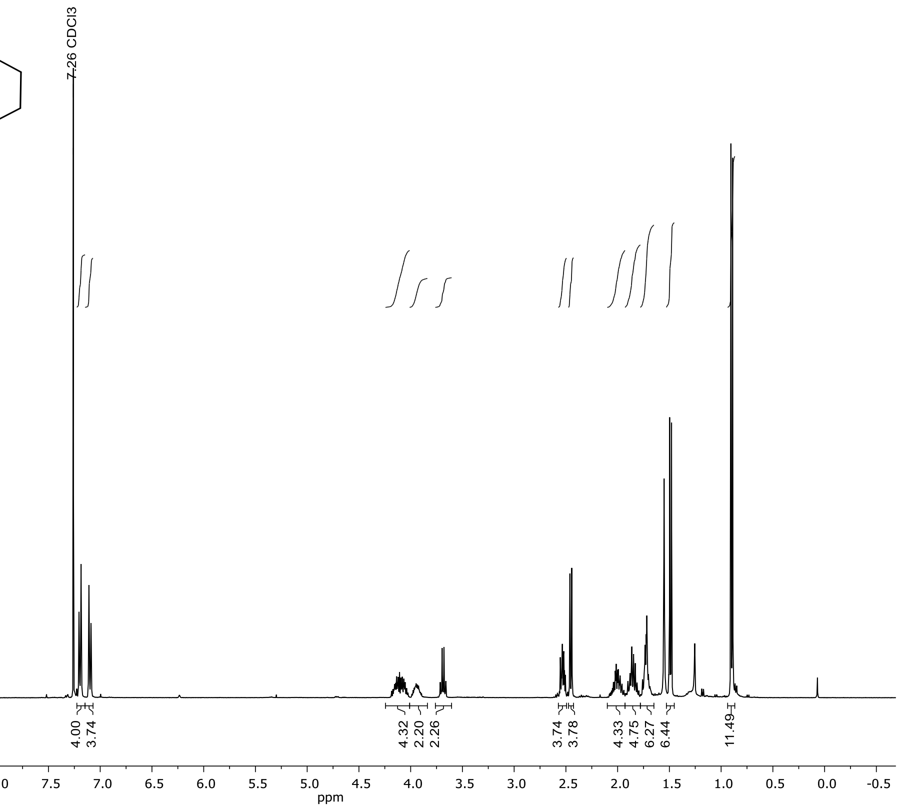

^{13}C-NMR (101MHz)

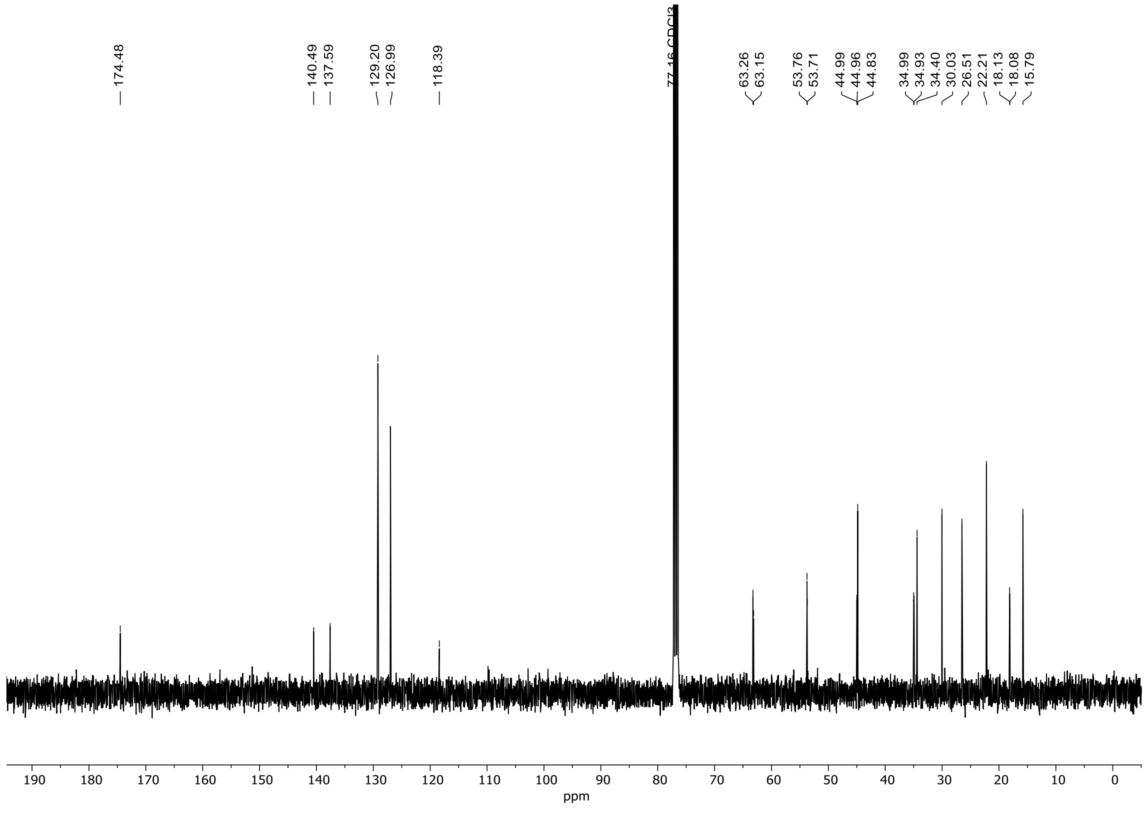

3-Bromo-2-(cyanomethyl)-3-methylbutylacetyl-L-phenylalaninate (65)

^{1}H-NMR (400MHz)

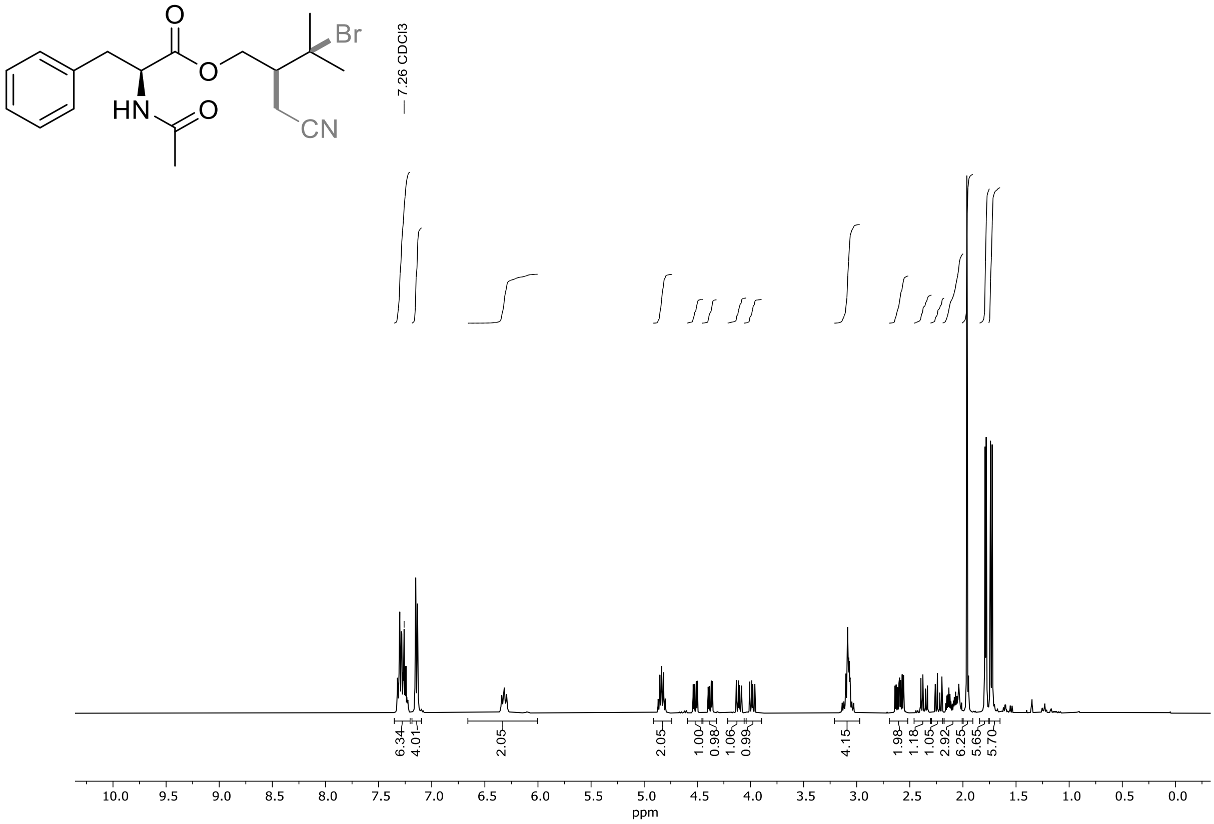

^{13}C-NMR (101MHz)

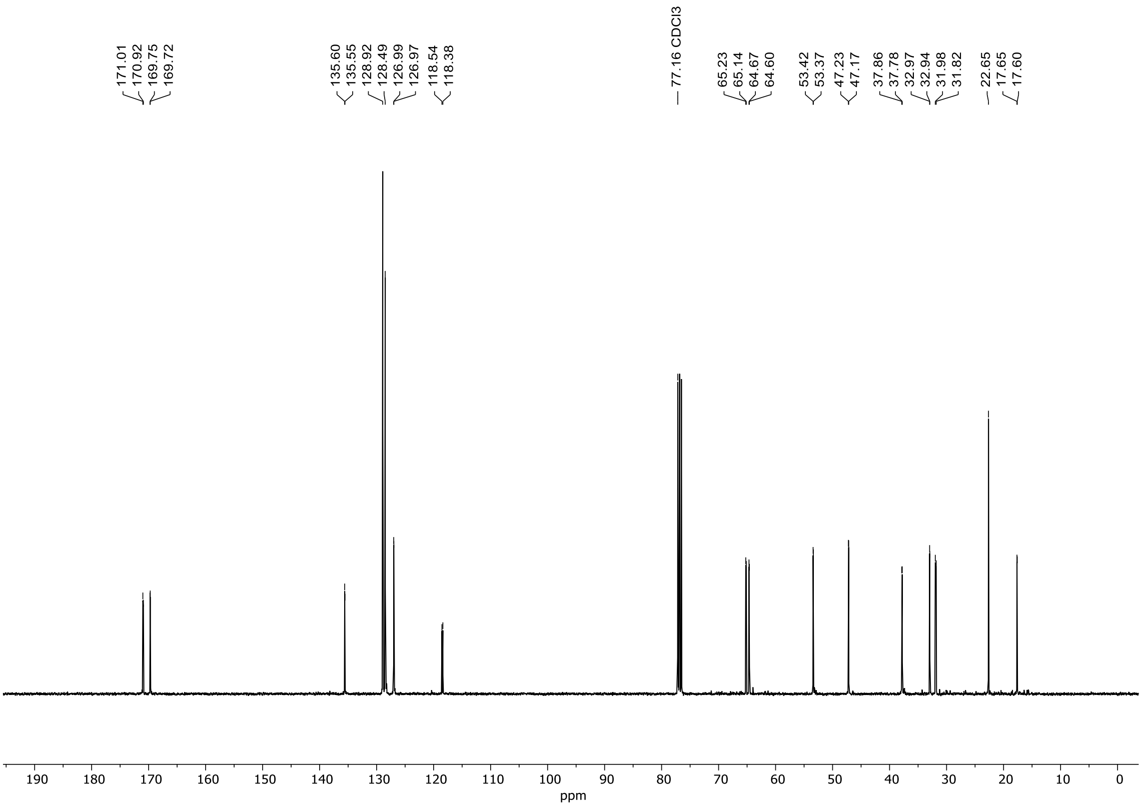

3-(Bromomethyl)-1-tosyl-4-(2,2,2-trichloroethyl)pyrrolidine (66)

^{1}H-NMR (400MHz)

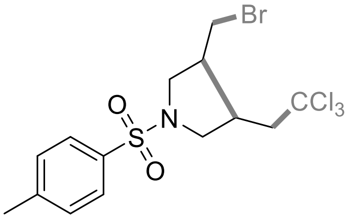

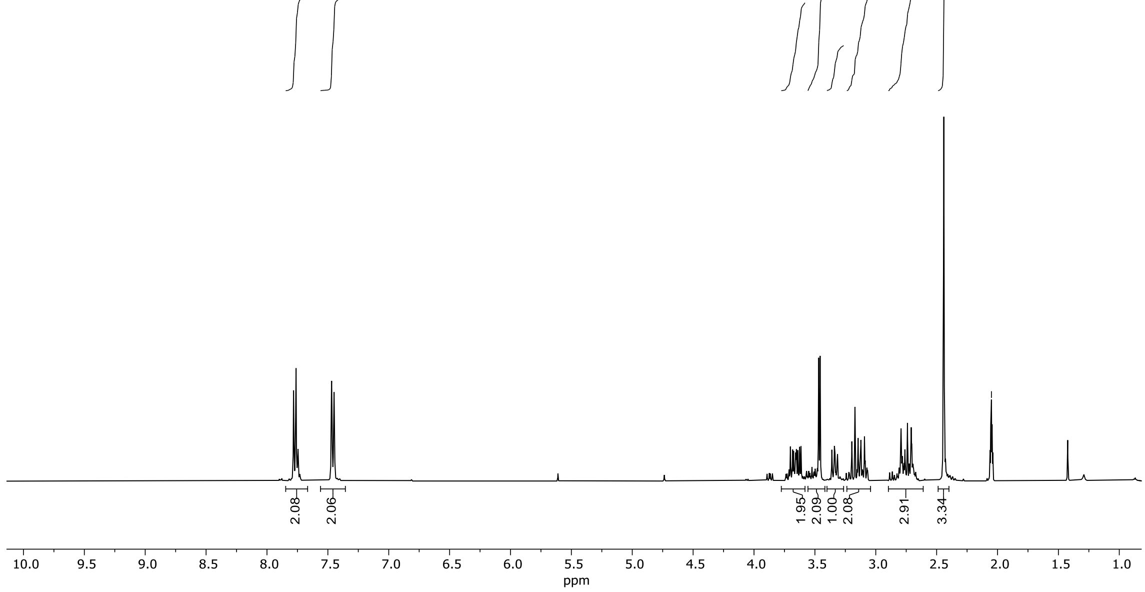

^{13}C-NMR (101MHz)

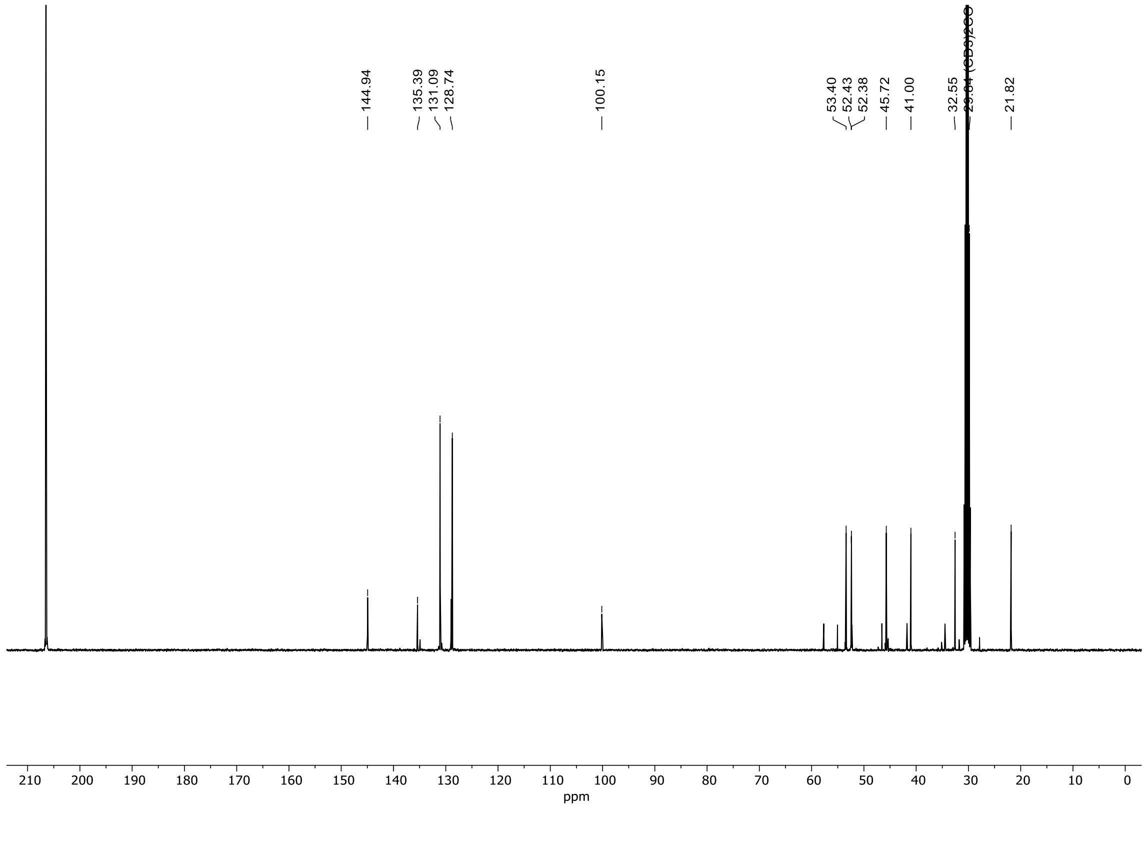

4-Azido-6-cyanohexyl benzoate (67)

^{1}H-NMR (400MHz)

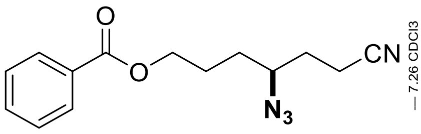

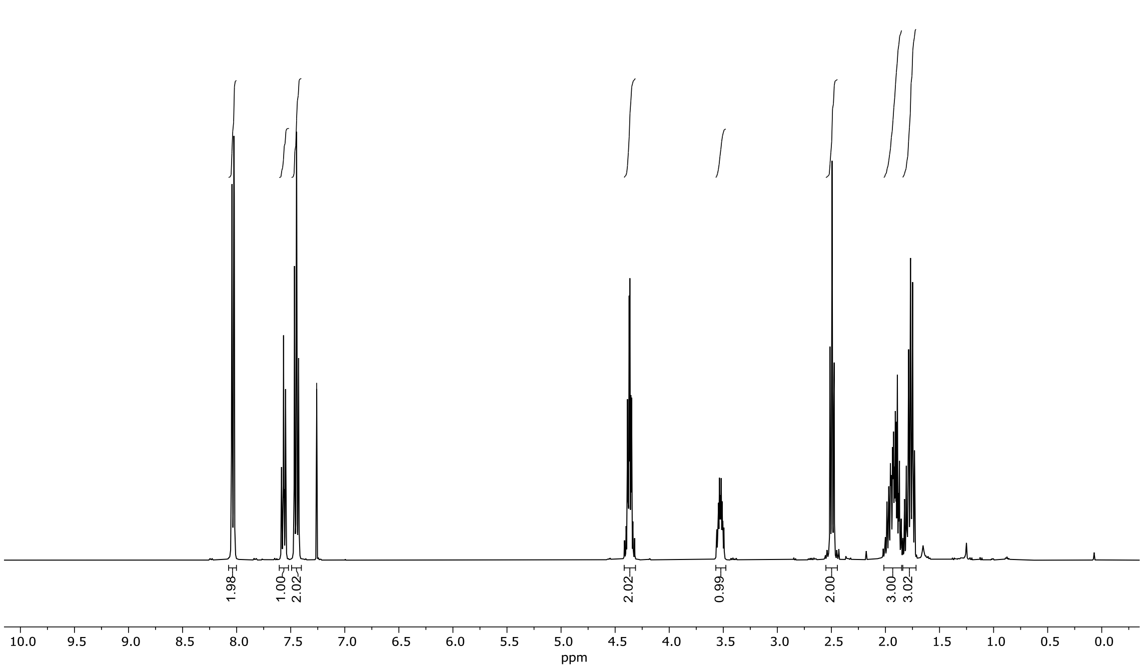

^{13}C-NMR (101MHz)

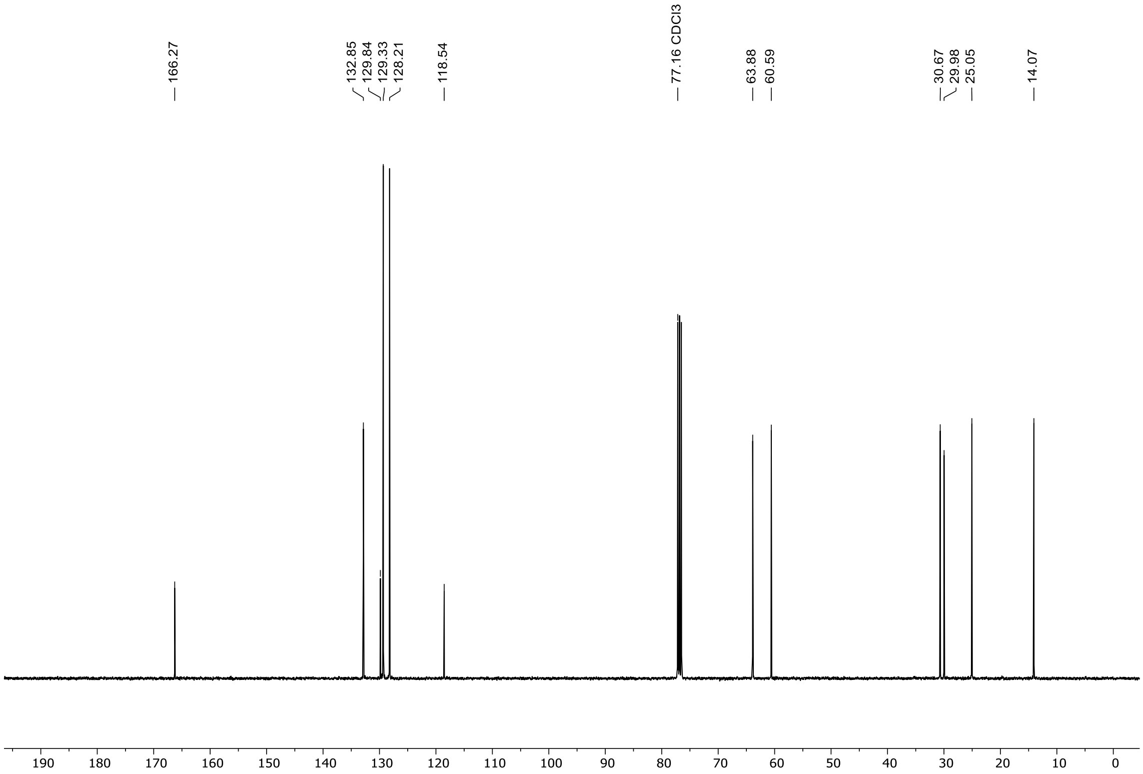

Supplement: Supplementary file 1 — ol5c00780_si_001.pdf [file ol5c00780_si_001.pdf]
